# A stereodivergent multicomponent approach for the synthesis of C–N atropisomeric peptide analogues†

**DOI:** 10.1039/d4sc04700a

**Published:** 2024-09-16

**Authors:** Natalie J. Roper, Aaron D. G. Campbell, Paul G. Waddell, Alistair K. Brown, Kristaps Ermanis, Roly J. Armstrong

**Affiliations:** a School of Natural and Environmental Sciences, Newcastle University Newcastle Upon Tyne NE1 7RU UK roly.armstrong@newcastle.ac.uk; b Biosciences Institute, Faculty of Medical Sciences, Newcastle University Newcastle Upon Tyne NE2 4HH UK; c School of Chemistry, University of Nottingham, University Park Nottingham NG7 2RD UK kristaps.ermanis@nottingham.ac.uk

## Abstract

A four-component Ugi reaction is described for the stereoselective synthesis of novel C–N atropisomeric peptide analogues. Using this approach, a combination of simple, readily available starting materials (*ortho*-substituted anilines, aldehydes, carboxylic acids and isocyanides) could be combined to access complex products possessing both central and axial chirality in up to 92% yield and >95 : 5 d.r. Variation of the reaction temperature enabled the development of stereodivergent reactions capable of selectively targeting either diastereoisomer of a desired product from a single set of starting materials with high levels of stereocontrol. Detailed experimental and computational studies have been performed to probe the reaction mechanism and stereochemical outcome of these reactions. Preliminary studies show that novel atropisomeric scaffolds prepared using this method display inhibitory activity against *M. tuberculosis* with a significant difference in activity observed between different atropisomers.

## Abstract 2

Four-component reactions at room temperature delivered C–N atropisomeric peptide analogues possessing both central and axial chirality with complete diastereocontrol. Reactions at elevated temperature selectively afforded the other diastereoisomer.

## Graphical abstract

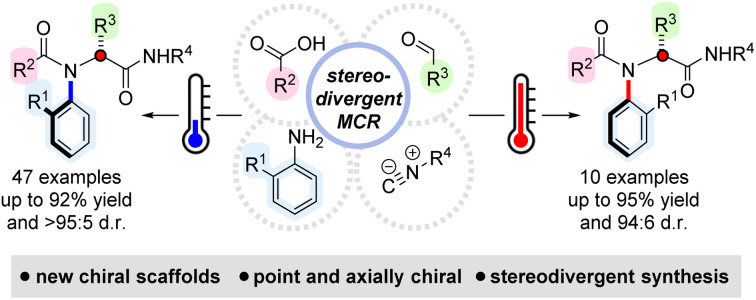

## Introduction

**Scheme 1:**
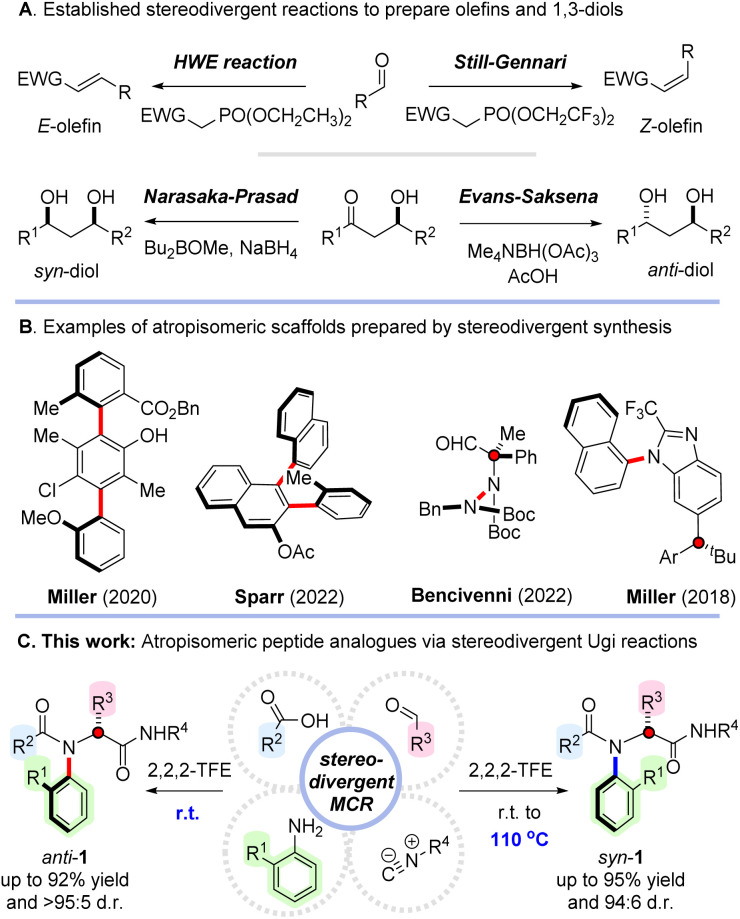
Previous work and strategy for stereodivergent multicomponent synthesis of C–N atropisomeric peptide analogues.

Recent advances in stereoselective synthesis have led to a wealth of processes enabling the synthesis of materials with precisely controlled relative stereochemistry.^1^ In the majority of cases, diastereoselectivity in such reactions is governed by substrate control in which an existing element of chirality guides the stereochemical outcome of the reaction. However, in the event that a different diastereoisomer is required it can be extremely challenging to override this innate selectivity. Much less common are diastereodivergent reactions capable of selectively converting a common precursor to either diastereoisomer of a desired target.^2^ For example, elegant methods have been established for diastereodivergent olefination of aldehydes to afford either *E*- or *Z*-configured alkenes, along with powerful diastereodivergent reduction processes enabling the synthesis of 1,3-diols (Scheme 1A).^3,4^ The flexibility of such reactions to selectively and predictably deliver different stereoisomers on demand is hugely valuable, particularly in drug discovery, where subtle configurational changes can dramatically influence biological activity.^5^

The synthesis of atropisomeric materials is an area that is rapidly gaining traction owing to their emerging applications in materials science, catalysis, and medicine.^6^ An array of powerful synthetic methods have been reported which enable the construction of scaffolds displaying restricted rotation about C–C, C–N and N–N bonds.^6,7^ However, the preparation of atropisomeric molecules that contain an additional element of stereogenicity is significantly complicated by the potential for formation of multiple diastereoisomers.^8^ Comparatively few stereoselective methods are available for the synthesis of such molecules, and only a handful of approaches capable of diastereodivergence have been reported.^2*b*,9,10^ In particular, the groups of Miller^10*a*^ and Sparr^10*b*^ have independently disclosed elegant stereodivergent syntheses of molecules containing multiple stereogenic C–C axes and Bencivenni^10*c*^ and Miller^10*d*^ have reported efficient stereodivergent syntheses of materials combining central chirality with atropisomerism about N–N and C–N bonds respectively (Scheme 1B). Despite these important innovations, selectively accessing scaffolds in which atropisomerism is combined with an additional element of stereochemistry remains a significant challenge.

Given these considerations, we recently questioned whether it might be possible to prepare the first examples of C–N atropisomeric peptide analogues 1 possessing both central and axial chirality (Scheme 1C).^11–14^ Given the ubiquitous nature of peptides in pharmaceuticals and agrochemicals, the idea of developing C–N atropisomeric analogues would be of significant interest and we proposed that it might be possible to directly access them *via* a stereoselective Ugi reaction between *ortho*-substituted anilines, aldehydes, carboxylic acids and isocyanides.^15^ This approach has the potential to provide access to these novel scaffolds with unparalleled efficiency. However, we recognized that to prove successful, several important challenges would need to be overcome, namely: (i) identification of mild conditions capable of engaging extremely hindered aniline reagents whilst avoiding competing Passerini side reactions; (ii) simultaneously achieving high levels of stereoselectivity; (iii) discovering a complementary method to access the alternative atropisomer of the peptidic products. Here we describe our results in this area and how we were ultimately able to develop efficient stereodivergent multicomponent reactions that allow a single set of starting materials to be converted to either *syn*- or *anti*-configured atropisomeric peptide analogues with excellent efficiency, broad scope, and near complete stereocontrol.

## Results and discussion

We commenced our study by evaluating a room-temperature multicomponent reaction between an equimolar mixture of 2-*tert*-butylaniline, benzoic acid, benzaldehyde and *tert*-butyl isocyanide. Disappointingly, under typical Ugi conditions employing MeOH (0.2 M) as the reaction solvent, only trace conversion to the desired *N*-arylated amide was observed (Table 1, entry 1). This is likely a consequence of the extremely hindered nature of the aniline reagent. However, tantalizingly the trace amount of *anti*-1a formed in this reaction appeared to be present as a single diastereoisomer. In an attempt to boost conversion, we explored introducing various additives which have been shown to accelerate other challenging Ugi reactions.^16^ Brønsted acids as well as scandium and titanium-based Lewis acids all gave comparable or inferior results (Table 1, entries 2–5). However, upon addition of 0.1 equivalent of MgCl_2_ the desired product was formed in 21% yield and >95 : 5 d.r. (Table 1, entry 6). Employing MgCl_2_ as the Lewis acid, we then explored the effect of varying the reaction solvent. Poor results were observed in aprotic solvents such as dichloromethane and acetonitrile (entries 7 and 8), but we were pleased to find that fluorinated alcohol solvents were particularly effective for this transformation, with 71% yield and >95 : 5 d.r. obtained in 2,2,2-trifluoroethanol (2,2,2-TFE) (Table 1, entries 9 and 10).^17^ A control experiment performed in the absence of MgCl_2_ revealed that the Lewis acid additive was no longer beneficial in 2,2,2-TFE, affording the desired product in an improved yield of 82% (Table 1, entry 11). Finally, we found that increasing the concentration to 0.5 M was also beneficial, allowing us to isolate *anti*-1a in 92% yield as a single diastereoisomer (Table 1, entry 12). We were fortunate to be able to obtain crystals of *anti*-1a suitable for single crystal X-ray diffraction, which enabled us to unambiguously assign the relative stereochemistry of the major diastereoisomer as *anti*.

**Table 1:** Optimization of diastereoselective four-component coupling. Reaction conditions: (i) 2-*tert*-butylaniline (1 eq.), benzoic acid (1 eq.), benzaldehyde (1 eq.), *tert*-butyl isocyanide (1 eq.), r.t., 24 h

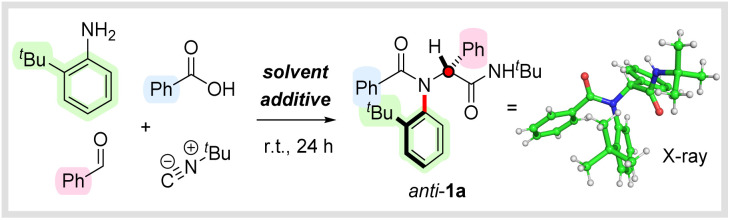				
Entry	Solvent	Additive (0.1 eq.)	Yield *anti*-1a^a^/%	d.r. *anti*-1a^b^
1	MeOH (0.2 M)	—	8	>95 : 5
2	MeOH (0.2 M)	*p*-TSA	<5	—
3	MeOH (0.2 M)	HCl^c^	5	>95 : 5
4	MeOH (0.2 M)	Sc(OTf)_3_	<5	—
5	MeOH (0.2 M)	Ti(O^i^Pr)_4_	13	>95 : 5
6	MeOH (0.2 M)	MgCl_2_	21	>95 : 5
7	CH_2_Cl_2_ (0.2 M)	MgCl_2_	<5	—
8	MeCN (0.2 M)	MgCl_2_	11	>95 : 5
9	HFIP (0.2 M)	MgCl_2_	62	>95 : 5
10	2,2,2-TFE (0.2 M)	MgCl_2_	71	>95 : 5
11	2,2,2-TFE (0.2 M)	—	82	>95 : 5
12	2,2,2-TFE (0.5 M)	—	90 (92)	>95 : 5

a Determined by ^1^H NMR analysis *vs.* CHCl_2_CHCl_2_ as an internal standard; values in parentheses indicate the yield of isolated product.

b Diastereoselectivity determined by ^1^H NMR analysis of the crude reaction mixture.

c HCl was added as a 2 M solution in diethyl ether.

**Scheme 2:**
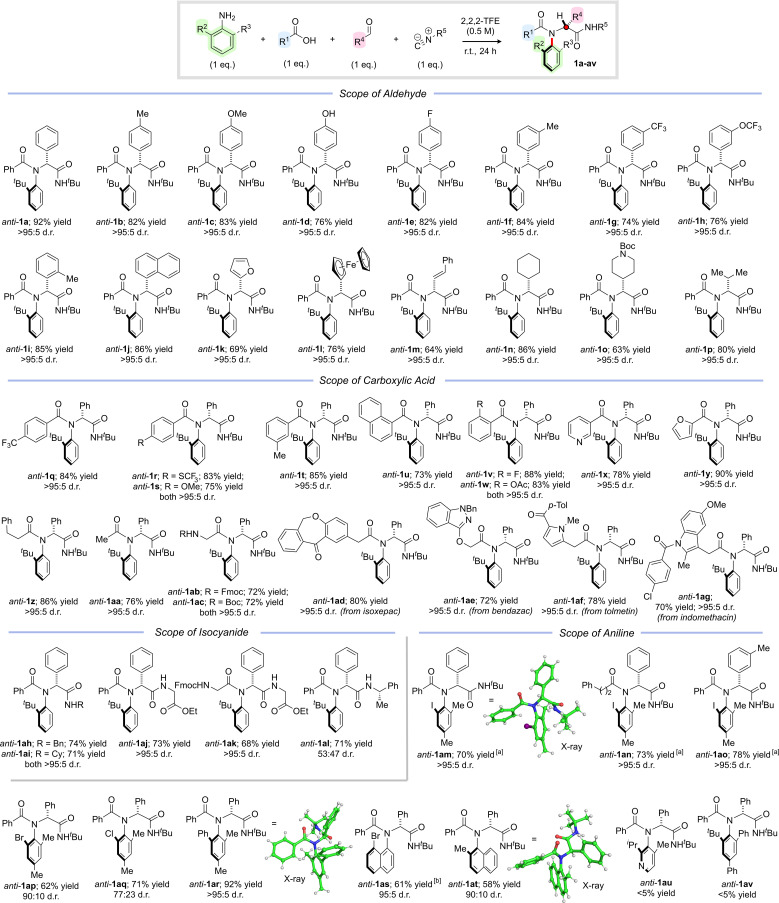
Stereoselective multicomponent synthesis of C–N atropisomeric amides. Reaction conditions: *ortho*-substituted aniline (1 eq.), aldehyde (1 eq.), carboxylic acid (1 eq.), isocyanide (1 eq.), 2,2,2-trifluoroethanol (0.5 M), r.t., 24 h. Yields refer to isolated material after column chromatography. Diastereoselectivity determined by ^1^H NMR analysis of the crude reaction mixture. ^*a*^An additional portion of isocyanide (1 eq.) was added after 24 h and the reaction was stirred for a further 24 h. ^*b*^Relative configuration assigned on the basis of a crystal structure of *syn*-1as (see Scheme 5 and ESI†).

With optimal conditions established to access *anti*-configured C–N atropisomeric peptide analogues, we set out to establish the generality of the method, initially focusing on the aldehyde partner (Scheme 2). We were pleased to find that a variety of aromatic aldehydes were well tolerated, delivering products *anti*-1a–l in uniformly high yields and with complete diastereocontrol, including examples containing unprotected phenols (*anti*-1d), sterically encumbered groups (*anti*-1i–j), heterocycles (*anti*-1k) and metallocenes (*anti*-1l). The chemistry was not restricted to aromatic aldehydes, and a series of vinylic, aliphatic, and saturated heterocyclic products (*anti*-1m–p) were isolated in excellent yields with >95 : 5 d.r. We next investigated the scope of the carboxylic acid partner and were pleased to find that a variety of different aromatic, heteroaromatic and aliphatic carboxylic acids reacted smoothly to afford the corresponding amides *anti*-1q–aa in high yields with complete diastereoselectivity. Notably, *N*-protected amino acids could also be employed as substrates, leading to dipeptide analogues *anti*-1ab–1ac in high yields and >95 : 5 d.r. To highlight the generality of the chemistry, we also investigated the late-stage functionalization of pharmaceutically active carboxylic acids and were delighted to find that atropisomeric peptide analogues *anti*-1ad–ag derived from isoxepac, bendazac, tolmetin and indomethacin were all obtained in good to excellent yields with complete selectivity in favour of the *anti*-diastereoisomer. Reactions with other commerically available isocyanides were also successful, enabling the synthesis of *N*-benzyl and *N*-cyclohexyl analogues *anti*-1ah and *anti*-1ai in yields of 74% and 71% respectively. Moreover, employing commercially available ethyl isocyanoacetate enabled efficient access to atropisomeric di- and tripeptide analogues *anti*-1aj and *anti*-1ak, again with complete stereocontrol. A reaction with commercially available (*S*)-α-methylbenzyl isocyanide afforded *anti*-1al as a separable mixture of diastereoisomers (53 : 47 d.r.), thereby providing a means to access enantiopure products (see ESI† for details). Finally, we investigated alternative aniline components bearing two *ortho*-substituents and were pleased to find that reactions with 2-iodo-4,6-dimethylaniline also proceeded with essentially complete diastereocontrol to afford *anti*-1am–ao in 70–78% yield. The relative stereochemistry of *anti*-1am was unambiguously assigned *via* single crystal X-ray diffraction and confirmed to have the bulky iodine substituent oriented *anti* to the phenyl side chain. Analogous products *anti*-1ap and *anti*-1aq substituted with bromine and chlorine respectively were obtained in good yields, but somewhat lower diastereoselectivity (90 : 10 d.r. and 77 : 23 d.r. respectively), which is likely due to the smaller degree of steric differentiation between *ortho*-substituents. A multicomponent reaction with 2-phenyl-4,6-dimethylaniline delivered *anti*-1ar in 92% yield as a single diastereoisomer, whose relative configuration was again confirmed by X-ray analysis. We were pleased to discover that substituted naphthylamines were also compatible with our new method, with peptide analogues *anti*-1as and *anti*-1at both obtained in good yields and excellent diastereoselectivity. Interestingly, in *anti*-1as (which lacks a C2-substituent) the naphthyl backbone is orientated *anti*- to the phenyl group, but in *anti*-1at the C2-methyl group preferentially adopts the equivalent position. Not all of the anilines we investigated underwent the desired multicomponent reaction. For example, reactions with extremely sterically congested amines such as 2-isopropyl-4-methylpyridin-3-amine and 2-*tert*-butyl-4,6-diphenylaniline did not afford the desired products *anti*-1au and *anti*-1av instead delivering a mixture of unreacted amine, imine and Passerini product (see ESI† for details).

**Scheme 3:**
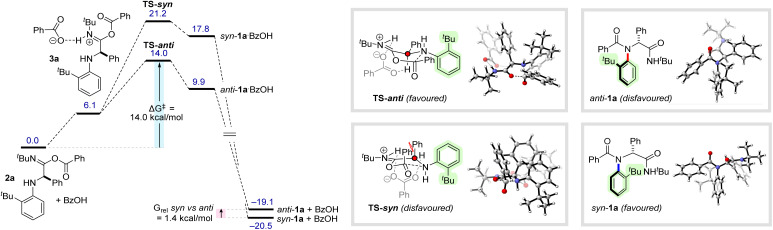
Computational modeling of the reaction pathway. Calculations were carried out at the wB97M-V/def2-QZVPP/SMD(CF_3_CH_2_OH)//PBE0-D3/def2-TZVP/SMD(CF_3_CH_2_OH) level, with free energy corrections applied for 298.15 K using a qRRHO approximation.^18^ All free energies are reported in kcal mol^−1^.

With the generality of the new methodology established, we set out to perform computational modelling with the aim of rationalizing the stereochemical outcome of the transformation (Scheme 3). Although several mechanisms have been proposed for four-component Ugi reactions, there is strong consensus that the final step involves intramolecular acyl transfer (Mumm rearrangement) of an imidate intermediate, and we rationalised that this would be the step which establishes the relative configuration of the atropisomeric products.^19^ For a representative imidate 2a, geometry minimization was performed at the wB97M-V^20^/def2-QZVPP/SMD(CF_3_CH_2_OH)//PBE0-D3 ^21,22^/def2 ^23^-TZVP^23^/SMD(CF_3_CH_2_OH)^24^ level of theory. We were unable to find any energetically feasible processes involving direct Mumm rearrangement of 2a, so inspired by recent work by Houk, Tan and co-workers, we investigated the potential for an acid mediated rearrangement.^16*a*^ Protonation of 2a by benzoic acid was calculated to be a mildly endergonic process (Δ*G* = +6.1 kcal mol^−1^) to form protonated imidate 3a – this was assumed to be very rapid. This intermediate was observed to undergo facile acyl transfer *via*TS-*anti* (Δ*G*^‡^ = 14.0 kcal mol^−1^) leading to amidinium species *anti*-1a·BzOH (9.9 kcal mol^−1^), which was assumed to undergo very rapid deprotonation to form *anti*-1a (−19.1 kcal mol^−1^). In line with our experimental observations, formation of the *syn*-configured product *via*TS-*syn* was calculated to be significantly less favourable (Δ*G*^‡^ = 21.2 kcal mol^−1^). The key difference appears to be that in the favoured *anti*-transition state, the bulky *ortho-tert* butyl group can be comfortably accommodated between the two equatorially disposed phenyl groups. Conversely, in the disfavoured *syn*-transition state, the phenyl substituents are forced closer to an axial arrangement, leading to an unfavourable 1,3-interaction. Notably, in both transition states, benzoic acid was observed to play a key role, forming a stabilizing C–H⋯O interaction with the benzylic hydrogen atom. Interestingly, these computational calculations also predicted that *anti*-1a produced as the kinetic product of the Ugi reaction is destabilized with respect to *syn*-1a by 1.4 kcal mol^−1^. This result raised the interesting possibility of performing a selective thermal atropisomerization of the kinetically formed *anti*-diastereoisomer to selectively access the thermodynamic *syn*-atropisomer.

**Scheme 4:**
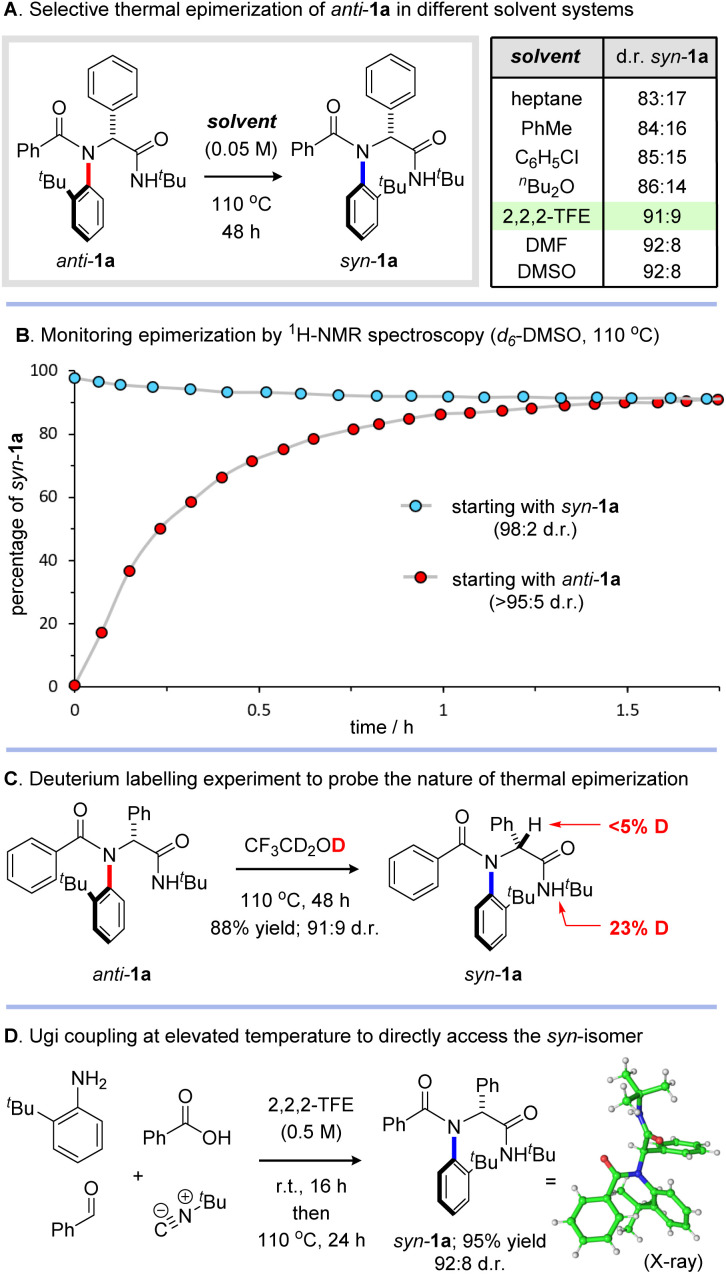
Selective atropisomerization studies. (A) *anti*-1a (0.045 mmol), solvent, 110 °C, 48 h. Diastereomeric ratios determined by ^1^H NMR analysis of the crude reaction mixture. (B) Isomerization was carried out in an NMR tube and analyzed by variable temperature ^1^H-NMR spectroscopy (500 MHz, 110 °C) starting with either *syn*-1a or *anti*-1a (∼10 mg), *d*_6_-DMSO (0.5 mL). (C) Reaction conditions: *anti*-1a (0.090 mmol), CF_3_CD_2_OD (1 mL), 110 °C, 48 h. The extent of deuterium labelling was determined by ^1^H NMR spectroscopy (see ESI† for details). (D) Reaction conditions: *ortho*-substituted aniline (1 eq.), aldehyde (1 eq.), carboxylic acid (1 eq.), isocyanide (1 eq.), 2,2,2-trifluoroethanol (0.5 M), r.t. 16 h then 110 °C, 24 h. Yield refers to isolated material after column chromatography. Diastereoselectivity determined by ^1^H NMR analysis of the crude reaction mixture.

To test this hypothesis experimentally, we investigated the thermal isomerization of *anti*-1a in heptane at 110 °C (Scheme 4A). In line with our computational predictions, we were delighted to observe an efficient switch in relative configuration to afford *syn-*1a as the major diastereoisomer (83 : 17 d.r.). Our computational calculations also suggested that the *syn*-diastereoisomer is significantly more polar than the *anti*-isomer (calculated dipole moments for *syn*-1a and *anti*-1a are 3.51 a.u. and 2.41 a.u. respectively) and accordingly, we questioned whether the thermodynamic preference for the *syn*-diastereomer could be exacerbated by a more polar solvent. To test this theory, we explored heating *anti*-1a in a variety of different solvents and were pleased to find that more polar solvents indeed proved beneficial, with toluene, chlorobenzene and dibutyl ether affording improved selectivity. The best results were obtained in polar protic or aprotic solvents, with 2,2,2-TFE, DMSO and DMF all delivering *syn*-1a with excellent diastereoselectivity (up to 92 : 8 d.r.). To probe the kinetics of this thermal atropisomerization process we studied the isomerization of both *syn*- and *anti*-1a in *d*_6_-DMSO at 110 °C (Scheme 4B).^25^^1^H NMR spectra were recorded at regular intervals, revealing smooth conversion to a thermodynamic mixture of diastereomers within 2 hours. From the data for *anti*-1a we calculated free energy barriers for atropisomerization of 28.2 kcal mol^−1^ (Δ*G*^‡^_f_) and 30.0 kcal mol^−1^ (Δ*G*^‡^_b_) at 110 °C (see ESI† for details). This corresponds to a half-life of around 1 year at room temperature, confirming these molecules to be configurationally stable atropisomers. To verify that isomerization proceeds by thermal atropisomerization and rule out a pathway involving enolization of the α-stereogenic centre, we performed an isotopic labelling experiment where *anti*-1a was heated in *d*_3_-trifluoroethanol (Scheme 4C). Under these conditions *syn*-1a was obtained in 88% yield and 91 : 9 d.r. with no deuterium incorporation at the α-stereogenic centre, strongly supporting a mechanism involving atropisomerization. These thermal isomerization experiments also led us to consider whether it might be possible to augment our *anti*-selective multicomponent reactions (Scheme 2) with four component couplings performed at elevated temperature to directly access *syn*-configured peptide analogues. To test this hypothesis, an equimolar mixture of 2-*tert*-butylaniline, benzoic acid, benzaldehyde and *tert*-butyl isocyanide in 2,2,2-TFE was stirred overnight in 2,2,2-TFE at room temperature and then heated at 110 °C for 24 h (Scheme 4D). Under these conditions, we were delighted to isolate the desired product *syn*-1a in an excellent yield of 95% with very high levels of stereocontrol (92 : 8 d.r.). Excitingly, this result indicates that simply increasing the reaction temperature results in near complete stereodivergence, allowing the alternative stereoisomer of C–N atropisomeric peptide analogues to be selectively assembled from the same set of starting materials.

**Scheme 5:**
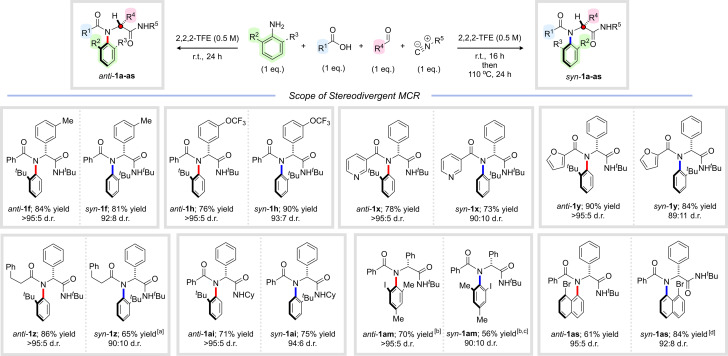
Stereodivergent multicomponent synthesis of C–N atropisomeric amides (results for *anti*-selective reactions from Scheme 2 are reproduced for clarity). Yields refer to isolated material after column chromatography. Diastereoselectivity determined by ^1^H NMR analysis of the crude reaction mixture. Reaction conditions for *anti*-selective reactions are shown in Scheme 2. Reaction conditions for *syn*-selective reactions: *ortho*-substituted aniline (1 eq.), aldehyde (1 eq.), carboxylic acid (1 eq.), isocyanide (1 eq.), 2,2,2-trifluoroethanol (0.5 M), r.t., 16 h then 110 °C, 24 h. ^*a*^Heated to 110 °C in DMSO for 6 days. ^*b*^An additional portion of isocyanide (1 eq.) was added after 24 h and the reaction was stirred for a further 24 h at r.t. ^*c*^Heated to 150 °C in DMSO for 5 hours. ^*d*^Relative stereochemistry assigned by single crystal X-ray analysis.

We next explored the scope of this *syn*-selective multicomponent reaction, initially focusing on the aldehyde component (Scheme 5 – in each case, the result of the corresponding *anti*-selective reaction from Scheme 2 is reproduced alongside for clarity). We were delighted to find that substituted aldehydes participated efficiently in the thermal four-component reaction, delivering peptide analogues *syn*-1f and *syn*-1h in excellent yields and with near complete stereocontrol. Likewise, the acid component could be successfully varied, and reactions of heterocyclic and aliphatic carboxylic acids furnished *syn*-1x–z, all in excellent yields and diastereoselectivities. Interestingly, for *syn*-1z, more forcing thermal conditions were required to achieve high levels of stereocontrol and detailed analysis revealed substantially higher free energy barriers for atropisomerization in this case of 31.5 kcal mol^−1^ (Δ*G*^‡^_f_) and 33.3 kcal mol^−1^ (Δ*G*^‡^_b_) (see ESI† for details). This is in line with our previous work on other C–N atropisomeric scaffolds where we observed that C–N atropisomeric amides bearing aliphatic acyl substituents undergo atropisomerization significantly more slowly than aromatic analogues.^26^ The isocyanide component could also readily be varied, with isomeric cyclohexyl substituted amide *syn*-1ai isolated in excellent yield and stereoselectivity (94 : 6 d.r.). Other *ortho*-substituted anilines could also be employed under thermal conditions, leading to *syn*-1am and *syn*-1as in good to excellent yields with very high levels of diastereocontrol. The structural and stereochemical assignment of *syn*-1as was unambiguously confirmed by single crystal X-ray analysis.

**Scheme 6:**
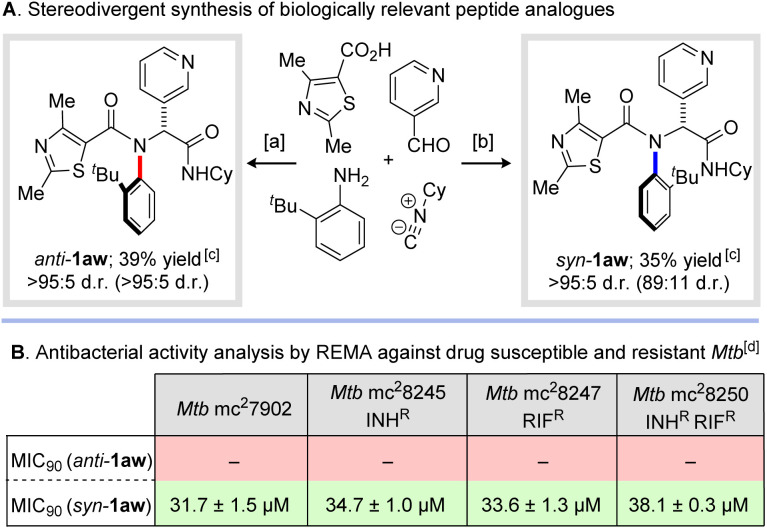
Synthesis and inhibitory activity of *N*-aryl peptide analogues against drug-susceptible and drug-resistant strains of *Mycobacterium tuberculosis*. ^*a*^2-*tert*-Butylaniline (1 eq.), nicotinaldehyde (1 eq.), carboxylic acid (1 eq.), cyclohexylisocyanide (1 eq.), 2,2,2-trifluoroethanol (0.5 M), r.t., 24 h. ^*b*^2-*tert*-Butylaniline (1 eq.), nicotinaldehyde (1 eq.), carboxylic acid (1 eq.), cyclohexylisocyanide (1 eq.), 2,2,2-trifluoroethanol (0.5 M), r.t., 16 h then 110 °C, 24 h. ^*c*^Yields and diastereomeric ratios refer to material obtained after column chromatography (d.r. values in parentheses measured from crude ^1^H NMR spectrum). Additional purification by recrystallization was carried out prior to biological assays. ^*d*^Values correspond to minimum concentrations required to achieve 90% inhibition (MIC_90_) as determined by the Resazurin Microtiter Assay (REMA) method against drug susceptible and resistant *Mtb*. Values of – correspond to no activity observed at 64 μM. See ESI† for details.

Finally, we sought to highlight the potential of our newly developed methodology to prepare medicinally relevant molecules. Inspired by recent elegant reports on the application of scaffolds derived from Ugi multicomponent reactions for the selective inhibition of *Mycobacterium tuberculosis* (*Mtb*), we applied our newly developed methodology to prepare both diastereoisomers of atropisomeric peptide analogues *syn*- and *anti*-1aw (Scheme 6A).^27^ The anti-tubercular activity of these stereoisomeric peptide analogues was assessed against a small panel of clinically relevant drug-susceptible and drug-resistant strains of *Mtb* using the Resazurin Microtiter Assay (REMA) method developed by Palomino and co-workers.^28^ (Scheme 6B). Excitingly, the *syn*-configured diastereoisomer displayed a promising level of activity (MIC_90_ = 31.7 ± 1.5 μM) against the auxotrophic wild-type strain (*Mtb* mc^2^7902).^29^ In contrast, the *anti*-configured diastereoisomer displayed no inhibition up to the maximal concentration of 64 μM employed in the assay. Similar results were observed across a small panel of clinically relevant singly and doubly drug resistant *Mtb*, with *syn*-1aw displaying antitubercular activity (MIC_90_ ≤ 38.1 μM), and the *anti*-diastereoisomer showing no inhibition (see ESI† for details). These results show significant potential for future development of new highly active and selective antimicrobial agents and underscore the powerful impact atropisomerism can play in the biological activity of small molecules.

## Conclusions

C–N atropisomeric amides are rapidly emerging as powerful scaffolds for catalysis and medicinal chemistry but methods for their stereoselective synthesis are underdeveloped, particularly in cases where the presence of additional stereogenic elements leads to the potential for multiple diastereoisomers. We have developed a new multicomponent approach for synthesis of the first examples of C–N atropisomeric peptide analogues possessing both central and axial chirality. These reactions enable simple, readily available building blocks (aldehydes, carboxylic acids, anilines and isocyanides) to be assembled under mild conditions to deliver a wide range of C–N atropisomeric peptide analogues with very high efficiency and complete diastereocontrol. The flexibility, generality and functional group tolerance of the method is particularly noteworthy – extensive variation of all four reaction components was possible including the use of complex pharmaceutically active molecules as reagents. Remarkably, we found that increasing the reaction temperature could enable stereodivergent reactions capable of selectively targeting the other diastereoisomer of C–N atropisomeric products, again with broad scope, excellent yields and near complete stereocontrol. The mechanism of these reactions was interrogated employing detailed experimental and computational studies, shedding light on the kinetic and thermodynamic aspects of stereocontrol. Finally, we employed our new methodology to prepare novel C–N atropisomeric peptide scaffolds that display inhibitory activity against mc^2^7092, a H37Rv BSL-2 arroved model organism, with a significant difference in activity observed between different atropisomers. We anticipate that this chemistry will spur the development of other new C–N atropisomeric scaffolds based upon biologically relevant peptide scaffolds, especially efforts to achieve stereocontrolled synthesis of atropisomers by exploiting thermodynamic *versus* kinetic control.

## Data availability

The data that support the findings of this study are available in the ESI† of this article, including detailed experimental procedures, characterization data for new compounds, details of computational methods, details of biological assays. Crystallographic data have been deposited with the Cambridge Crystallographic Data Centre (CCDC 2355697–2355699 and 2382977–2382979).

## Author contributions

N. J. R., A. D. G. C. and R. J. A. conceived and designed the study. N. J. R. and A. D. G. C. performed the synthetic experiments and analysed data for all compounds. P. G. W. performed single crystal X-ray analysis and analysed the associated data. A. K. B. and N. J. R. carried out biological assays. K. E. performed the computational calculations. R. J. A. and K. E. wrote the manuscript with input from all other authors.

## Conflicts of interest

There are no conflicts to declare.

## Supplementary Material

SC-OLF-D4SC04700A-s001

SC-OLF-D4SC04700A-s002

SC-OLF-D4SC04700A-s003
