# Supplementary material for: A stereodivergent multicomponent approach for the synthesis of C–N atropisomeric peptide analogues
Source: Chem Sci. 2024 Sep 16;15(40):16743–51. doi: 10.1039/d4sc04700a (PMC11418089; doi:10.1039/d4sc04700a)
Supplement: SC-015-D4SC04700A-s002 [file SC-015-D4SC04700A-s002.pdf]

This journal is © The Royal Society of Chemistry 2024

Contents

**1.**	**General Information**	**S2**
**2.**	**General Procedures**	**S3**
**3.**	**Experimental Procedures**	**S4**
3.1.	*Synthesis of anti-configured peptide analogues*	**S4**
3.2.	*Mechanistic experiments and configurational stability studies*	**S47**
3.3.	*Synthesis of syn-configured peptide analogues*	**S61**
**4.**	**X-ray Crystallography**	**S71**
**5.**	**Computational Modelling**	**S72**
5.1.	*Computational methods*	**S72**
5.2	*Summary of the associated computational dataset contents*	**S72**
**6.**	**M. tuberculosis inhibition**	**S77**
6.1	*Results*	**S77**
6.2	*Materials and methods*	**S77**
**7.**	**References**	**S81**
**8.**	**NMR spectra**	**S82**

1. General Information

Reactions were carried out under an atmosphere of nitrogen. Room temperature (RT) refers to 20-25 °C. Temperatures of 0 °C were obtained using an ice/water bath. Reactions at elevated temperature were performed using an oil bath equipped with a contact thermometer.

All solvents (including anhydrous solvents) were used as supplied without prior purification. All other reagents were used directly as supplied by major chemical suppliers, or following purification procedures described by Perrin and Armarego.[1]

Thin layer chromatography was performed on Merck Kieselgel 60 F_{254} 0.25 mm pre-coated aluminium plates. Product spots were visualized under UV light (λ = 254 nm) and/or by staining with potassium permanganate solution. Flash chromatography was performed using VWR silica gel 60 (40-63 µm particle size) using head pressure by means of a nitrogen line.

NMR spectroscopy was carried out using Bruker 300 MHz, 400 MHz, 500 MHz, Cryo 700 MHz spectrometers in the deuterated solvent stated, using the residual non-deuterated solvent signal as an internal reference. Chemical shifts are quoted in ppm with signal splitting recorded as singlet (s), doublet (d), triplet (t), quartet (q), quintet (qn), sextet (sext), septet (sept), octet (oct), nonet (non) and multiplet (m). The abbreviation br denotes broad. Coupling constants, *J*, are measured to the nearest 0.1 Hz and are presented as observed.

Infrared spectra were recorded on a PerkinElmer UATR Two spectrometer with attenuated total reflectance. Absorption maxima (λ_{max}) are reported in wavenumbers (cm^{-1}).

HRMS was recorded on a Waters Xevo G2-XS Quadrupole Time-of-Flight (QToF) spectrometer equipped with a Waters Acquity UPLC i-Class LC system, under conditions of electrospray ionisation (ESI). The mass reported is that containing the most abundant isotopes, with each value rounded to 4 decimal places and within 10 ppm of the calculated mass.

Normal phase chiral HPLC was performed on a Dionex Ultimate 3000 HPLC unit equipped with UV-vis diode-array detector, fitted with the appropriate DAICEL column (dimensions: 0.46 cm ø x 25 cm) along with the corresponding guard column (0.4 cm ø x 1 cm). Wavelengths (λ) are reported in nm, retention times (t_R) are reported in minutes and solvent flow rates are reported in mL min^{-1}.

2. General Procedures

General Procedure A: *Four-component coupling reactions at room temperature*

A round bottomed flask equipped with a stirrer bar, was sequentially charged with the appropriate aniline (1.0 equiv.), the appropriate carboxylic acid (1.0 equiv.), the appropriate aldehyde (1.0 equiv.), 2,2,2-trifluoroethanol (2 mL/mmol aniline) and the appropriate isocyanide (1.0 equiv.). The resulting mixture was stirred at room temperature for 24 hours. The reaction mixture was concentrated to dryness, followed by addition of addition of EtOAc and saturated aqueous Na_2CO_3. The layers were separated, and the aqueous layer was extracted twice with EtOAc. The combined organic extracts were dried over $MgSO_4$, filtered and the resulting solution was concentrated and purified by column chromatography (see experimental methods section for details).

General Procedure B: *Four-component coupling reactions at elevated temperature*

An Ace pressure tube (capacity ~15 mL, L × O.D. 10.2 cm × 25.4 mm) equipped with a stirrer bar, was sequentially charged with the appropriate aniline (1.0 equiv.), the appropriate carboxylic acid (1.0 equiv.), the appropriate aldehyde (1.0 equiv.), 2,2,2-trifluoroethanol (2 mL/mmol aniline) and the appropriate isocyanide (1.0 equiv.). The tube was sealed, and the resulting mixture was stirred at room temperature for 16 hours and then heated to 110 ^{o}C in a preheated oil bath for a further 24 h. The resulting solution was cooled to room temperature and concentrated to dryness, followed by addition of EtOAc and saturated aqueous Na_2CO_3. The layers were separated, and the aqueous layer was extracted twice with EtOAc. The combined organic extracts were dried over $MgSO_4$, filtered and the resulting solution was concentrated and purified by column chromatography (see experimental methods section for details).

3. Experimental Procedures

3.1. Synthesis of anti-configured peptide analogues

***rac*-(R_a,*R*)-N-(2-(*tert*-Butyl)phenyl)-N-(2-(*tert*-butylamino)-2-oxo-1-phenylethyl)benzamide, *anti*-1a**

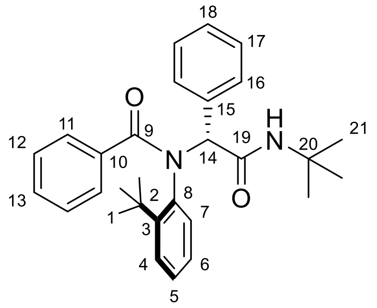

<u>1.20 mmol scale reaction:</u> 2-*tert*-Butylaniline (180 mg, 1.20 mmol, 1.0 eq.), benzoic acid (147 mg, 1.20 mmol, 1.0 eq.), benzaldehyde (122 µL, 1.20 mmol, 1.0 eq.), 2,2,2-trifluoroethanol (2.4 ml, 0.5 M) and *tert*-butyl isocyanide (136 µL, 1.20 mmol, 1.0 eq.) were subjected to **General Procedure A**. [N.B. in this case, the crude residue obtained upon removal of 2,2,2-trifluoroethanol was directly purified via column chromatography without an aqueous workup]. Purification *via* column chromatography eluting with petrol 40-60/EtOAc 90:10 afforded the title compound *anti*-**1a** as a pale-yellow solid (491 mg, 92% yield, >95:5 d.r.).

<u>4.00 mmol scale reaction:</u> 2-*tert*-Butylaniline (597 mg, 4.00 mmol, 1.0 eq.), benzoic acid (489 mg, 4.00 mmol, 1.0 eq.), benzaldehyde (0.41 mL, 4.00 mmol, 1.0 eq.), 2,2,2-trifluoroethanol (8.0 ml, 0.5 M) and *tert*-butyl isocyanide (0.45 mL, 4.00 mmol, 1.0 eq.) were subjected to **General Procedure A**. [N.B. in this case, the crude residue obtained upon removal of 2,2,2-trifluoroethanol was directly purified *via* column chromatography without an aqueous workup]. Purification *via* column chromatography eluting with petrol 40-60/Et_2O 90:10 afforded the title compound *anti*-**1a** as a pale-yellow solid (1.52 g, 86% yield).

^{1}H NMR (300 MHz, $CDCl_3$): δ_H 7.93 – 7.88 (m, 1H, H_7), 7.78 – 7.65 (m, 2H, H_{16}), 7.46 – 7.36 (m, 3H, H_{17} and H_{18}), 7.33 – 7.14 (m, 6H, H_4, H_5, H_6, H_{11} and H_{13}), 7.13 – 7.04 (m, 2H, H_{12}), 5.69 (s, 1H, NH), 5.43 (s, 1H, H_{14}), 1.30 (s, 9H, H_{21}), 0.83 (s, 9H, H_1).

^{13}C NMR (75 MHz, $CDCl_3$): δ_C 170.5, 167.9, 146.8, 141.3, 136.7, 136.5, 132.3, 130.8, 130.7, 129.8, 129.6, 129.1, 128.9, 128.0, 127.4, 126.8, 74.3, 51.7, 36.2, 32.2, 28.7.

FTIR (cm^{-1}): 3305, 3071, 2958, 1686, 1615, 1551, 1361, 760, 699, 641.

Melting Point (°C): 170 – 172.

HRMS (ESI): Exact mass calculated for $C_{29}H_{35}N_2O_2^+$ $[M+H]^+$: 443.2693, found: 443.2700. Δ = 1.6 ppm.

***rac*-(R_a,*R*)-N-(2-(*tert*-butyl)phenyl)-N-(2-(*tert*-butylamino)-2-oxo-1-(*p*-tolyl)ethyl)benzamide, *anti*-1b**

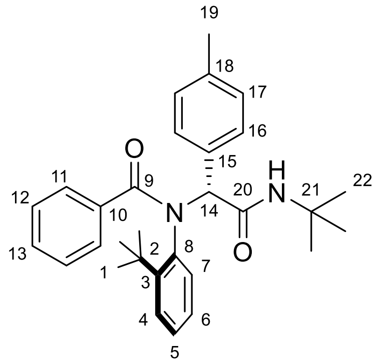

2-*tert*-Butylaniline (180 mg, 1.20 mmol, 1.0 eq.), benzoic acid (147 mg, 1.20 mmol, 1.0 eq.), and 4-methylbenzaldehyde (141 µL, 1.20 mmol, 1.0 eq.), 2,2,2-trifluoroethanol (2.4 ml, 0.5 M) and *tert*-butyl isocyanide (136 µL, 1.20 mmol, 1.0 eq.) were subjected to **General Procedure A**. Purification *via* column chromatography eluting with petrol 40-60/EtOAc 90:10 afforded the title compound *anti*-**1b** as a pale yellow solid (449 mg, 82% yield, >95:5 d.r.).

^{1}H NMR (300 MHz, CDCl$_3$): δ_H δ 7.97 – 7.87 (m, 1H, H_7), 7.59 (d, *J* = 8.1 Hz, 2H, H_{16}), 7.31 – 7.14 (m, 8H, H_4, H_5, H_6, H_{13}, H_{11} and H_{17}), 7.08 (dd, *J* = 8.2, 6.6 Hz, 2H, H_{12}), 5.71 (s, 1H, NH), 5.38 (s, 1H, H_{14}), 2.39 (s, 3H, H_{19}), 1.30 (s, 9H, H_{22}), 0.82 (s, 9H, H_1).

^{13}C NMR (75 MHz, CDCl$_3$): δ_C 170.4, 168.2, 146.8, 141.4, 138.6, 136.6, 133.6, 132.3, 130.8, 130.7, 129.8, 129.6, 127.9, 127.3, 126.8, 74.1, 51.7, 36.2, 32.2, 28.7, 21.4. N.B. the peak at 129.8 ppm corresponds to two overlapping signals.

FTIR (cm^{-1}): 3316, 2970, 2922, 1688, 1613, 1548, 1356, 713, 627.

Melting Point (°C): 155 – 156.

HRMS (ESI): Exact mass calculated for $C_{30}H_{37}N_2O_2^+$ [M+H]$^+$: 457.2850, found: 457.2851. Δ = 0.2 ppm.

***rac*-(R_a,*R*)-N-(2-(*tert*-butyl)phenyl)-N-(2-(*tert*-butylamino)-1-(4-methoxyphenyl)-2-oxoethyl)benzamide, *anti*-1c**

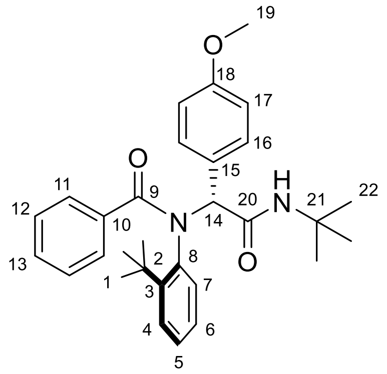

2-*tert*-Butylaniline (180 mg, 1.20 mmol, 1.0 eq.), benzoic acid (147 mg, 1.20 mmol, 1.0 eq.), 4-methoxybenzaldehyde (146 µL, 1.20 mmol, 1.0 eq.), 2,2,2-trifluoroethanol (2.4 ml, 0.5 M) and *tert*-butyl isocyanide (136 µL, 1.20 mmol, 1.0 eq.) were subjected to **General Procedure A**. Purification *via* column chromatography eluting with petrol 40-60/Et_2O 70:30 afforded the title compound *anti*-**1c** as a white solid (469 mg, 83% yield, >95:5 d.r.).

^{1}H NMR (300 MHz, $CDCl_3$): δ_H 7.95 (d, *J* = 7.9 Hz, 1H, H_7), 7.65 (d, *J* = 8.7 Hz, 2H, H_{16}), 7.31 – 7.13 (m, 6H, H_4, H_5, H_6, H_{11} and H_{13}), 7.07 (dd, *J* = 8.1, 6.5 Hz, 2H, H_{12}), 6.92 (d, *J* = 8.7 Hz, 2H, H_{17}), 5.69 (s, 1H, NH), 5.31 (s, 1H, H_{14}), 3.85 (s, 3H, H_{19}), 1.31 (s, 9H, H_{22}), 0.80 (s, 9H, H_1).

^{13}C NMR (75 MHz, $CDCl_3$): δ_C 170.4, 168.3, 159.9, 146.7, 141.5, 136.6, 132.5, 132.3, 130.6, 129.8, 129.5, 128.7, 127.8, 127.3, 126.9, 114.3, 74.0, 55.4, 51.7, 36.2, 32.2, 28.8.

FTIR (cm^{-1}): 3313, 2964, 2909, 1688, 1613, 1549, 1509, 1358, 1251, 758, 715, 628.

Melting Point (°C): 154 – 155.

HRMS (ESI): Exact mass calculated for $C_{30}H_{37}N_2O_3^+$ $[M+H]^+$: 473.2799, found: 473.2818. Δ = 4.0 ppm.

***rac*-(R_a,*R*)-N-(2-(*tert*-butyl)phenyl)-N-(2-(*tert*-butylamino)-1-(4-hydroxyphenyl)-2-oxoethyl)benzamide, *anti*-1d**

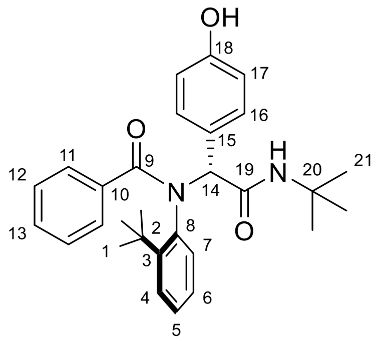

2-*tert*-Butylaniline (180 mg, 1.20 mmol, 1.0 eq.), benzoic acid (147 mg, 1.20 mmol, 1.0 eq.), 4-hydroxybenzaldehyde (147 mg, 1.20 mmol, 1.0 eq.), 2,2,2-trifluoroethanol (2.4 ml, 0.5 M) and *tert*-butyl isocyanide (136 µL, 1.20 mmol, 1.0 eq.) were subjected to **General Procedure A**. [N.B. in this case, the crude residue obtained upon removal of 2,2,2-trifluoroethanol was directly purified via column chromatography without an aqueous workup]. Purification *via* column chromatography eluting with petrol 40-60/EtOAc 80:20 afforded the title compound *anti*-**1d** as a white solid (416 mg, 76% yield, >95:5 d.r.).

^{1}H NMR (300 MHz, $CDCl_3$): δ_H 7.93 (dd, *J* = 7.6, 1.9 Hz, 1H, H_7), 7.84 – 7.54 (br s, 1H, OH), 7.48 (d, *J* = 8.6 Hz, 2H, H_{16}), 7.32 – 7.13 (m, 6H, H_4, H_5, H_6, H_{11} and H_{13}), 7.08 (dd, *J* = 8.2, 6.6 Hz, 2H, H_{12}), 6.80 (d, *J* = 8.6 Hz, 2H, H_{17}), 5.71 (s, 1H, NH), 5.33 (s, 1H, H_{14}), 1.26 (s, 9H, H_{21}), 0.83 (s, 9H, H_1).

^{13}C NMR (75 MHz, $CDCl_3$): δ_C 170.9, 168.3, 157.2, 146.9, 141.0, 136.2, 132.1, 132.1, 130.9, 129.8, 129.8, 128.0, 127.5, 126.8, 116.4, 74.4, 51.8, 36.3, 32.2, 28.7, N.B. The peak at 127.5 ppm corresponds to two overlapping signals.

FTIR (cm^{-1}): 3418, 3229, 2972, 1667, 1615, 1511, 717, 500.

Melting Point (°C): 172 – 173.

HRMS (ESI): Exact mass calculated for $C_{29}H_{35}N_2O_3^+$ [M+H]$^+$: 459.2642, found: 459.2625. Δ = –3.7 ppm.

***rac*-(R_a,*R*)-N-(2-(*tert*-butyl)phenyl)-N-(2-(*tert*-butylamino)-1-(4-fluorophenyl)-2-oxoethyl)benzamide, *anti*-1e**

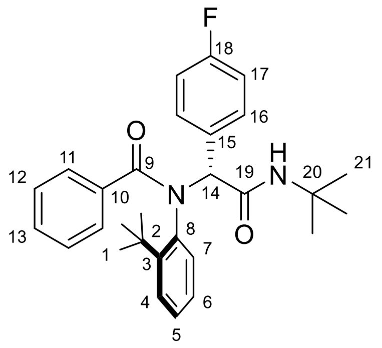

2-*tert*-Butylaniline (180 mg, 1.20 mmol, 1.0 eq.), benzoic acid (147 mg, 1.20 mmol, 1.0 eq.), 4-fluorobenzaldehyde (129 µL, 1.20 mmol, 1.0 eq.), 2,2,2-trifluoroethanol (2.4 ml, 0.5 M) and *tert*-butyl isocyanide (136 µL, 1.20 mmol, 1.0 eq.) were subjected to **General Procedure A**. [N.B. in this case, the crude residue obtained upon removal of 2,2,2-trifluoroethanol was directly purified via column chromatography without an aqueous workup]. Purification *via* column chromatography eluting with petrol 40-60/EtOAc 90:10 afforded the title compound *anti*-**1e** as a white solid (455 mg, 82% yield, >95:5 d.r.).

^{1}H NMR (300 MHz, CDCl$_3$): δ_H 7.86 (dd, *J* = 7.2, 2.2 Hz, 1H, H_7), 7.76 – 7.67 (m, 2H, H_{16}), 7.31 – 7.15 (m, 6H, H_4, H_5, H_6, H_{13} and H_{11}), 7.14 – 7.05 (m, 4H, H_{12} and H_{17}), 5.67 (s, 1H, NH), 5.41 (s, 1H, H_{14}), 1.31 (s, 9H, H_{21}), 0.83 (s, 9H, H_1).

^{13}C NMR (75 MHz, CDCl$_3$): δ_C 170.7, 167.7, 163.0 (d, *J* = 248.5 Hz) 146.8, 141.2, 136.4, 132.9 (d, *J* = 8.0 Hz), 132.6 (d, *J* = 3.4 Hz), 132.3, 130.8, 129.8, 129.7, 128.1, 127.4, 126.9, 116.0 (d, *J* = 21.3 Hz), 73.5, 51.8, 36.2, 32.2, 28.8.

^{19}F NMR (282 MHz, CDCl$_3$): δ_F –112.5 – –112.7 (m).

FTIR (cm^{-1}): 3312, 2972, 1687, 1635, 1614, 1549, 1359, 1221, 762, 710, 694, 626.

Melting Point (°C): 161 – 162.

HRMS (ESI): Exact mass calculated for $C_{29}H_{34}FN_2O_2^+$ [M+H]$^+$: 461.2599, found: 461.2619. Δ = 4.3 ppm.

***rac*-(*R*$_a$,*R*)-N-(2-(*tert*-butyl)phenyl)-N-(2-(*tert*-butylamino)-2-oxo-1-(*m*-tolyl)ethyl)benzamide, *anti*-1f**

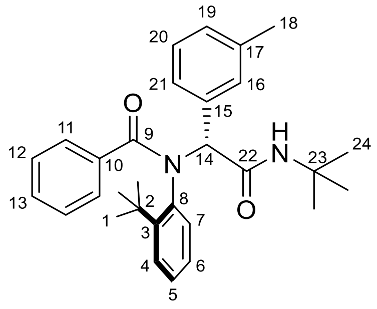

2-*tert*-Butylaniline (180 mg, 1.20 mmol, 1.0 eq.), benzoic acid (147 mg, 1.20 mmol, 1.0 eq.), 3-methyl benzaldehyde (141 µL, 1.20 mmol, 1.0 eq.), 2,2,2-trifluoroethanol (2.4 ml, 0.5 M) and *tert*-butyl isocyanide (136 µL, 1.20 mmol, 1.0 eq.) were subjected to **General Procedure A**. Purification *via* column chromatography eluting with petrol 40-60/Et_2O 85:15 afforded the title compound *anti*-**1f** as a white solid (459 mg, 84% yield, >95:5 d.r.).

^{1}H NMR (300 MHz, $CDCl_3$): δ_H 7.91 – 7.84 (m, 1H, H_7), 7.58 (dt, *J* = 7.7, 1.5 Hz, 1H, H_{21}), 7.41 (s, 1H, H_{16}), 7.35 – 7.13 (m, 8H, H_4, H_5, H_6, H_{11}, H_{13}, H_{19} and H_{20}), 7.12 – 7.04 (m, 2H, H_{12}), 5.73 (s, 1H, NH), 5.40 (s, 1H, H_{14}), 2.35 (s, 3H, H_{18}), 1.29 (s, 9H, H_{24}), 0.82 (s, 9H, H_1).

^{13}C NMR (75 MHz, $CDCl_3$): δ_C 170.4, 168.0, 146.8, 141.3, 138.8, 136.6, 132.3, 131.5, 130.7, 129.8, 129.6, 129.6, 129.0, 127.9, 127.8, 127.3, 126.8, 74.1, 51.7, 36.3, 32.2, 28.7, 21.6. N.B. The peak at 136.6 ppm corresponds to two overlapping signals.

FTIR (cm^{-1}): 3308, 2962, 1690, 1615, 1551, 1359, 761, 700, 648.

Melting Point (°C): 153 – 154.

HRMS (ESI): Exact mass calculated for $C_{30}H_{37}N_2O_2^+$ [M+H]$^+$: 457.2850, found: 457.2850. Δ = 0.0 ppm.

***rac*-(R_a,*R*)-N-(2-(*tert*-butyl)phenyl)-N-(2-(*tert*-butylamino)-2-oxo-1-(3-(trifluoromethyl)phenyl)ethyl)benzamide, *anti*-1g**

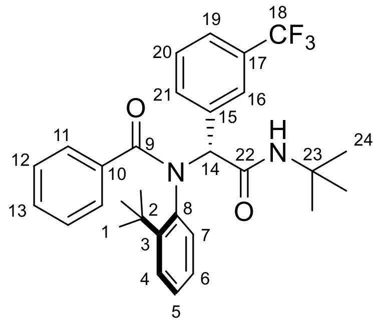

2-*tert*-Butylaniline (180 mg, 1.20 mmol, 1.0 eq.), benzoic acid (147 mg, 1.20 mmol, 1.0 eq.), 3-trifluoromethyl benzaldehyde (161 µL, 1.20 mmol, 1.0 eq.), 2,2,2,-trifluoroethanol (2.4 ml, 0.5 M) and *tert*-butyl isocyanide (136 µL, 1.20 mmol, 1.0 eq.) were subjected to **General Procedure A**. Purification *via* column chromatography eluting with petrol 40-60/Et_2O 90:10 afforded the title compound *anti*-**1g** as a white solid (455 mg, 74% yield, >95:5 d.r.).

^{1}H NMR (300 MHz, $CDCl_3$): δ_H 8.02 (d, *J* = 7.6 Hz, 1H, H_{21}), 7.85 (s, 1H, H_{16}), 7.80 – 7.60 (m, 2H, H_7 and H_{19}), 7.55 (t, *J* = 7.8 Hz, 1H, H_{20}), 7.33 – 7.15 (m, 6H, H_4, H_5, H_6, H_{11} and H_{13}), 7.13 – 7.05 (m, 2H, H_{12}), 5.81 (s, 1H, NH), 5.57 (s, 1H, H_{14}), 1.31 (s, 9H, H_{24}), 0.85 (s, 9H, H_1).

^{13}C NMR (101 MHz, $CDCl_3$): δ_C 171.0, 167.2, 146.8, 140.6, 137.7, 136.1, 134.0, 132.3, 131.3 (q, *J* = 32.4 Hz), 130.9, 129.9, 129.8, 129.5, 128.3, 127.7 (q, *J* = 3.9 Hz) 127.5, 126.9, 125.6 (q, J = 3.9 Hz), 124.0 (q, *J* = 272.4 Hz), 73.1, 52.0, 36.3, 32.2, 28.7.

^{19}F NMR (282 MHz, $CDCl_3$): δ_F –62.7 (s).

FTIR (cm^{-1}): 3293, 2971, 1698, 1685, 1631, 1328, 1130, 1119, 714, 707.

Melting Point (°C): 143 – 144.

HRMS (ESI): Exact mass calculated for $C_{30}H_{34}F_3N_2O_2^+$ [M+H]$^+$: 511.2567, found: 511.2567. Δ = 0.0 ppm.

***rac*-(R_a,*R*)-N-(2-(*tert*-butyl)phenyl)-N-(2-(*tert*-butylamino)-2-oxo-1-(3-(trifluoromethoxy)phenyl)ethyl)benzamide, *anti*-1h**

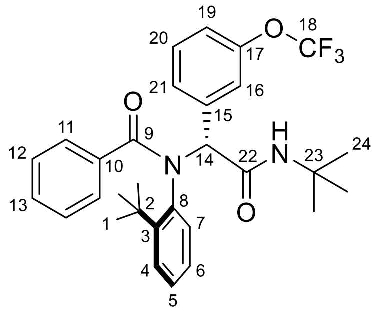

2-*tert*-Butylaniline (180 mg, 1.20 mmol, 1.0 eq.), benzoic acid (147 mg, 1.20 mmol, 1.0 eq.), 3-(trifluoromethoxy)benzaldehyde (172 µL, 1.20 mmol, 1.0 eq.), 2,2,2-trifluoroethanol (2.4 ml, 0.5 M) and *tert*-butyl isocyanide (136 µL, 1.20 mmol, 1.0 eq.) were subjected to **General Procedure A**. Purification *via* column chromatography eluting with petrol 40-60/Et_2O 80:20 afforded the title compound *anti*-**1h** as a pale-yellow solid (482 mg, 76% yield, >95:5 d.r.).

^{1}H NMR (300 MHz, $CDCl_3$): δ_H 7.83 – 7.77 (m, 1H, H_7), 7.74 (s, 1H, H_{16}), 7.59 (d, *J* = 7.8 Hz, 1H, H_{21}), 7.43 (t, *J* = 8.0 Hz, 1H, H_{20}), 7.32 – 7.15 (m, 7H, H_4, H_5, H_6, H_{11}, H_{13} and H_{19}), 7.09 (t, *J* = 7.5 Hz, 2H, H_{12}), 5.70 (s, 1H, NH), 5.46 (s, 1H, H_{14}), 1.30 (s, 9H, H_{24}), 0.84 (s, 9H, H_1).

^{13}C NMR (75 MHz, $CDCl_3$): δ_C 170.8, 167.2, 149.7 (q, *J* = 1.7 Hz), 146.8, 140.9, 139.0, 136.2, 132.2, 130.8, 130.4, 129.9, 129.8, 129.3, 128.2, 127.5, 126.9, 123.7, 121.6, 120.6 (q, *J* = 258.1 Hz), 73.4, 52.0, 36.3, 32.2, 28.7.

^{19}F NMR (282 MHz, $CDCl_3$): δ_F –57.8 (s).

FTIR (cm^{-1}): 3308, 3068, 2971, 1692, 1613, 1360, 1257, 1212, 1166, 701.

Melting Point (°C): 148 – 150.

HRMS (ESI): Exact mass calculated for $C_{30}H_{34}F_3N_2O_3^+$ $[M+H]^+$: 527.2516, found: 527.2531. Δ = 2.8 ppm.

***rac*-(R_a,*R*)-N-(2-(*tert*-butyl)phenyl)-N-(2-(*tert*-butylamino)-2-oxo-1-(*o*-tolyl)ethyl)benzamide, *anti*-1i**

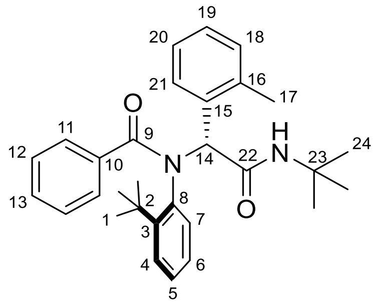

2-*tert*-Butylaniline (180 mg, 1.20 mmol, 1.0 eq.), benzoic acid (147 mg, 1.20 mmol, 1.0 eq.), 2-methyl benzaldehyde (139 µL, 1.20 mmol, 1.0 eq.), 2,2,2-trifluoroethanol (2.4 ml, 0.5 M) and *tert*-butyl isocyanide (136 µL, 1.20 mmol, 1.0 eq.) were subjected to **General Procedure A**. Purification *via* column chromatography eluting with petrol 40-60/Et_2O 80:20 afforded the title compound *anti*-**1i** as a white solid (467 mg, 85% yield, >95:5 d.r.).

^{1}H NMR (300 MHz, $CDCl_3$): δ_H 8.12 (d, *J* = 7.6 Hz, 1H, H_{21}), 7.93 (d, J = 7.1 Hz, 1H, H_7), 7.39 – 7.15 (m, 9H, H_4, H_5, H_6, H_{11}, H_{13}, H_{18}, H_{19}, H_{20}), 7.14 – 7.07 (m, 2H, H_{12}), 5.80 (s, 1H, H_{14}), 5.45 (s, 1H, NH), 2.20 (s, 3H, H_{17}), 1.15 (s, 9H, H_{24}), 0.92 (s, 9H, H_1).

^{13}C NMR (75 MHz, $CDCl_3$): δ_C 170.1, 167.4, 147.7, 140.6 (br), 137.4, 136.4, 135.3, 132.2, 131.2, 131.0, 129.9, 129.7, 129.0 (br), 128.5, 128.0, 127.4, 127.0, 126.4, 70.0, 51.5, 36.4, 32.0, 28.4, 20.0.

FTIR (cm^{-1}): 3306, 2981, 3966, 2981, 2907, 1682, 1615, 1548, 1356, 762, 717.

Melting Point (°C): 195 – 196.

HRMS (ESI): Exact mass calculated for $C_{30}H_{37}N_2O_2^+$ $[M+H]^+$: 457.2850, found: 457.2850. Δ = 0.0 ppm.

***rac*-(R_a,*R*)-N-(2-(*tert*-butyl)phenyl)-N-(2-(*tert*-butylamino)-1-(naphthalen-1-yl)-2-oxoethyl)benzamide, *anti*-1j**

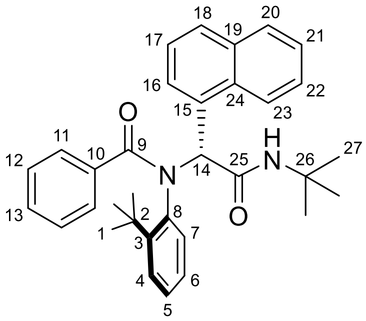

2-*tert*-Butylaniline (180 mg, 1.20 mmol, 1.0 eq.), benzoic acid (147 mg, 1.20 mmol, 1.0 eq.), 1-naphthaldehyde (163 µL, 1.20 mmol, 1.0 eq.), 2,2,2-trifluoroethanol (2.4 ml, 0.5 M) and *tert*-butyl isocyanide (136 µL, 1.20 mmol, 1.0 eq.) were subjected to **General Procedure A**. Purification *via* column chromatography eluting with petrol 40-60/Et_2O 80:20 afforded the title compound *anti*-**1j** as a white solid (509 mg, 86% yield, >95:5 d.r.).

^{1}H NMR (300 MHz, $CDCl_3$): δ_H 8.25 (d, *J* = 7.3 Hz, 1H, H_{Ar}), 7.96 – 7.85 (m, 3H, H_{Ar}), 7.72 (br s, 1H, H_{Ar}), 7.62 – 7.42 (m, 3H, H_{Ar}), 7.40 – 7.32 (m, 2H, H_{11}), 7.31 – 7.17 (m, 4H, H_{Ar}), 7.11 (m, 2H, H_{12}), 6.61 (s, 1H, H_{14}), 5.45 (s, 1H, NH), 1.04 (s, 9H, H_{27}), 0.91 (s, 9H, H_1).

^{13}C NMR (75 MHz, $CDCl_3$): δ_C 170.1, 167.3, 148.2, 139.4 (br), 136.3, 134.1, 132.3, 132.2, 132.1, 131.4, 130.1, 129.9, 129.4, 129.2, 128.1, 127.4, 126.9, 126.1, 125.9, 125.6, 123.3, 68.1, 51.5, 36.5, 31.9, 28.2. N.B. The peak at 126.9 ppm corresponds to two overlapping signals.

FTIR (cm^{-1}): 3313, 3066, 2962, 1685, 1616, 1542, 1345, 795, 763.

Melting Point (°C): 179 – 181.

HRMS (ESI): Exact mass calculated for $C_{33}H_{37}N_2O_2^+$ [M+H]$^+$: 493.2850, found: 493.2865. Δ = 3.0 ppm.

***rac*-(*R*$_a$,*R*)-N-(2-(*tert*-butyl)phenyl)-N-(2-(*tert*-butylamino)-1-(furan-2-yl)-2-oxoethyl)benzamide, *anti*-1k**

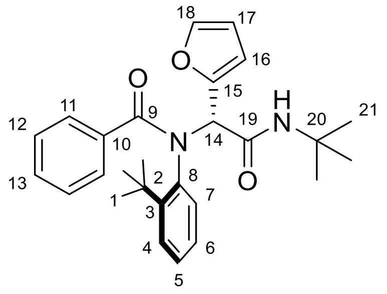

2-*tert*-Butylaniline (180 mg, 1.20 mmol, 1.0 eq.), benzoic acid (147 mg, 1.20 mmol, 1.0 eq.), furfural (99 µL, 1.20 mmol, 1.0 eq.), 2,2,2-trifluoroethanol (2.4 ml, 0.5 M) and *tert*-butyl isocyanide (136 µL, 1.20 mmol, 1.0 eq.) were subjected to **General Procedure A**. Purification *via* column chromatography eluting with petrol 40-60/Et_2O 80:20 afforded the title compound *anti*-**1k** as a yellow solid (356 mg, 69% yield, >95:5 d.r.).

^{1}H NMR (300 MHz, $CDCl_3$): δ_H 8.09 – 8.04 (m, 1H, H_7), 7.44 (dd, *J* = 1.9, 0.8 Hz, 1H, H_{18}), 7.34 – 7.14 (m, 6H, H_4, H_5, H_6, H_{11} and H_{13}), 7.13 – 7.00 (m, 2H, H_{12}), 6.86 (dd, *J* = 3.4, 0.8 Hz, 1H, H_{16}), 6.47 (dd, *J* = 3.3, 1.8 Hz, 1H, H_{17}), 6.05 (s, 1H, NH), 5.36 (s, 1H, H_{14}), 1.34 (s, 9H, H_{21}), 0.86 (s, 9H, H_1).

^{13}C NMR (75 MHz, $CDCl_3$): δ_C 169.8, 166.6, 150.1, 146.7, 142.3, 141.1, 136.0, 132.2, 130.6, 129.8, 128.2, 127.4, 127.1, 112.6, 111.7, 67.4, 51.8, 36.2, 31.9, 28.7. N.B. The peak at 129.8 ppm corresponds to two overlapping signals.

FTIR (cm^{-1}): 3300, 2958, 1688, 1624, 1551, 1349, 748, 717, 601.

Melting Point (°C): 139 – 140.

HRMS (ESI): Exact mass calculated for $C_{27}H_{33}N_2O_3^+$ [M+H]$^+$: 433.2486, found: 433.2498. Δ = 2.8 ppm.

***rac*-(R_a,*R*)-N-(2-(*tert*-butyl)phenyl)-N-(2-(*tert*-butylamino)-1-(ferrocoene)-2-oxoethyl)benzamide, *anti*-1l**

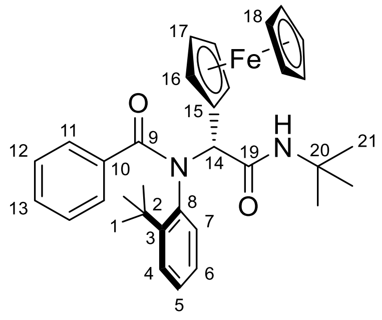

2-*tert*-Butylaniline (180 mg, 1.20 mmol, 1.0 eq.), benzoic acid (147 mg, 1.20 mmol, 1.0 eq.), ferrocenecarboxaldehyde (257 mg, 1.20 mmol, 1.0 eq.), 2,2,2-trifluoroethanol (2.4 ml, 0.5 M) and *tert*-butyl isocyanide (136 µL, 1.20 mmol, 1.0 eq.) were subjected to **General Procedure A**. Purification *via* column chromatography eluting with petrol 40-60/Et_2O 85:15 afforded the title compound *anti*-**1l** as an orange solid (503 mg, 76% yield, >95:5 d.r.).

^{1}H NMR (300 MHz, $CDCl_3$): δ_H 7.28 – 7.22 (m, 1H, H_4), 7.19 – 7.10 (m, 4H, H_{11} and H_{Ar}), 7.09 – 7.01 (m, 2H, H_{12}), 7.00 – 6.89 (m, 2H, H_{Ar}), 6.87 (s, 1H, NH), 5.99 (s, 1H, H_{14}), 4.50 – 4.47 (m, 1H, H_{Cp}), 4.36 – 4.32 (m, 1H, H_{Cp}), 4.27 – 4.17 (m, 7H, H_{Cp}), 1.47 (s, 9H, H_{21}), 1.02 (s, 9H, H_1).

^{13}C NMR (75 MHz, $CDCl_3$): δ_C 170.4, 167.2, 147.0, 138.0, 136.6, 132.5, 130.9, 129.7, 129.6, 128.0, 127.4, 125.4, 83.7, 70.3, 70.2, 69.2, 68.6, 68.5, 64.6, 51.7, 36.6, 32.5, 29.1.

FTIR (cm^{-1}): 3309, 2982, 1683, 1613, 1598, 1373, 1344, 762, 699.

Melting Point (°C): 146 – 148.

HRMS (ESI): Exact mass calculated for $[C_{33}H_{38}FeN_2O_2]^+$ $[M]^+$: 550.2277, found: 550.2284. Δ = 1.3 ppm.

***rac*-(R_a,*R*)-N-(2-(*tert*-butyl)phenyl)-N-(1-(*tert*-butylamino)-1-oxo-4-phenylbut-3-en-2-yl)benzamide, *anti*-1m**

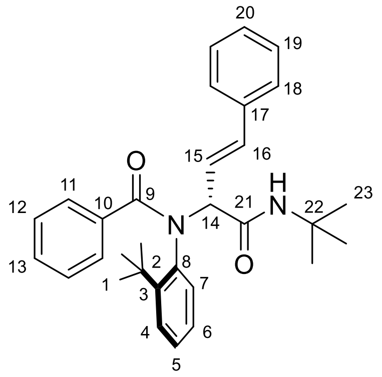

2-*tert*-Butylaniline (180 mg, 1.20 mmol, 1.0 eq.), benzoic acid (147 mg, 1.20 mmol, 1.0 eq.), *trans*-cinnamaldehyde (151 µL, 1.20 mmol, 1.0 eq.), 2,2,2-trifluroethanol (2.4 ml, 0.5 M) and *tert*-butyl isocyanide (136 µL, 1.20 mmol, 1.0 eq.) were subjected to **General Procedure A**. Purification *via* column chromatography eluting with petrol 40-60/Et_2O 80:20 afforded the title compound *anti*-**1m** as a yellow solid (359 mg, 64% yield, >95:5 d.r.).

^{1}H NMR (300 MHz, $CDCl_3$): δ_H 8.06 (dd, *J* = 7.6, 1.8 Hz, 1H, H_7), 7.46 (d, *J* = 7.0 Hz, 2H, H_{18}), 7.43 – 7.05 (m, 12H, H_4, H_5, H_6, H_{11}, H_{12}, H_{13}, H_{15} and H_{19} and H_{20}), 6.56 (d, *J* = 16.2 Hz, 1H, H_{16}), 6.19 (s, 1H, NH), 4.63 (d, *J* = 9.0 Hz, 1H, H_{14}), 1.39 (s, 9H, H_{23}), 1.06 (s, 9H, H_1).

^{13}C NMR (75 MHz, $CDCl_3$): δ_C 170.3, 168.4, 146.3, 141.7, 136.7, 136.2, 136.1, 132.2, 130.6, 129.8, 129.8, 128.9, 128.5, 128.1, 127.5, 127.3, 126.9, 125.0, 74.3, 51.8, 36.5, 32.6, 28.9.

FTIR (cm^{-1}): 3401, 2971, 1681, 1633, 1501, 1395, 757, 714, 690.

Melting Point (°C): 149 – 150.

HRMS (ESI): Exact mass calculated for $C_{31}H_{37}N_2O_2^+$ [M+H]$^+$: 469.2850, found: 469.2870. Δ = 4.3 ppm.

***rac*-(R_a,*R*)-N-(2-(*tert*-butyl)phenyl)-N-(2-(*tert*-butylamino)-1-cyclohexyl-2-oxoethyl)benzamide, *anti*-1n**

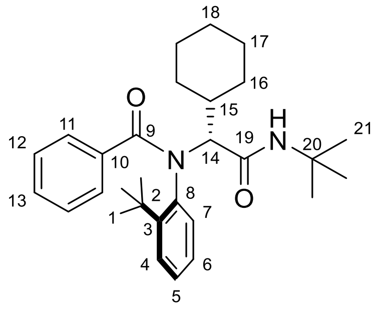

2-*tert*-Butylaniline (180 mg, 1.20 mmol, 1.0 eq.), benzoic acid (147 mg, 1.20 mmol, 1.0 eq.), cyclohexanecarboxaldehyde (145 µL, 1.20 mmol, 1.0 eq.), 2,2,2-trifluoroethanol (2.4 ml, 0.5 M) and *tert*-butyl isocyanide (136 µL, 1.20 mmol, 1.0 eq.) were subjected to **General Procedure A**. Purification *via* column chromatography eluting with petrol 40-60/Et_2O 80:20 afforded the title compound *anti*-**1n** as a pale-yellow solid (464 mg, 86% yield, >95:5 d.r.).

^{1}H NMR (300 MHz, $CDCl_3$): δ_H 7.35 – 7.14 (m, 7H, H_4, H_5, H_6, H_7, H_{11}, H_{13}), 7.14 – 7.04 (m, 2H, H_{12}), 6.05 (s, 1H, NH), 5.17 (d, *J* = 10.6 Hz, 1H, H_{14}), 2.33 (m, 1H, H_{15}), 2.24 – 2.16 (m, 1H, H_{Cy}), 1.80 (m, 3H, H_{Cy}), 1.44 – 1.15 (m, 15H, H_{21} and H_{Cy}), 1.07 (s, 9H, H_1).

^{13}C NMR (75 MHz, $CDCl_3$): δ_C 171.1, 168.3, 147.4, 137.6, 136.7, 131.4, 129.9, 129.7, 128.1, 127.4, 125.7, 67.1, 51.7, 41.2, 36.6, 32.5, 30.5, 30.5, 28.9, 26.5, 26.3, 26.2. N.B. The peak at 131.4 ppm corresponds to two overlapping signals.

FTIR (cm^{-1}): 3339, 3296, 2924, 2851, 1679, 1616, 1543, 715, 630.

Melting Point (°C): 169 – 171.

HRMS (ESI): Exact mass calculated for $C_{29}H_{41}N_2O_2^+$ [M+H]$^+$: 449.3163, found: 449.3177. Δ = 3.1 ppm.

***rac*-(R_a,*R*)-*tert*-butyl 4-(1-(N-(2-(*tert*-butyl)phenyl)benzamido)-2-(*tert*-butylamino)-2-oxoethyl)piperidine-1-carboxylate, *anti*-1o**

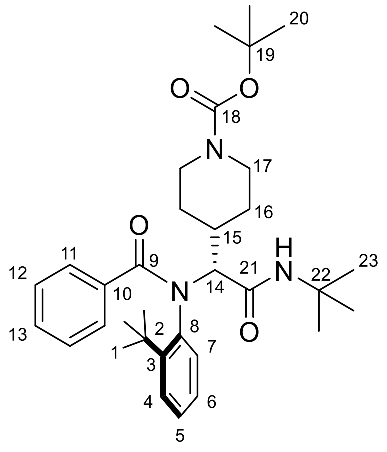

2-*tert*-Butylaniline (180 mg, 1.20 mmol, 1.0 eq.), benzoic acid (147 mg, 1.20 mmol, 1.0 eq.), piperidine-4-carboxaldehyde (256 mg, 1.20 mmol, 1.0 eq.), 2,2,2-trifluoroethanol (2.4 ml, 0.5 M) and *tert*-butyl isocyanide (136 µL, 1.20 mmol, 1.0 eq.) were subjected to **General Procedure A**. Purification *via* column chromatography eluting with petrol 40-60/Et_2O 65:35 afforded the title compound *anti*-**1o** as a white solid (413 mg, 63% yield, >95:5 d.r.).

^{1}H NMR (400 MHz, DMSO-d_6): δ_H 8.04 (s, 1H, NH), 7.33 – 7.28 (m, 1H, H_7), 7.25 – 7.14 (m, 4H, H_4, H_5, H_6 and H_{13}), 7.14 – 7.08 (m, 4H, H_{11} and H_{12}), 5.46 (d, *J* = 10.7 Hz, 1H, H_{14}), 4.07 – 3.88 (m, 2H, $H_{16/17}$), 3.00 – 2.63 (m, 2H, $H_{16/17}$), 2.41 – 2.27 (m, 1H, H_{15}), 2.10 – 2.02 (m, 1H, $H_{16/17}$), 1.40 (s, 12H, $H_{16/17}$ and H_{20}), 1.19 (s, 9H, H_{23}), 0.99 (s, 9H, H_1).

^{13}C NMR (101 MHz, DMSO-d_6): δ_C 169.7, 167.3, 153.8, 146.8, 137.3, 136.8, 131.8, 130.7, 129.1, 127.3, 127.1, 125.1, 78.5, 62.9, 50.5, 42.9 (br), 36.1, 32.0, 28.8 (br), 28.4, 28.1. N.B. A carbon is obscured by the DMSO residual solvent peak but can be observed by HSQC. The peak at 129.1 ppm corresponds to two overlapping signals.

FTIR (cm^{-1}): 3315, 2971, 1685, 1616, 1595, 1360, 1163, 776, 702.

Melting Point (°C): 174 – 176.

HRMS (ESI): Exact mass calculated for $C_{33}H_{48}N_3O_4^+$ [M+H]$^+$: 550.3639, found: 550.3648. Δ = 1.6 ppm.

***rac*-(R_a,*R*)-N-(2-(*tert*-butyl)phenyl)-N-(1-(*tert*-butylamino)-3-methyl-1-oxobutan-2-yl)benzamide, *anti*-1p**

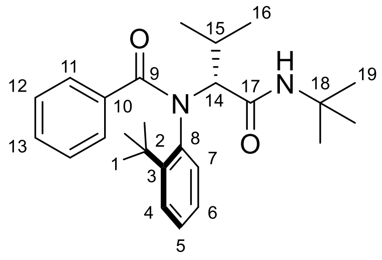

2-*tert*-Butylaniline (180 mg, 1.20 mmol, 1.0 eq.), benzoic acid (147 mg, 1.20 mmol, 1.0 eq.), isobutyraldehyde (110 µL, 1.20 mmol, 1.0 eq.), 2,2,2-trifluoroethanol (2.4 ml, 0.5 M) and *tert*-butyl isocyanide (136 µL, 1.20 mmol, 1.0 eq.) were subjected to **General Procedure A**. Purification *via* column chromatography eluting with petrol 40-60/Et_2O 70:30 afforded the title compound *anti*-**1p** as a white solid (394 mg, 80% yield, >95:5 d.r.).

^{1}H NMR (300 MHz, $CDCl_3$): δ_H 7.39 – 7.34 (m, 1H, H_7), 7.31 – 7.24 (m, 3H, H_4 and H_{11}), 7.23 – 7.15 (m, 3H, H_5, H_6 and H_{13}), 7.14 – 7.06 (m, 2H, H_{12}), 6.22 (s, 1H, NH), 5.14 (d, J = 10.6 Hz, 1H, H_{14}), 2.68 (d sext, *J* = 10.6, 6.6 Hz, 1H, H_{15}), 1.30 (d, *J* = 6.7 Hz, 3H, H_{16}), 1.26 (s, 9H, H_{19}), 1.12 (d, *J* = 6.5 Hz, 3H, $H_{16'}$), 1.08 (s, 9H, H_1).

^{13}C NMR (75 MHz, $CDCl_3$): δ_C 171.2, 168.5, 147.3, 137.7, 136.7, 131.4, 129.8, 129.7, 128.2, 127.4, 125.6, 68.1, 51.7, 36.6, 32.5, 32.1, 28.8, 20.3, 20.1. N.B. The peak at 131.4 ppm corresponds to two overlapping signals.

FTIR (cm^{-1}): 3317, 2965, 1681, 1613, 1543, 1359, 758, 716, 627.

Melting Point (°C): 173 – 175.

HRMS (ESI): Exact mass calculated for $C_{26}H_{37}N_2O_2^+$ [M+H]$^+$: 409.2850, found: 409.2849. Δ = –0.2 ppm.

rac-(R_a,R)-N-(2-(tert-butyl)phenyl)-N-(2-(tert-butylamino)-2-oxo-1-phenylethyl)-4-(trifluoromethyl)benzamide, *anti*-1q

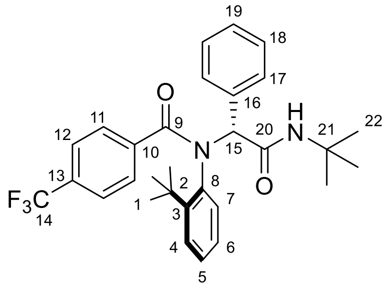

2-*tert*-Butylaniline (180 mg, 1.20 mmol, 1.0 eq.), 4-(trifluoromethyl)benzoic acid (228 mg, 1.20 mmol, 1.0 eq.), benzaldehyde (122 µL, 1.20 mmol, 1.0 eq.), 2,2,2-trifluoroethanol (2.4 ml, 0.5 M) and *tert*-butyl isocyanide (136 µL, 1.20 mmol, 1.0 eq.) were subjected to **General Procedure A**. [N.B. in this case, the crude residue obtained upon removal of 2,2,2-trifluoroethanol was directly purified via column chromatography without an aqueous workup]. Purification *via* column chromatography eluting with petrol 40-60/EtOAc 90:10 afforded the title compound *anti*-**1q** as a white solid (513 mg, 84% yield, >95:5 d.r.).

^{1}H NMR (300 MHz, CDCl$_3$): δ_H 7.99 (dd, *J* = 7.9, 2.0 Hz, 1H, H_7), 7.76 – 7.66 (m, 2H, H_{17}), 7.49 – 7.31 (m, 7H, H_{11}, H_{12}, H_{18} and H_{19}), 7.30 – 7.18 (m, 3H, H_4, H_5 and H_6), 5.60 (s, 1H, NH), 5.32 (s, 1H, H_{15}), 1.30 (s, 9H, H_{22}), 0.78 (s, 9H, H_1).

^{13}C NMR (75 MHz, CDCl$_3$): δ_C 169.3, 167.7, 146.8, 141.2, 140.1, 136.3, 132.2, 131.2 (q, *J* = 33.1 Hz), 131.1, 130.8, 130.1, 129.3, 129.2, 128.4, 127.2, 124.4 (q, *J* = 3.8 Hz), 123.7 (q, *J* = 272.3 Hz), 75.0, 51.9, 36.2, 32.2, 28.7.

^{19}F{^{1}H} NMR (282 MHz, CDCl$_3$): δ_F –63.0.

FTIR (cm^{-1}): 3301, 2966, 1683, 1638, 1324, 1314, 1164, 1127, 1066, 770, 702.

Melting Point (°C): 168 – 169.

HRMS (ESI): Exact mass calculated for $C_{30}H_{34}F_3N_2O_2^+$ [M+H]$^+$: 511.2567, found: 511.2580. Δ = 2.5 ppm.

***rac*-(R_a,*R*)-N-(2-(*tert*-butyl)phenyl)-N-(2-(*tert*-butylamino)-2-oxo-1-phenylethyl)-4-((trifluoromethyl)thio)benzamide, *anti*-1r**

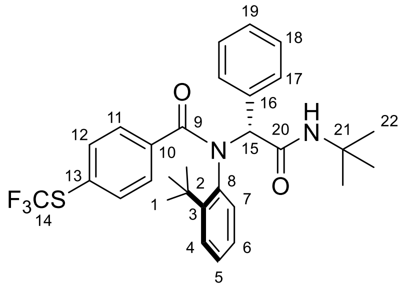

2-*tert*-Butylaniline (180 mg, 1.20 mmol, 1.0 eq.), 4-(trifluoromethylthio)benzoic acid (267 mg, 1.20 mmol, 1.0 eq.), benzaldehyde (122 µL, 1.20 mmol, 1.0 eq.), 2,2,2-trifluoroethanol (2.4 ml, 0.5 M) and *tert*-butyl isocyanide (136 µL, 1.20 mmol, 1.0 eq.) were subjected to **General Procedure A**. [N.B. in this case, the crude residue obtained upon removal of 2,2,2-trifluoroethanol was directly purified via column chromatography without an aqueous workup]. Purification via column chromatography eluting with dichloromethane, increasing to dichloromethane:MeOH 99:1 afforded the title compound *anti*-**1r** as an off-white solid (538 mg, 83% yield, >95:5 d.r.).

^{1}H NMR (300 MHz, CDCl$_3$): δ_H 7.97 (dd, *J* = 7.9, 2.1 Hz, 1H, H_7), 7.71 (dd, *J* = 6.8, 2.9 Hz, 2H, H_{17}), 7.47 – 7.35 (m, 5H, H_{12}, H_{18} and H_{19}), 7.33 – 7.19 (m, 5H, H_4, H_5, H_6 and H_{11}), 5.61 (s, 1H, NH), 5.35 (s, 1H, H_{15}), 1.30 (s, 9H, H_{22}), 0.77 (s, 9H, H_1).

^{13}C NMR (75 MHz, CDCl$_3$): δ_C 169.3, 167.7, 146.8, 141.1, 138.9, 136.3, 135.0, 132.1, 131.1, 130.9, 130.7, 129.4 (q, *J* = 308.2 Hz), 129.3, 129.2, 128.3, 127.2, 126.0 (q, *J* = 2.1 Hz), 74.9, 51.9, 36.2, 32.1, 28.7.

^{19}F NMR (282 MHz, CDCl$_3$): δ_F –42.5 (s).

FTIR (cm^{-1}): 3299, 2982, 1681, 1637, 1541, 1114, 1084, 769, 701, 618.

Melting Point (°C): 153 – 155.

HRMS (ESI): Exact mass calculated for $C_{30}H_{34}F_3N_2O_2S^+$ [M+H]$^+$: 543.2288, found: 543.2288. Δ = 0.0 ppm.

rac-(R_a,R)-N-(2-(tert-butyl)phenyl)-N-(2-(tert-butylamino)-2-oxo-1-phenylethyl)-4-methoxybenzamide, anti-1s

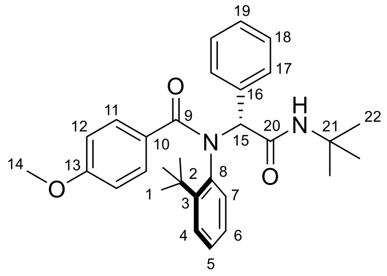

2-*tert*-Butylaniline (180 mg, 1.20 mmol, 1.0 eq.), 4-methoxybenzoic acid (183 mg, 1.20 mmol, 1.0 eq.), benzaldehyde (122 µL, 1.20 mmol, 1.0 eq.), 2,2,2-trifluoroethanol (2.4 ml, 0.5 M) and *tert*-butyl isocyanide (136 µL, 1.20 mmol, 1.0 eq.) were subjected to **General Procedure A**. Purification *via* column chromatography eluting with petrol 40-60/EtOAc 85:15 afforded the title compound *anti*-**1s** as a white solid (423 mg, 75% yield, >95:5 d.r.).

^{1}H NMR (300 MHz, $CDCl_3$): δ_H 7.85 – 7.79 (m, 1H, H_7), 7.68 (dd, J = 7.4, 2.2 Hz, 2H, H_{17}), 7.43 – 7.33 (m, 3H, H_{18} and H_{19}), 7.32 – 7.27 (m, 1H, H_4), 7.25 – 7.16 (m, 4H, H_5, H_6 and H_{11}), 6.62 – 6.54 (m, 2H, H_{12}), 5.72 (s, 1H, NH), 5.42 (s, 1H, H_{15}), 3.70 (s, 3H, H_{14}), 1.27 (s, 9H, H_{22}), 0.85 (s, 9H, H_1).

^{13}C NMR (75 MHz, $CDCl_3$): δ_C 170.0, 168.1, 160.6, 146.9, 141.6, 136.8, 132.3, 131.8, 130.8, 130.7, 129.0, 128.9, 128.7, 127.9, 126.8, 112.7, 74.3, 55.3, 51.7, 36.3, 32.2, 28.7.

FTIR (cm^{-1}): 3302, 2972, 1685, 1655, 1611, 1342, 1258, 1176, 760, 698.

Melting Point (°C): 145 – 147.

HRMS (ESI): Exact mass calculated for $C_{30}H_{37}N_2O_3^+$ [M+H]$^+$: 473.2799, found: 473.2822. Δ = 4.9 ppm.

***rac*-(R_a,*R*)-N-(2-(*tert*-butyl)phenyl)-N-(2-(*tert*-butylamino)-2-oxo-1-phenylethyl)-3-methylbenzamide, *anti*-1t**

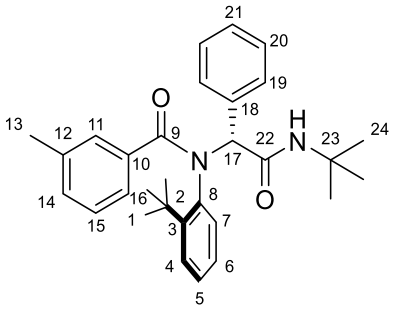

2-*tert*-Butylaniline (180 mg, 1.20 mmol, 1.0 eq.), 3-methylbenzoic acid (163 mg, 1.20 mmol, 1.0 eq.), benzaldehyde (122 µL, 1.20 mmol, 1.0 eq.), 2,2,2-trifluoroethanol (2.4 ml, 0.5 M) and *tert*-butyl isocyanide (136 µL, 1.20 mmol, 1.0 eq.) were subjected to **General Procedure A**. Purification via column chromatography eluting with petrol 40-60/EtOAc 90:10 afforded the title compound *anti*-**1t** as a pale yellow solid (463 mg, 85% yield, >95:5 d.r.).

^{1}H NMR (300 MHz, CDCl$_3$): δ_H 7.95 – 7.86 (m, 1H, H_7), 7.74 – 7.66 (m, 2H, H_{19}), 7.43 – 7.34 (m, 3H, H_{20} and H_{21}), 7.30 – 7.17 (m, 3H, H_4, H_5 and H_6), 7.11 (d, *J* = 2.0 Hz, 1H, H_{11}), 7.05 – 6.90 (m, 3H, H_{14}, H_{15} and H_{16}), 5.70 (s, 1H, NH), 5.40 (s, 1H, H_{17}), 2.13 (s, 3H, H_{13}), 1.29 (s, 9H, H_{24}), 0.82 (s, 9H, H_1).

^{13}C NMR (75 MHz, CDCl$_3$): δ_C 170.7, 168.0, 146.8, 141.5, 137.0, 136.7, 136.4, 132.4, 130.9, 130.6, 130.6, 130.3, 129.1, 128.8, 127.9, 127.2, 126.8, 126.7, 74.4, 51.7, 36.2, 32.2, 28.7, 21.2.

FTIR (cm^{-1}): 3308, 2981, 1687, 1616, 1553, 1360, 769, 701, 617.

Melting Point (^{o}C): 142 – 143.

HRMS (ESI): Exact mass calculated for $C_{30}H_{37}N_2O_2^+$ [M+H]$^+$: 457.2850 , found: 457.2854 Δ = 0.9 ppm.

***rac*-(*R_a*,*R*)-N-(2-(*tert*-butyl)phenyl)-N-(2-(*tert*-butylamino)-2-oxo-1-phenylethyl)-1-naphthamide, *anti*-1u**

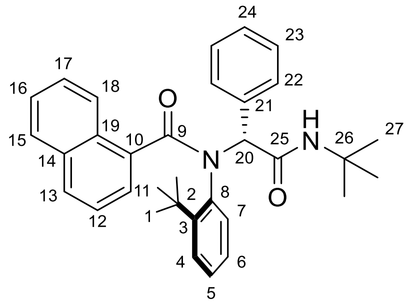

2-*tert*-Butylaniline (180 mg, 1.20 mmol, 1.0 eq.), 1-napthoic acid (207 mg, 1.20 mmol, 1.0 eq.), benzaldehyde (122 µL, 1.20 mmol, 1.0 eq.), 2,2,2-trifluoroethanol (2.4 ml, 0.5 M) and *tert*-butyl isocyanide (136 µL, 1.20 mmol, 1.0 eq.) were subjected to **General Procedure A** with a modified workup procedure. The concentrated crude reaction mixture was dissolved in dichloromethane and saturated aqueous Na_2CO_3 was added. The layers were separated, and the aqueous layer was extracted twice with dichloromethane. The combined organic extracts were dried over $MgSO_4$, filtered and the resulting solution was concentrated. Purification via column chromatography eluting with dichloromethane/Et_2O 99:1 afforded the title compound *anti*-**1u** as a pale-yellow solid (432 mg, 73% yield, >95:5 d.r.).

^{1}H NMR (300 MHz, $CDCl_3$): δ_H 8.45 (d, *J* = 8.5 Hz, 1H, H_{Ar}), 8.10 – 7.98 (m, 1H, H_7), 7.86 – 7.78 (m, 2H, H_{22}), 7.71 (d, J = 8.1 Hz, 1H, H_{Ar}), 7.62 (d, *J* = 8.2 Hz, 1H, H_{Ar}), 7.52 (ddd, *J* = 8.5, 6.8, 1.5 Hz, 1H, H_{Ar}), 7.48 – 7.38 (m, 4H, H_{Ar}), 7.30 – 7.24 (m, 1H, H_{11}), 7.20 – 7.13 (m, 1H, H_4), 7.12 – 7.01 (m, 3H, H_{Ar}), 5.80 (s, 1H, NH), 5.39 (s, 1H, H_{20}), 1.34 (s, 9H, H_{27}), 0.79 (s, 9H, H_1).

^{13}C NMR (75 MHz, $CDCl_3$): δ_C 171.1, 168.5, 146.4, 141.8, 137.0, 133.7, 132.9, 132.9, 131.4, 131.1, 130.3, 129.7, 129.2, 128.9, 128.3, 127.8, 127.4, 126.7, 126.6, 125.8, 123.6, 74.8, 51.8, 36.2, 32.4, 28.8. N.B. The peak at 126.7 ppm corresponds to two overlapping signals.

FTIR (cm^{-1}): 3308, 2971, 1692, 1613, 1552, 1360, 1257, 1212, 1166.

Melting Point (°C): 190 – 191.

HRMS (ESI): Exact mass calculated for $C_{33}H_{37}N_2O_2^+$ [M+H]$^+$: 493.2850, found: 493.2860. Δ = 2.0 ppm.

***rac*-(R_a,*R*)-N-(2-(*tert*-butyl)phenyl)-N-(2-(*tert*-butylamino)-2-oxo-1-phenylethyl)-2-fluorobenzamide, *anti*-1v**

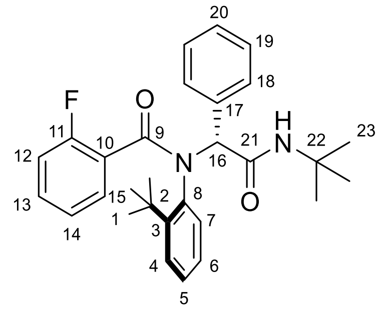

2-*tert*-Butylaniline (180 mg, 1.20 mmol, 1.0 eq.), 2-fluorobenzoic acid (168 mg, 1.20 mmol, 1.0 eq.), benzaldehyde (122 µL, 1.20 mmol, 1.0 eq.), 2,2,2-trifluoroethanol (2.4 ml, 0.5 M) and *tert*-butyl isocyanide (136 µL, 1.20 mmol, 1.0 eq.) were subjected to **General Procedure A**. [N.B. in this case, the crude residue obtained upon removal of 2,2,2-trifluoroethanol was directly purified via column chromatography without an aqueous workup]. Purification *via* column chromatography eluting with petrol 40-60/EtOAc 90:10 afforded the title compound *anti*-**1v** as a white solid (484 mg, 88% yield, >95:5 d.r.).

^{1}H NMR (300 MHz, CDCl$_3$): δ_H 7.95 – 7.90 (m, 1H, H_7), 7.71 (dd, *J* = 7.5, 2.2 Hz, 2H, H_{18}), 7.48 – 7.33 (m, 3H, H_{19} and H_{20}), 7.29 – 7.03 (m, 5H, H_4, H_5, H_6, H_{13} and H_{15}), 6.91 (ddd, *J* = 10.4, 8.3, 1.1 Hz, 1H, H_{12}), 6.79 (td, *J* = 7.6, 1.2 Hz, 1H, H_{14}), 5.69 (s, 1H, NH), 5.41 (s, 1H, H_{16}), 1.29 (s, 9H, H_{23}), 0.94 (s, 9H, H_1).

^{13}C NMR (75 MHz, CDCl$_3$): δ_C 167.9, 167.3 (d, J = 1.2 Hz), 159.7 (d, J = 251.8 Hz), 147.1, 140.4, 136.6, 132.7, 131.0 (d, J = 8.6 Hz), 130.7, 130.4, 130.4, 129.2, 128.9, 128.1, 126.5, 125.0 (d, J = 13.5 Hz), 122.9 (d, J = 3.6 Hz), 116.0 (d, J = 22.1 Hz), 73.9, 51.8, 36.3, 32.3, 28.7.

^{19}F NMR (282 MHz, CDCl$_3$): δ_F –110.0 (dt, J = 11.4, 6.3 Hz).

FTIR (cm^{-1}): 3315, 3069, 2958, 1685, 1619, 1552, 1364, 758, 700, 641.

Melting Point (°C): 171 – 173.

HRMS (ESI): Exact mass calculated for $C_{29}H_{34}FN_2O_2^+$ [M+H]$^+$: 461.2599, found: 461.2614. Δ = 3.3 ppm.

***rac*-(R_a,*R*)-2-((2-(*tert*-butyl)phenyl)(2-(*tert*-butylamino)-2-oxo-1-phenylethyl)carbamoyl)phenyl acetate, *anti*-1w**

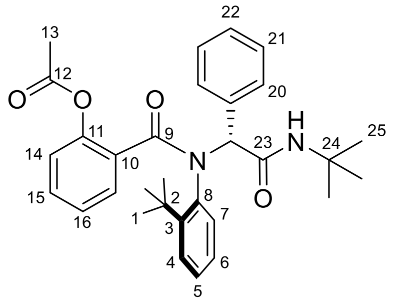

2-*tert*-Butylaniline (180 mg, 1.20 mmol, 1.0 eq.), 2-acetoxybenzoic acid (216 mg, 1.20 mmol, 1.0 eq.), benzaldehyde (122 µL, 1.20 mmol, 1.0 eq.), 2,2,2-trifluoroethanol (2.4 ml, 0.5 M) and *tert*-butyl isocyanide (136 µL, 1.20 mmol, 1.0 eq.) were subjected to **General Procedure A**. Purification via column chromatography eluting with petrol 40-60/Et_2O 70:30 afforded the title compound *anti*-**1w** as a white solid (498 mg, 83% yield, >95:5 d.r.).

^{1}H NMR (300 MHz, $CDCl_3$): δ_H 7.83 (dd, *J* = 6.0, 3.6 Hz, 1H, H_7), 7.69 (dd, *J* = 6.7, 2.9 Hz, 2H, H_{20}), 7.46 – 7.33 (m, 3H, H_{21} and H_{22}), 7.31 – 7.21 (m, 1H, H_4), 7.20 – 7.11 (m, 3H, H_5, H_6, H_{Ar}), 7.08 – 6.99 (m, 2H, H_{17} and H_{Ar}), 6.92 – 6.78 (m, 1H, H_{Ar}), 5.67 (s, 1H, NH), 5.42 (s, 1H, H_{18}), 2.31 (s, 3H, H_{13}), 1.27 (s, 9H, H_{25}), 1.00 (s, 9H, H_1).

^{13}C NMR (75 MHz, $CDCl_3$): 169.0, 167.9, 148.5, 147.0, 140.4, 137.0, 132.9, 130.6, 130.5, 129.9, 129.6, 129.2, 129.0, 128.8, 128.1, 126.4, 124.5, 123.5, 72.9, 51.7, 36.3, 32.3, 28.7, 21.3. N.B. The peak at 167.9 ppm corresponds to two overlapping signals.

FTIR (cm^{-1}): 3324, 2981, 1771, 1692, 1622, 1548, 1358, 1190, 761, 641.

Melting Point (°C): 151 – 152.

HRMS (ESI): Exact mass calculated for $C_{31}H_{37}N_2O_4^+$ [M+H]$^+$: 501.2748, found: 501.2761. Δ = 2.6 ppm.

***rac*-(R_a,*R*)-N-(2-(*tert*-butyl)phenyl)-N-(2-(*tert*-butylamino)-2-oxo-1-phenylethyl)picolinamide, *anti*-1x**

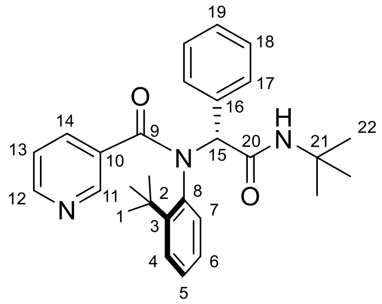

2-*tert*-Butylaniline (180 mg, 1.20 mmol, 1.0 eq.), nicotinic acid (148 mg, 1.20 mmol, 1.0 eq.), benzaldehyde (122 µL, 1.20 mmol, 1.0 eq.), 2,2,2-trifluoroethanol (2.4 ml, 0.5 M) and *tert*-butyl isocyanide (136 µL, 1.20 mmol, 1.0 eq.) were subjected to **General Procedure A**. Purification via column chromatography eluting with petrol 40-60/Et_2O 30:70 afforded the title compound *anti*-**1x** as a white solid (414 mg, 78% yield, >95:5 d.r.).

^{1}H NMR (300 MHz, $CDCl_3$): δ_H 8.52 (dd, *J* = 2.4, 0.9 Hz, 1H, H_{11}), 8.40 (dd, *J* = 4.9, 1.7 Hz, 1H, H_{12}), 8.04 – 7.95 (m, 1H, H_7), 7.75 – 7.67 (m, 2H, H_{17}), 7.48 (ddd, *J* = 8.1, 2.3, 1.7 Hz, 1H, H_{14}), 7.45 – 7.38 (m, 3H, H_{18} and H_{19}), 7.34 – 7.20 (m, 3H, H_4, H_5 and H_6), 7.01 (ddd, *J* = 8.0, 4.9, 0.9 Hz, 1H, H_{13}), 5.61 (s, 1H, NH), 5.35 (s, 1H, H_{15}), 1.30 (s, 9H, H_{22}), 0.81 (s, 9H, H_1).

^{13}C NMR (75 MHz, $CDCl_3$): δ_C 168.5, 167.6, 150.6, 150.2, 146.8, 141.0, 136.9, 136.3, 132.6, 132.1, 131.0, 130.8, 129.3, 129.2, 128.5, 127.3, 122.2, 74.8, 51.9, 36.2, 32.2, 28.7.

FTIR (cm^{-1}): 3317, 2964, 1685, 1615, 1548, 1362, 761, 701, 642.

Melting Point (°C): 156 – 158.

HRMS (ESI): Exact mass calculated for $C_{28}H_{34}N_3O_2^+$ $[M+H]^+$: 444.2646, found: 444.2662. Δ = 3.6 ppm.

***rac*-(R_a,*R*)-N-(2-(*tert*-butyl)phenyl)-N-(2-(*tert*-butylamino)-2-oxo-1-phenylethyl)furan-2-carboxamide, *anti*-1y**

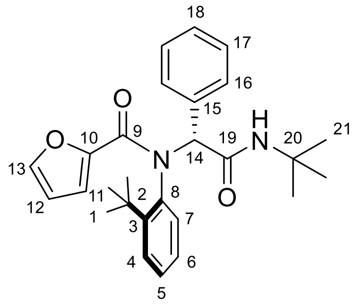

2-*tert*-Butylaniline (180 mg, 1.20 mmol, 1.0 eq.), 2-furoic acid (135 mg, 1.20 mmol, 1.0 eq.), benzaldehyde (122 µL, 1.20 mmol, 1.0 eq.), 2,2,2-trifluoroethanol (2.4 ml, 0.5 M) and *tert*-butyl isocyanide (136 µL, 1.20 mmol, 1.0 eq.) were subjected to **General Procedure A**. [N.B. in this case, the crude residue obtained upon removal of 2,2,2-trifluoroethanol was directly purified via column chromatography without an aqueous workup]. Purification via column chromatography eluting with petrol 40-60/Et_2O 70:30 afforded the title compound *anti*-**1y** as a white solid (465 mg, 90% yield, >95:5 d.r.).

^{1}H NMR (300 MHz, $CDCl_3$): δ_H 7.84 (dd, *J* = 7.8, 1.7 Hz, 1H, H_7), 7.70 – 7.64 (m, 2H, H_{16}), 7.48 (dd, *J* = 8.0, 1.7 Hz, 1H, H_4), 7.40 – 7.32 (m, 5H, H_5, H_{13}, H_{17} and H_{18}), 7.28 (td, *J* = 7.5, 1.8 Hz, 1H, H_6), 6.12 (dd, *J* = 3.6, 1.7 Hz, 1H, H_{12}), 5.70 (s, 1H, NH), 5.22 (s, 1H, H_{14}), 5.15 (dd, *J* = 3.6, 0.7 Hz, 1H, H_{11}), 1.29 (s, 9H, H_{21}), 0.98 (s, 9H, H_1).

^{13}C NMR (75 MHz, $CDCl_3$): δ_C 168.0, 161.1, 147.7, 147.5, 144.7, 141.4, 136.6, 132.7, 130.9, 129.9, 129.0, 128.8, 128.8, 127.7, 116.9, 111.1, 74.3, 51.7, 36.3, 32.2, 28.7.

FTIR (cm^{-1}): 3315, 2961, 1686, 1624, 1556, 1359, 758, 699, 559.

Melting Point (°C): 159 – 160.

HRMS (ESI): Exact mass calculated for $C_{27}H_{33}N_2O_3^+$ $[M+H]^+$: 433.2486, found: 433.2488. Δ = 0.5 ppm.

rac-(R_a,R)-N-(2-(tert-butyl)phenyl)-N-(2-(tert-butylamino)-2-oxo-1-phenylethyl)-3-phenylpropanamide, anti-1z

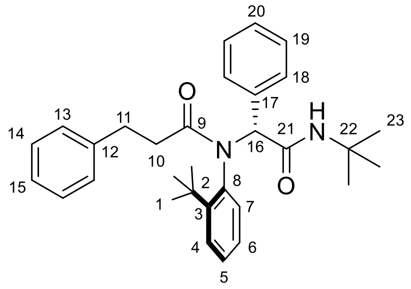

2-*tert*-Butylaniline (180 mg, 1.20 mmol, 1.0 eq.), hydrocinnamic acid (180 mg, 1.20 mmol, 1.0 eq.), benzaldehyde (122 µL, 1.20 mmol, 1.0 eq.), 2,2,2-trifluoroethanol (2.4 ml, 0.5 M) and *tert*-butyl isocyanide (136 µL, 1.20 mmol, 1.0 eq.) were subjected to **General Procedure A**. [N.B. in this case, the crude residue obtained upon removal of 2,2,2-trifluoroethanol was directly purified via column chromatography without an aqueous workup]. Purification *via* column chromatography eluting with petrol 40-60/EtOAc 90:10 afforded the title compound *anti*-**1z** as a pale-yellow solid (484 mg, 86% yield, >95:5 d.r.).

^{1}H NMR (300 MHz, CDCl$_3$): δ_H 7.66 (dd, J = 7.7, 1.8 Hz, 1H, H_7), 7.58 – 7.52 (m, 2H, H_{18}), 7.45 (dd, J = 7.9, 1.8 Hz, 1H, H_4), 7.40 – 7.31 (m, 3H, H_{19} and H_{20}), 7.28 – 7.10 (m, 5H, H_5, H_6, H_{14} and H_{15}, 7.05 (d, J = 6.7 Hz, 2H, H_{13}), 5.74 (s, 1H, NH), 5.04 (s, 1H, H_{16}), 3.15 – 2.76 (m, 2H, H_{11}), 2.37 – 2.25 (m, 2H, H_{10}), 1.29 (s, 9H, H_{23}), 1.12 (s, 9H, H_1).

^{13}C NMR (75 MHz, CDCl$_3$): δ_C 175.1, 168.6, 146.7, 142.3, 141.4, 136.9, 132.1, 130.4, 129.5, 128.9, 128.6, 128.5, 128.4, 127.7, 126.0, 73.4, 51.6, 38.7, 36.2, 32.1, 31.4, 28.7. N.B. The peak at 128.5 ppm corresponds to two overlapping signals.

FTIR (cm^{-1}): 3340, 2960, 1686, 1631, 1536, 754, 698, 541.

Melting Point (°C): 121 – 122.

HRMS (ESI): Exact mass calculated for $C_{31}H_{39}N_2O_2^+$ [M+H]$^+$: 471.3006, found: 471.3010. Δ = 0.8 ppm.

***rac*-(R_a,*R*)-N-(*tert*-butyl)-2-(N-(2-(*tert*-butyl)phenyl)acetamido)-2-phenylacetamide, *anti*-1aa**

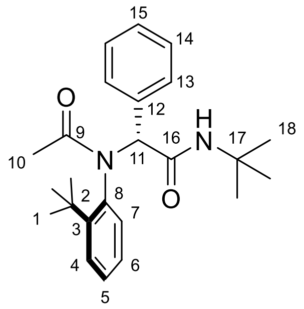

2-*tert*-Butylaniline (180 mg, 1.20 mmol, 1.0 eq.), acetic acid (69 µL, 1.20 mmol, 1.0 eq.), benzaldehyde (122 µL, 1.20 mmol, 1.0 eq.), 2,2,2-trifluroethanol (2.4 ml, 0.5 M) and *tert*-butyl isocyanide (136 µL, 1.20 mmol, 1.0 eq.) were subjected to **General Procedure A**. Purification via column chromatography eluting with petrol 40-60/Et_2O 75:25 afforded the title compound *anti*-**1aa** as a white solid (349 mg, 76% yield, >95:5 d.r.).

^{1}H NMR (300 MHz, $CDCl_3$): δ_H 7.72 (dd, *J* = 7.6, 1.8 Hz, 1H, H_7), 7.58 – 7.53 (m, 2H, H_{13}), 7.47 (dd, *J* = 7.8, 1.9 Hz, 1H, H_4), 7.38 – 7.31 (m, 3H, H_{14} and H_{15}), 7.31 – 7.19 (m, 2H, H_5 and H_6), 5.68 (s, 1H, NH), 5.04 (s, 1H, H_{11}), 1.85 (s, 3H, H_{10}), 1.27 (s, 9H, H_{18}), 1.15 (s, 9H, H_1).

^{13}C NMR (75 MHz, $CDCl_3$): δ_C 173.7, 168.5, 146.7, 143.1, 137.0, 132.1, 130.6, 129.5, 128.9, 128.6, 128.4, 127.7, 73.1, 51.6, 36.1, 32.1, 28.7, 24.8.

FTIR (cm^{-1}): 3306, 2968, 1684, 1627, 1555, 1333, 763, 702, 555.

Melting Point (°C): 131 – 133.

HRMS (ESI): Exact mass calculated for $C_{24}H_{32}N_2O_2Na^+$ $[M+Na]^+$: 403.2356, found: 403.2371. Δ = 3.7 ppm.

***rac*-(R_a,*R*)- (9H-fluoren-9-yl)methyl (2-((2-(*tert*-butyl)phenyl)(2-(*tert*-butylamino)-2-oxo-1-phenylethyl)amino)-2-oxoethyl)carbamate, *anti*-1ab**

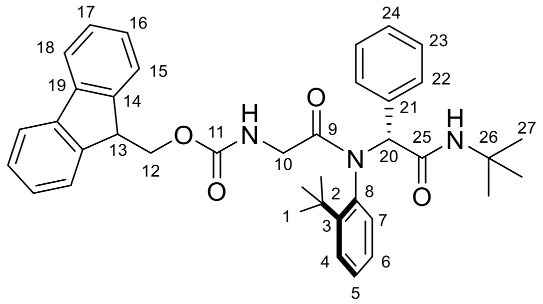

2-*tert*-Butylaniline (180 mg, 1.20 mmol, 1.0 eq.), Fmoc-glycine (357 mg, 1.20 mmol, 1.0 eq.), benzaldehyde (122 µL, 1.20 mmol, 1.0 eq.), 2,2,2-trifluoroethanol (2.4 ml, 0.5 M) and *tert*-butyl isocyanide (136 µL, 1.20 mmol, 1.0 eq.) were subjected to **General Procedure A**. Purification via column chromatography eluting with petrol 40-60/Et_2O 65:35 afforded the title compound *anti*-**1ab** as a white solid (536 mg, 72% yield, >95:5 d.r.).

^{1}H NMR (300 MHz, $CDCl_3$): δ_H 7.83 (dd, *J* = 7.5, 2.0 Hz, 1H, H_{Ar}), 7.75 (d, *J* = 7.5 Hz, 2H, H_{Ar}), 7.63 – 7.55 (m, 4H, H_{22} and H_{Ar}), 7.50 (dd, *J* = 7.7, 2.0 Hz, 1H, H_{Ar}), 7.43 – 7.23 (m, 9H, H_{Ar}), 5.70 (dd, *J* = 6.7, 2.7 Hz, 1H, NH), 5.44 (s, 1H, NH), 4.97 (s, 1H, H_{20}), 4.39 – 4.24 (m, 2H, H_{12}), 4.19 (t, *J* = 7.2 Hz, 1H, H_{13}), 3.87 (dd, *J* = 17.9, 6.6 Hz, 1H, H_{10}), 3.55 (dd, *J* = 17.9, 2.7 Hz, 1H, $H_{10'}$), 1.28 (s, 9H, H_{27}), 1.11 (s, 9H, H_1).

^{13}C NMR (75 MHz, $CDCl_3$): δ_C 171.4, 167.8, 156.0, 147.0, 144.1, 141.4, 140.4, 136.4, 131.7, 130.7, 130.1, 129.2, 129.1, 128.2, 127.7, 127.2, 125.3, 120.0, 74.3, 67.1, 51.8, 47.3, 45.5, 36.3, 32.2, 28.7. N.B. The peak at 129.2 ppm corresponds to two overlapping signals.

FTIR (cm^{-1}): 3311, 2954, 1714, 1673, 1539, 1273, 1242, 730.

Melting Point (°C): 175 – 176.

HRMS (ESI): Exact mass calculated for $C_{39}H_{44}N_3O_4^+$ [M+H]$^+$: 618.3326, found: 618.3336. Δ = 1.6 ppm.

***rac*-(R_a,*R*)-*tert*-butyl-(2-((2-(*tert*-butyl)phenyl)(2-(*tert*-butylamino)-2-oxo-1-phenylethyl)amino)-2-oxoethyl)carbamate, *anti*-1ac**

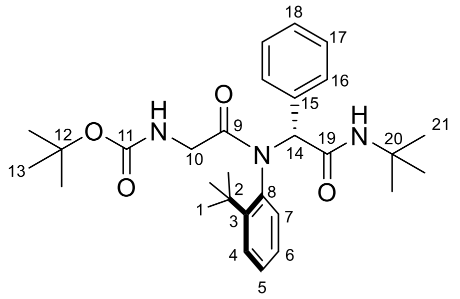

2-*tert*-Butylaniline (180 mg, 1.20 mmol, 1.0 eq.), N-Boc Glycine (210 mg, 1.20 mmol, 1.0 eq.), benzaldehyde (122 µL, 1.20 mmol, 1.0 eq.), 2,2,2-trifluoroethanol (2.4 ml, 0.5 M) and *tert*-butyl isocyanide (136 µL, 1.20 mmol, 1.0 eq.) were subjected to **General Procedure A**. Purification via column chromatography eluting with petrol 40-60/Et_2O 80:20 afforded the title compound *anti*-**1ac** as a white solid (427 mg, 72% yield, >95:5 d.r.).

^{1}H NMR (300 MHz, $CDCl_3$): δ_H 7.67 (dd, *J* = 7.6, 1.9 Hz, 1H, H_7), 7.51 – 7.44 (m, 2H, H_{16}), 7.42 – 7.35 (m, 1H, H_4), 7.30 – 7.24 (m, 3H, H_{17} and H_{18}), 7.23 – 7.09 (m, 2H, H_5 and H_6), 5.36 (s, 1H, NH), 5.31 (br s, 1H, NH), 4.88 (s, 1H, H_{14}), 3.64 (dd, *J* = 18.0, 6.1 Hz, 1H, H_{10}), 3.40 (dd, *J* = 18.0, 2.9 Hz, 1H, $H_{10'}$), 1.30 (s, 9H, H_{13}), 1.16 (s, 9H, H_{21}), 1.02 (s, 9H, H_1).

^{13}C NMR (75 MHz, $CDCl_3$): δ_C 171.9, 167.9, 155.5, 147.0, 140.4, 136.6, 131.8, 130.7, 129.9, 129.1, 129.1, 128.9, 128.1, 79.4, 74.0, 51.8, 45.2, 36.2, 32.1, 28.7, 28.4.

FTIR (cm^{-1}): 3419, 2961, 1715, 1668, 1487, 1165, 765, 710.

Melting Point (^{o}C): 163 – 165.

HRMS (ESI): Exact mass calculated for $C_{29}H_{42}N_3O_4^+$ [M+H]$^+$: 496.3170, found: 496.3188. Δ = 3.6 ppm.

rac-(_R_$_a$,_R_)-N-(_tert_-butyl)-2-(N-(2-(_tert_-butyl)phenyl)-2-(11-oxo-6,11-dihydrodibenzo[b,e]oxepin-2-yl)acetamido)-2-phenylacetamide, _anti_-1ad

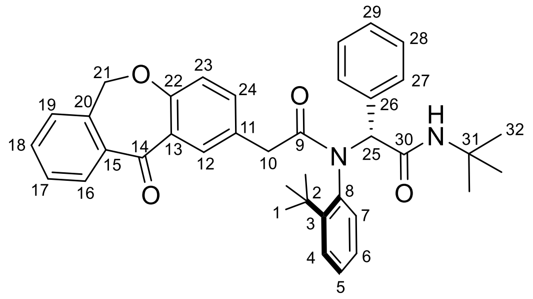

2-*tert*-Butylaniline (180 mg, 1.20 mmol, 1.0 eq.), isoxepac (322 mg, 1.20 mmol, 1.0 eq.), benzaldehyde (122 µL, 1.20 mmol, 1.0 eq.), 2,2,2-trifluoroethanol (2.4 ml, 0.5 M) and *tert*-butyl isocyanide (136 µL, 1.20 mmol, 1.0 eq.) were subjected to **General Procedure A**. Purification *via* column chromatography eluting with petrol 40-60/Et_2O 70:30 afforded the title compound *anti*-**1ad** as a yellow solid (567 mg, 80% yield, >95:5 d.r.).

^{1}H NMR (400 MHz, $CDCl_3$): δ_H 7.86 (dd, *J* = 7.7, 1.5 Hz, 1H, H_{16}), 7.80 (d, *J* = 2.3 Hz, 1H, H_{12}), 7.66 (dd, *J* = 7.9, 1.6 Hz, 1H, H_7), 7.59 – 7.49 (m, 4H, H_4, H_{27} and H_{Ar}), 7.48 – 7.42 (m, 1H, H_{17}), 7.41 (dd, *J* = 8.5, 2.4 Hz, 1H, H_{24}), 7.37 – 7.29 (m, 5H, H_5, H_{19}, H_{28} and H_{Ar}), 7.23 – 7.18 (m, 1H, H_6), 6.98 (d, *J* = 8.4 Hz, 1H, H_{23}), 5.71 (s, 1H, NH), 5.16 (s, 2H, H_{21}), 5.08 (s, 1H, H_{25}), 3.40 (s, 2H, H_{10}), 1.24 (s, 9H, H_1), 1.23 (s, 9H, H_{32}).

^{13}C NMR (101 MHz, $CDCl_3$): δ_C 190.9, 173.8, 168.6, 160.4, 146.8, 142.1, 140.7, 137.2, 136.8, 135.8, 132.7, 132.6, 130.3, 129.6, 129.5, 129.3, 129.0, 128.9, 128.8, 128.5, 127.9, 127.8, 124.9, 120.8, 73.7, 73.7, 51.6, 42.1, 36.3, 32.3, 28.6. N.B The peak at 132.7 ppm corresponds to two overlapping signals.

FTIR (cm^{-1}): 3387, 3319, 2967, 1686, 1656, 1488, 1303, 1242, 759, 696.

Melting Point (°C): 159 – 161.

HRMS (ESI): Exact mass calculated for $C_{38}H_{41}N_2O_4^+$ $[M+H]^+$: 589.3061, found: 589.3072. Δ = 1.9 ppm.

***rac*-(*R*$_a$,*R*)-2-((1-benzyl-1H-indazol-3-yl)oxy)-N-(2-(*tert*-butyl)phenyl)-N-(2-(*tert*-butylamino)-2-oxo-1-phenylethyl)acetamide, *anti*-1ae**

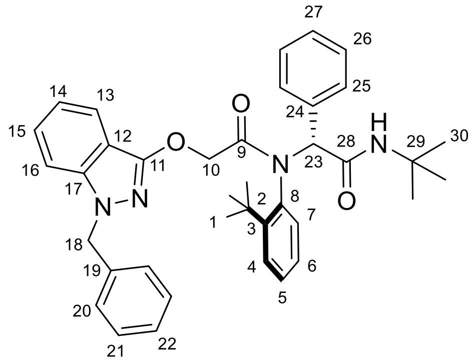

2-*tert*-Butylaniline (180 mg, 1.20 mmol, 1.0 eq.), benzadac (339 mg, 1.20 mmol, 1.0 eq.), benzaldehyde (122 µL, 1.20 mmol, 1.0 eq.), 2,2,2-trifluoroethanol (2.4 ml, 0.5 M) and *tert*-butyl isocyanide (136 µL, 1.20 mmol, 1.0 eq.) were subjected to **General Procedure A**. Purification *via* column chromatography eluting with petrol 40-60/Et_2O 75:25 afforded the title compound *anti*-**1ae** as a white solid (522 mg, 72% yield, >95:5 d.r.).

^{1}H NMR (400 MHz, $CDCl_3$): δ_H 7.74 (d, *J* = 8.1 Hz, 1H, H_{13}), 7.62 – 7.54 (m, 3H, H_7 and H_{25}), 7.51 (dd, *J* = 8.2, 1.6 Hz, 1H, H_4), 7.35 – 7.21 (m, 8H, H_5, H_{15}, H_{21}, H_{22}, H_{26}, and H_{27}), 7.17 (td, *J* = 7.5, 1.5 Hz, 1H, H_6), 7.13 – 7.08 (m, 3H, H_{16} and H_{20}), 7.05 – 6.97 (m, 1H, H_{14}), 5.79 (s, 1H, NH), 5.30 (s, 2H, H_{18}), 5.27 (s, 1H, H_{23}), 4.79 (d, *J* = 15.5 Hz, 1H, H_{10}), 4.59 (d, *J* = 15.6 Hz, 1H, $H_{10'}$), 1.26 (s, 9H, H_{30}), 1.25 (s, 9H, H_1).

^{13}C NMR (101 MHz, $CDCl_3$): 170.5, 168.0, 155.2, 147.6, 141.8, 139.2, 137.6, 136.5, 132.4, 130.5, 130.2, 129.0, 128.8, 128.6, 128.6, 127.6, 127.5, 127.4, 127.1, 120.7, 119.3, 112.9, 108.8, 72.1, 68.0, 52.4, 51.6, 36.5, 32.4, 28.7.

FTIR (cm^{-1}): 3329, 2981, 2909, 1689, 1644, 1529, 756, 700.

Melting Point (°C): 162 – 164.

HRMS (ESI): Exact mass calculated for $C_{38}H_{43}N_4O_3^+$ $[M+H]^+$: 603.3330, found: 603.3326. Δ = – 0.7 ppm.

rac-(R_a,R)-N-(tert-butyl)-2-(N-(2-(tert-butyl)phenyl)-2-(1-methyl-5-(4-methylbenzoyl)-1H-pyrrol-2-yl)acetamido)-2-phenylacetamide, anti-1af

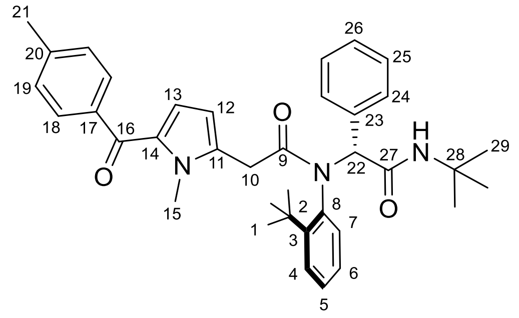

2-*tert*-Butylaniline (180 mg, 1.20 mmol, 1.0 eq.), tolmetin (309 mg, 1.20 mmol, 1.0 eq.), benzaldehyde (122 µL, 1.20 mmol, 1.0 eq.), 2,2,2-trifluoroethanol (2.4 ml, 0.5 M) and *tert*-butyl isocyanide (136 µL, 1.20 mmol, 1.0 eq.) were subjected to **General Procedure A**. Purification *via* column chromatography eluting with petrol 40-60/Et_2O 70:30 afforded the title compound *anti*-**1af** as a white solid (540 mg, 78% yield, >95:5 d.r.).

^{1}H NMR (400 MHz, $CDCl_3$): δ_H 8.08 – 8.00 (m, 1H, H_7), 7.72 – 7.68 (m, 2H, H_{18}), 7.64 – 7.56 (m, 2H, H_{24}), 7.54 – 7.49 (m, 1H, H_4), 7.40 – 7.27 (m, 5H, H_5, H_6, H_{25} and H_{26}), 7.22 (d, *J* = 7.9 Hz, 2H, H_{19}), 6.63 (d, *J* = 4.0 Hz, 1H, H_{13}), 5.93 (d, *J* = 4.0 Hz, 1H, H_{12}), 5.48 (s, 1H, NH), 4.94 (s, 1H, H_{22}), 3.86 (s, 3H, H_{15}), 3.42 (s, 2H, H_{10}), 2.41 (s, 3H, H_{21}), 1.26 (s, 9H, H_{29}), 1.16 (s, 9H, H_1).

^{13}C NMR (101 MHz, $CDCl_3$): δ_C 185.8, 172.0, 168.4, 146.8, 142.7, 141.6, 137.8, 136.7, 136.1, 132.1, 131.3, 130.8, 129.6, 129.5, 129.1, 128.9, 128.9, 128.7, 128.3, 122.5, 109.2, 74.9, 51.8, 36.2, 35.4, 33.6, 32.2, 28.6, 21.6.

FTIR (cm^{-1}): 2970, 1669, 1623, 1482, 1455, 1267, 881, 748, 705.

Melting Point (°C): 178 – 180.

HRMS (ESI): Exact mass calculated for $C_{37}H_{44}N_3O_3^+$ [M+H]$^+$: 578.3377, found: 578.3394. Δ = 2.9 ppm.

***rac*-(R_a,*R*)-N-(*tert*-butyl)-2-(N-(2-(*tert*-butyl)phenyl)-2-(1-(4-chlorobenzoyl)-5-methoxy-2-methyl-1H-indol-3-yl)acetamido)-2-phenylacetamide, *anti*-1ag**

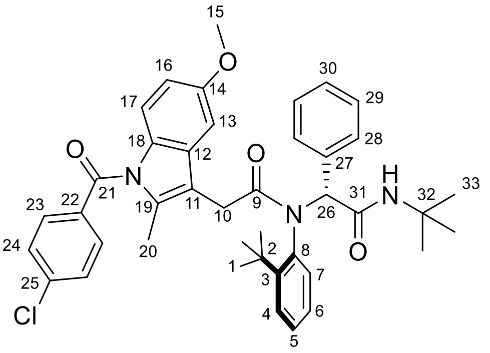

2-*tert*-Butylaniline (180 mg, 1.20 mmol, 1.0 eq.), indomethacin (429 mg, 1.20 mmol, 1.0 eq.), benzaldehyde (122 µL, 1.20 mmol, 1.0 eq.), 2,2,2-trifluoroethanol (2.4 ml, 0.5 M) and *tert*-butyl isocyanide (136 µL, 1.20 mmol, 1.0 eq.) were subjected to **General Procedure A**. Purification *via* column chromatography eluting with dichloromethane/Et_2O 98:2 afforded the title compound *anti*-**1ag** as a yellow solid (571 mg, 70% yield, >95:5 d.r.).

^{1}H NMR (400 MHz, $CDCl_3$): δ_H 7.90 (dd, *J* = 7.8, 1.8 Hz, 1H, H_7), 7.58 (d, *J* = 8.5 Hz, 2H, H_{23}), 7.54 – 7.45 (m, 3H, H_4 and H_{28}), 7.37 (d, *J* = 8.5 Hz, 2H, H_{24}), 7.23 (m, 5H, H_5, H_6, H_{29} and H_{30}), 6.85 (d, *J* = 2.5 Hz, 1H, H_{13}), 6.81 (d, *J* = 9.0 Hz, 1H, H_{17}), 6.55 (dd, *J* = 9.0, 2.5 Hz, 1H, H_{16}), 5.39 (s, 1H, NH), 4.95 (s, 1H, H_{26}), 3.77 (s, 3H, H_{15}), 3.39 (d, *J* = 17.4 Hz, 1H, H_{10}), 3.30 (d, *J* = 17.4 Hz, 1H, $H_{10'}$), 2.07 (s, 3H, H_{20}), 1.17 (s, 9H, H_1), 1.14 (s, 9H, H_{33}).

^{13}C NMR (101 MHz, $CDCl_3$): δ_C 172.7, 168.4, 168.3, 156.1, 147.1, 142.3, 139.0, 136.9, 136.0, 134.4, 132.5, 131.4, 131.3, 131.0, 130.8, 130.0, 129.1, 129.0, 128.8, 128.7, 128.1, 114.9, 114.0, 111.8, 102.0, 74.4, 55.8, 51.6, 36.4, 33.0, 32.5, 28.6, 13.8.

FTIR (cm^{-1}): 2981, 1670, 1476, 1363, 1220, 909, 728.

Melting Point (°C): 105 – 113.

HRMS (ESI): Exact mass calculated for $C_{41}H_{45}ClN_3O_4^+$ [M+H]$^+$: 678.3093, found: 678.3094. Δ = 0.1 ppm.

***rac*-(R_a,R)-N-(2-(benzylamino)-2-oxo-1-phenylethyl)-N-(2-(*tert*-butyl)phenyl)benzamide, *anti*-1ah**

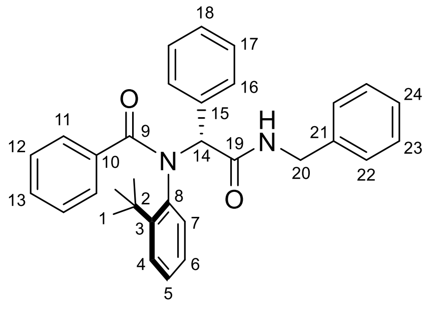

2-*tert*-Butylaniline (180 mg, 1.20 mmol, 1.0 eq.), benzoic acid (147 mg, 1.20 mmol, 1.0 eq.), benzaldehyde (122 µL, 1.20 mmol, 1.0 eq.), 2,2,2-trifluoroethanol (2.4 ml, 0.5 M) and benzyl isocyanide (146 µL, 1.20 mmol, 1.0 eq.) were subjected to **General Procedure A**. Purification via column chromatography eluting with petrol 40-60/Et_2O 75:25 afforded the title compound *anti*-**1ah** as a white solid (422 mg, 74% yield, >95:5 d.r.).

^{1}H NMR (300 MHz, $CDCl_3$): δ_H 8.08 – 8.02 (m, 1H, H_7), 7.80 – 7.73 (m, 2H, H_{16}), 7.42 – 7.35 (m, 3H, H_{17} and H_{18}), 7.34 – 7.15 (m, 11H, H_4, H_5, H_6, H_{11}, H_{13}, H_{22}, H_{23} and H_{24}), 7.13 – 7.05 (m, 2H, H_{12}), 6.12 (t, J = 5.8 Hz, 1H, NH), 5.38 (s, 1H, H_{14}), 4.61 (dd, *J* = 15.1, 6.1 Hz, 1H, H_{20}), 4.45 (dd, *J* = 15.1, 5.5 Hz, 1H, $H_{20'}$), 0.79 (s, 9H, H_1).

^{13}C NMR (75 MHz, $CDCl_3$): δ_C 170.7, 169.1, 146.7, 141.8, 138.2, 136.3, 135.8, 132.2, 131.4, 130.7, 129.8, 129.7, 129.3, 129.2, 128.7, 128.0, 127.6, 127.4, 127.2, 75.1, 44.1, 36.2, 32.2. N.B. The peak at 127.4 ppm corresponds to two overlapping signals.

FTIR (cm^{-1}): 3311, 2966, 1691, 1616, 1546, 1344, 695, 643.

Melting Point (°C): 127 – 128.

HRMS (ESI): Exact mass calculated for $C_{32}H_{33}N_2O_2^+$ $[M+H]^+$: 477.2537, found: 477.2545. Δ = 1.7 ppm.

***rac*-(R_a,*R*)-N-(2-(*tert*-butyl)phenyl)-N-(2-(cyclohexylamino)-2-oxo-1-phenylethyl)benzamide, *anti*-1ai**

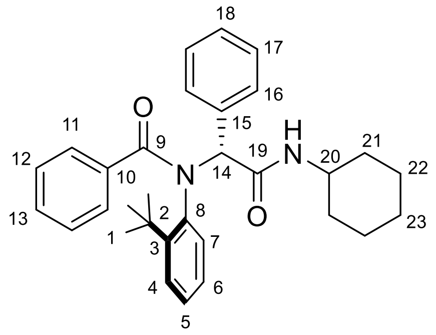

2-*tert*-Butylaniline (180 mg, 1.20 mmol, 1.0 eq.), benzoic acid (147 mg, 1.20 mmol, 1.0 eq.), benzaldehyde (122 µL, 1.20 mmol, 1.0 eq.), 2,2,2-trifluoroethanol (2.4 ml, 0.5 M) and cyclohexyl isocyanide (149 µL, 1.20 mmol, 1.0 eq.) were subjected to **General Procedure A**. Purification via column chromatography eluting with petrol 40-60/Et_2O 80:20 afforded the title compound *anti*-**1ai** as a pale yellow solid (400 mg, 71% yield, >95:5 d.r.).

^{1}H NMR (300 MHz, CDCl$_3$): δ_H 8.03 (dd, *J* = 7.9, 2.0 Hz, 1H, H_7), 7.77 – 7.69 (m, 2H, H_{16}), 7.46 – 7.37 (m, 3H, H_{17} and H_{18}), 7.31 – 7.14 (m, 6H, H_4, H_5, H_6, H_{11} and H_{13}), 7.13 – 7.04 (m, 2H, H_{12}), 5.68 (d, *J* = 8.2 Hz, 1H, NH), 5.34 (s, 1H, H_{14}), 3.93 – 3.79 (m, 1H, H_{20}), 2.00 – 1.89 (m, 1H, H_{Cy}), 1.88 – 1.78 (m, 1H, H_{Cy}), 1.63 – 0.90 (m, 8H, H_{Cy}), 0.79 (s, 9H, H_1).

^{13}C NMR (75 MHz, CDCl$_3$): δ_C 170.7, 168.1, 146.7, 141.8, 136.5, 136.2, 132.2, 131.3, 130.6, 129.8, 129.6, 129.2, 129.1, 128.0, 127.3, 127.0, 74.8, 48.9, 36.2, 32.9, 32.8, 32.2, 25.6, 24.9, 24.8.

FTIR (cm^{-1}): 3299, 2929, 2853, 1686, 1610, 1570, 1352, 715, 700.

Melting Point (°C): 123 – 125.

HRMS (ESI): Exact mass calculated for $C_{31}H_{37}N_2O_2^+$ [M+H]$^+$: 469.2850, found: 469.2841. Δ = –1.9 ppm.

***rac*-(R_a,*R*)-Ethyl (2-(N-(2-(*tert*-butyl)phenyl)benzamido)-2-phenylacetyl)glycinate, *anti*-1aj**

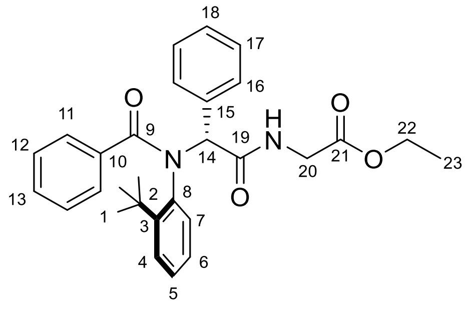

2-*tert*-Butylaniline (180 mg, 1.20 mmol, 1.0 eq.), benzoic acid (147 mg, 1.20 mmol, 1.0 eq.), benzaldehyde (122 µL, 1.20 mmol, 1.0 eq.), 2,2,2-trifluoroethanol (2.4 ml, 0.5 M) and ethyl isocyanoacetate (131 µL, 1.20 mmol, 1.0 eq.) were subjected to **General Procedure A**. Purification via column chromatography eluting with petrol 40-60/Et_2O 60:40 afforded the title compound *anti*-**1aj** as a pale yellow solid (415 mg, 73% yield, >95:5 d.r.).

^{1}H NMR (300 MHz, $CDCl_3$): δ_H 8.06 – 8.00 (m, 1H, H_7), 7.81 – 7.74 (m, 2H, H_{16}), 7.46 – 7.37 (m, 3H, H_{17} and H_{18}), 7.33 – 7.13 (m, 6H, H_4, H_5, H_6, H_{11} and H_{13}), 7.13 – 7.03 (m, 2H, H_{12}), 6.34 (t, *J* = 5.1 Hz, 1H, NH), 5.34 (s, 1H, H_{14}), 4.21 – 4.10 (m, 3H, H_{20} and H_{22}), 4.03 (dd, *J* = 18.4, 4.8 Hz, 1H, $H_{20'}$), 1.24 (t, *J* = 7.1 Hz, 3H, H_{23}), 0.78 (s, 9H, H_1).

^{13}C NMR (75 MHz, $CDCl_3$): δ_C 170.6, 169.7, 169.3, 146.7, 141.8, 136.2, 135.5, 132.0, 131.4, 130.6, 129.8, 129.3, 129.3, 128.1, 127.4, 127.3, 75.0, 61.6, 42.0, 36.1, 32.2, 14.2. N.B. The peak at 129.8 ppm corresponds to two overlapping signals.

FTIR (cm^{-1}): 3363, 2957, 1727, 1689, 1626, 1324, 1204, 719, 693.

Melting Point (°C): 122 – 124.

HRMS (ESI): Exact mass calculated for $C_{29}H_{33}N_2O_4^+$ [M+H]$^+$: 473.2435, found: 473.2436. Δ = 0.2 ppm.

***rac*-(R_a,*R*)-ethyl (2-(2-((((9H-fluoren-9-yl)methoxy)carbonyl)amino)-N-(2-(*tert*-butyl)phenyl) acetamido)-2-phenylacetyl)glycinate, *anti*-1ak**

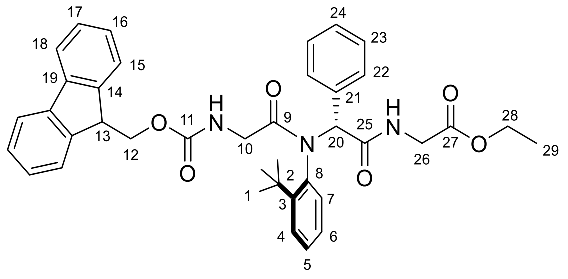

2-*tert*-Butylaniline (180 mg, 1.20 mmol, 1.0 eq.), Fmoc-Glycine (357 mg, 1.20 mmol, 1.0 eq.), benzaldehyde (122 µL, 1.20 mmol, 1.0 eq.), 2,2,2-trifluoroethanol (2.4 ml, 0.5 M) and ethyl isocyanoacetate (131 µL, 1.20 mmol, 1.0 eq.) were subjected to **General Procedure A**. Purification via column chromatography eluting with petrol 40-60/Et_2O 40:60 afforded the title compound *anti*-**1ak** as a white solid (530 mg, 68% yield, >95:5 d.r.).

^{1}H NMR (300 MHz, $CDCl_3$): δ_H 7.96 – 7.90 (m, 1H, H_7), 7.75 (d, *J* = 7.5 Hz, 2H, H_{Ar}), 7.69 – 7.64 (m, 2H, H_{22}), 7.62 – 7.56 (m, 2H, H_{Ar}), 7.51 (dd, *J* = 7.1, 2.5 Hz, 1H, H_4), 7.45 – 7.24 (m, 9H, H_5, H_6, H_{23}, H_{24} and H_{Ar}), 6.10 (t, *J* = 5.0 Hz, 1H, NH), 5.69 (m, 1H, NH), 4.97 (s, 1H, H_{20}), 4.40 – 4.24 (m, 2H, H_{12}), 4.23 – 3.80 (m, 6H, H_{10}, H_{13}, H_{26} and H_{28}), 3.58 (dd, *J* = 18.0, 2.7 Hz, 1H, $H_{10'}$), 1.24 (t, *J* = 7.1 Hz, 3H, H_{29}), 1.09 (s, 9H, H_1).

^{13}C NMR (75 MHz, $CDCl_3$): δ_C 171.8, 169.5, 169.1, 156.0, 146.9, 144.0, 141.4, 140.7, 135.4, 131.4, 131.1, 130.0, 129.5, 129.4, 129.4, 128.6, 127.8, 127.2, 125.3, 120.0, 76.7, 67.1, 61.7, 47.2, 45.5, 42.0, 36.2, 32.1, 14.2.

FTIR (cm^{-1}): 3294, 2951, 1756, 1674, 1546, 1199, 734.

Melting Point (°C): 142 – 144.

HRMS (ESI): Exact mass calculated for $C_{39}H_{42}N_3O_6^+$ [M+H]$^+$: 648.3068, found: 648.3079. Δ = 1.7 ppm.

N-(2-(*tert*-butyl)phenyl)-N-((*R*, *R*$_a$)-2-oxo-1-phenyl-2-(((*S*)-1-phenylethyl)amino)ethyl)benzamide & N-(2-(*tert*-butyl)phenyl)-N-((*S*, *S*$_a$)-2-oxo-1-phenyl-2-(((*S*)-1-phenylethyl)amino)ethyl)benzamide, *anti*-1al

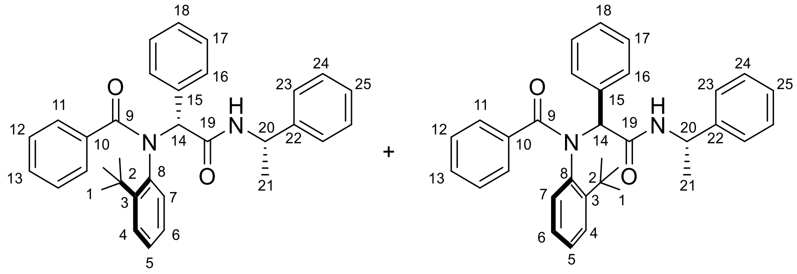

2-*tert*-Butylaniline (180 mg, 1.20 mmol, 1.0 eq.), benzoic acid (147 mg, 1.20 mmol, 1.0 eq.), benzaldehyde (122 µL, 1.20 mmol, 1.0 eq.), 2,2,2-trifluoroethanol (2.4 ml, 0.5 M) and (*S*)-(-)-α-methylbenzyl isocyanide (162 µL, 1.20 mmol, 1.0 eq.) were subjected to **General Procedure A**. Purification via column chromatography eluting with petrol 40-60/Et_2O 80:20 afforded the title compound *anti*-**1al** as a white solid (420 mg, 71% yield, 53:47 d.r.). A 30 mg analytical sample of this mixture was further purified using preparative TLC (dichloromethane/Et_2O 98:2) to yield 13 mg of each diastereoisomer (both >95:5 d.r.).

Diastereoisomer 1:

R_f (dichloromethane/Et_2O 98:2): 0.54.

^{1}H NMR (400 MHz, $CDCl_3$): δ_H 7.84 – 7.78 (m, 1H, H_7), 7.74 – 7.68 (m, 2H, H_{16}), 7.41 – 7.34 (m, 3H, H_{17} and H_{18}), 7.34 – 7.25 (m, 7H, H_4, H_{11}, H_{23} and H_{Ar}), 7.25 – 7.15 (m, 4H, H_6 and H_{Ar}), 7.09 (dd, *J* = 8.3, 6.9 Hz, 2H, H_{12}), 6.18 (d, *J* = 7.9 Hz, 1H, NH), 5.44 (s, 1H, H_{14}), 5.23 (p, *J* = 7.1 Hz, 1H, H_{20}), 1.43 (d, *J* = 6.9 Hz, 3H, H_{21}), 0.85 (s, 9H, H_1).

^{13}C NMR (101 MHz, $CDCl_3$): δ_C 170.8, 168.2, 146.7, 142.8, 141.5, 136.4, 135.8, 132.3, 131.1, 130.7, 129.8, 129.7, 129.1, 129.0, 128.7, 128.0, 127.4, 127.3, 127.0, 126.3, 74.5, 49.1, 36.2, 32.3, 21.5.

FTIR (cm^{-1}): 3324, 3062, 2962, 2927, 1679, 1616, 1491, 729, 697.

HRMS (ESI): Exact mass calculated for $C_{33}H_{35}N_2O_2^+$ [M+H]$^+$: 491.2693, found: 491.2679. Δ = –2.8 ppm.

$[\alpha]_D{}^{20}$: –126.2 (c = 0.01, $CHCl_3$).

Diastereoisomer 2:

R_f (dichloromethane/Et_2O 98:2): 0.46.

^{1}H NMR (400 MHz, CDCl$_3$): δ_H 8.00 (dd, *J* = 7.5, 1.9 Hz, 1H, H_7), 7.78 – 7.70 (m, 2H, H_{16}), 7.47 – 7.35 (m, 3H, H_{17} and H_{18}), 7.32 – 7.15 (m, 11H, H_4, H_5, H_6, H_{11}, H_{13}, H_{23}, H_{24} and H_{25}), 7.09 (dd, *J* = 8.3, 7.0 Hz, 2H, H_{12}), 6.04 (d, *J* = 7.9 Hz, 1H, NH), 5.36 (s, 1H, H_{14}), 5.20 (p, *J* = 7.1 Hz, 1H, H_{20}), 1.44 (d, *J* = 7.0 Hz, 3H, H_{21}), 0.77 (s, 9H, H_1).

^{13}C NMR (101 MHz, CDCl$_3$): δ_C 170.8, 168.2, 146.8, 143.4, 141.6, 136.4, 136.0, 132.3, 131.2, 130.7, 129.8, 129.7, 129.3, 129.2, 128.7, 128.0, 127.4, 127.3, 127.1, 126.1, 74.7, 49.5, 36.2, 32.2, 22.2.

FTIR (cm^{-1}): 3426, 3304, 3062, 2966, 1680, 1616, 1490, 728, 697.

HRMS (ESI): Exact mass calculated for $C_{33}H_{35}N_2O_2^+$ [M+H]$^+$: 491.2693, found: 491.2688. Δ = –1.0 ppm.

$[\alpha]_D^{20}$: +161.0 (c = 0.01, CHCl$_3$).

***rac*-(R_a,*R*)-N-(2-(*tert*-butylamino)-2-oxo-1-phenylethyl)-N-(2-iodo-4,6-dimethylphenyl)benzamide, *anti*-1am**

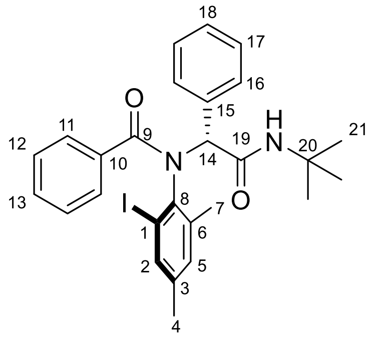

Synthesised according to a modification to **General Procedure A.** A round bottomed flask equipped with a stirrer bar, was sequentially charged with 2-iodo-4,6-dimethylaniline (75 mg, 0.30 mmol, 1.0 eq.), benzoic acid (37 mg, 0.30 mmol, 1.0 eq.), benzaldehyde (31 µL, 0.30 mmol, 1.0 eq.), 2,2,2-trifluoroethanol (0.6 mL, 0.5 M) and *tert*-butyl isocyanide (34 µL, 0.30 mmol, 1.0 eq.). The resulting mixture was stirred at room temperature for 24 hours. After this time a further portion of *tert*-butyl isocyanide (34 µL, 0.30 mmol, 1.0 eq.) was charged and the mixture was stirred for a further 24 hours. The reaction mixture was concentrated to dryness, followed by addition of EtOAc and saturated aqueous Na_2CO_3. The layers were separated, and the aqueous layer was extracted twice with EtOAc. The combined organic extracts were dried over $MgSO_4$, filtered and the resulting solution was concentrated. Purification via column chromatography eluting with petrol 40-60/Et_2O 70:30 afforded the title compound *anti*-**1am** as a white solid (114 mg, 70% yield, >95:5 d.r.).

^{1}H NMR (300 MHz, $CDCl_3$): δ_H 7.57 – 7.50 (m, 4H, H_{11} and H_{16}), 7.38 – 7.35 (m, 1H, H_2), 7.31 – 7.23 (m, 4H, H_{13}, H_{17} and H_{18}), 7.22 – 7.14 (m, 2H, H_{12}), 6.98 (s, 1H, NH), 6.83 – 6.78 (m, 1H, H_5), 5.19 (s, 1H, H_{14}), 2.37 (s, 3H, H_7), 2.13 (s, 3H, H_4), 1.38 (s, 9H, H_{21}).

^{13}C NMR (75 MHz, $CDCl_3$): δ_C 171.7, 168.1, 141.3, 139.6, 139.1, 138.5, 135.8, 134.6, 132.7, 130.6, 130.1, 128.7, 128.2, 128.1, 127.6, 101.9, 71.9, 51.6, 28.8, 20.3, 20.0.

FTIR (cm^{-1}): 3338, 2964, 2924, 1686, 1634, 1546, 1338, 717, 698, 585.

Melting Point (°C): 133 – 135.

HRMS (ESI): Exact mass calculated for $C_{27}H_{30}IN_2O_2^+$ [M+H]$^+$: 541.1347, found: 541.1345. Δ = –0.4 ppm.

***rac*-(R_a,*R*)-N-(2-(*tert*-butylamino)-2-oxo-1-phenylethyl)-N-(2-iodo-4,6-dimethylphenyl)-3-phenylpropanamide, *anti*-1an**

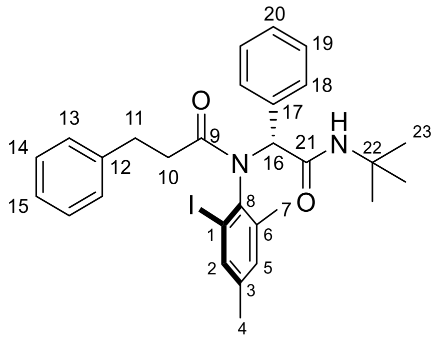

Synthesised according to a modification to **General Procedure A.** A round bottomed flask equipped with a stirrer bar, was sequentially charged with 2-iodo-4,6-dimethylaniline (75 mg, 0.30 mmol, 1.0 eq.), hydrocinnamic acid (46 mg, 0.30 mmol, 1.0 eq.), benzaldehyde (31 µL, 0.30 mmol, 1.0 eq.), 2,2,2-trifluoroethanol (0.6 mL, 0.5 M) and *tert*-butyl isocyanide (34 µL, 0.30 mmol, 1.0 eq.). The resulting mixture was stirred at room temperature for 24 hours. After this time a further portion of *tert*-butyl isocyanide (34 µL, 0.30 mmol, 1.0 eq.) was charged and the mixture was stirred for a further 24 hours. The reaction mixture was concentrated to dryness, followed by addition of EtOAc and saturated aqueous Na_2CO_3. The layers were separated, and the aqueous layer was extracted twice with EtOAc. The combined organic extracts were dried over $MgSO_4$, filtered and the resulting solution was concentrated. Purification via column chromatography eluting with petrol 40-60/Et_2O 70:30 afforded the title compound *anti*-**1an** as an off-white solid (124 mg, 73% yield, >95:5 d.r.).

^{1}H NMR (400 MHz, CDCl$_3$) δ_H 7.49 – 7.47 (m, 1H, H_2), 7.36 – 7.33 (m, 2H, H_{18}), 7.25 – 7.21 (m, 2H, H_{14}), 7.19 – 7.12 (m, 6H, H_{13}, H_{15}, H_{19}, H_{20}), 6.80 – 6.77 (m, 1H, H_5), 6.76 (s, 1H, NH), 5.27 (s, 1H, H_{16}), 3.02 – 2.94 (m, 2H, H_{11} + $H_{11'}$), 2.44 (ddd, *J* = 16.5, 8.4, 7.0 Hz, 1H, H_{10}), 2.20 – 2.08 (m, 4H, H_4, $H_{10'}$), 2.01 (s, 3H, H_7), 1.33 (s, 9H, H_{23}).

^{13}C NMR (75 MHz, CDCl$_3$) δ_C 173.9, 167.9, 141.3, 140.2, 139.6, 138.9, 138.6, 134.5, 132.6, 130.7, 128.8, 128.5, 128.4, 128.0, 126.2, 103.4, 69.6, 51.6, 37.4, 31.2, 28.8, 20.4, 19.8.

FTIR (cm^{-1}): 3390, 2966, 2924, 1671, 1517, 1496, 1453, 1226, 699.

Melting point (°C): 144 –146.

HRMS (ESI): Exact mass calculated for $C_{29}H_{34}IN_2O_2^+$ [M+H]$^+$ 569.1659, found 569.1665. Δ = 1.1 ppm.

***rac*-(R_a,*R*)-N-(2-(*tert*-butylamino)-2-oxo-1-(m-tolyl)ethyl)-N-(2-iodo-4,6-dimethylphenyl)benzamide, *anti*-1ao**

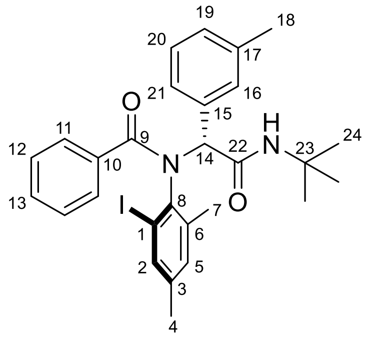

Synthesised according to a modification to **General Procedure A.** A round bottomed flask equipped with a stirrer bar, was sequentially charged with 2-iodo-4,6-dimethylaniline (75 mg, 0.30 mmol, 1.0 eq.), benzoic acid (37 mg, 0.30 mmol, 1.0 eq.), 3-methylbenzaldehyde (35 µL, 0.30 mmol, 1.0 eq.), 2,2,2-trifluoroethanol (0.6 mL, 0.5 M) and *tert*-butyl isocyanide (34 µL, 0.30 mmol, 1.0 eq.). The resulting mixture was stirred at room temperature for 24 hours. After this time a further portion of *tert*-butyl isocyanide (34 µL, 0.30 mmol, 1.0 eq.) was charged and the mixture was stirred for a further 24 hours. The reaction mixture was concentrated to dryness, followed by addition of EtOAc and saturated aqueous Na_2CO_3. The layers were separated, and the aqueous layer was extracted twice with EtOAc. The combined organic extracts were dried over $MgSO_4$, filtered and the resulting solution was concentrated. Purification via column chromatography eluting with petrol 40-60/Et_2O 70:30 afforded the title compound *anti*-**1ao** as a white solid (129 mg, 78% yield, >95:5 d.r.).

^{1}H NMR (400 MHz, $CDCl_3$) δ_H 7.56 – 7.51 (m, 2H, H_{11}), 7.38 – 7.36 (m, 1H, H_2), 7.35 – 7.27 (m, 3H, H_{13}, H_{20}, H_{21}), 7.20 – 7.11 (m, 3H, H_{16} and H_{12}), 7.08 – 7.04 (m, 1H, H_{19}), 6.99 (s, 1H, NH), 6.82 – 6.79 (m, 1H, H_5), 5.17 (s, 1H, H_{14}), 2.35 (s, 3H, H_7), 2.29 (s, 3H, H_{18}), 2.12 (s, 3H, H_4), 1.37 (s, 9H, H_{24}).

^{13}C NMR (75 MHz, $CDCl_3$) δ_C 171.7, 168.2, 141.3, 139.5, 139.0, 138.5, 137.6, 135.9, 134.4, 132.6, 130.8, 130.6, 128.9, 128.7, 127.9, 127.6, 127.3, 102.0, 71.7, 51.5, 28.8, 21.6, 20.3, 20.1.

FTIR (cm^{-1}): 3340, 2972, 2905, 1679, 1623, 1363, 1243, 773.

Melting point (°C): 132 – 135.

HRMS (ESI): Exact mass calculated for $C_{28}H_{32}IN_2O_2^+$ $[M+H]^+$ 555.1503, found 555.1501. Δ = – 0.4 ppm.

rac-(R_a,R)-N-(2-bromo-4,6-dimethylphenyl)-N-(2-(*tert*-butylamino)-2-oxo-1-phenylethyl)benzamide, *anti*-1ap

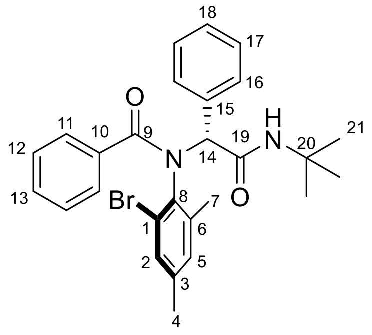

2-Bromo-4,6-dimethylaniline (60 mg, 0.30 mmol, 1.0 eq.), benzoic acid (37 mg, 0.30 mmol, 1.0 eq.), benzaldehyde (31 µL, 0.30 mmol, 1.0 eq.), 2,2,2-trifluoroethanol (0.6 ml, 0.5 M) and *tert*-butyl isocyanide (34 µL, 0.30 mmol, 1.0 eq.) were subjected to **General Procedure A**. [N.B. in this case, the crude residue obtained upon removal of 2,2,2-trifluoroethanol was directly purified via column chromatography without an aqueous workup]. Purification *via* column chromatography eluting with petrol 40-60/Et_2O 65:35 afforded the title compound *anti*-**1ap** as a pale-yellow oil (91 mg, 62% yield, 90:10 d.r.). An analytically pure sample of the *anti*-diastereoisomer was obtained by further column chromatography eluting with petrol 40-60/Et_2O 70:30.

^{1}H NMR (300 MHz, $CDCl_3$): δ_H 7.52 – 7.48 (m, 2H, H_{11}), 7.46 (dd, J = 6.7, 2.9 Hz, 2H, H_{16}), 7.29 – 7.19 (m, 4H, H_{13}, H_{17} and H_{18}), 7.16 (t, J = 7.7 Hz, 2H, H_{12}), 7.13 (s, 1H, NH), 7.07 (d, J = 2.0 Hz, 1H, H_2), 6.73 – 6.68 (m, 1H, H_5), 5.30 (s, 1H, H_{14}), 2.22 (s, 3H, H_7), 2.12 (s, 3H, H_4), 1.37 (s, 9H, H_{21}).

^{13}C NMR (75 MHz, $CDCl_3$): δ_C 172.1, 168.4, 139.6, 138.9, 138.0, 135.8, 134.5, 132.0, 131.6, 130.6, 129.6, 128.1, 128.1, 127.7, 124.7, 71.3, 51.5, 28.8, 20.6, 19.5. N.B. Peak at 128.1 ppm corresponds to two overlapping signals.

FTIR (cm^{-1}): 2971, 1669, 1640, 1454, 1392, 1364, 1307, 1225, 728, 698.

Melting Point (°C): 149 – 151

HRMS (ESI): Exact mass calculated for $C_{27}H_{30}BrN_2O_2^+$ $[M+H]^+$: 493.1485, found: 493.1482. Δ = -0.6 ppm.

***rac*-(R_a,*R*)-N-(2-(*tert*-butylamino)-2-oxo-1-phenylethyl)-N-(2-chloro-4,6-dimethylphenyl)benzamide, *anti*-1aq**

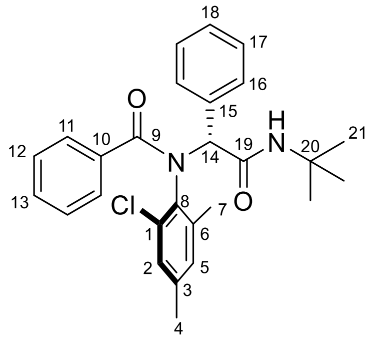

2-Chloro-4,6-dimethylaniline (47 mg, 0.30 mmol, 1.0 eq.), benzoic acid (37 mg, 0.30 mmol, 1.0 eq.), benzaldehyde (31 µL, 0.30 mmol, 1.0 eq.), 2,2,2-trifluoroethanol (0.6 ml, 0.5 M) and *tert*-butyl isocyanide (34 µL, 0.30 mmol, 1.0 eq.) were subjected to **General Procedure A**. [N.B. in this case, the crude residue obtained upon removal of 2,2,2-trifluoroethanol was directly purified via column chromatography without an aqueous workup]. Purification *via* column chromatography eluting with petrol 40-60/Et_2O 70:30 afforded the title compound *anti*-**1aq** as a white solid (96 mg, 71% yield, 77:23 d.r.). An analytically pure sample of the *anti*-diastereoisomer was obtained by further column chromatography eluting with petrol 40-60/Et_2O 70:30.

^{1}H NMR (400 MHz, $CDCl_3$): δ_H 7.51 – 7.41 (m, 4H, H_{11} and H_{16}), 7.30 – 7.09 (m, 7H, H_{12}, H_{13}, H_{17}, H_{18} and NH), 6.90 (d, J = 2.1 Hz, 1H, H_2), 6.64 (d, J = 2.1 Hz, 1H, H_5), 5.38 (s, 1H, H_{14}), 2.16 (s, 3H, H_7), 2.12 (s, 3H, H_4), 1.39 (s, 9H, H_{21}).

^{13}C NMR (101 MHz, $CDCl_3$): δ_C 172.3, 168.5, 139.3, 138.7, 136.5, 135.9, 134.4, 133.5, 130.7, 130.6, 129.4, 128.6, 128.2, 128.1, 127.8, 127.7, 70.8, 51.5, 28.8, 20.8, 19.1.

FTIR (cm^{-1}): 3301, 2965, 2926, 1667, 1520, 1454, 1324, 1309, 1226, 731, 699.

Melting Point (°C): 60 – 61.

HRMS (ESI): Exact mass calculated for $C_{27}H_{30}ClN_2O_2^+$ $[M+H]^+$: 449.1991, found: 449.1987. Δ = -0.9 ppm.

rac-(R_a,*R*) N-(2-(*tert*-butylamino)-2-oxo-1-phenylethyl)-N-(3,5-dimethyl-[1,1'-biphenyl]-2-yl)benzamide, *anti*-1ar

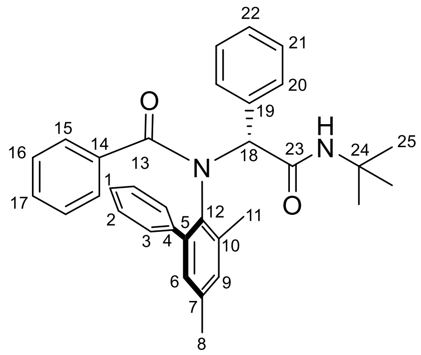

3,5-Dimethyl-[1,1'-biphenyl]-2-amine (59 mg, 0.30 mmol, 1.0 eq.), benzoic acid (37 mg, 0.30 mmol, 1.0 eq.), benzaldehyde (31 µL, 0.30 mmol, 1.0 eq.), 2,2,2-trifluoroethanol (0.6 ml, 0.5 M) and *tert*-butyl isocyanide (34 µL, 0.30 mmol, 1.0 eq.) were subjected to **General Procedure A**. [N.B. in this case, the crude residue obtained upon removal of 2,2,2-trifluoroethanol was directly purified via column chromatography without an aqueous workup]. Purification *via* column chromatography eluting with petrol 40-60/Et_2O 70:30 afforded the title compound *anti*-**1ar** as a pale-yellow oil (136 mg, 92% yield, >95:5 d.r.).

^{1}H NMR (400 MHz, $CDCl_3$): δ_H 7.37 (dd, J = 8.0, 1.4 Hz, 2H, H_{15}), 7.33 – 7.28 (m, 1H, H_{17}), 7.21 – 7.03 (m, 8H, H_1, H_{16}, H_{20}, H_{21} and H_{22}), 7.00 (d, J = 2.1 Hz, 1H, H_9), 6.97 (t, J = 7.6 Hz, 2H, H_2), 6.74 – 6.71 (m, 2H, NH and H_6), 6.59 (d, J = 7.2 Hz, 2H, H_3), 4.95 (s, 1H, H_{18}), 2.48 (s, 3H, H_{11}), 2.27 (s, 3H, H_8), 1.29 (s, 9H, H_{25}).

^{13}C NMR (101 MHz, $CDCl_3$): δ_C 170.3, 168.7, 140.5, 139.5, 138.5, 137.7, 136.4, 135.6, 135.4, 132.2, 131.1, 130.6, 129.4, 129.3, 128.8, 128.2, 127.9, 127.6, 127.6, 127.1, 74.8, 51.3, 28.7, 20.9, 19.2.

FTIR (cm^{-1}): 3287, 2970, 1673, 1642, 1512, 1448, 1364, 1306, 1226, 729, 698.

HRMS (ESI): Exact mass calculated for $C_{33}H_{35}N_2O_2^+$ [M+H]$^+$: 491.2693, found: 491.2689. Δ = -0.8 ppm.

***rac*-(R_a,R)-N-(8-Bromonaphthalen-1-yl)-N-(2-(tert-butylamino)-2-oxo-1-phenylethyl)benzamide, *anti*-1as**

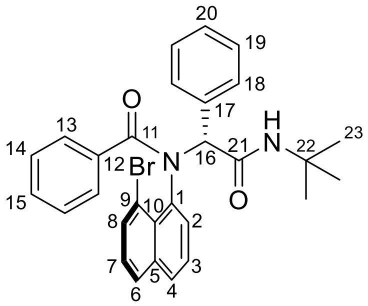

8-Bromonaphthalen-1-amine (67 mg, 0.30 mmol, 1.0 eq.), benzoic acid (37 mg, 0.30 mmol, 1.0 eq.), benzaldehyde (31 µL, 0.30 mmol, 1.0 eq.), 2,2,2-trifluoroethanol (0.6 mL, 0.5 M) and *tert*-butyl isocyanide (34 µL, 0.30 mmol, 1.0 eq.) were subjected to **General Procedure A**. Purification *via* column chromatography eluting with petrol 40-60/Et_2O 65:35 afforded the title compound *anti*-**1as** as a brown solid (94 mg, 61% yield, 95:5 d.r.).

^{1}H NMR (400 MHz, $CDCl_3$): δ_H 7.74 – 7.69 (m, 2H, H_4 and H_8), 7.69 – 7.64 (m, 3H, H_6 and H_{18}), 7.54 (dd, J = 7.5, 1.3 Hz, 1H, H_2), 7.42 (t, J = 7.4 Hz, 2H, H_{19}), 7.39 – 7.33 (m, 2H, H_3 and H_{20}), 7.24 – 7.20 (m, 2H, H_{13}), 7.13 (t, J = 7.8 Hz, 1H, H_7), 7.00 – 6.95 (m, 1H, H_{15}), 6.91 (t, J = 7.5 Hz, 2H, H_{14}), 6.30 (s, 1H, H_{16}), 6.06 (s, 1H, N-H), 1.00 (s, 9H, H_{23}).

^{13}C NMR (101 MHz, $CDCl_3$): δ_C 171.1, 166.9, 137.4, 137.3, 136.5, 136.4, 134.5, 131.2, 130.2, 129.5, 129.1, 129.1, 128.7, 128.2, 128.1, 127.3, 126.1, 125.5, 116.9, 68.1, 51.5, 28.2. N.B. Peak at 129.5 ppm corresponds to two overlapping signals.

FTIR (cm^{-1}): 3331, 3061, 2969, 1687, 1627, 1363.

Melting Point (°C): 196-199.

HRMS (ESI): Exact mass calculated for $C_{29}H_{28}BrN_2O_2^+$ $[M+H]^+$: 515.1329, found 515.1325. Δ = - 0.8 ppm.

rac-(S_a,_R_)-N-(2-(tert-Butylamino)-2-oxo-1-phenylethyl)-N-(2-methylnaphthalen-1-yl)benzamide, _anti_-1at

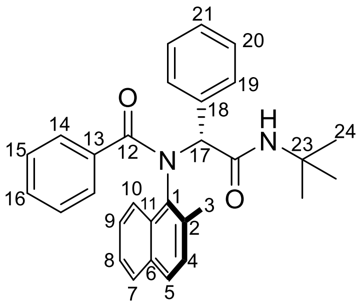

2-methyl-1-naphthylamine (47 mg, 0.30 mmol, 1.0 eq.), benzoic acid (37 mg, 0.30 mmol, 1.0 eq.), benzaldehyde (31 µL, 0.30 mmol, 1.0 eq.), 2,2,2-trifluoroethanol (0.6 mL, 0.5 M) and *tert*-butyl isocyanide (34 µL, 0.30 mmol, 1.0 eq.) were subjected to **General Procedure A**. Purification *via* column chromatography eluting with petrol 40-60/Et_2O 70:30 afforded the title compound *anti*-**1at** as a white solid (78 mg, 58% yield, 90:10 d.r.). An analytically pure sample of the *anti* diastereoisomer was obtained by further column chromatography (petrol 40-60/Et_2O 70:30).

^{1}H NMR (400 MHz, $CDCl_3$): δ_H 9.03 (d, J = 8.5 Hz, 1H, H_{10}/H_7), 7.81 – 7.70 (m, 2H, H_{Ar}), 7.57 – 7.45 (m, 4H, H_{Ar}), 7.31 (d, J = 7.8 Hz, 2H, H_{14}), 7.29 – 7.19 (m, 3H, H_{Ar}), 7.09 (t, J = 7.5 Hz, 1H, H_{16}), 6.94 (t, J = 7.6 Hz, 2H, H_{15}), 6.88 (d, J = 8.4 Hz, 1H, H_4), 5.79 (s, 1H, NH), 5.08 (s, 1H, H_{17}), 1.63 (s, 3H, H_3), 1.40 (s, 9H, H_{24}).

^{13}C NMR (101 MHz, $CDCl_3$): δ_C 171.7, 167.4, 136.2, 136.2, 135.8, 135.2, 133.2, 131.4, 131.0, 130.0, 129.0, 128.8, 128.7, 128.3, 128.0, 127.7, 127.4, 125.7, 124.2, 70.8, 51.8, 28.9, 18.4. N.B. Peak at 128.3 ppm corresponds to two overlapping signals.

FTIR (cm^{-1}): 3318, 3059, 2969, 1682, 1644, 1511, 1365, 729.

Melting Point (°C): 191-194.

HRMS (ESI): Exact mass calculated for $C_{30}H_{31}N_2O_2^+$ [M+H]$^+$: 451.2380, found 451.2377. Δ = - 0.8 ppm.

Attempted synthesis of N-(2-(*tert*-butylamino)-2-oxo-1-phenylethyl)-N-(2-isopropyl-4-methylpyridin-3-yl)benzamide, *anti*-1au

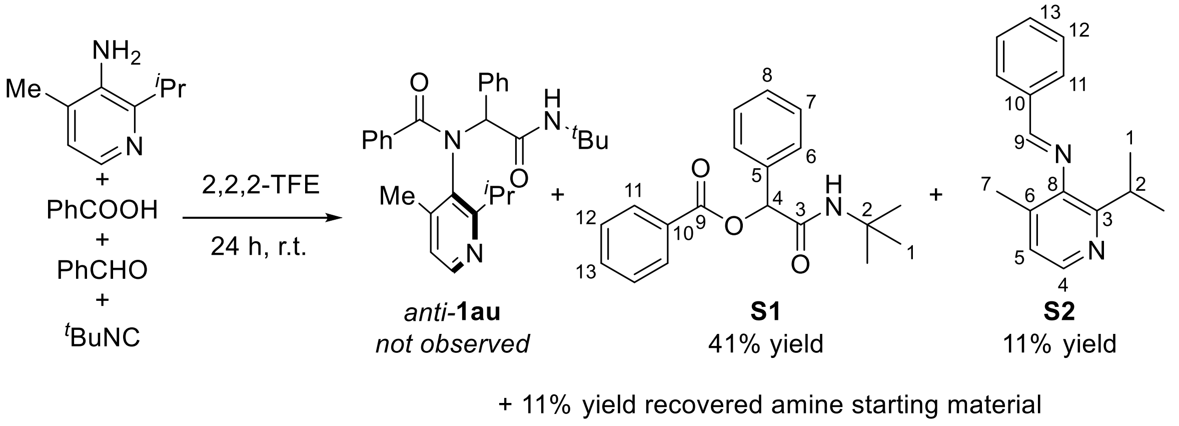

2-isopropyl-4-methylpyridin-3-amine (45 mg, 0.30 mmol, 1.0 eq.), benzoic acid (37 mg, 0.30 mmol, 1.0 eq.), benzaldehyde (31 µL, 0.30 mmol, 1.0 eq.), 2,2,2-trifluoroethanol (0.6 mL, 0.5 M) and *tert*-butyl isocyanide (34 µL, 0.30 mmol, 1.0 eq.) were subjected to **General Procedure A**. Purification *via* column chromatography eluting with CH_2Cl_2/MeOH (90:10) afforded **S1** as a white solid (38 mg, 41% yield)**,** **S2** as a yellow oil (8 mg, 11% yield) and recovered 2-isopropyl-4-methylpyridin-3-amine (5 mg, 11% yield).

Data for *rac*-2-(tert-Butylamino)-2-oxo-1-phenylethyl benzoate, S1

^{1}H NMR (400 MHz, $CDCl_3$): δ_H 8.09 (d, J = 7.2 Hz, 2H, H_{11}), 7.61 (t, J = 7.4 Hz, 1H, H_{13}), 7.55 – 7.49 (m, 2H, H_{Ar}), 7.48 (t, J = 7.7 Hz, 2H, H_{Ar}), 7.43 – 7.35 (m, 3H, H_{Ar}), 6.22 (s, 1H, H_4), 6.00 (s, 1H, NH), 1.37 (s, 9H, H_1).

^{13}C NMR (101 MHz, $CDCl_3$): δ_C 167.5, 165.0, 136.1, 133.7, 129.9, 129.5, 129.0, 128.9, 128.8, 127.6, 76.2, 51.7, 28.8.

The spectral data matched that reported in the literature.[2]

Data for N-(2-isopropyl-4-methylpyridin-3-yl)-1-phenylmethanimine, S2

^{1}H NMR (400 MHz, $CDCl_3$): δ_H 8.25 (d, J = 4.9 Hz, 1H, H_4), 8.23 (s, 1H, H_9), 7.94 – 7.89 (m, 2H, H_{11}), 7.58 – 7.48 (m, 3H, H_{12} and H_{13}), 6.99 (d, J = 4.9 Hz, 1H, H_5), 3.15 (sept, J = 6.8 Hz, 1H, H_2), 2.14 (s, 3H, H_7), 1.23 (d, J = 6.8 Hz, 6H, H_1).

^{13}C NMR (101 MHz, $CDCl_3$): δ_C 163.6, 156.2, 146.1, 144.7, 135.8, 135.7, 132.0, 129.0, 128.8, 123.2, 30.2, 21.9, 18.1.

FTIR (cm^{-1}): 3049, 2963, 2927, 1638, 1580, 1183.

HRMS (ESI): Exact mass calculated for $C_{16}H_{19}N_2^+$ $[M+H]^+$: 239.1543, found 239.1541. Δ = - 0.8 ppm.

Attempted synthesis of N-(2-(*tert*-butylamino)-2-oxo-1-phenylethyl)-N-(2-isopropyl-4-methylpyridin-3-yl)benzamide, *anti*-1av

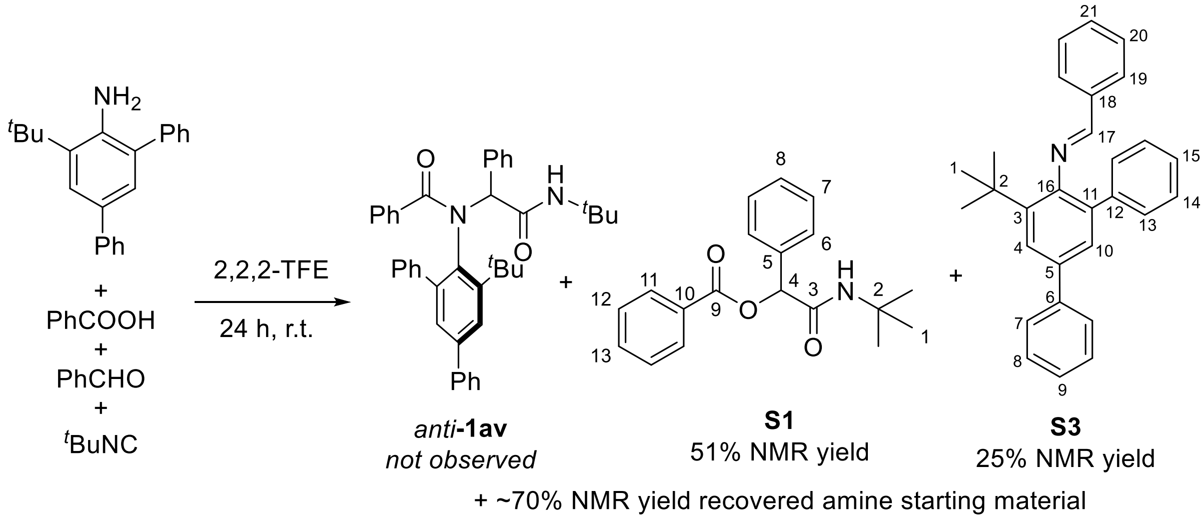

5'-(*tert*-butyl)-[1,1':3',1''-terphenyl]-4'-amine (90 mg, 0.30 mmol, 1.0 eq.), benzoic acid (37 mg, 0.30 mmol, 1.0 eq.), benzaldehyde (31 µL, 0.3 mmol, 1.0 eq.), 2,2,2-trifluoroethanol (0.6 mL, 0.5 M) and *tert*-butyl isocyanide (34 µL, 0.30 mmol, 1.0 eq.) were subjected to **General Procedure A**. In this case, the crude residue obtained upon removal of 2,2,2-trifluoroethanol was dissolved in $CDCl_3$ (~2 mL) and 1,1,2,2-tetrachloroethane (16 µL, 0.15 mmol) was added. The resulting solution was analysed by quantitative ^{1}H NMR (400 MHz, relaxation delay = 25 s) and was determined to contain **S1** (51% NMR yield), **S3** (25% NMR yield) and unreacted 5'-(*tert*-butyl)-[1,1':3',1''-terphenyl]-4'-amine (~70% NMR yield).

The spectral data for **S1** was identical to that described above. The spectral data for **S3** was identical to an authentic sample prepared as described below.

N-(5'-(*tert*-butyl)-[1,1':3',1''-terphenyl]-4'-yl)-1-phenylmethanimine, S3

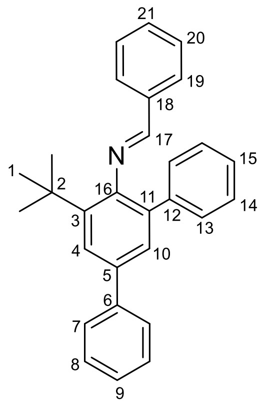

5'-(*tert*-butyl)-[1,1':3',1''-terphenyl]-4'-amine (1.51 g, 5.00 mmol, 1.0 eq.) was dissolved in ethanol (20 mL) under nitrogen. Benzaldehyde (0.51 mL, 5.0 mmol, 1.0 eq.) was added and the resulting solution was refluxed for 17 hours. The resulting mixture was cooled to room temperature, concentrated and then purified by column chromatography (petrol/Et_2O 98:2) to afford **S3** as a brown solid (760 mg, 39%).

^{1}H NMR (400 MHz, $CDCl_3$): δ_H 7.86 (s, 1H, H_{17}), 7.68 – 7.64 (m, 3H, H_4 and H_{Ar}), 7.60 (dt, *J* = 8.1, 1.6 Hz, 2H, H_{Ar}), 7.52 (d, *J* = 2.0 Hz, 1H, H_{10}), 7.47 – 7.30 (m, 8H, H_{Ar}), 7.27 – 7.20 (m, 2H, H_{Ar}), 7.16 – 7.09 (m, 1H, H_{Ar}), 1.50 (s, 9H, H_1).

^{13}C NMR (101 MHz, $CDCl_3$): δ_C 164.0, 149.6, 142.4, 141.5, 140.9, 136.8, 136.5, 133.5, 131.2, 130.7, 128.9, 128.7, 128.5, 128.2, 128.1, 127.2, 127.1, 126.2, 124.7, 36.2, 30.7.

FTIR (cm $^{-1}$): 3059, 2956, 2870, 1634, 1601, 1579, 1423, 907, 885, 762, 751, 731, 691.

Melting Point (°C): 119-120.

HRMS (ESI): Exact mass calculated for $C_{29}H_{28}N$ $[M+H]^+$: 390.2216, found: 390.2199. Δ = 4.4 ppm.

***rac*-(R_a,*R*)- N-(2-(*tert*-butyl)phenyl)-N-(2-(cyclohexylamino)-2-oxo-1-phenylethyl)-2,4-dimethylthiazole-5-carboxamide, *anti*-1aw**

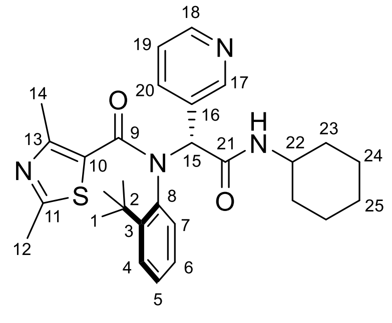

2-*tert*-Butylaniline (180 mg, 1.20 mmol, 1.0 eq.), 2,4-dimethyl-1,3-thiazole-5-carboxylic acid (189 mg, 1.20 mmol, 1.0 eq.), 3-pyridinecarboxaldehyde (113 µL 1.20 mmol, 1.0 eq.), 2,2,2-trifluoroethanol (2.4 ml, 0.5 M) and cyclohexyl isocyanide (149 µL, 1.20 mmol, 1.0 eq.) were subjected to **General Procedure A**. Purification *via* column chromatography eluting with petrol 40-60/EtOAc 20:80 afforded the title compound *anti*-**1aw** as a white solid (239 mg, 39% yield, >95:5 d.r.). An analytical quantity of *anti*-**1aw** was further purified by recrystallization from boiling petrol 40-60/EtOAc which was used for biological testing.

^{1}H NMR (400 MHz, CDCl$_3$): δ_H 8.63 (d, *J* = 2.3 Hz, 1H, H_{17}), 8.60 (dd, *J* = 4.8, 1.6 Hz, 1H, H_{18}), 8.21 (dt, *J* = 8.1, 2.0 Hz, 1H, H_{20}), 7.65 (dd, *J* = 7.9, 1.6 Hz, 1H, H_7), 7.43 (dd, *J* = 8.2, 1.6 Hz, 1H, H_4), 7.37 – 7.29 (m, 2H, H_5 and H_{19}), 7.21 (td, *J* = 7.5, 1.6 Hz, 1H, H_6), 5.81 (d, *J* = 8.0 Hz, 1H, NH), 5.35 (s, 1H, H_{15}), 3.86 – 3.74 (m, 1H, H_{22}), 2.64 (s, 3H, H_{14}), 2.40 (s, 3H, H_{12}), 1.98 – 1.77 (m, 2H, H_{Cy}), 1.69 – 1.50 (m, 3H, H_{Cy}), 1.39 – 1.22 (m, 2H, H_{Cy}), 1.05 (s, 12H, H_1 and H_{Cy}).

^{13}C NMR (101 MHz, CDCl$_3$): δ_C 167.8, 167.2, 165.5, 160.5, 151.8, 150.3, 148.2, 140.3, 138.8, 133.2, 132.0, 130.6, 129.6, 127.7, 123.8, 123.3, 71.0, 49.1, 36.3, 32.9, 32.7, 32.2, 25.5, 24.8, 24.8, 18.7, 18.5.

FTIR (cm^{-1}): 2931, 1678, 1627, 1512, 1428, 1312, 909, 727.

Melting Point (°C): 153 – 155.

HRMS (ESI): Exact mass calculated for $C_{29}H_{37}N_4O_2S^+$ [M+H]$^+$: 505.2632, found: 505.2630. Δ = – 0.4 ppm.

3.2. Mechanistic experiments and configurational stability studies

*(a) Thermal atropisomerization of anti-**1a** in different solvents:*

A solution of *anti*-**1a** (20 mg, 0.045 mmol) was dissolved in the appropriate solvent (0.5 mL) and the resulting solution was heated (in either a microwave vial or Ace pressure tube as appropriate) to 110 °C for 48 h. The resulting solution was cooled to room temperature and concentrated to dryness. The residue was dissolved in $CDCl_3$ (0.6 mL) and the resulting solution was analysed by ^{1}H NMR spectroscopy to determine the d.r. of *syn*-**1a**.

*(b) Monitoring epimerization of **1a** by ^{1}H-NMR spectroscopy*

*Epimerization of anti-**1a**:* an NMR sample of *anti*-**1a** (>95:5 d.r.; ~10 mg) in 0.5 mL of d_6-DMSO was loaded into a 500 MHz NMR spectrometer which had been preheated to 110 °C. ^{1}H NMR spectra were recorded at regular intervals over the course of 2.74 hours. In each spectrum, diagnostic signals for *anti*-**1a** (δ_H = 6.07 ppm) and *syn*-**1a** (δ_H = 6.01 ppm) were integrated to obtain the diastereomeric ratios. The resulting data were as follows:

time / hours	fraction *anti*-**1a**	fraction *syn*-**1a**	$\ln([anti]_t - [anti]_{eq})$
0.000	0.9952	0.0048	-0.0960
0.073	0.8288	0.1712	-0.2983
0.149	0.6333	0.3667	-0.6040
0.232	0.4991	0.5009	-0.8858
0.315	0.4152	0.5848	-1.1132
0.399	0.3372	0.6628	-1.3843
0.482	0.2848	0.7152	-1.6190
0.567	0.2486	0.7514	-1.8208
0.649	0.2151	0.7849	-2.0526
0.757	0.1848	0.8152	-2.3218
0.826	0.1693	0.8307	-2.4937
0.907	0.1515	0.8485	-2.7364
0.993	0.1371	0.8629	-2.9878
1.073	0.1333	0.8667	-3.0662
1.159	0.1261	0.8739	-3.2340
1.240	0.1178	0.8822	-3.4705
1.329	0.1098	0.8902	-3.7679
1.412	0.1039	0.8961	-4.0628
1.493	0.1006	0.8994	–
1.581	0.1009	0.8991	–
1.659	0.0947	0.9053	–
1.745	0.0901	0.9099	–

1.832	0.0869	0.9131	–
1.909	0.0867	0.9133	–
1.996	0.0905	0.9095	–
2.079	0.0872	0.9128	–
2.156	0.0855	0.9145	–
2.243	0.0872	0.9128	–
2.329	0.0856	0.9144	–
2.412	0.0853	0.9147	–
2.493	0.0850	0.9150	–
2.573	0.0843	0.9157	–
2.657	0.0861	0.9139	–
2.740	0.0867 = [*anti*-**1a**]$_{eq}$	0.9133	–

This data was used to construct the time course plot shown in Scheme 4B of the manuscript.

The associated kinetic parameters were calculated by plotting the deviation from equilibrium. i.e., ln([*anti*-**1a**]$_t$ – [*anti*-**1a**]$_{eq}$) against time to give a straight line with a gradient = –(k_f + k_b), where [*anti*-**1a**]$_t$ and [*anti*-**1a**]$_{eq}$ are the respective molar fractions of *anti*-**1a** at time *t* and at equilibrium and k_f and k_b are the forward and backward rate constants respectively. This analysis generated the following straight-line graph:

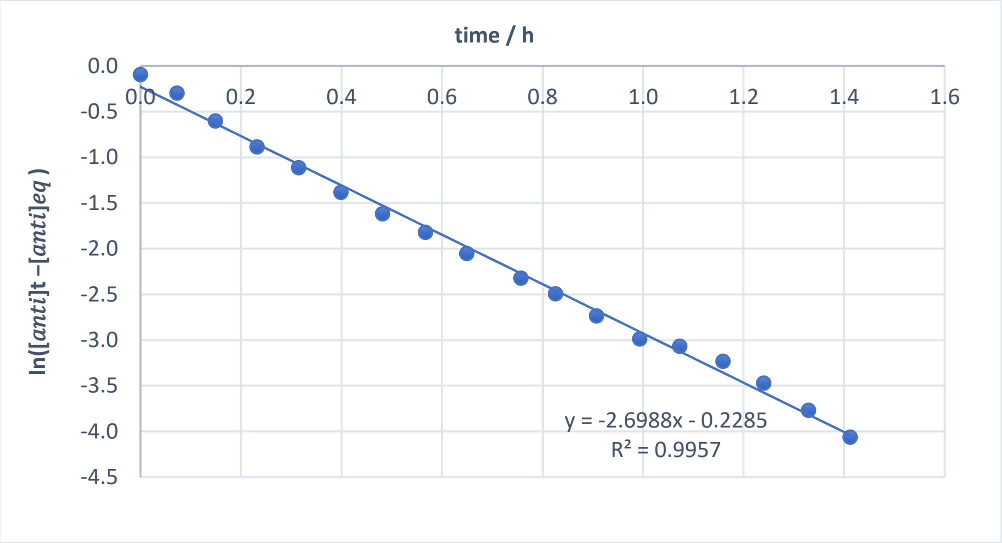

Therefore, (k_f + k_b) = 2.70 h^{-1}

Given the equilibrium ratio of *syn*/*anti*-**1a** of 0.9133:0.0867 measured after 2.74 hours, we calculated that:

$$K_{eq} = \frac{0.9133}{0.0867} = 10.53$$

And because $K_{eq} = \frac{k_f}{k_b}$

k_f = 2.46 h^{-1} and k_b = 0.23 h^{-1} (at 110 °C)

These values were substituted into the Eyring equation to calculate the associated values of $\Delta G^{\ddagger}$ (at 110 °C), which were as follows:

$\Delta G^{\ddagger}_f$ = 28.2 kcal/mol and $\Delta G^{\ddagger}_b$ = 30.0 kcal/mol

<u>*Epimerization of syn-***1a***:*</u> an NMR sample of *syn*-**1a** (>95:5 d.r.; ~10 mg) in 0.5 mL of d_6-DMSO was loaded into a 500 MHz NMR spectrometer which had been preheated to 110 °C. ^{1}H NMR spectra were recorded at regular intervals over the course of 1.82 hours. In each spectrum, diagnostic signals for *anti*-**1a** (δ_H = 6.07 ppm) and *syn*-**1a** (δ_H = 6.01 ppm) were integrated to obtain the diastereomeric ratios. The resulting data were as follows:

time / hours	fraction *syn*-**1a**	fraction *anti*-**1a**
0.000	0.9778	0.0222
0.064	0.9660	0.0340
0.123	0.9566	0.0434
0.211	0.9495	0.0505
0.314	0.9429	0.0571
0.414	0.9334	0.0666
0.519	0.9324	0.0676
0.614	0.9287	0.0713
0.716	0.9235	0.0765
0.819	0.9205	0.0795
0.914	0.9201	0.0799
1.014	0.9197	0.0803
1.111	0.9172	0.0828
1.217	0.9182	0.0818
1.319	0.9150	0.0850
1.417	0.9157	0.0843
1.511	0.9142	0.0858
1.616	0.9148	0.0852
1.715	0.9115	0.0885
1.763	0.9123	0.0877
1.820	0.9100	0.0900

This data was used to construct the time course plot shown in Scheme 4B of the manuscript.

(c) Monitoring epimerization of **1z** by HPLC analysis

A solution of *anti*-**1z** (>95:5 d.r.; 50 mg) in 0.5 mL of DMSO was prepared at room temperature in a microwave vial. The vial was stoppered with a rubber subaseal, and the tube was immersed in an oil bath preheated to 110 °C. At each timepoint specified below, a 5 µL aliquot was removed, placed in a HPLC vial, and concentrated to dryness under a stream of nitrogen. The residue was dissolved in HPLC grade hexane and analysed by HPLC (Chiralpak-IC column. Solvent ratio = 90:10 *n*-hexane: ^{i}PrOH. Temperature = 25 °C. Flow rate = 1 ml/min, λ= 210 nm, τ_{ret} 4.3 min (*syn*), 4.5-4.7 min (*anti* + *syn*); 5.5 min (*anti*). HLC traces for pure *anti*-**1z** and *syn*-**1z** and an equimolar mixture of *syn*/*anti*-**1z** are shown below.

Data for *anti*-**1z**

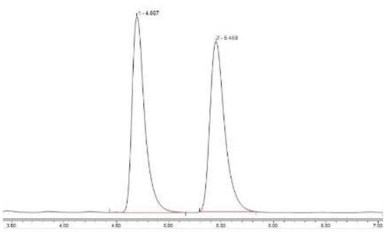

Ret.Time min	Amount n.a.	Rel.Area %	Area mAU*min	Height mAU
4.697	n.a.	49.64	322.0654	2301.05
5.450	n.a.	50.36	326.7198	2003.16

Data for *syn*-**1z**

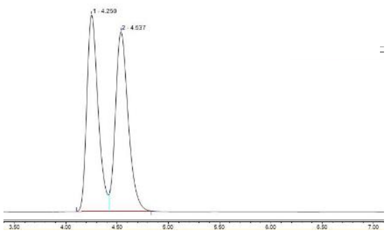

Ret.Time min	Amount n.a.	Rel.Area %	Area mAU*min	Height mAU
4.250	n.a.	49.53	228.0996	1779.54
4.537	n.a.	50.47	232.4276	1624.65

Mixture of *anti*/*syn*-**1z**

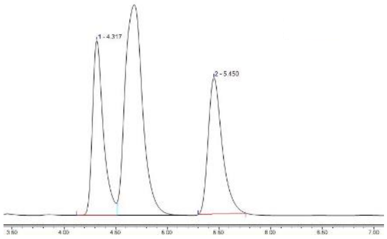

Ret.Time min	Amount n.a.	Rel.Area %	Area mAU*min	Height mAU
4.317	n.a.	49.80	240.6929	1974.26
5.450	n.a.	50.20	242.6605	1540.71

The resulting data (obtained *via* integration of the peaks at 4.3 and 5.5 min) were as follows:

time / hours	fraction *anti*-**1z**	fraction *syn*-**1z**	$\ln([anti]_t - [anti]_{eq})$
0.00	1.0000	0.0000	-0.0961
5.25	0.8666	0.1334	-0.2549
23.25	0.5448	0.4552	-0.7914
29.25	0.4653	0.5347	-0.9843
47.25	0.2849	0.7151	-1.6435
53.25	0.2611	0.7389	-1.7749
71.25	0.1801	0.8199	-2.4248
78.00	0.1672	0.8328	-2.5823
96.00	0.1324	0.8676	-3.1991
102.50	0.1221	0.8779	-3.4900
168.00	0.0918	0.9082	–
173.00	0.0916 = [*anti*-**1z**]$_{eq}$	0.9084	–

The associated kinetic parameters were calculated by plotting the deviation from equilibrium. i.e., ln([*anti*-**1z**]$_t$ – [*anti*-**1z**]$_{eq}$) against time to give a straight line with a gradient = $-(k_f + k_b)$, where [*anti*-**1z**]$_t$

and [*anti*-**1z**]$_{eq}$ are the respective molar fractions of *anti*-**1z** at time *t* and at equilibrium and k_f and k_b are the forward and backward rate constants respectively. This analysis generated the following straight-line graph:

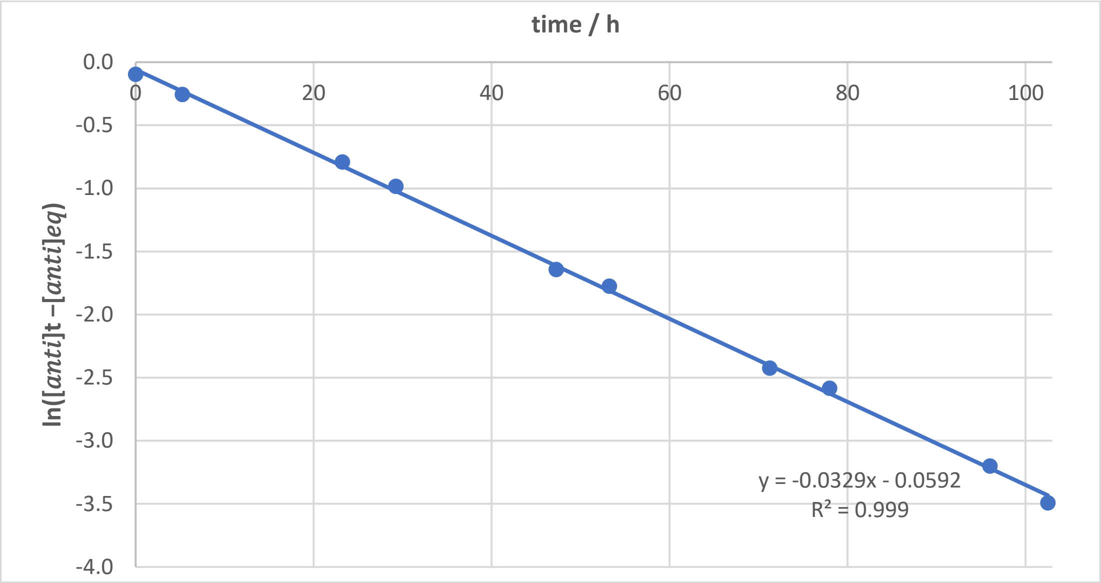

Therefore, $(k_f + k_b) = 0.0329\ h^{-1}$

Given the equilibrium ratio of *syn*/*anti*-**1z** of 0.9084:0.0916 measured after 173 hours, we calculated that:

$$K_{eq} = \frac{0.9084}{0.0916} = 9.92$$

And because $K_{eq} = \frac{k_f}{k_b}$

$k_f = 0.030\ h^{-1}$ and $k_b = 0.0030\ h^{-1}$ (at 110 °C)

These values were substituted into the Eyring equation to calculate the associated values of $\Delta G^{\ddagger}$ (at 110 °C), which were as follows:

$\Delta G^{\ddagger}{}_f = 31.5$ kcal/mol and $\Delta G^{\ddagger}{}_b = 33.3$ kcal/mol

(d) Deuterium labelling experiment to probe the nature of thermal epimerization

An Ace pressure tube (capacity ~15 mL, L × O.D. 10.2 cm × 25.4 mm) equipped with a stirrer bar, was sequentially charged with *anti*-**1a** (40 mg, 0.090 mmol) and d_3-trifluoroethanol (1.0 mL). The tube was sealed, and the resulting mixture was heated to 110 °C in a preheated oil bath for 48 h. The resulting solution was cooled to room temperature and concentrated to dryness. Purification *via* column chromatography eluting with petrol 40-60/EtOAc 80:20 afforded **1a** as a pale-yellow solid (35 mg, 88% yield, 91:9 d.r. *syn*/*anti*).

^{1}H NMR (400 MHz, CDCl$_3$): δ_H 8.07 (d, J = 7.7 Hz, 1H, H_7), 7.33 (d, J = 7.8 Hz, 2H, H_{11}), 7.28 – 7.02 (m, 11H, H_{16}, H_4, H_5, H_6, H_{12}, H_{13}, H_{17} and H_{18}), 6.05 (s, **1.00H**, H_{14}), 5.89 (s, **0.77H**, N-H), 1.37 (s, 9H, H_{21}), 0.84 (s, 9H, H_1). The peaks for H_{14} and the NH integrated to 1.00 and 0.77 respectively, implying <5% and 23% deuteration at these positions respectively.

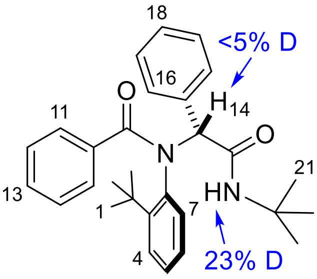

syn-**1a**
quant. ^{1}H NMR
400 MHz, CDCl$_3$

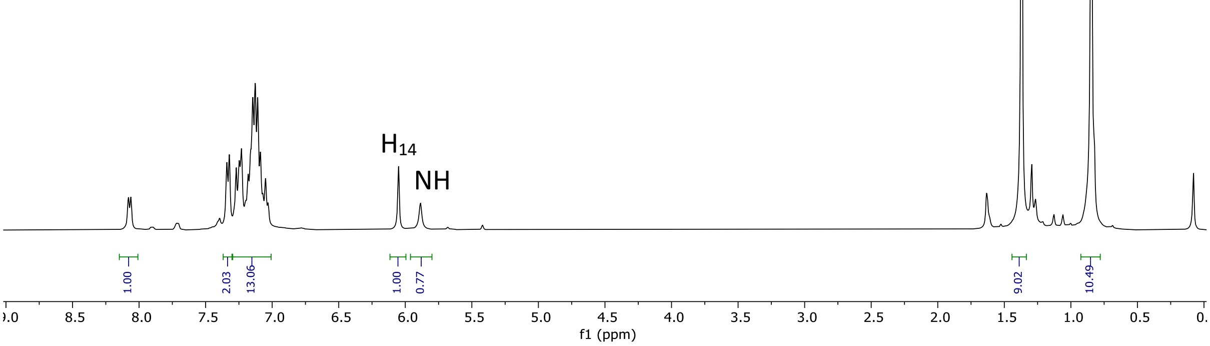

3.3. Synthesis of syn-configured peptide analogues

***rac*-(*S_a*,*R*)-N-(2-(*tert*-Butyl)phenyl)-N-(2-(*tert*-butylamino)-2-oxo-1-phenylethyl)benzamide, *syn*-1a**

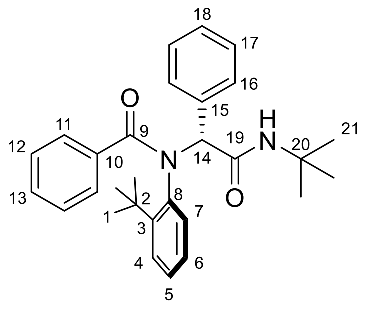

2-*tert*-Butylaniline (37 mg, 0.25 mmol, 1.0 eq.), benzoic acid (31 mg, 0.25 mmol, 1.0 eq.), benzaldehyde (25 µL, 0.25 mmol, 1.0 eq.), 2,2,2-trifluoroethanol (0.5 mL, 0.5 M) and *tert*-butyl isocyanide (28 µL, 0.25 mmol, 1.0 eq.) were subjected to **General Procedure B**. Purification via column chromatography eluting with petrol 40-60/Et_2O 70:30 afforded the title compound *syn*-**1a** as a white solid (105 mg, 95% yield, 92:8 d.r.). An analytically pure sample of the *syn*-diastereoisomer was obtained by further column chromatography (petrol 40-60/Et_2O 80:20).

^{1}H NMR (300 MHz, $CDCl_3$): δ_H 8.09 – 8.05 (m, 1H, H_7), 7.35 – 7.30 (m, 2H, H_{11}), 7.26 – 7.21 (m, 2H, H_{16}), 7.20 – 7.00 (m, 9H, H_4, H_5, H_6, H_{12}, H_{13}, H_{17} and H_{18}), 6.05 (s, 1H, H_{14}), 5.89 (s, 1H, NH), 1.36 (s, 9H, H_{21}), 0.84 (s, 9H, H_1).

^{13}C NMR (75 MHz, $CDCl_3$): δ 170.6, 169.6, 147.3, 136.6, 135.8, 134.7, 133.2, 132.0, 130.6, 129.5, 128.6, 128.4, 128.0, 127.4, 125.5, 67.9, 51.7, 36.1, 32.2, 28.8, N.B. The peak at 129.5 ppm corresponds to two overlapping signals.

FTIR (cm^{-1}): 3351, 3063, 2972, 1683, 1614, 1539, 1364, 730, 695, 561.

Melting Point (°C): 141 – 142.

HRMS (ESI): Exact mass calculated for $C_{29}H_{35}N_2O_2^+$ [M+H]$^+$: 443.2693, found: 443.2704. Δ = 2.5 ppm.

***rac*-(S_a,*R*)- N-(2-(*tert*-butyl)phenyl)-N-(2-(*tert*-butylamino)-2-oxo-1-(*m*-tolyl)ethyl)benzamide, *syn*-1f**

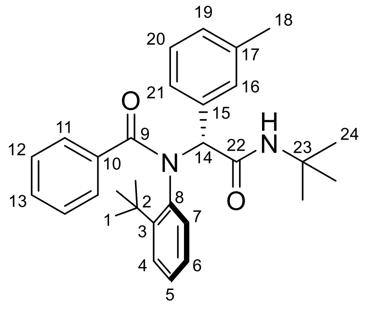

2-*tert*-Butylaniline (37 mg, 0.25 mmol, 1.0 eq.), benzoic acid (28 mg, 0.25 mmol, 1.0 eq.), 3-methylbenzaldehyde (30 µL, 0.25 mmol, 1.0 eq.), 2,2,2-trifluoroethanol (0.5 mL, 0.5 M) and *tert*-butyl isocyanide (28 µL, 0.25 mmol, 1.0 eq.) were subjected to **General Procedure B**. Purification via column chromatography eluting with petrol 40-60/ Et_2O 75:25, afforded the title compound *syn*-**1f** as a white solid (93 mg, 81% yield, 92:8 d.r.). An analytically pure sample of the *syn*-diastereoisomer was obtained by further column chromatography (petrol 40-60/Et_2O 70:30).

^{1}H NMR ($CDCl_3$, 400 MHz): δ_H 8.05 (dd, *J* = 7.7, 1.8 Hz, 1H, H_7), 7.35 – 7.30 (m, 2H, H_{11}), 7.19 – 7.14 (m, 1H, H_{20}), 7.13 – 6.99 (m, 8H, H_4, H_5, H_6, H_{12}, H_{13}, H_{16}, H_{19}), 6.96 – 6.93 (m, 1H, H_{21}), 5.98 (s, 1H, H_{14}), 5.85 (s, 1H, NH), 2.15 (s, 3H, H_{18}), 1.36 (s, 9H, H_{24}), 0.85 (s, 9H, H_1).

^{13}C NMR (101 MHz, $CDCl_3$): δ_C 170.5, 169.7, 147.3, 137.5, 136.7, 135.9, 134.8, 133.1, 132.8, 130.5, 129.6, 129.5, 129.3, 129.0, 128.3, 127.9, 127.4, 125.4, 67.9, 51.7, 36.1, 32.2, 28.8, 21.3.

IR (cm^{-1}): 3340, 2972, 2905, 1679, 1623, 1362, 1242.

Melting point (°C): 149 – 151.

HRMS (ESI): Exact mass calculated for $C_{30}H_{37}N_2O_2^+$ $[M+H]^+$ 457.2850, found 457.2854 Δ = 0.9 ppm.

***rac*-(S_a,*R*)-N-(2-(*tert*-Butyl)phenyl)-N-(2-(*tert*-butylamino)-2-oxo-1-(3-(trifluoromethoxy)phenyl) ethyl)benzamide, *syn*-1h**

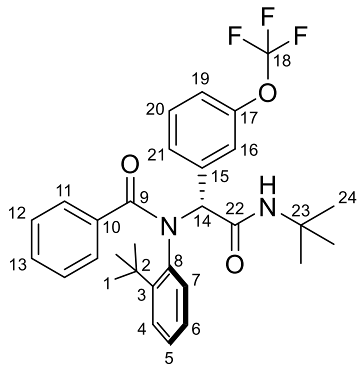

2-*tert*-Butylaniline (37 mg, 0.25 mmol, 1.0 eq.), benzoic acid (31 mg, 0.25 mmol, 1.0 eq.), 3-trifluoromethoxybenzaldehyde (36 µL, 0.25 mmol, 1.0 eq.), 2,2,2-trifluoroethanol (0.5 mL, 0.5 M) and *tert*-butyl isocyanide (28 µL, 0.25 mmol, 1.0 eq.) were subjected to **General Procedure B**. Purification via column chromatography eluting with petrol 40-60/Et_2O 70:30, afforded the title compound *syn*-**1h** as a white solid (119 mg, 90% yield, 93:7 d.r.). An analytically pure sample of the *syn*-diastereoisomer was obtained by further column chromatography (petrol 40-60/Et_2O 70:30).

^{1}H NMR (400 MHz, $CDCl_3$): δ_H 7.97 (dd, *J* = 7.5, 1.8 Hz, 1H, H_7), 7.30 (d, *J* = 7.7 Hz, 2H, H_{11}), 7.23 – 7.00 (m, 10H, H_{Ar}), 6.15 (br s, 1H, NH), 6.09 (s, 1H, H_{14}), 1.37 (s, 9H, H_{24}), 0.82 (s, 9H, H_1).

^{13}C NMR (101 MHz, $CDCl_3$): δ_C 170.7, 169.0, 148.8, 147.3, 136.3, 135.5, 135.4, 134.2, 130.9, 130.6, 129.7, 129.5, 129.2, 128.8, 127.5, 125.9, 124.9, 121.4, 120.4 (q, *J* = 257.4 Hz), 67.0, 51.9, 36.1, 32.1, 28.8.

^{19}F{^{1}H} NMR (376 MHz, $CDCl_3$): δ_F –57.8.

FTIR (cm^{-1}): 3344, 2971, 2919, 1685, 1623, 1250, 1163.

Melting point (°C): 154 – 157.

HRMS (ESI): Exact mass calculated for $C_{30}H_{33}F_3N_2O_3Na^+$ $[M+Na]^+$ 549.2335, found 549.2344. Δ = 1.6 ppm.

***rac*-(S_a,*R*)- N-(2-(*tert*-Butyl)phenyl)-N-(2-(*tert*-butylamino)-2-oxo-1-phenylethyl)nicotinamide, *syn*-1x**

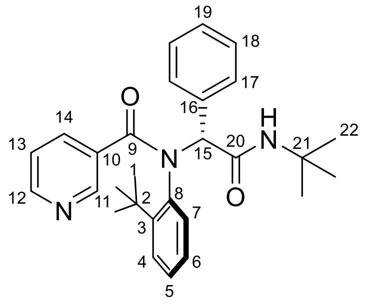

2-*tert*-Butylaniline (180 mg, 1.20 mmol, 1.0 eq.), nicotinic acid (148 mg, 1.20 mmol, 1.0 eq.), benzaldehyde (127 mg, 1.20 mmol, 1.0 eq.), 2,2,2-trifluoroethanol (2.4 mL, 0.5 M) and *tert*-butyl isocyanide (136 µL, 1.20 mmol, 1.0 eq.) were subjected to **General Procedure B**. Purification via column chromatography eluting with petrol 40-60/EtOAc 40:60, afforded the title compound *syn*-**1x** as a white solid (389 mg, 73% yield, 90:10 d.r.). An analytically pure sample of the *syn*-diastereoisomer was obtained by further column chromatography (petrol 40-60/EtOAc 40:60).

^{1}H NMR (400 MHz, $CDCl_3$): δ_H 8.58 (d, *J* = 2.3 Hz, 1H, H_{11}), 8.40 (dd, J = 4.9, 1.7 Hz, 1H, H_{12}), 8.12 (dd, *J* = 7.6, 1.9 Hz, 1H, H_7), 7.57 (dt, *J* = 8.0, 2.0 Hz, 1H, H_{14}), 7.24 – 7.20 (m, 2H, H_{17}), 7.17 – 7.09 (m, 5H, H_5, H_6, H_{18} and H_{19}), 7.09 – 7.01 (m, 2H, H_4 and H_{13}), 6.00 (s, 1H, H_{15}), 5.65 (s, 1H, NH), 1.34 (s, 9H, H_{22}), 0.85 (s, 9H, H_1).

^{13}C NMR (101 MHz, $CDCl_3$): δ_C 169.3, 168.2, 150.3, 150.0, 147.3, 136.9, 135.2, 134.6, 132.9, 132.7, 132.0, 130.8, 129.0, 128.9, 128.2, 125.9, 122.3, 68.1, 51.9, 36.1, 32.3, 28.8.

FTIR (cm^{-1}): 3245, 2981, 1682, 1640, 1563, 1380, 1155, 1095, 954.

Melting point (°C): 172 – 173.

HRMS (ESI): Exact mass calculated for $C_{28}H_{34}N_3O_2^+$ $[M+H]^+$: 444.2646, found 444.2650. Δ = 0.9 ppm.

***rac*-(S_a,*R*)- N-(2-(*tert*-Butyl)phenyl)-N-(2-(*tert*-butylamino)-2-oxo-1-phenylethyl)furan-2-carboxamide, *syn*-1y**

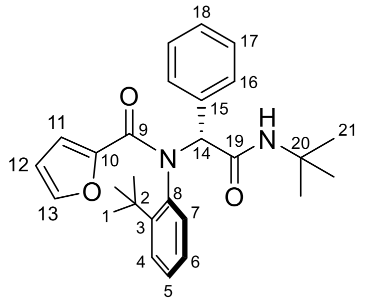

2-*tert*-Butylaniline (180 mg, 1.20 mmol, 1.0 eq.), 2-furoic acid (135 mg, 1.20 mmol, 1.0 eq.), benzaldehyde (122 µL, 1.20 mmol, 1.0 eq.), 2,2,2-trifluoroethanol (2.4 mL, 0.5 M) and *tert*-butyl isocyanide (136 µL, 1.20 mmol, 1.0 eq.) were subjected to **General Procedure B**. Purification via column chromatography eluting with petrol 40-60/Et_2O 70:30, afforded the title compound *syn*-**1y** as a white solid (436 mg, 84% yield, 89:11 d.r.). An analytically pure sample of the *syn*-diastereoisomer was obtained by further column chromatography (petrol 40-60/Et_2O 70:30).

^{1}H NMR (400 MHz, $CDCl_3$): δ_H 7.90 (d, *J* = 7.2 Hz, 1H, H_7), 7.39 (d, *J* = 1.9 Hz, 1H, H_{11}), 7.30 – 7.25 (m, 2H, H_4 and H_5), 7.24 – 7.08 (m, 6H, H_6, H_{16}, H_{17}, H_{18}), 6.13 (dd, *J* = 3.6, 1.8 Hz, 1H, H_{12}), 5.94 (br s, 1H, NH), 5.91 (s, 1H, H_{14}), 5.20 (d, *J* = 3.6 Hz, 1H, H_{13}), 1.32 (s, 9H, H_{21}), 0.91 (s, 9H, H_1).

^{13}C NMR (101 MHz, $CDCl_3$): δ_C 169.2, 160.2, 148.2, 147.3, 144.8, 136.0, 134.0, 133.0, 132.0, 130.6, 129.2, 128.7, 128.1, 126.6, 117.0, 111.1, 67.9, 51.6, 36.4, 32.3, 28.8.

FTIR (cm^{-1}): 3351, 2969, 1694, 1619, 1029, 760.

Melting point (°C): 173 – 175.

HRMS (ESI): Exact mass calculated for $C_{27}H_{32}N_2O_3Na^+$ $[M+Na]^+$ 455.2305, found 455.2301 Δ = -0.9 ppm.

***rac*-(S_a,*R*)- N-(2-(*tert*-butyl)phenyl)-N-(2-(*tert*-butylamino)-2-oxo-1-phenylethyl)-3-phenylpropanamide, *syn*-1z**

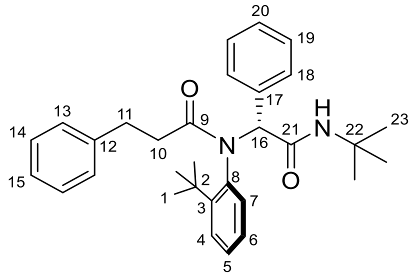

According to a modification of **General Procedure B**. To an oven-dried Schlenk tube, equipped with stirrer bar, was sequentially charged 2-*tert*-butylaniline (180 mg, 1.20 mmol, 1.0 eq.), hydrocinnamic acid (180 mg, 1.20 mmol, 1.0 eq.), benzaldehyde (122 µL, 1.20 mmol, 1.0 eq.), 2,2,2-trifluoroethanol (2.4 mL, 0.5 M) and *tert*-butyl isocyanide (136 µL, 1.20 mmol, 1.0 eq). The solution was stirred at room temperature for 16h. Dimethyl sulfoxide (2.4 mL, 0.5 M) was then charged, and the solution was heated to 110 °C for 6 days. After this time, the solution was cooled to room temperature and the reaction mixture was concentrated to dryness. This was followed by the addition of EtOAc and saturated Na_2CO_3 to the crude residue. The layers were separated, and the aqueous layer was extracted twice with EtOAc. The combined organics were washed once with brine, dried over $MgSO_4$, filtered, and concentrated *in vacuo*. The crude residue was purified by column chromatography (petrol 40-60/Et_2O 80:20) to afford *syn*-**1z** as an off-white solid (369 mg, 65% yield, 90:10 d.r.). An analytically pure sample of the *syn*-diastereoisomer was obtained by further column chromatography (petrol 40-60/Et_2O 80:20).

^{1}H NMR (400 MHz, $CDCl_3$): δ_H 7.73 (d, J = 7.2 Hz, 1H, H_7), 7.29 – 6.98 (m, 13H, H_{Ar}) 5.85 (s, 1H, NH), 5.77 (s, 1H, H_{16}), 3.05 – 2.87 (m, 2H, H_{11} and $H_{11'}$), 2.42 (ddd, J = 15.8, 9.6, 6.1 Hz, 1H, H_{10}), 2.32 (ddd, J = 16.2, 9.9, 6.4 Hz, 1H, $H_{10'}$), 1.31 (s, 9H, H_{23}), 0.95 (s, 9H, H_1).

^{13}C NMR (101 MHz, $CDCl_3$): δ_C 173.6, 169.5, 147.3, 141.5, 136.4, 133.6, 133.2, 131.8, 130.6, 128.7, 128.6, 128.6, 128.5, 128.1, 126.7, 126.0, 67.2, 51.5, 38.5, 36.3, 32.4, 31.3, 28.8.

FTIR (cm^{-1}): 3342, 2982, 1684, 1634, 1545, 1489, 1393, 700, 543.

Melting Point (°C): 108 – 109.

HRMS (ESI): Exact mass calculated for $C_{31}H_{39}N_2O_2^+$ [M+H]$^+$: 471.3006, found: 471.3001. Δ = –1.1 ppm.

***rac*-(S_a,*R*)-N-(2-(*tert*-Butyl)phenyl)-N-(2-(cyclohexylamino)-2-oxo-1-phenylethyl)benzamide, *syn*-1ai**

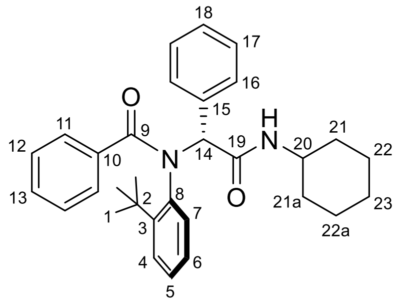

2-*tert*-Butylaniline (180 mg, 1.20 mmol, 1.0 eq.), benzoic acid (147 mg, 1.20 mmol, 1.0 eq.), benzaldehyde (122 µL, 1.20 mmol, 1.0 eq.), 2,2,2-trifluoroethanol (2.4 mL, 0.5 M) and cyclohexyl isocyanide (149 µL, 1.20 mmol, 1.0 eq.) were subjected to **General Procedure B**. Purification via column chromatography eluting with petrol 40-60/Et_2O 70:30, afforded the title compound *syn*-**1ai** as a white solid (423 mg, 75% yield, 94:6 d.r.). An analytically pure sample of the *syn*-diastereoisomer was obtained by further column chromatography (petrol 40-60/Et_2O 65:35).

^{1}H NMR (400 MHz, $CDCl_3$) δ_H 8.05 (dd, *J* = 7.0, 2.2 Hz, 1H, H_7), 7.36 – 7.30 (m, 2H, H_{11}), 7.24 – 7.20 (m, 2H, H_{16}), 7.19 – 7.06 (m, 8H, H_{Ar}), 7.06 – 7.03 (m, 1H, H_4), 6.12 (s, 1H, H_{14}), 5.89 (d, *J* = 8.1 Hz, 1H, NH), 3.91 – 3.77 (m, 1H, H_{20}), 2.02 (m, 1H, H_{Cy}), 1.86 – 1.78 (m, 1H, H_{Cy}), 1.72 – 1.51 (m, 3H, H_{Cy}), 1.39 – 1.28 (m, 2H, H_{Cy}), 1.24 – 0.98 (m, 3H, H_{Cy}), 0.85 (s, 9H, H_1).

^{13}C NMR (101 MHz, $CDCl_3$) δ_C 170.7, 169.6, 147.4, 136.6, 136.0, 134.5, 133.2, 132.0, 130.6, 129.5, 129.5, 128.7, 128.4, 128.1, 127.4, 125.6, 67.7, 49.0, 36.1, 33.0, 32.8, 32.2, 25.6, 24.9, 24.9.

FTIR (cm^{-1}): 3270, 2982, 2934, 1643, 1354, 696.

Melting Point (°C): 158 – 160.

HRMS (ESI): Exact mass calculated for $C_{31}H_{37}N_2O_2^+$ $[M+H]^+$: 469.2850, found 469.2852. Δ = 0.4 ppm.

***rac*-(S_a,*R*)-N-(2-(*tert*-butylamino)-2-oxo-1-phenylethyl)-N-(2-iodo-4,6-dimethylphenyl)benzamide, *syn*-1am**

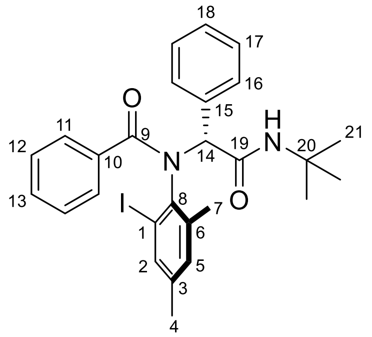

According to a modification of **General Procedure B**. To an oven-dried Schlenk tube, equipped with stirrer bar, was sequentially charged 2-iodo-4,6-dimethylaniline (297 mg, 1.20 mmol, 1.0 eq.), benzoic acid (147 mg, 1.20 mmol, 1.0 eq.), benzaldehyde (122 µL, 1.20 mmol, 1.0 eq.), 2,2,2-trifluoroethanol (2.4 mL, 0.5 M) and *tert*-butyl isocyanide (136 µL, 1.20 mmol, 1.0 eq). The mixture was stirred at room temperature for 24 hours. After this time a further portion of *tert*-butyl isocyanide (136 µL, 1.20 mmol, 1.0 eq.) was charged and stirred for a further 24 hours. The reaction was then concentrated to dryness and redissolved in dimethylsulfoxide (2.4 mL, 0.5 M), after which the reaction was heated to 150 °C for 5 hours in the dark. The solution was then cooled to room temperature and the reaction mixture was concentrated to dryness. The crude residue was purified by column chromatography (petrol 40-60/Et_2O 60:40) to afford *syn*-**1am** as an off-white solid (360 mg, 56% yield, 90:10 d.r.). An analytically pure sample of the *syn*-diastereoisomer was obtained by further column chromatography (petrol 40-60/EtOAc 83:17).

^{1}H NMR (400 MHz, $CDCl_3$): δ_H 7.49 – 7.42 (m, 4H, H_{11} and H_{16}), 7.27 – 7.08 (m, 7H, H_2, H_{12}, H_{13}, H_{17} and H_{18}), 6.65 (s, 1H, H_5), 5.93 (br s, 1H, NH), 5.54 (s, 1H, H_{14}), 2.36 (s, 3H, H_7), 2.03 (s, 3H, H_4), 1.38 (s, 9H, H_{21}).

^{13}C NMR (101 MHz, $CDCl_3$): δ_C 171.7, 168.4, 140.4, 139.5, 139.3, 137.9, 136.3, 133.2, 131.9, 130.9, 130.0, 128.6, 128.1, 127.9, 127.5, 103.9, 67.9, 51.7, 28.8, 20.4, 20.1.

IR (cm^{-1}) 3349, 2971, 2923, 1689, 1645, 1335, 697.

Melting Point (°C): 90 – 93.

HRMS (ESI): Exact mass calculated for $C_{27}H_{29}N_2INaO_2^+$ [M+Na]$^+$: 563.1166, found 563.1163.
Δ -0.5 ppm.

***rac*-(S_a,*R*)-N-(8-Bromonaphthalen-1-yl)-N-(2-(tert-butylamino)-2-oxo-1-phenylethyl)benzamide, *syn*-1as**

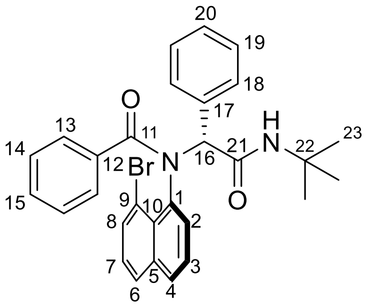

8-Bromonaphthalen-1-amine (67 mg, 0.30 mmol, 1.0 eq.), benzoic acid (37 mg, 0.30 mmol, 1.0 eq.), benzaldehyde (31 µL, 0.3 mmol, 1.0 eq.), 2,2,2-trifluoroethanol (0.6 mL, 0.5 M) and *tert*-butyl isocyanide (34 µL, 0.30 mmol, 1.0 eq.) were subjected to **General Procedure B**. Purification *via* column chromatography eluting with petrol 40-60/Et_2O 60:40 afforded the title compound *syn*-**1as** as a light orange solid (129 mg, 84% yield, 92:8 d.r.). An analytically pure sample of the *syn*-diastereoisomer was obtained by further column chromatography (petrol 40-60/Et_2O 65:35).

^{1}H NMR (400 MHz, $CDCl_3$): δ_H 8.12 (d, J = 7.3 Hz, 1H, H_2), 7.63 (d, J = 7.4 Hz, 1H, H_8), 7.53 – 7.47 (m, 2H, H_4 + H_6), 7.36 – 7.32 (m, 2H, H_{13}), 7.29 – 7.23 (m, 3H, H_3 + H_{18}), 7.05 – 6.95 (m, 4H, H_{Ar}), 6.91 – 6.80 (m, 3H, H_{Ar}), 5.96 (s, 1H, H_{16}), 5.60 (s, 1H, NH), 1.39 (s, 9H, H_{23}).

^{13}C NMR (101 MHz, $CDCl_3$): δ_C 170.9, 169.7, 136.9, 135.8, 135.3, 134.5, 134.2, 133.1, 131.0, 129.9, 129.4, 129.0, 129.0, 128.3, 128.1, 127.6, 127.4, 125.8, 125.3, 116.7, 68.1, 51.8, 28.8.

FTIR (cm^{-1}): 3442, 2968, 1682, 1635, 1364.

Melting Point (°C): 194-196.

HRMS (ESI): Exact mass calculated for $C_{29}H_{28}BrN_2O_2^+$ [M+H]$^+$: 515.1329, found 515.1326. Δ = - 0.6 ppm.

***rac*-(S_a,*R*)- N-(2-(*tert*-butyl)phenyl)-N-(2-(cyclohexylamino)-2-oxo-1-phenylethyl)-2,4-dimethylthiazole-5-carboxamide, *syn*-1aw**

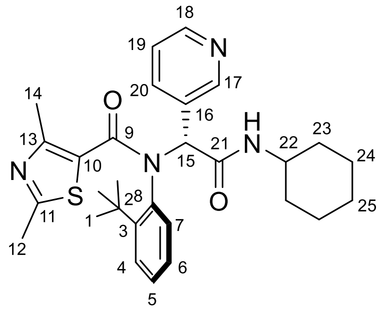

2-*tert*-Butylaniline (180 mg, 1.20 mmol, 1.0 eq.), 2,4-dimethyl-1,3-thizaole-5-carboxylic acid (189 mg, 1.20 mmol, 1.0 eq.), 3-pyridinecarboxaldehyde (113 µL, 1.20 mmol, 1.0 eq.), 2,2,2-trifluoroethanol (2.4 mL, 0.5 M) and cyclohexyl isocyanide (149 µL, 1.20 mmol, 1.0 eq) were subjected to **General Procedure B**. Purification via column chromatography eluting with Et_2O, afforded the title compound *syn*-**1aw** as a pale brown solid (209 mg, 35% yield, 89:11 d.r.). An analytically pure sample of the *syn*-diastereoisomer (>95:5 d.r.) was obtained by recrystallization from boiling hexane/EtOAc, which was used for biological testing.

^{1}H NMR (400 MHz, $CDCl_3$): δ_H 8.51 (d, *J* = 2.5 Hz, 1H, H_{17}), 8.43 (dd, *J* = 4.8, 1.6 Hz, 1H, H_{18}), 7.93 – 7.85 (m, 1H, H_7), 7.38 (dt, *J* = 8.2, 2.0 Hz, 1H, H_{20}), 7.35 – 7.22 (m, 3H, H_4, H_5 and H_6), 7.01 (dd, *J* = 8.1, 4.8 Hz, 1H, H_{19}), 6.30 – 6.17 (m, 1H, NH), 6.08 (s, 1H, H_{15}), 3.85 – 3.74 (m, 1H, H_{22}), 2.71 (s, 3H, H_{14}), 2.44 (s, 3H, H_{12}), 2.08 – 1.53 (m, 5H, H_{Cy}), 1.43 – 0.98 (m, 5H, H_{Cy}), 0.90 (s, 9H, H_1).

^{13}C NMR (101 MHz, $CDCl_3$): δ_C 168.7, 167.8, 164.5, 160.4, 152.4, 150.0, 148.8, 139.3, 135.0, 134.6, 131.3, 130.2, 129.3, 126.9, 122.9, 122.7, 64.6, 49.1, 36.3, 32.9, 32.8, 32.2, 25.6, 24.9, 24.8, 18.7, 18.7.

FTIR (cm^{-1}): 2972, 2931, 2855, 1662, 1625, 1490, 1375, 1344, 763, 732.

Melting Point (°C): 229 – 230.

HRMS (ESI): Exact mass calculated for $C_{29}H_{37}N_4O_2S^+$ $[M+H]^+$: 505.2632, found: 505.2642. Δ = 2.0 ppm.

4. *X-ray crystallography*

Single crystal diffraction data were collected on an XtaLAB Synergy HyPix-Arc 100 diffractometer using copper radiation ($\lambda_{CuK\alpha}$ = 1.54184 Å) at 150 K using an Oxford Cryosystems CryostreamPlus open-flow N_2 cooling device. Intensities were corrected for absorption using a multifaceted crystal model created by indexing the faces of the crystal for which data were collected.[3] Cell refinement, data collection and data reduction were undertaken via the software CrysAlisPro.[4]

All structures were solved using XT[5] and refined by XL[6] using the Olex2 interface.[7] All non-hydrogen atoms were refined anisotropically and hydrogen atoms were positioned with idealised geometry, with the exception of those bound to heteroatoms, the positions of which were located using peaks in the Fourier difference map. The displacement parameters of the hydrogen atoms were constrained using a riding model with $U_{(H)}$ set to be an appropriate multiple of the U_{eq} value of the parent atom.

5. Computational Modelling

5.1. Computational methods

All conformational searches were performed using MacroModel (Version 13.6)[8] in the gas phase utilizing the MMFF force field[9-14] and a mixture of Low Mode following and Monte Carlo search algorithms.[15,16] Quantum mechanical calculations were carried out using ORCA 5.0.2.[17] The molecular geometries were optimized using the PBE0-D3 functional[18,19] with the def2-TZVP basis set[20,21]. The optimisations were done using the implicit SMD solvent model.[22] Single-point energies were calculated using ωB97M-V functional,[23] def2-TZVP basis set and SMD solvent model.

Frequency calculations were performed on all structures and confirmed to contain no imaginary frequencies or just one imaginary frequency for ground states and transition states, respectively. Full set of DFT output files with optimized structures, frequencies and high-level single-point energies are provided in the accompanied archive. The archive structure is shown below in section 5.2.

5.2. Summary of the associated computational dataset contents

This dataset contains ORCA DFT output files of the key ground-states and transition state DFT optimized structures. The dataset contains 122 files in total. The data is organized to match the structure numbering in Scheme 3 in the manuscript. Each of the subfolders correspond to a step in the reaction pathway, and an additional folder for benzoic acid data. Each of the subfolders contain the output of the final optimization calculation (*opt*.out), frequency calculation (*freq.out) and single-point optimization (*_single_point.out). All optimized geometries are also provided as *.xyz files for even better usability. The full dataset structure is shown below.

All of the files can be opened in any text editor. ORCA output structures can be viewed and the frequency modes visualised in Avogadro, jmol and in most other molecular viewers/editors. *.xyz files can be viewed in essentially all 3D molecular editors and viewers.

```
├── BzOH
│   ├── BzOH_single_point.out
│   ├── BzOHfreq.out
│   ├── BzOHopt.out
│   └── BzOHopt.xyz
├── _1_2a
│   ├── ImineE1_single_point.out
│   ├── ImineE1freq.out
│   ├── ImineE1opt.out
```

```
│   ├── ImineE1opt.xyz
│   ├── ImineE2_single_point.out
│   ├── ImineE2freq.out
│   ├── ImineE2opt.out
│   ├── ImineE2opt.xyz
│   ├── ImineE3_single_point.out
│   ├── ImineE3freq.out
│   ├── ImineE3opt.out
│   ├── ImineE3opt.xyz
│   ├── ImineZ1_single_point.out
│   ├── ImineZ1freq.out
│   ├── ImineZ1opt.out
│   ├── ImineZ1opt.xyz
│   ├── ImineZ2_single_point.out
│   ├── ImineZ2freq.out
│   ├── ImineZ2opt.out
│   ├── ImineZ2opt.xyz
│   ├── ImineZ3_single_point.out
│   ├── ImineZ3freq.out
│   ├── ImineZ3opt.out
│   ├── ImineZ3opt.xyz
│   ├── ImineZ4_single_point.out
│   ├── ImineZ4freq.out
│   ├── ImineZ4optb.out
│   ├── ImineZ4optb.xyz
│   ├── ImineZ5_single_point.out
│   ├── ImineZ5freq.out
│   ├── ImineZ5opt.out
│   ├── ImineZ5opt.xyz
│   ├── ImineZ6_single_point.out
│   ├── ImineZ6freq.out
│   ├── ImineZ6opt.out
│   └── ImineZ6opt.xyz
├── _2_Imine-BzOH
│   ├── ImineBzOH10rAfreq.out
│   ├── ImineBzOH10r_single_point.out
│   ├── ImineBzOH10rfreq.out
│   ├── ImineBzOH10ropt.out
│   ├── ImineBzOH10ropt.xyz
│   ├── ImineBzOH11rAfreq.out
│   ├── ImineBzOH11r_single_point.out
│   ├── ImineBzOH11rfreq.out
│   ├── ImineBzOH11ropt.out
│   ├── ImineBzOH11ropt.xyz
│   ├── ImineBzOH12rAfreq.out
│   ├── ImineBzOH12r_single_point.out
│   ├── ImineBzOH12ropt.out
│   ├── ImineBzOH12ropt.xyz
│   ├── ImineBzOH1Afreq.out
│   ├── ImineBzOH1_single_point.out
│   ├── ImineBzOH1opt.out
```

```
│   ├── ImineBzOH1opt.xyz
│   ├── ImineBzOH2Afreq.out
│   ├── ImineBzOH2_single_point.out
│   ├── ImineBzOH2optb.out
│   ├── ImineBzOH2optb.xyz
│   ├── ImineBzOH3Afreq.out
│   ├── ImineBzOH3_single_point.out
│   ├── ImineBzOH3opt.out
│   ├── ImineBzOH3opt.xyz
│   ├── ImineBzOH4Afreq.out
│   ├── ImineBzOH4_single_point.out
│   ├── ImineBzOH4optb.out
│   ├── ImineBzOH4optb.xyz
│   ├── ImineBzOH5Afreq.out
│   ├── ImineBzOH5_single_point.out
│   ├── ImineBzOH5optc.out
│   ├── ImineBzOH5optc.xyz
│   ├── ImineBzOH6Afreq.out
│   ├── ImineBzOH6_single_point.out
│   ├── ImineBzOH6opt.out
│   ├── ImineBzOH6opt.xyz
│   ├── ImineBzOH7Afreq.out
│   ├── ImineBzOH7_single_point.out
│   ├── ImineBzOH7opt.out
│   ├── ImineBzOH7opt.xyz
│   ├── ImineBzOH8rAfreq.out
│   ├── ImineBzOH8r_single_point.out
│   ├── ImineBzOH8ropt.out
│   ├── ImineBzOH8ropt.xyz
│   ├── ImineBzOH9Afreq.out
│   ├── ImineBzOH9_single_point.out
│   ├── ImineBzOH9opt.out
│   └── ImineBzOH9opt.xyz
├── _3_3a
│   ├── IminiumBzOate1_single_point.out
│   ├── IminiumBzOate1freq.out
│   ├── IminiumBzOate1opt.out
│   ├── IminiumBzOate1opt.xyz
│   ├── IminiumBzOate2_single_point.out
│   ├── IminiumBzOate2freq.out
│   ├── IminiumBzOate2opt.out
│   ├── IminiumBzOate2opt.xyz
│   ├── IminiumBzOate3_single_point.out
│   ├── IminiumBzOate3freq.out
│   ├── IminiumBzOate3optb.out
│   ├── IminiumBzOate3optb.xyz
│   ├── IminiumBzOate4_single_point.out
│   ├── IminiumBzOate4freq.out
│   ├── IminiumBzOate4optb.out
│   ├── IminiumBzOate4optb.xyz
│   ├── IminiumBzOate_R3_single_point.out
```

```
│   ├── IminiumBzOate_R3freq.out
│   ├── IminiumBzOate_R3opt.out
│   ├── IminiumBzOate_R3opt.xyz
│   ├── IminiumBzOate_S3_single_point.out
│   ├── IminiumBzOate_S3freq.out
│   ├── IminiumBzOate_S3optb.out
│   └── IminiumBzOate_S3optb.xyz
├── _4_TS
│   ├── ImineBzOHTS_R3B_single_point.out
│   ├── ImineBzOHTS_R3Btsopt.out
│   ├── ImineBzOHTS_R3Btsopt.xyz
│   ├── ImineBzOHTS_R3Cfreq.out
│   ├── ImineBzOHTS_S3D_single_point.out
│   ├── ImineBzOHTS_S3Dfreq.out
│   ├── ImineBzOHTS_S3Dtsoptc.out
│   └── ImineBzOHTS_S3Dtsoptc.xyz
├── _5_1a-BzOH
│   ├── AmidiniumBzoateR3_single_point.out
│   ├── AmidiniumBzoateR3freq.out
│   ├── AmidiniumBzoateR3opt.out
│   ├── AmidiniumBzoateR3opt.xyz
│   ├── AmidiniumBzoateS3B_single_point.out
│   ├── AmidiniumBzoateS3Bfreq.out
│   ├── AmidiniumBzoateS3Boptc.out
│   ├── AmidiniumBzoateS3Boptc.xyz
│   ├── ImineBzOH6post_single_point.out
│   ├── ImineBzOH6postfreq.out
│   ├── ImineBzOH6postopt.out
│   ├── ImineBzOH6postopt.xyz
│   ├── ImineBzOH8rpost_single_point.out
│   ├── ImineBzOH8rpostfreq.out
│   ├── ImineBzOH8rpostopt.out
│   └── ImineBzOH8rpostopt.xyz
└── _6_1a
    ├── Prod_RR1_single_point.out
    ├── Prod_RR1freq.out
    ├── Prod_RR1opt.out
    ├── Prod_RR1opt.xyz
    ├── Prod_RR2_single_point.out
    ├── Prod_RR2freq.out
    ├── Prod_RR2opt.out
    ├── Prod_RR2opt.xyz
    ├── Prod_RR3_single_point.out
    ├── Prod_RR3freq.out
    ├── Prod_RR3opt.out
    ├── Prod_RR3opt.xyz
    ├── Prod_RS1_single_point.out
    ├── Prod_RS1freq.out
    ├── Prod_RS1opt.out
    ├── Prod_RS1opt.xyz
    ├── Prod_RS2_single_point.out
```

```
├── Prod_RS2freq.out
├── Prod_RS2opt.out
├── Prod_RS2opt.xyz
├── Prod_RS3_single_point.out
├── Prod_RS3freq.out
├── Prod_RS3opt.out
└── Prod_RS3opt.xyz
```

6. M. tuberculosis inhibition

6.1 Results

Table S1 – Antibacterial activity analysis of compounds *anti*-**1aw** and *syn*-**1aw** alongside control compounds (**S4**,[24] **RIF**, **INH**, **EMB** and **ETH**) was performed by REMA against drug susceptible and resistant *Mtb*, and *Mab*. Values correspond to minimum inhibitory concentrations (MIC) 90% and reported in µM. Values of "–" correspond to no activity observed at 64 µM.

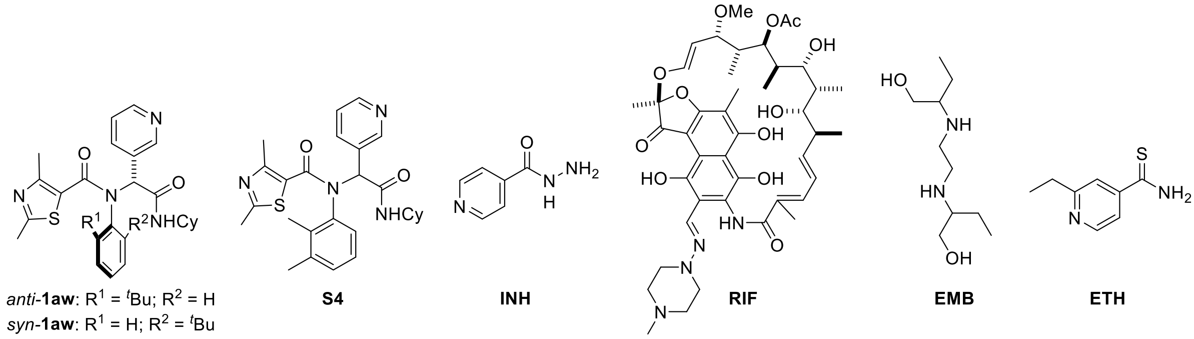

	Mtb mc²7902	*Mtb* mc²8245 INH^R	*Mtb* mc²8247 RIF^R	*Mtb* mc²8250 INH^R RIF^R	*Mab* NCTC 13031
anti-**1aw**	–	–	–	–	–
syn-**1aw**	31.7 ± 1.470	34.7 ± 0.955	33.6 ± 1.269	38.1 ± 0.314	–
**S4**	0.55 ± 0.004	0.55 ± 0.049	0.48 ± 0.037	0.56 ± 0.033	–
**INH**	0.06 ± 0.006	–	0.06 ± 0.005	–	–
**RIF**	0.02 ± 0.001	0.02 ± 0.003	–	–	–
**EMB**	8.79 ± 0.567	9.89 ± 0.332	9.69 ± 0.155	9.77 ± 0.487	–
**ETH**	6.02 ± 0.580	6.11 ± 0.473	5.88 ± 0.457	6.51 ± 0.912	16.23 ± 1.118

6.2 Materials and methods

Mycobacterial species were cultured in either Middlebrook 7H9 broth or Middlebrook 7H10 agar media supplemented with albumin–dextrose–catalase (ADC) or oleic acid–albumin–dextrose–catalase (OADC) enrichments purchased from BD Biosciences and 0.2% glycerol, 0.2% w/v casamino acids, 24 µg/mL pantothenate, 200 µg/mL arginine, 50 µg/mL leucine, 1 µg/mL penicillin G, and 10 µg/mL

cyclohexamide, (7H9OPALPen^{1}Cyc^{10}). All reagents were purchased from Sigma-Aldrich unless stated otherwise.

Bacterial strains: BSL2 mycobacterial species used in this study were all kindly supplied by Prof William R. Jacobs Jr (Table S3).[25] Non-mycobacterial strains were purchased from National Collection of Type Cultures, UK (Table S4).

Bacterial Growth Inhibition Assays: Bacterial minimum inhibition concentrations (MIC) were determined using the Resazurin Microtiter Assay (REMA) method according to Palomino *et al*.[26] Stock solutions of the tested compounds were prepared in sterile dimethyl sulfoxide (DMSO), then diluted in 7H9OPALPen^{1}Cyc^{10} to obtain a final drug concentration range of 0.032 – 32 µg/mL. A suspension of the test *Mycobacterium* was cultured in 7H9OPALPen^{1}Cyc^{10} containing 0.05% (v/v) Tween 80 for one week at 37 °C, 5% CO_2. The bacterial suspension was adjusted to McFarland 0.5 and diluted in 7H9OPALPen^{1}Cyc^{10} 1:25. 100 µL of the inoculum was added to each well of a 96-well microplate together with 100 µL of the compound titration. The plate was incubated at 37 °C, 5% CO_2. After 5 days, 10.5 µL 0.05% (w/v) sterile resazurin in 0.02% (v/v) Tween^{80} was added. Reduced resazurin was detected using fluorescence (Ex/Em 530/590 nm) in a FLUOstar Optima, BMG Labtech. All titre measurements were plotted in SigmaPlot™ and 4-parameter logistic (4PL) model regressions conducted. The relative MIC_{90} of each curve was calculated and averaged, standard errors were reported for all MIC (Table S1). Testing was performed in duplicate with two independent biological repeats. Similarly, REMA assays were performed for the non-mycobacterial organisms but in the optimal media suggested by the EUCAST to determine the MIC_{95}. Though, for these cultures, dilutions were performed at 1:200 prior to inoculation of the plate and incubation for 4 hours (Table S4).

Table S2 – *Mycobacterium* strains used in this study.

Strains	Description	Source or reference
Mtb mc^{2}7902	*ΔleuCD ΔpanCD ΔargB,* Leucine, pantothenate and arginine triple auxotroph	Vilcheze *et al.*[25]
Mtb mc^{2}8245 (INH^R)	mc^{2}7902 derived Δ2116169–2162530; Δ2116169–2162530 genome deletion, INH^R	Vilcheze *et al.*[25]
Mtb mc^{2}8247 (RIF^R)	mc^{2}7902 derived, *rpoB* (H445Y); *rpoB* His445 → Lys, RIF^R	Vilcheze *et al.*[25]
Mtb mc^{2}8250 (RIF^R & INH^R)	mc^{2}8247 derived, *rpoB* (H445Y) Δ2122397–2170320; *rpoB* His445 → Lys, RIF^R, Δ2122397–2170320 genome deletion, INH^R	Vilcheze *et al.*[25]
Mab	*Mycobacterium abscessus* NCTC 13031	National Collection of Type Cultures, UK

Table S3 – Non-mycobacterial strains used in this study and growth media used.

Strain	Media used for MIC determination
Enterococcus faecalis NCTC 775	Mueller Hinton broth
Enterococcus faecium NCTC 7171	Mueller Hinton broth
Staphylococcus aureus NCTC 2981	Mueller Hinton broth
Klebsiella pneumoniae NCTC 9633	Mueller Hinton broth
Acinetobacter baumannii NCTC 12156	Mueller Hinton broth
Pseudomonas aeruginosa DSM 19880	Mueller Hinton broth
Enterobacter cloacae NCTC 10005	Mueller Hinton broth
Escherichia coli K12	Mueller Hinton broth

Table S4 – Non-tuberculosis REMA assay MIC determination.

	Minimum inhibitory concentration 95% (µM)						
	anti-**1aw**	*syn*-**1aw**	**S4**	**RIF**	**INH**	**ETH**	**EMB**
E. faecalis NCTC 775	>32	>32	>32	>12.8	>32	>32	>32
E. faecium NCTC 7171	>32	>32	>32	1.733 ± 0.017	>32	>32	>32
S. aureus NCTC 2981	>32	>32	>32	<0.0125	>32	>32	>32
K. pneumoniae NCTC 9633	>32	>32	>32	>12.8	>32	>32	>32
A. baumannii NCTC 12156	>32	>32	>32	1.363 ± 0.012	>32	>32	>32
P. aeruginosa DSM 19880	>32	>32	>32	>12.8	>32	>32	>32
E. cloacae NCTC 10005	>32	>32	>32	>12.8	>32	>32	>32
E. coli K12	>32	>32	>32	3.034 ± 0.001	>32	>32	>32

7. References

[1] *Purification of Laboratory Chemicals*, 3rd edition. D. D. Perrin, W. L. F. Armarego, *Pergamon Press*, Oxford, **1988**.

[2] S. Sharma, R. A. Maurya, K.-I. Min, G.-Y. Jeong, D.-P. Kim, *Angew. Chem. Int. Ed.*, **2013**, *52*, 7564.

[3] R. C. Clark, J. S. Reid, *Acta Cryst.,* **1995**, *A51*, 887.

[4] CrysAlisPro, Rigaku Oxford Diffraction, Tokyo, Japan.

[5] G. M. Sheldrick, *Acta Crystallogr., Sect. A: Found. Crystallogr.* **2015,** *71*, 3.

[6] G. M. Sheldrick, *Acta Crystallogr., Sect. A: Found. Crystallogr.* **2008,** *64*, 112.

[7] O. V. Dolomanov, L. J. Bourhis, R. J. Gildea, J. A. K Howard, H. Puschmann, *J. Appl. Cryst.* **2009,** *42*, 339.

[8] MacroModel, version 13.6, Schrödinger, LLC, New York, NY, 2022.

[9] T. A. Halgren, *J. Comput. Chem.*, **1996**, *17*, 490.

[10] T. A. Halgren, *J. Comput. Chem.*, **1996**, *17*, 520.

[11] T. A. Halgren, *J. Comput. Chem.*, **1996**, *17*, 553.

[12] T. A. Halgren, R. B. Nachbar, *J. Comput. Chem.*, **1996**, *17*, 587.

[13] T. A. Halgren, *J. Comput. Chem.*, **1996**, *17*, 616.

[14] T. A. Halgren, *J. Comput. Chem.*, **1999**, *20*, 720.

[15] I. Kolossváry, W. C. Guida, *J. Comput. Chem.*, **1999**, *20*, 1671.

[16] I. Kolossváry, W. C. Guida, *J. Am. Chem. Soc.*, **1996**, *118*, 5011.

[17] F. Neese, F. Wennmohs, U. Becker, C. Riplinger, *J. Chem. Phys.* **2020**, *152*, 224108.

[18] C. Adamo, V. Barone, *J. Chem. Phys.*, **1999**, *110*, 6158.

[19] S. Grimme, J. Antony, S. Ehrlich, H. Krieg, *J. Chem. Phys.*, **2010**, *132*, 154104.

[20] F. Weigend, R. Ahlrichs, *Phys. Chem. Chem. Phys.* **2005**, *7*, 3297.

[21] F. Weigend, *Phys. Chem. Chem. Phys.* **2006**, *8*, 1057.

[22] A. V. Marenich, C. J. Cramer, D. G. Truhlar, *J. Phys. Chem. B* **2009**, *113*, 6378.

[23] N. Mardirossian, M. Head-Gordon, *J. Chem. Phys.* **2016**, *144*, 214110.

[24] V. B. Makane, V. S. Krishna, E. V. Krishna, M. Shukla, B. Mahizhaveni, S. Misra, S. Chopra, D. Sriram, V. N. A. Dusthackeer, H. B. Rode, *Eur. J. Med. Chem.* **2019**, *164*, 665.

[25] C. Vilchèze, J. Copeland, T. L. Keiser, T. Weisbrod, J. Washington, P. Jain, A. Malek, B. Weinrick, W. R. Jacobs, *mBio* **2018**, *9*, 10.1128/mbio.00938-18.

[26] J.-C. Palomino, A. Martin, M. Camacho, H. Guerra, J. Swings, F. Portaels, *Antimicrob. Agents Chemother.* **2002**, *46*, 2720.

8. NMR Spectra

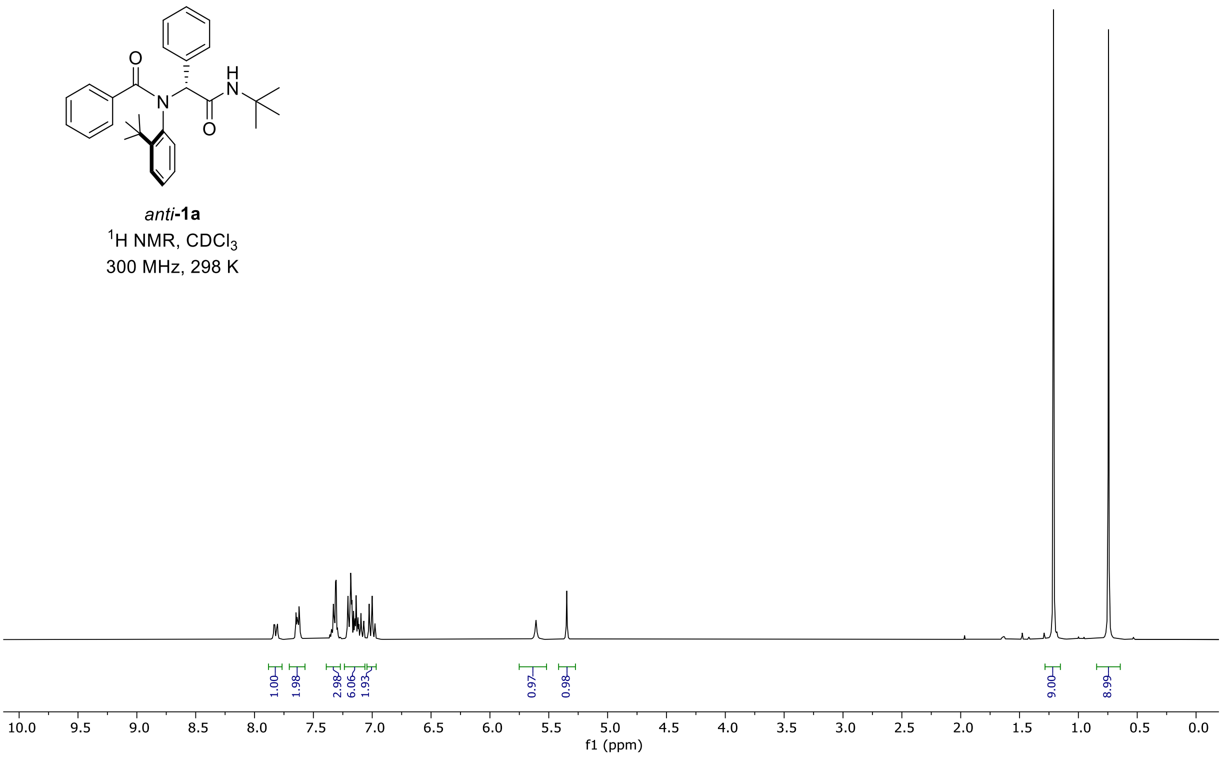

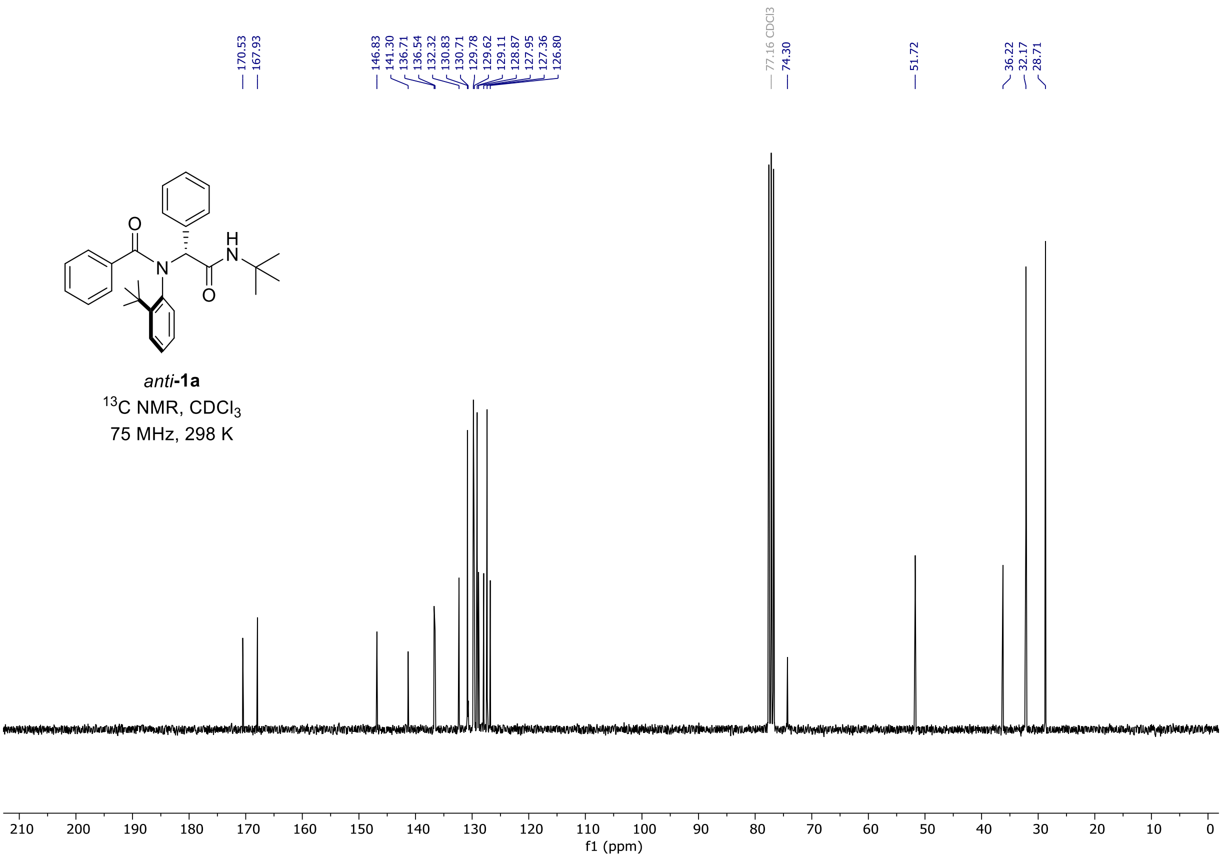

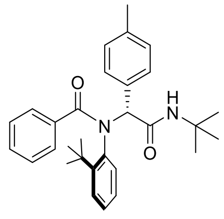

anti-**1b**
^{1}H NMR, $CDCl_3$
300 MHz, 298 K

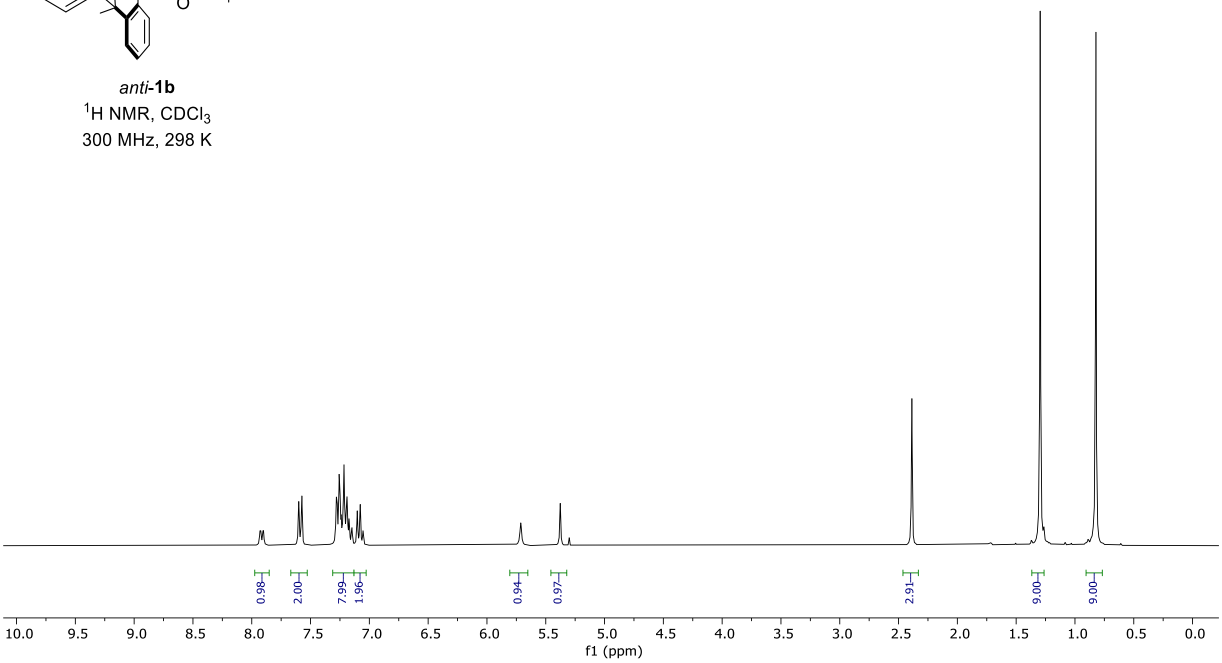

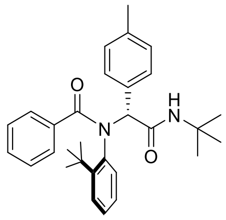

anti-**1b**
^{13}C NMR, $CDCl_3$
75 MHz, 298 K

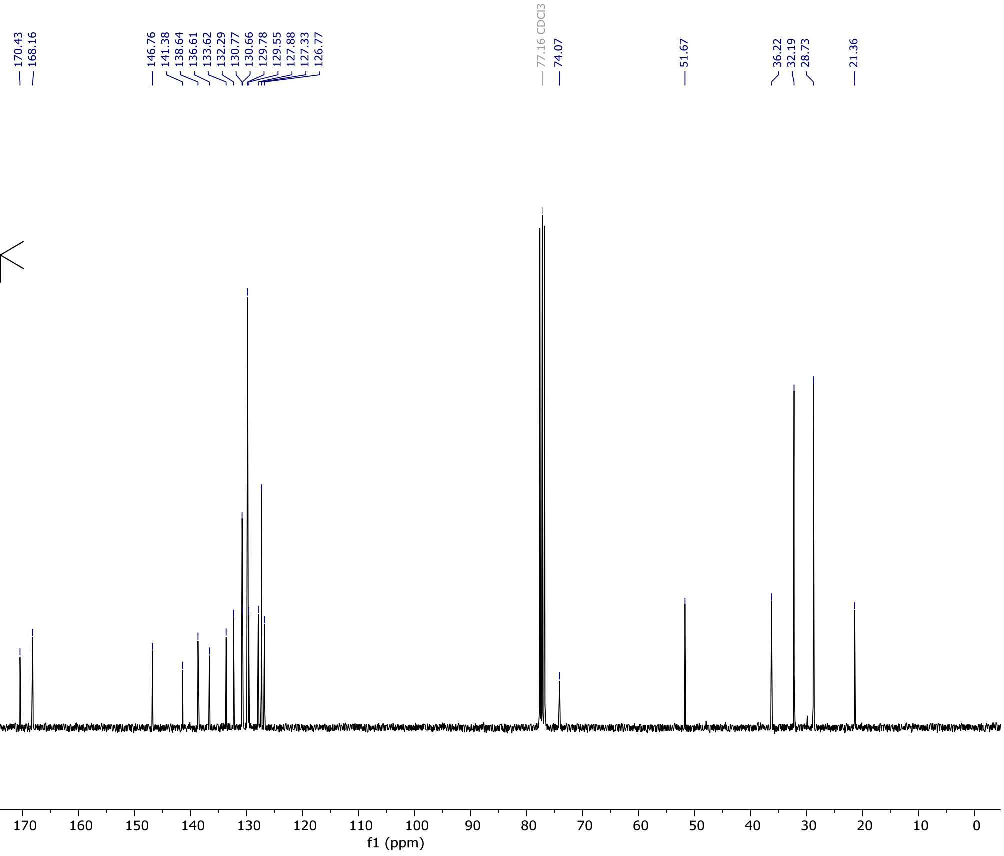

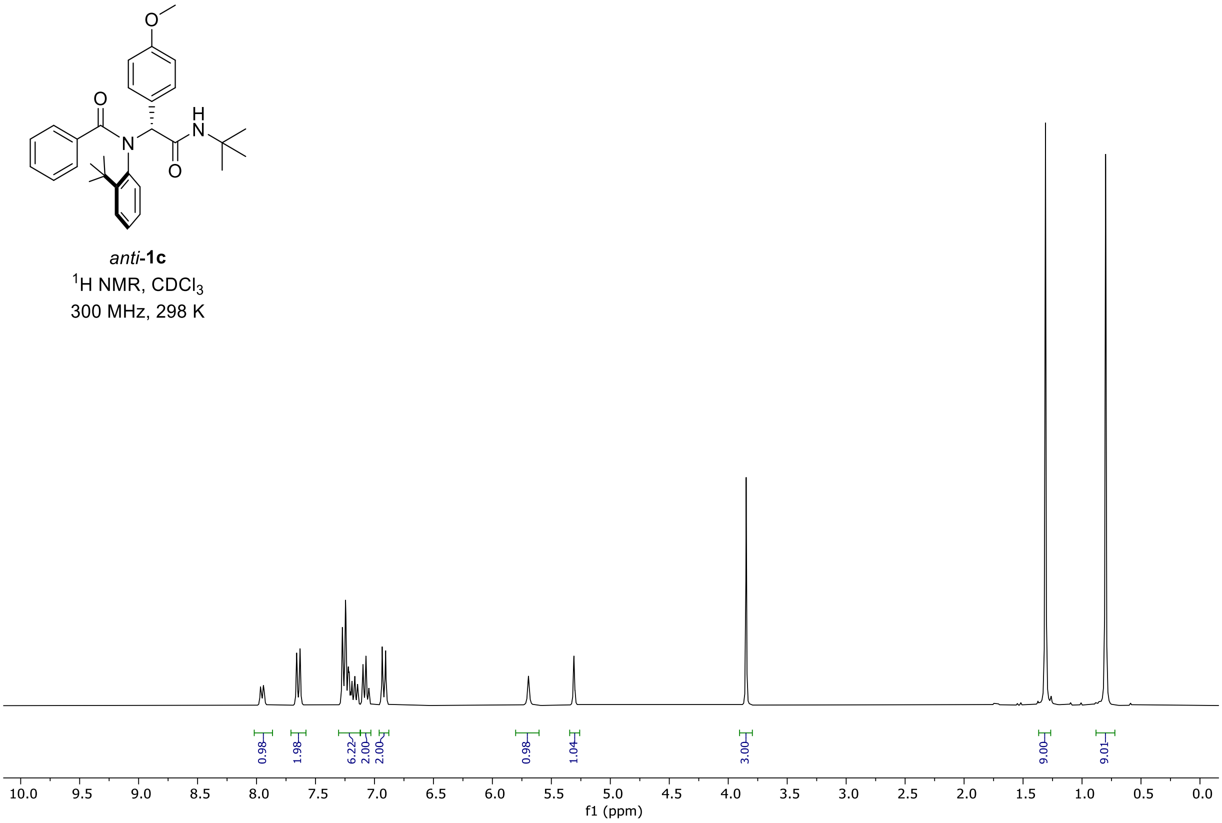

anti-1c
1H NMR, CDCl3
300 MHz, 298 K
0.98
1.98
6.22
2.00
2.00
0.98
1.04
3.00
9.00
9.01
f1 (ppm)

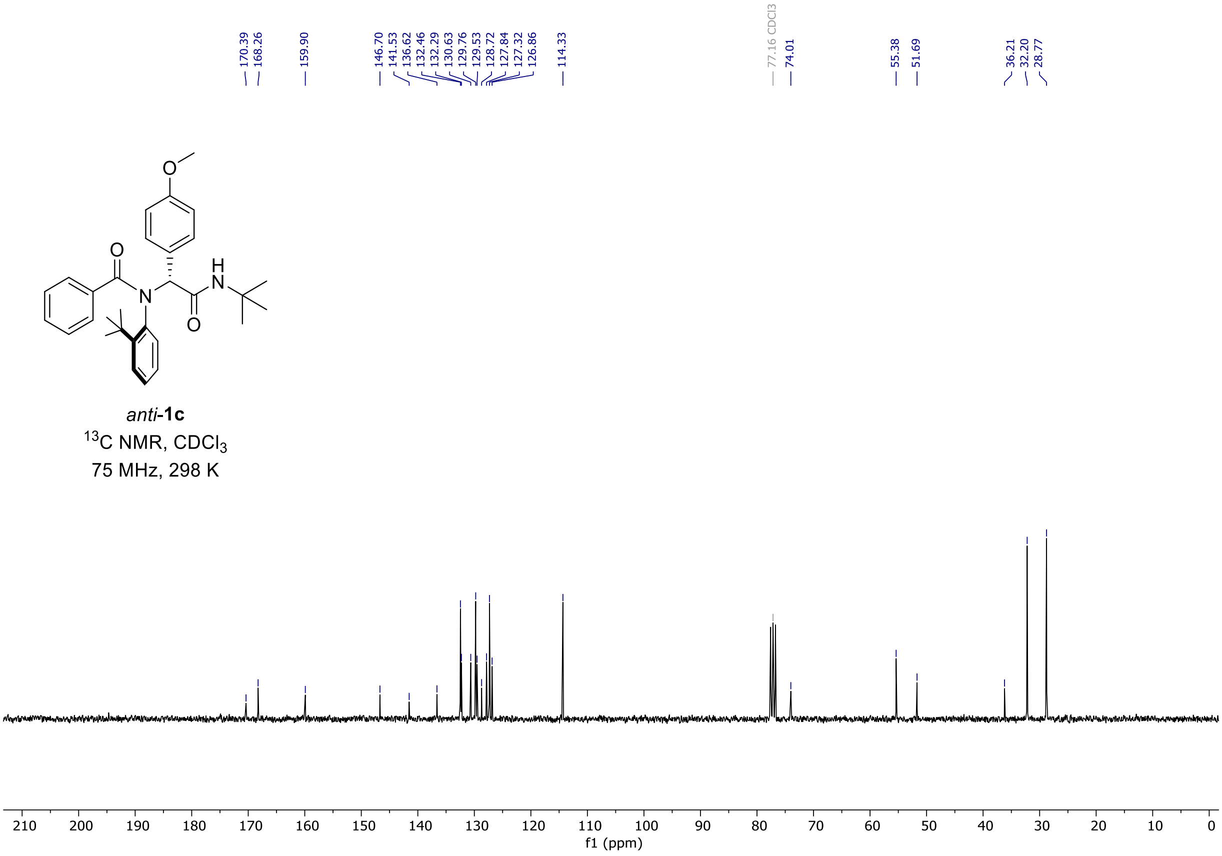

170.39
168.26
159.90
146.70
141.53
136.62
132.46
132.29
130.63
129.76
129.53
128.72
127.84
127.32
126.86
114.33
77.16 CDCl3
74.01
55.38
51.69
36.21
32.20
28.77
anti-1c
13C NMR, CDCl3
75 MHz, 298 K
f1 (ppm)

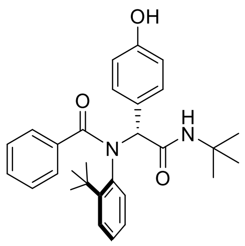

anti-**1d**
^{1}H NMR, $CDCl_3$
300 MHz, 298 K

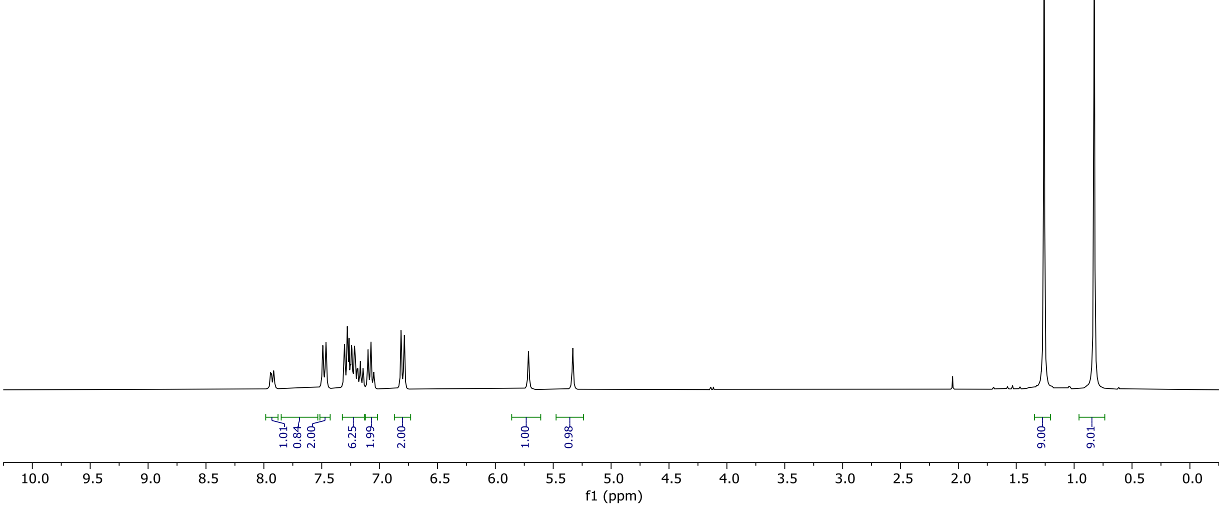

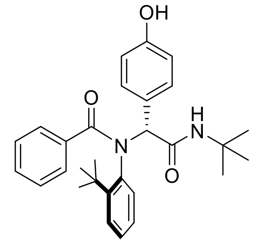

anti-**1d**
^{13}C NMR, $CDCl_3$
75 MHz, 298 K

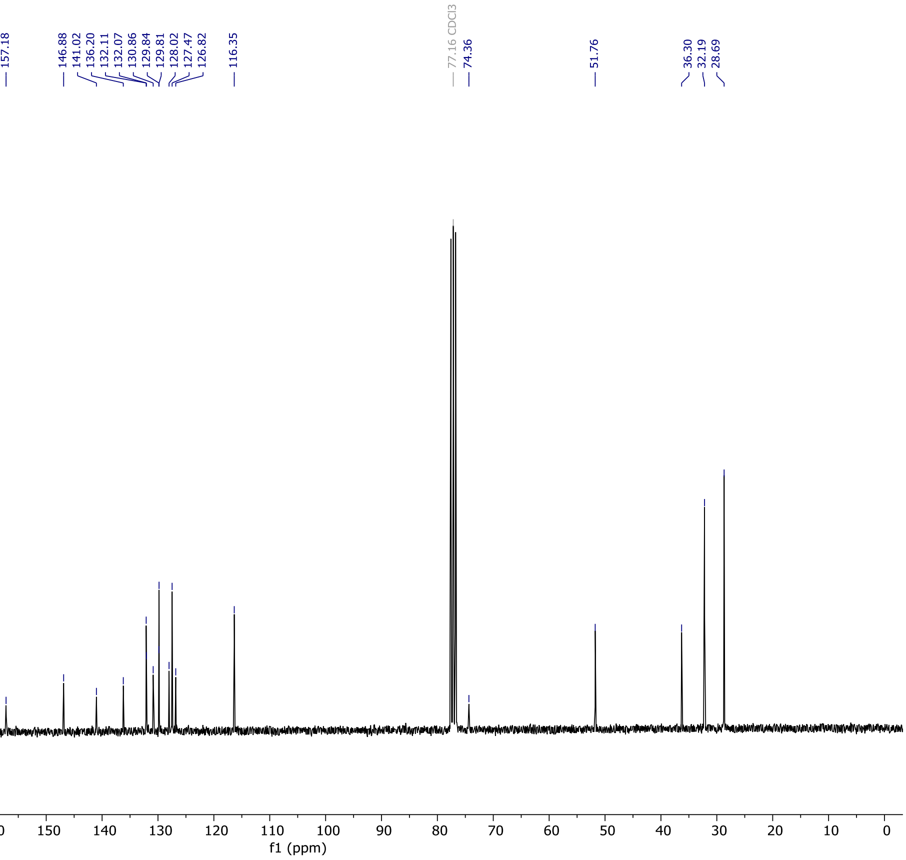

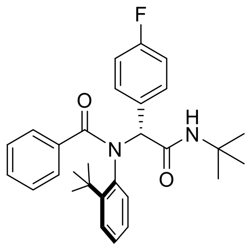

anti-**1e**
^{1}H NMR, $CDCl_3$
300 MHz, 298 K

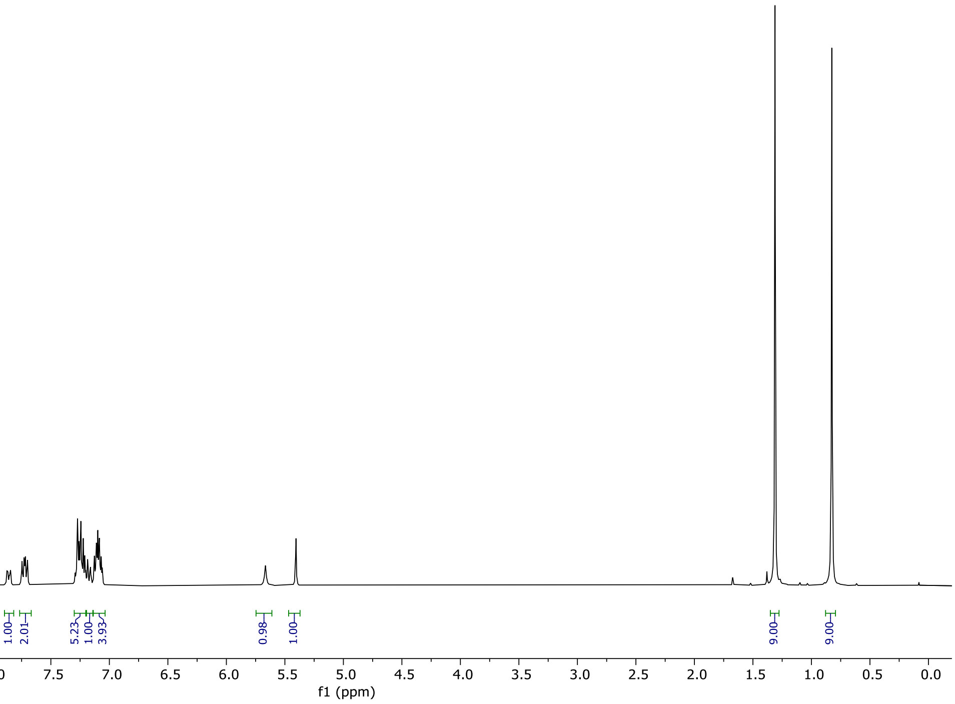

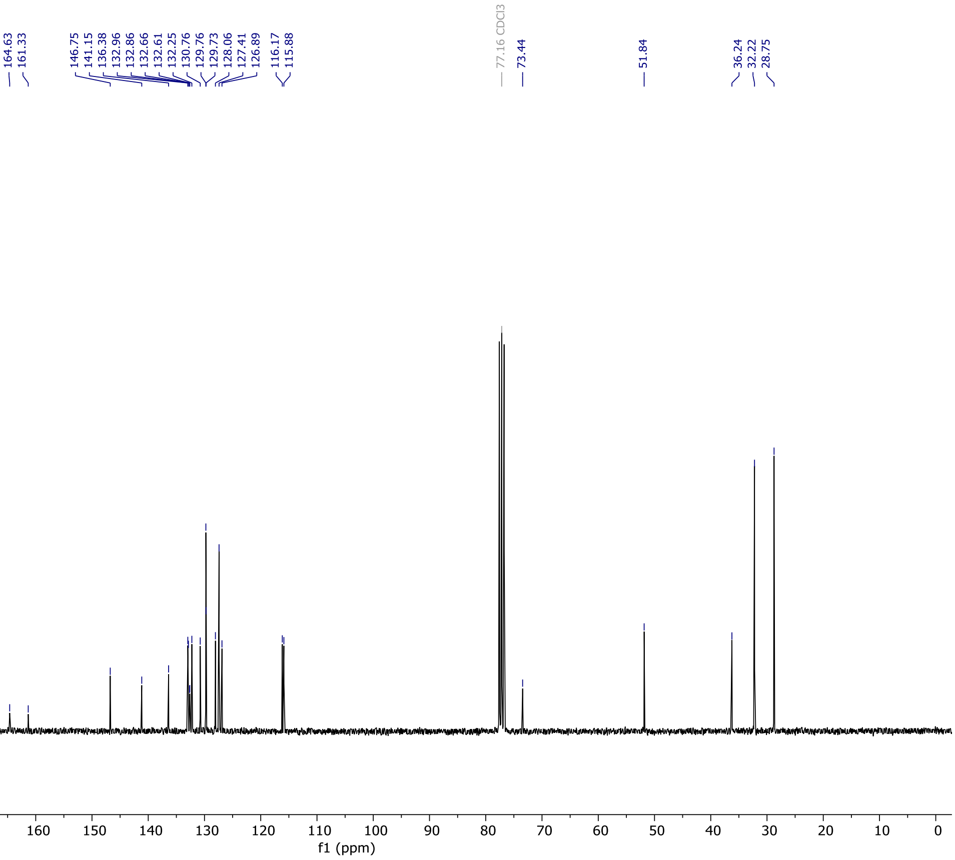

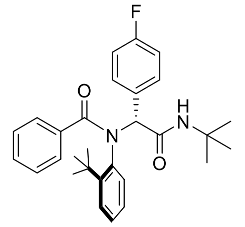

anti-**1e**
^{13}C NMR, $CDCl_3$
75 MHz, 298 K

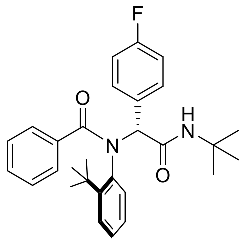

anti-**1e**
^{19}F NMR, $CDCl_3$
282 MHz, 298 K

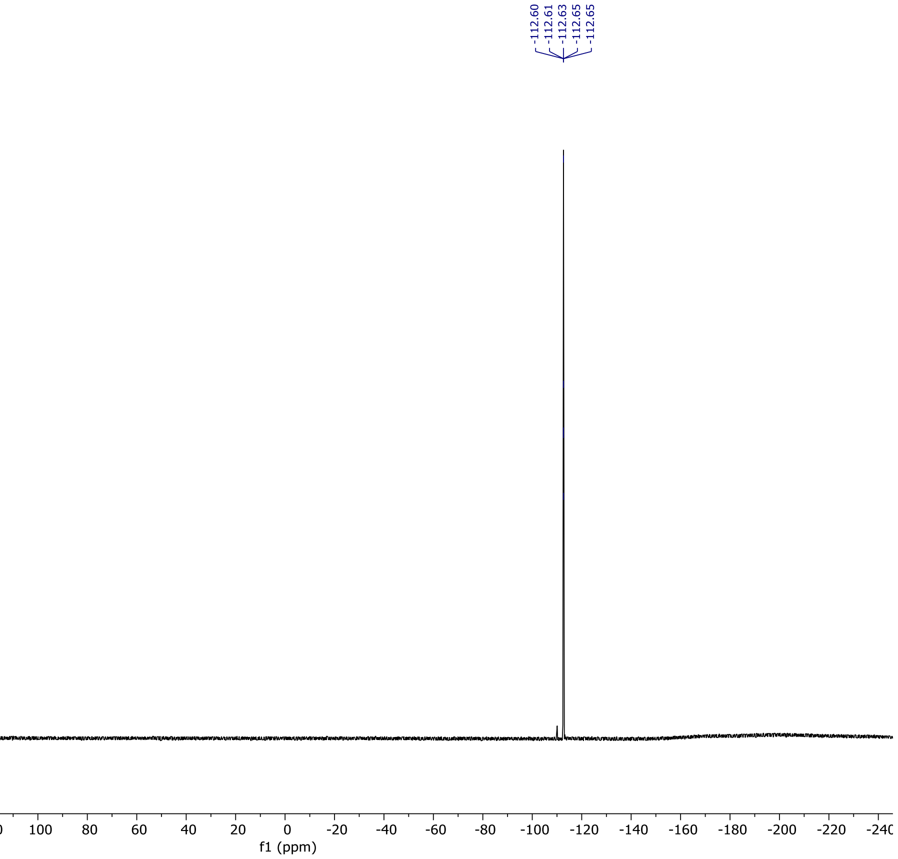

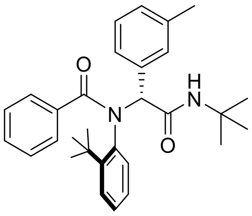

anti-**1f**
^{1}H NMR, $CDCl_3$
300 MHz, 298 K

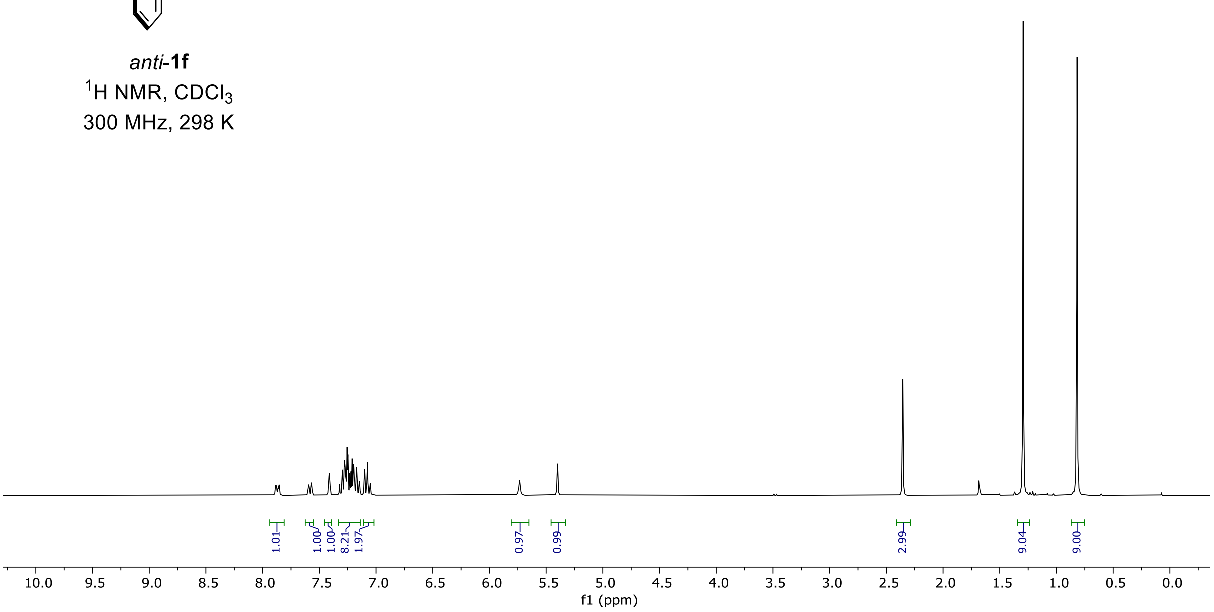

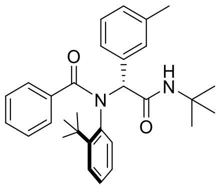

anti-**1f**
^{13}C NMR, $CDCl_3$
75 MHz, 298 K

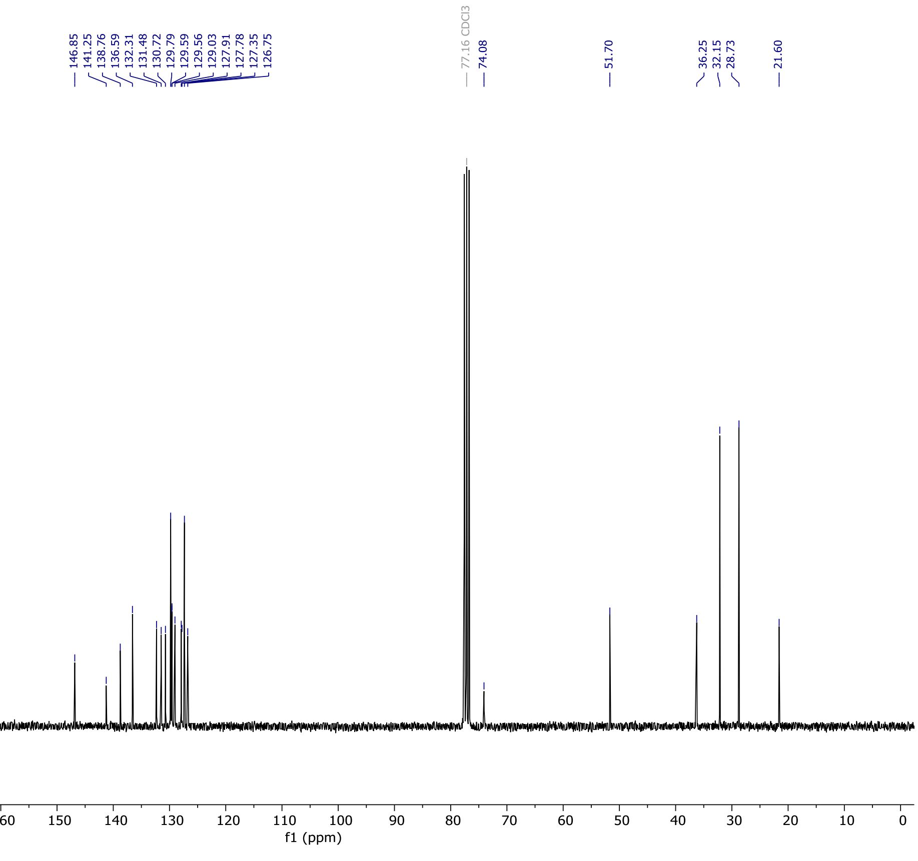

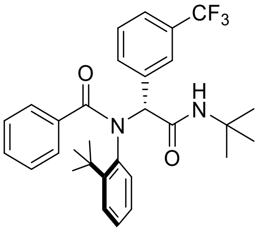

anti-**1g**

^{1}H NMR, $CDCl_3$

300 MHz, 298 K

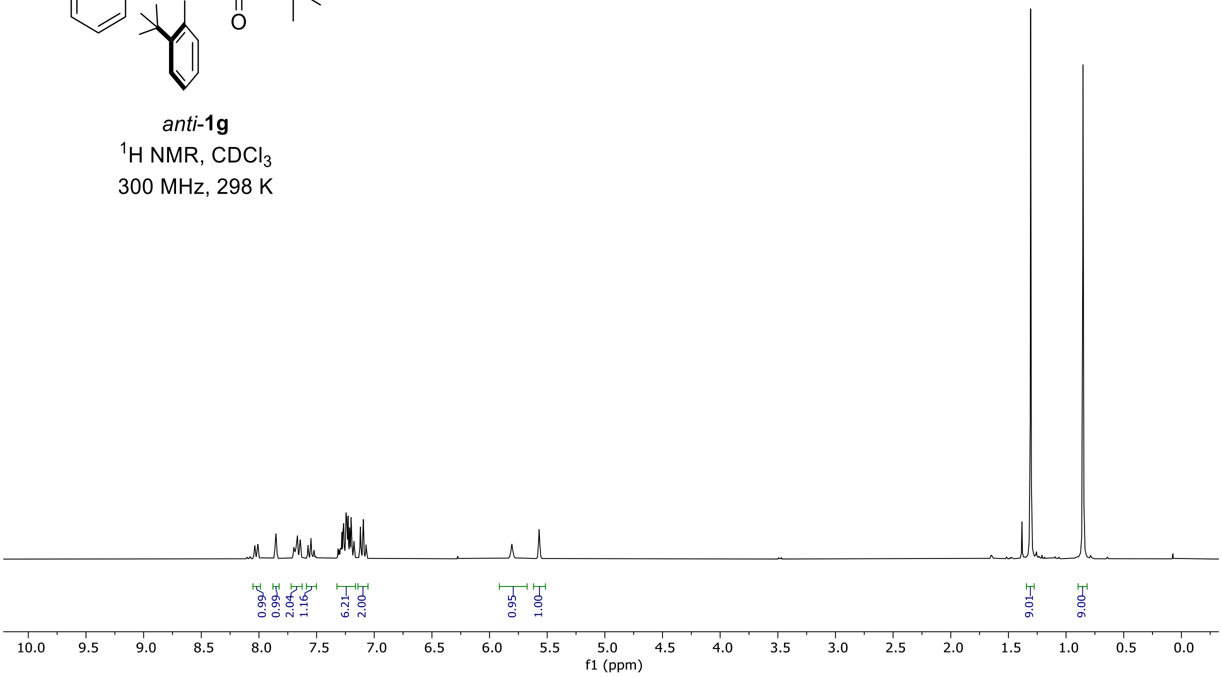

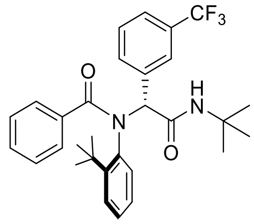

anti-**1g**

^{13}C NMR, $CDCl_3$

101 MHz, 298 K

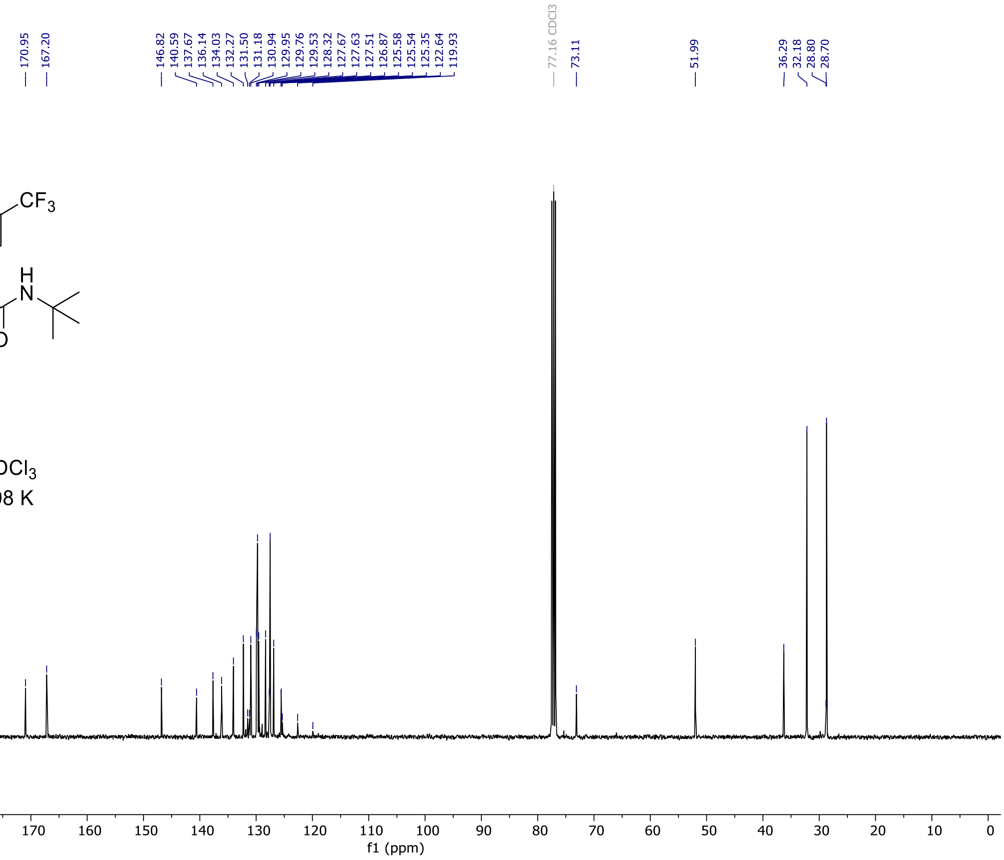

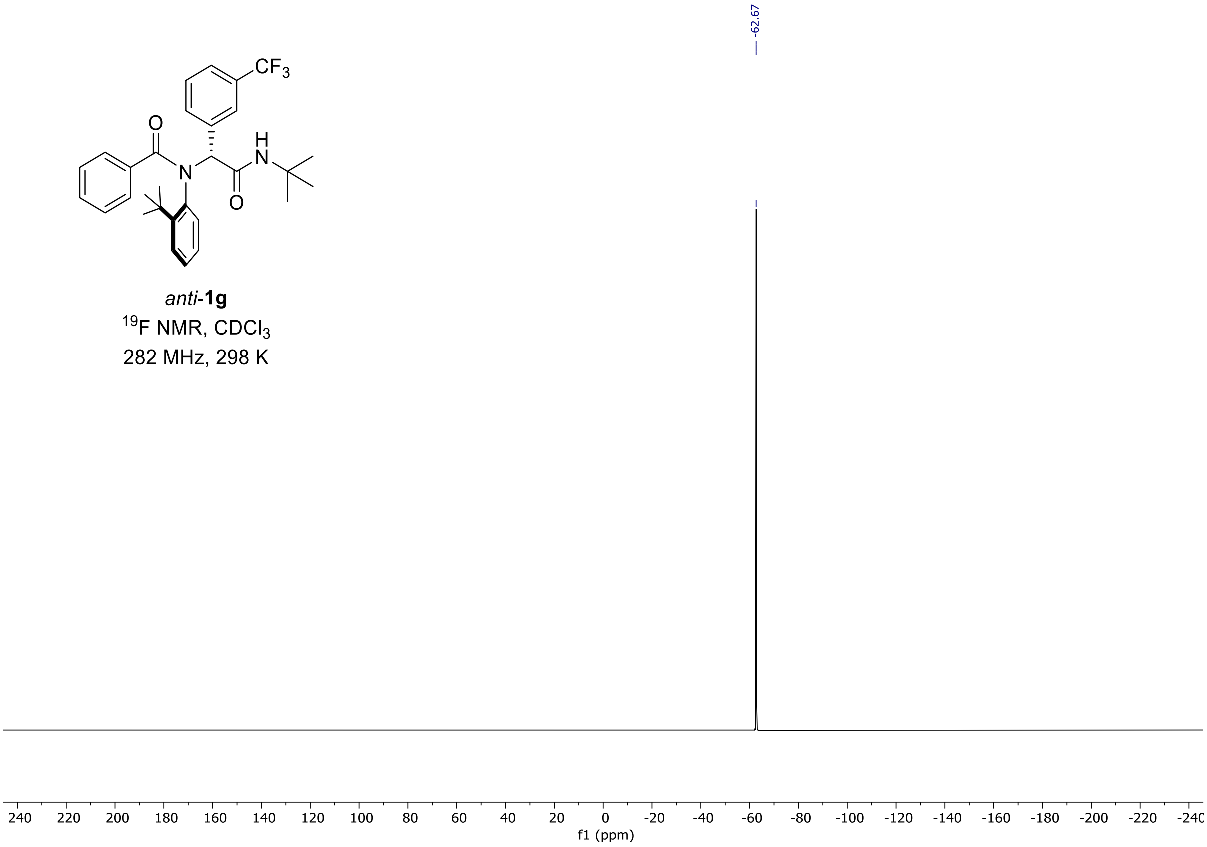

anti-**1g**
^{19}F NMR, $CDCl_3$
282 MHz, 298 K

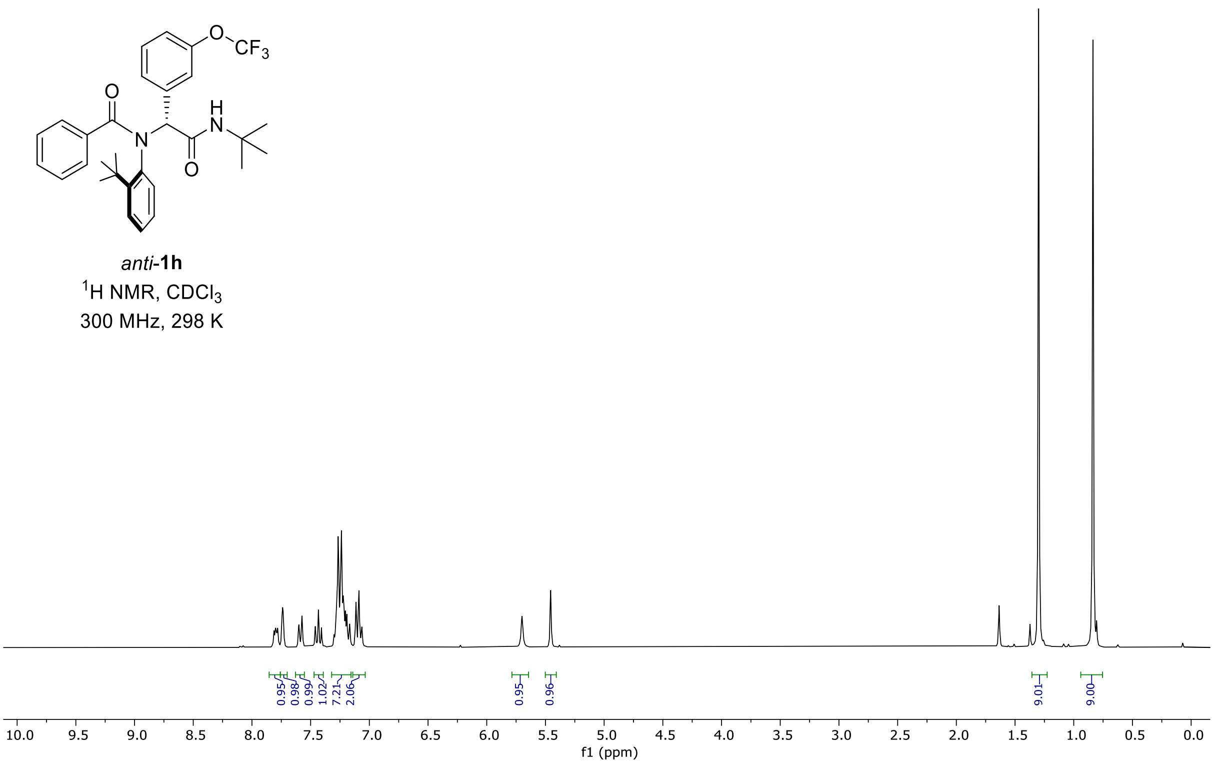

anti-**1h**
^{1}H NMR, $CDCl_3$
300 MHz, 298 K

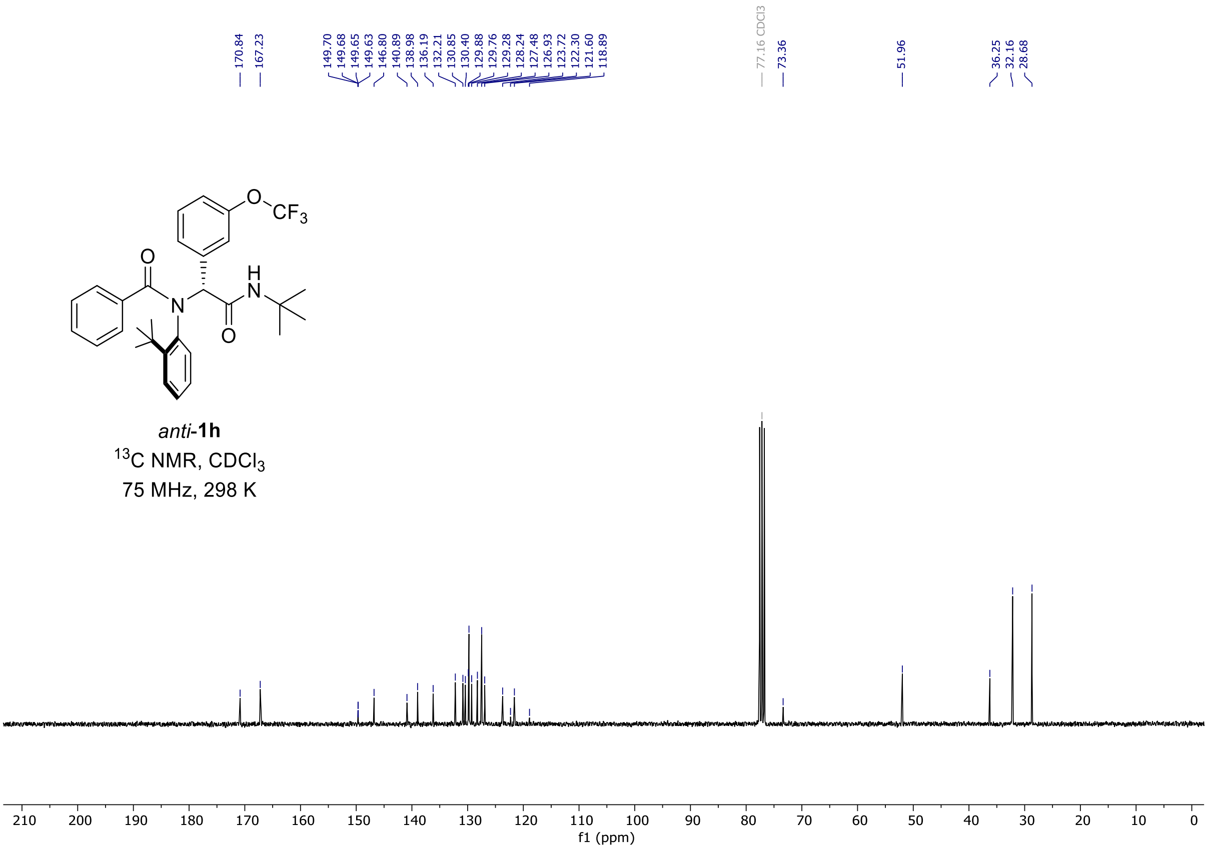

anti-**1h**
^{13}C NMR, $CDCl_3$
75 MHz, 298 K

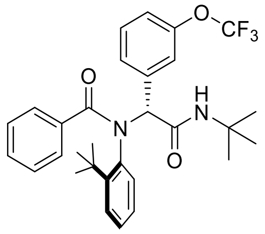

anti-**1h**
^{19}F NMR, $CDCl_3$
282 MHz, 298 K

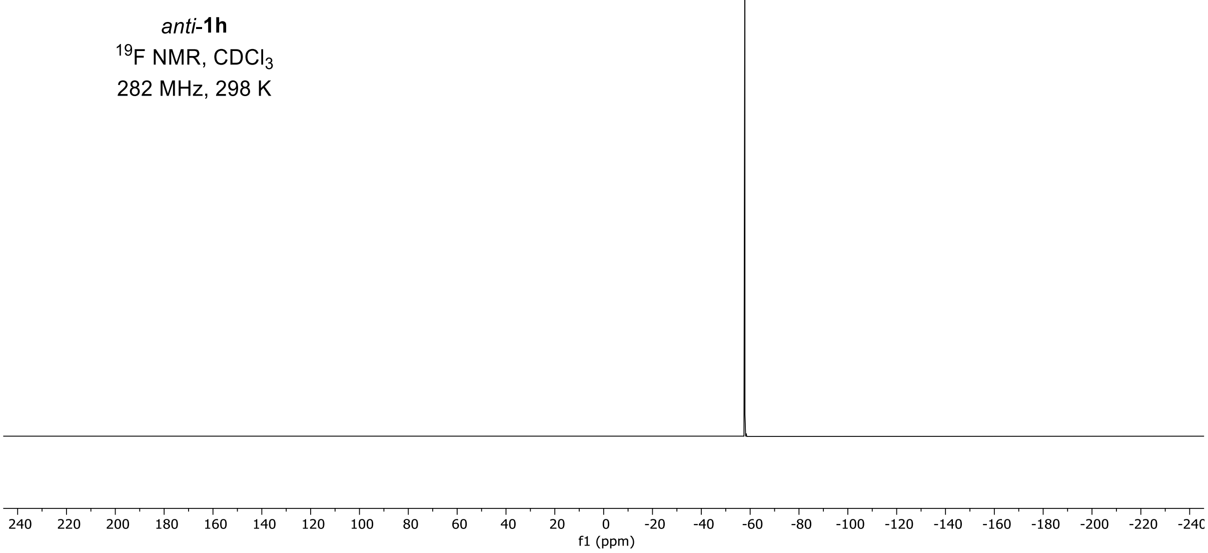

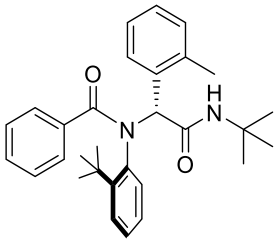

anti-**1i**
^{1}H NMR, $CDCl_3$
300 MHz, 298 K

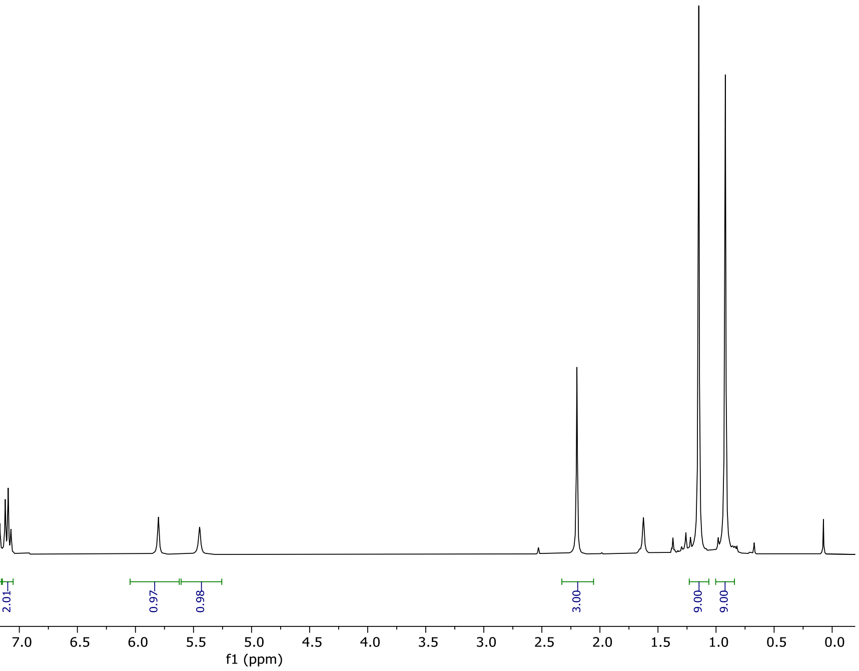

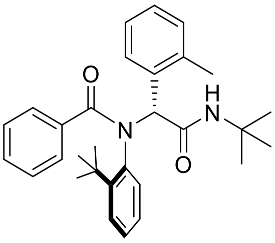

anti-**1i**
^{13}C NMR, $CDCl_3$
75 MHz, 298 K

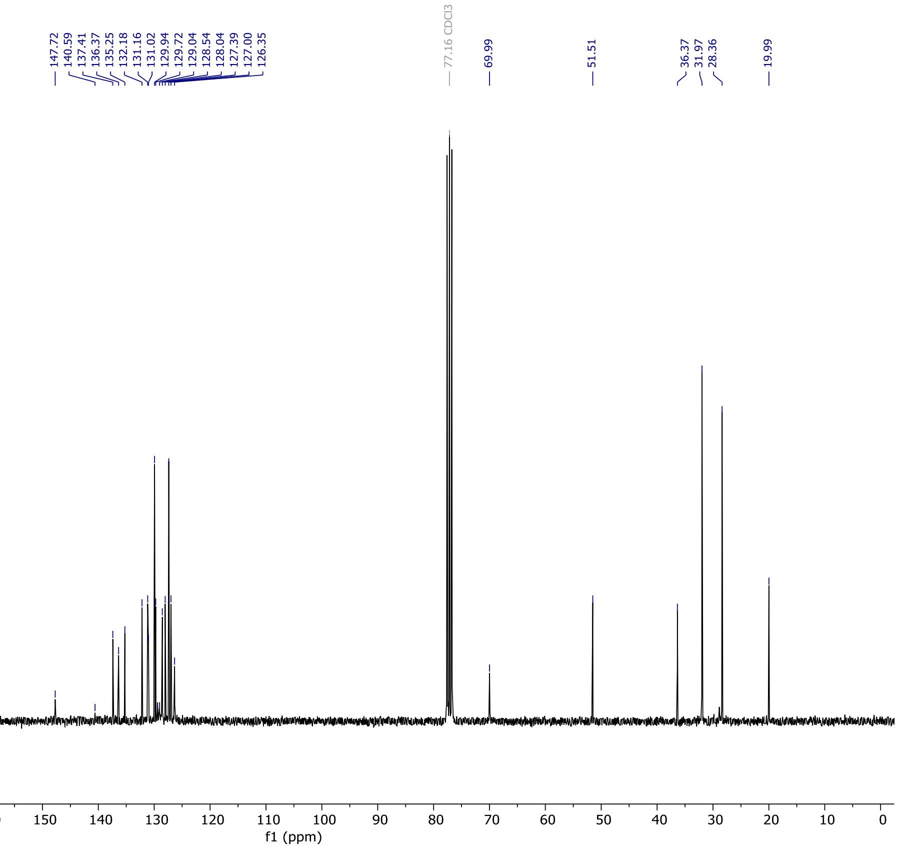

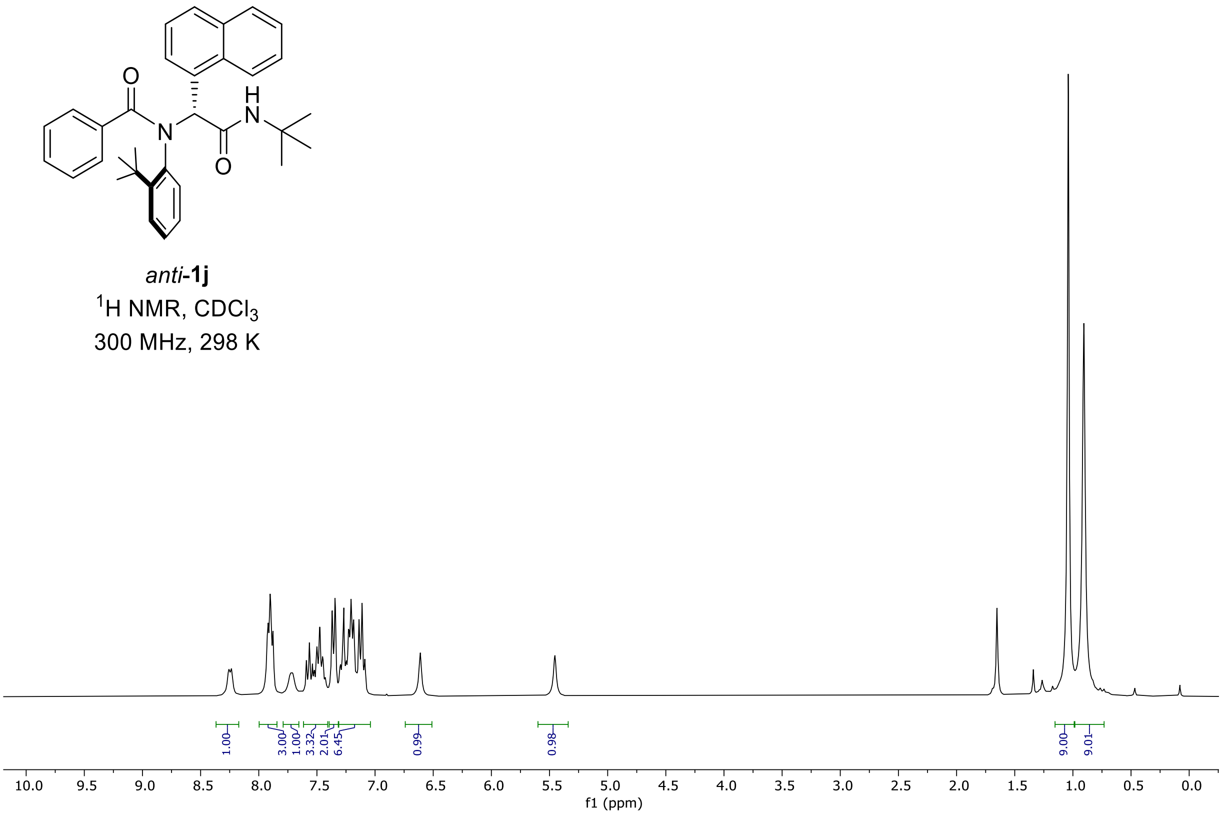

anti-**1j**
^{1}H NMR, $CDCl_3$
300 MHz, 298 K

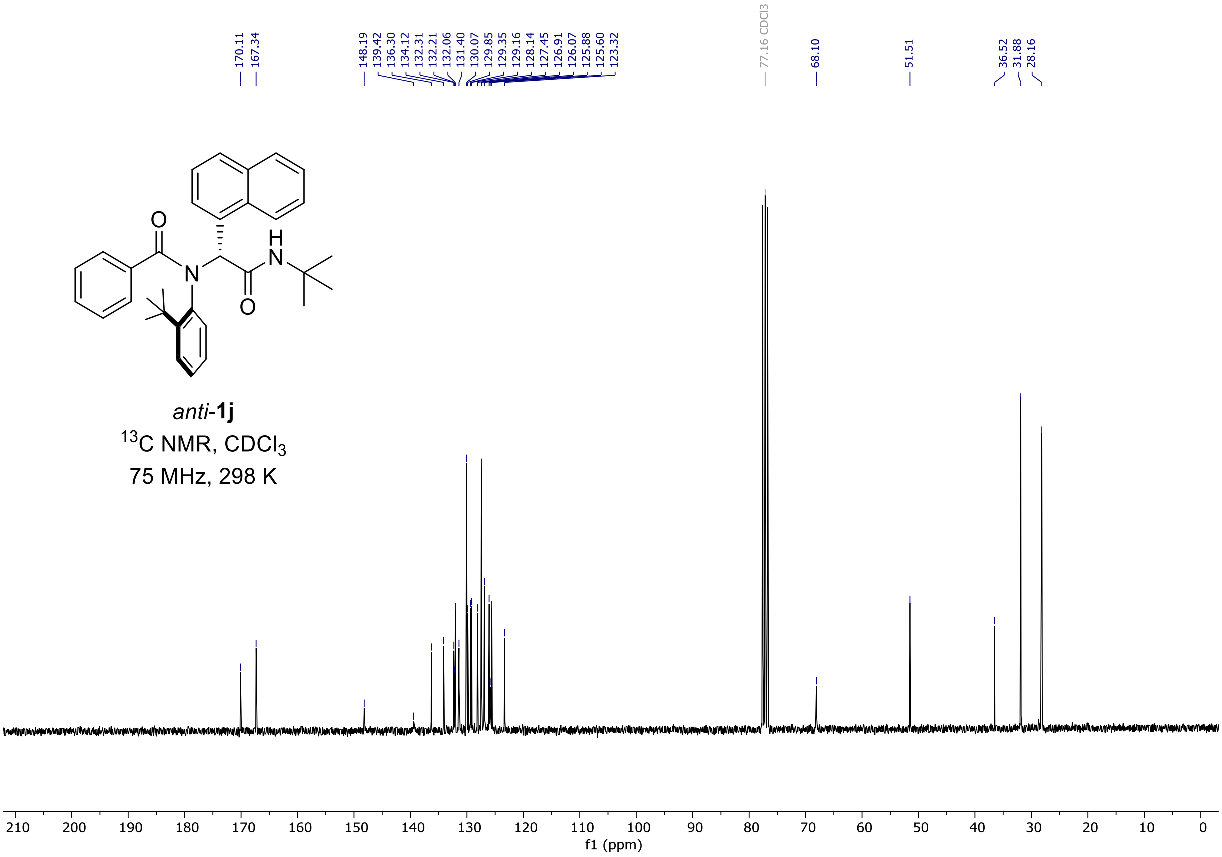

anti-**1j**
^{13}C NMR, $CDCl_3$
75 MHz, 298 K

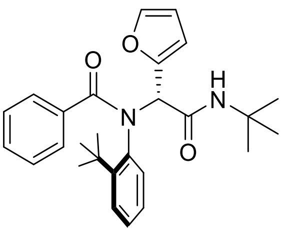

anti-**1k**
^{1}H NMR, $CDCl_3$
300 MHz, 298 K

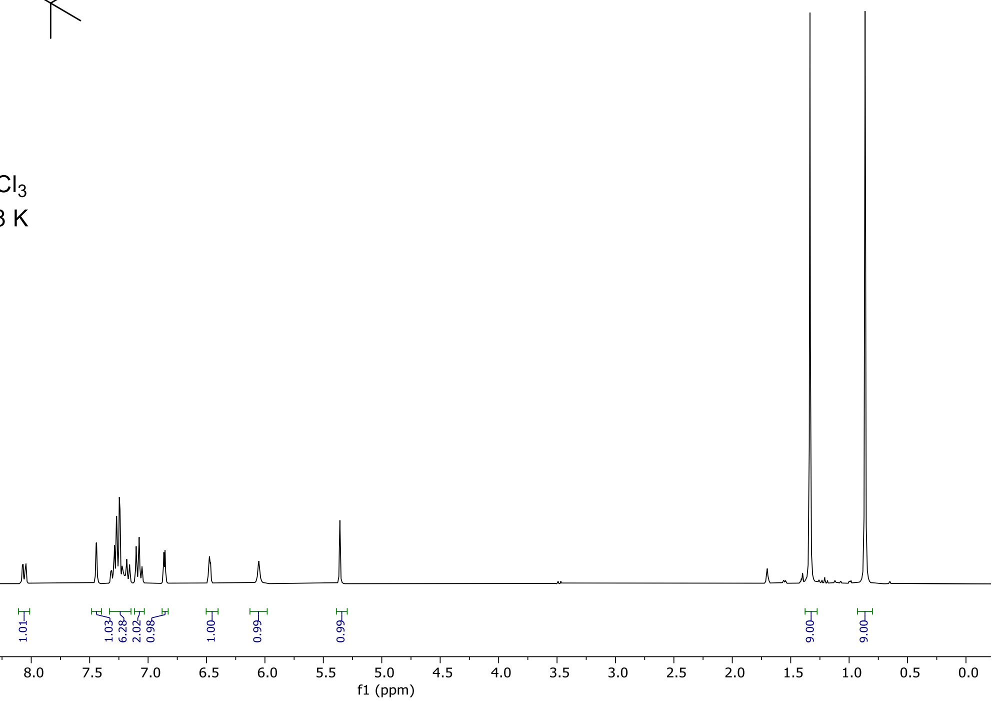

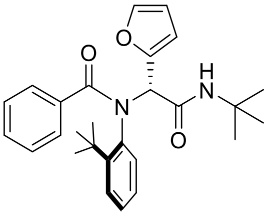

anti-**1k**
^{13}C NMR, $CDCl_3$
75 MHz, 298 K

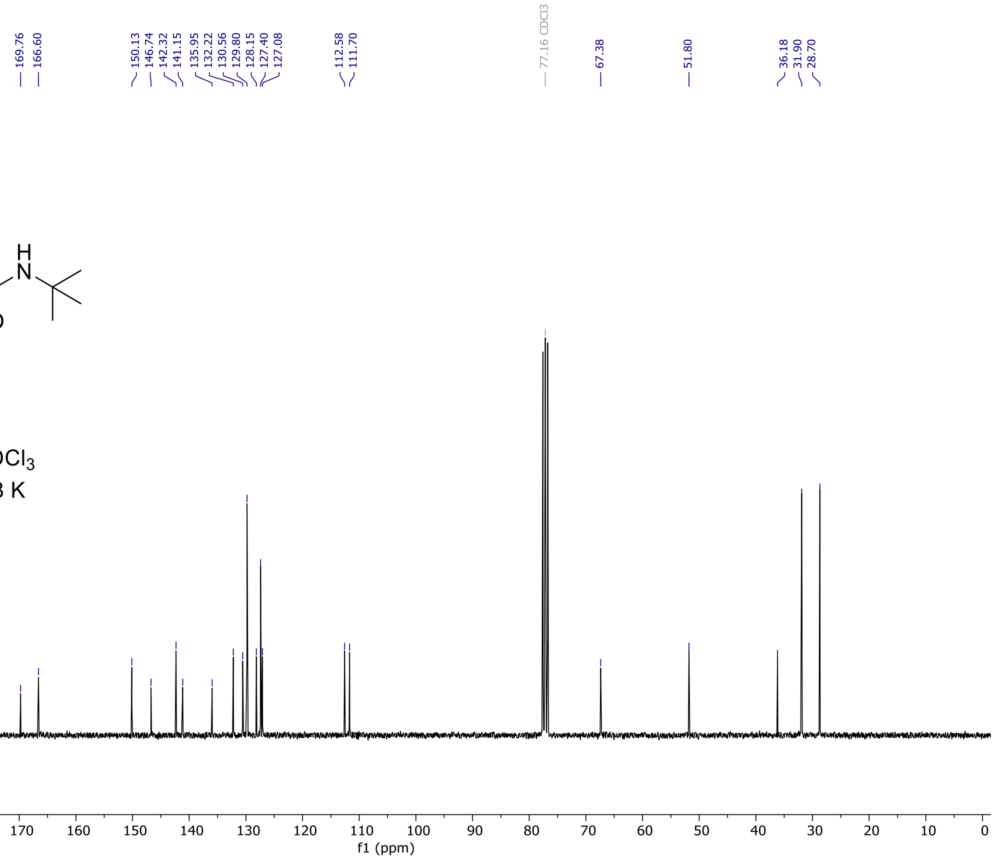

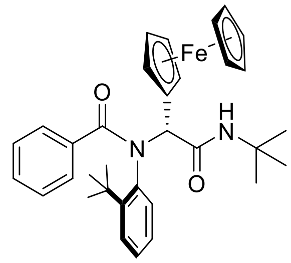

anti-**1l**
^{1}H NMR, $CDCl_3$
300 MHz, 298 K

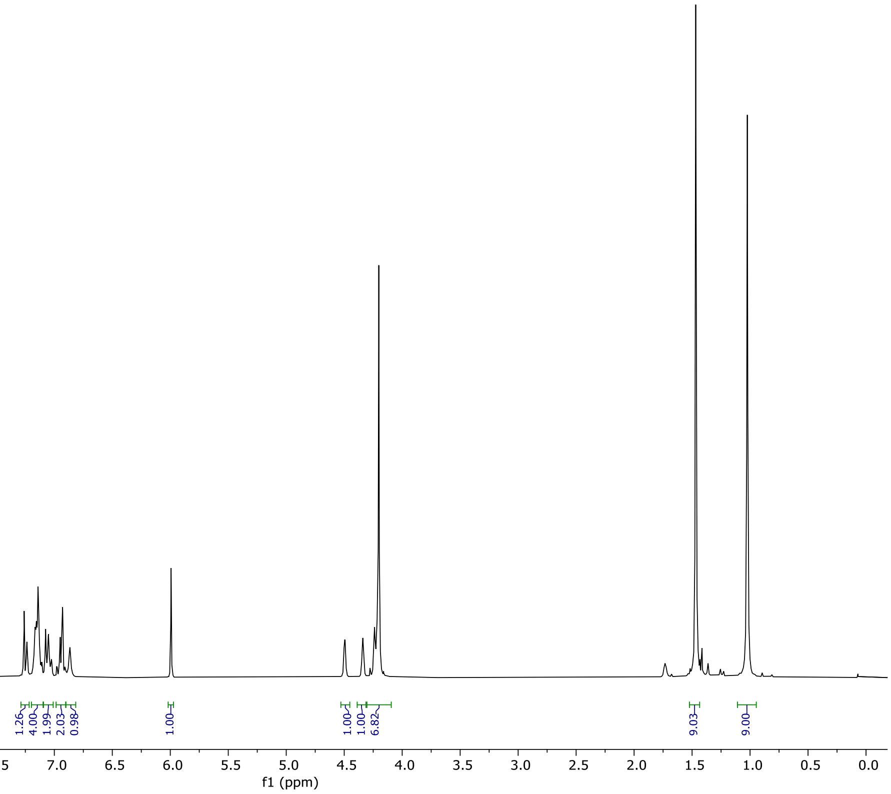

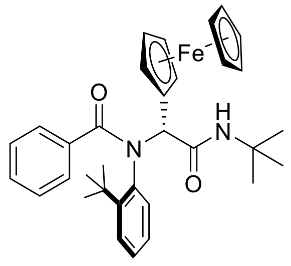

anti-**1l**
^{13}C NMR, $CDCl_3$
75 MHz, 298 K

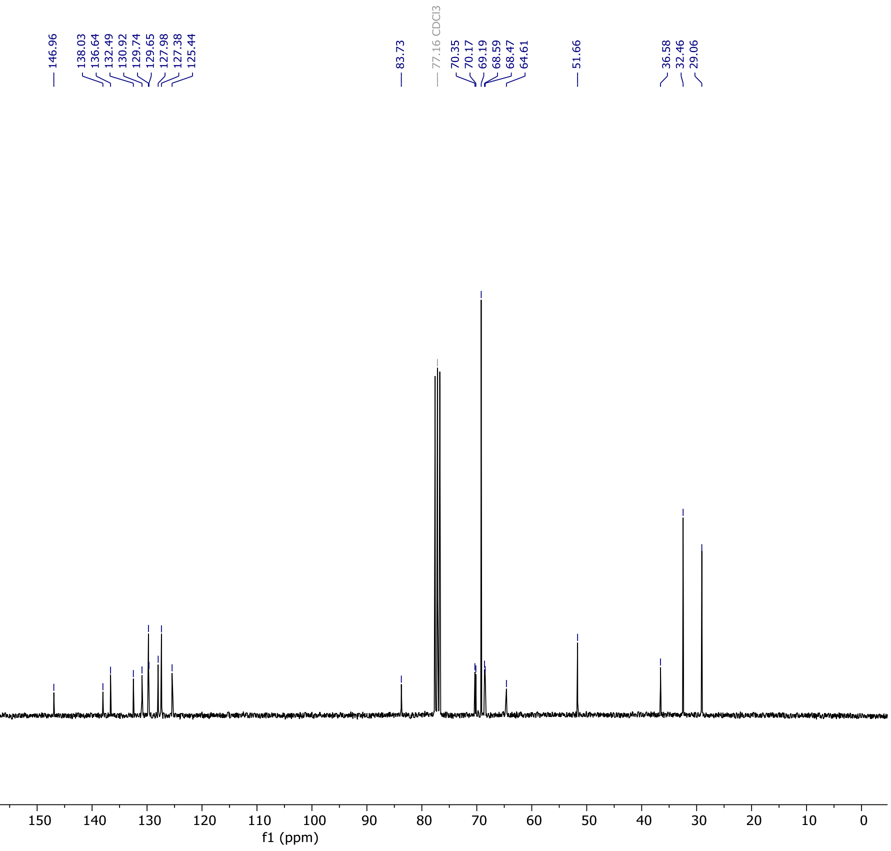

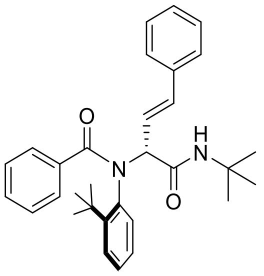

anti-**1m**
^{1}H NMR, $CDCl_3$
300 MHz, 298 K

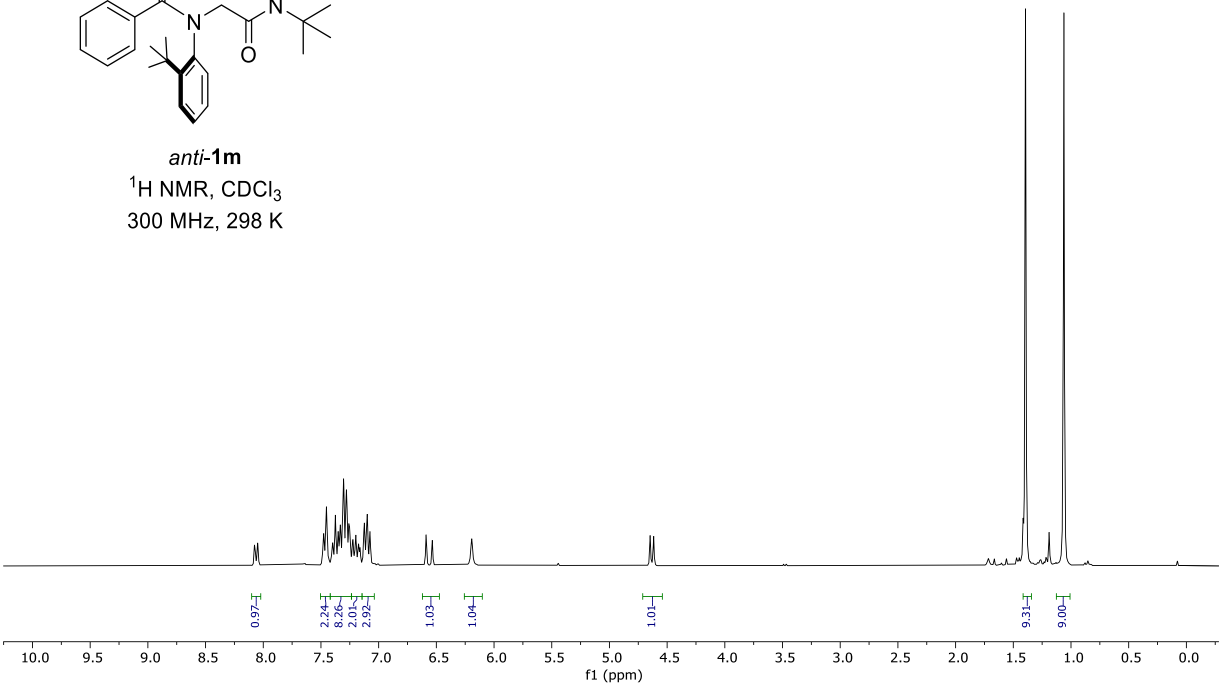

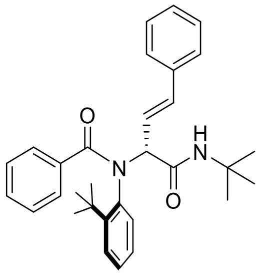

anti-**1m**
^{13}C NMR, $CDCl_3$
75 MHz, 298 K

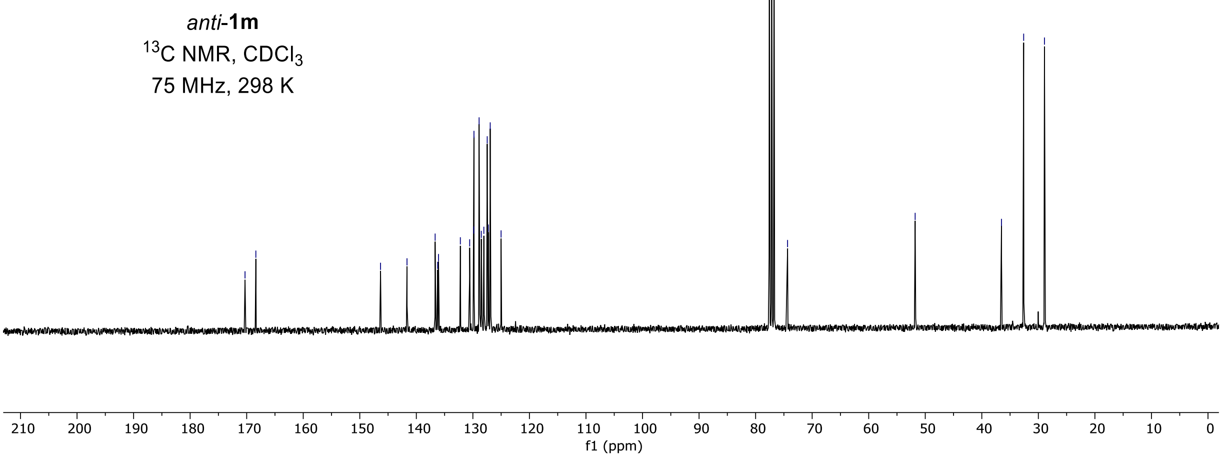

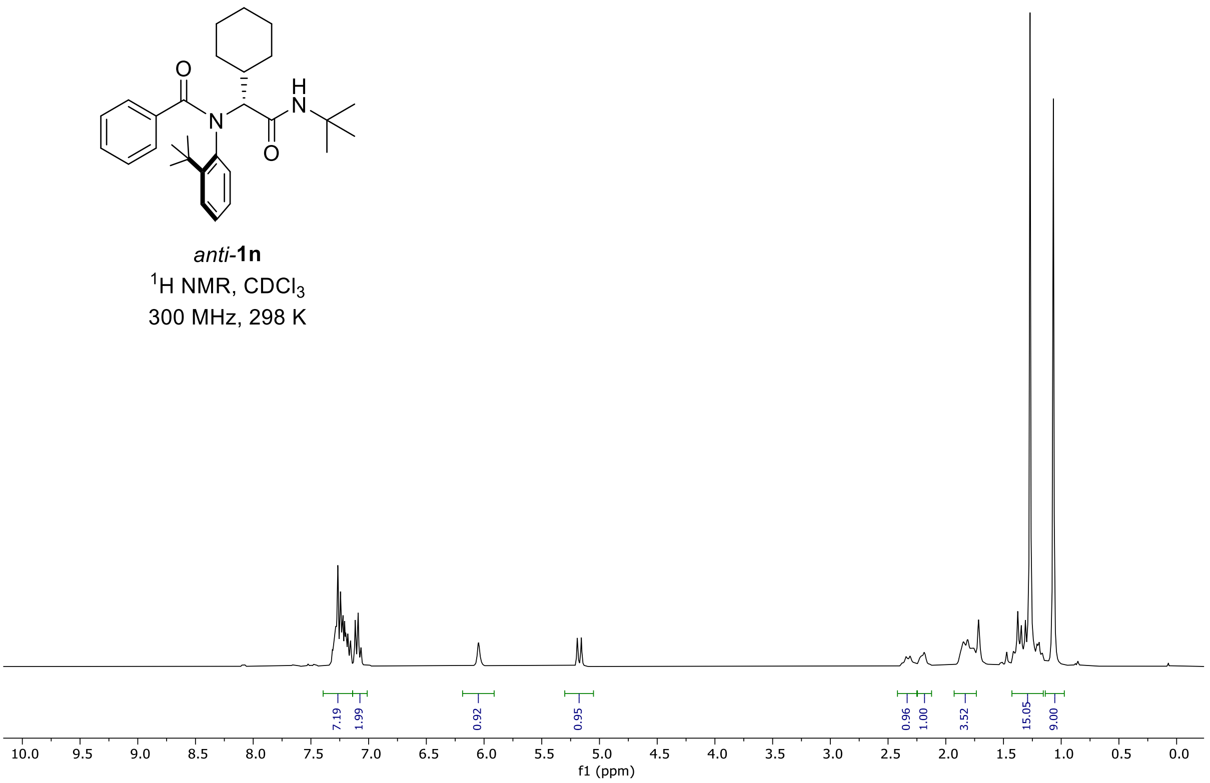

anti-1n
¹H NMR, CDCl3
300 MHz, 298 K
7.19
1.99
0.92
0.95
0.96
1.00
3.52
15.05
9.00
f1 (ppm)

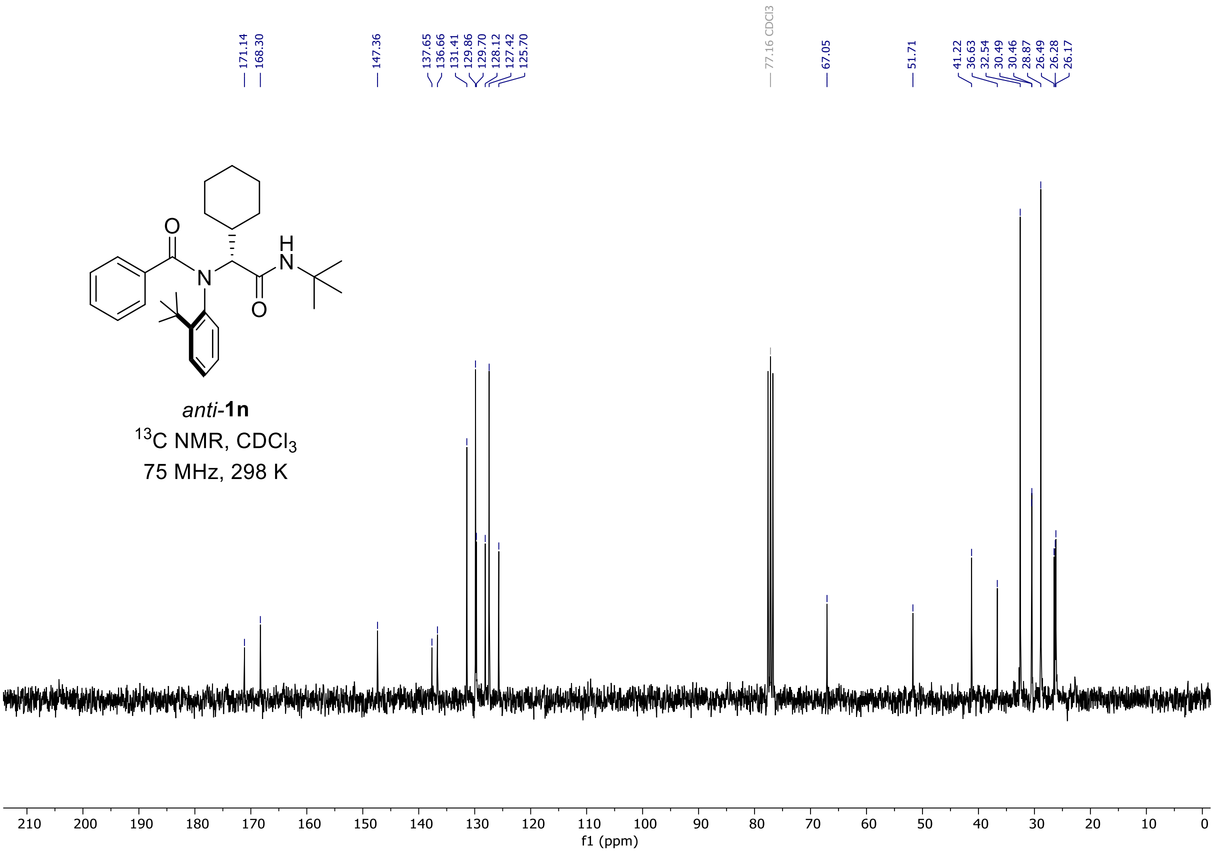

171.14
168.30
147.36
137.65
136.66
131.41
129.86
129.70
128.12
127.42
125.70
77.16 CDCl3
67.05
51.71
41.22
36.63
32.54
30.49
30.46
28.87
26.49
26.28
26.17
anti-1n
¹³C NMR, CDCl3
75 MHz, 298 K
f1 (ppm)

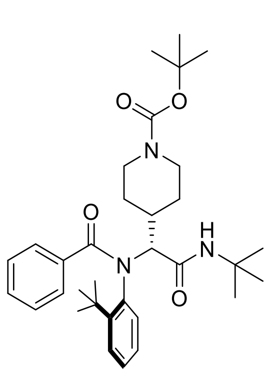

anti-**1o**
^{1}H NMR, DMSO-d_6
400 MHz, 298 K

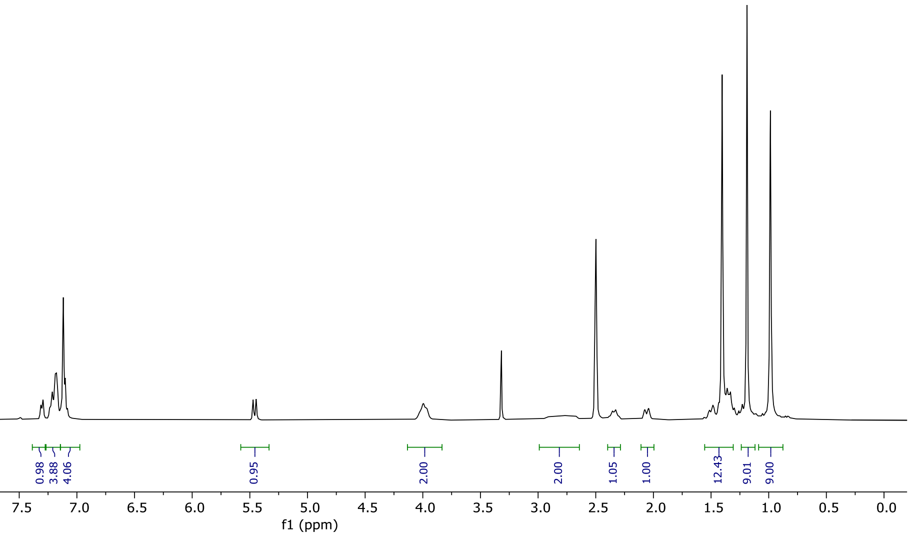

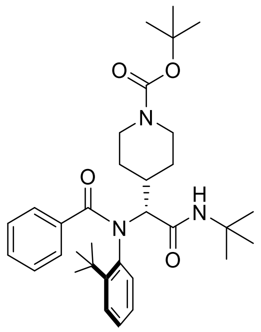

anti-**1o**
^{13}C NMR, DMSO-d_6
101 MHz, 298 K

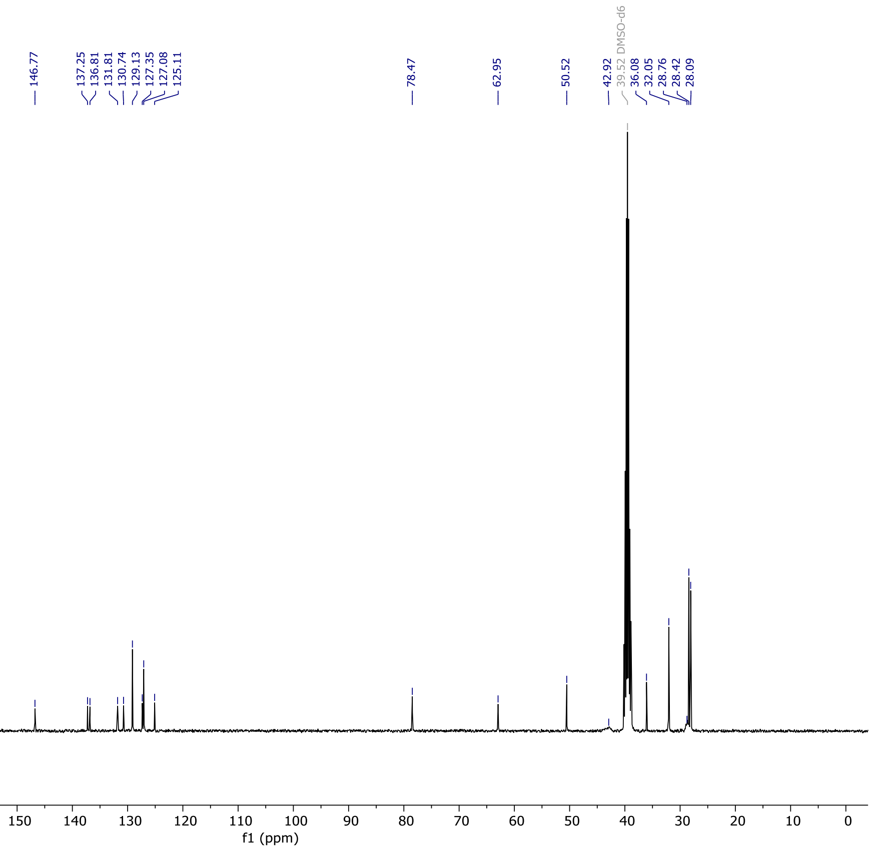

anti-**1p**
^{1}H NMR, $CDCl_3$
300 MHz, 298 K

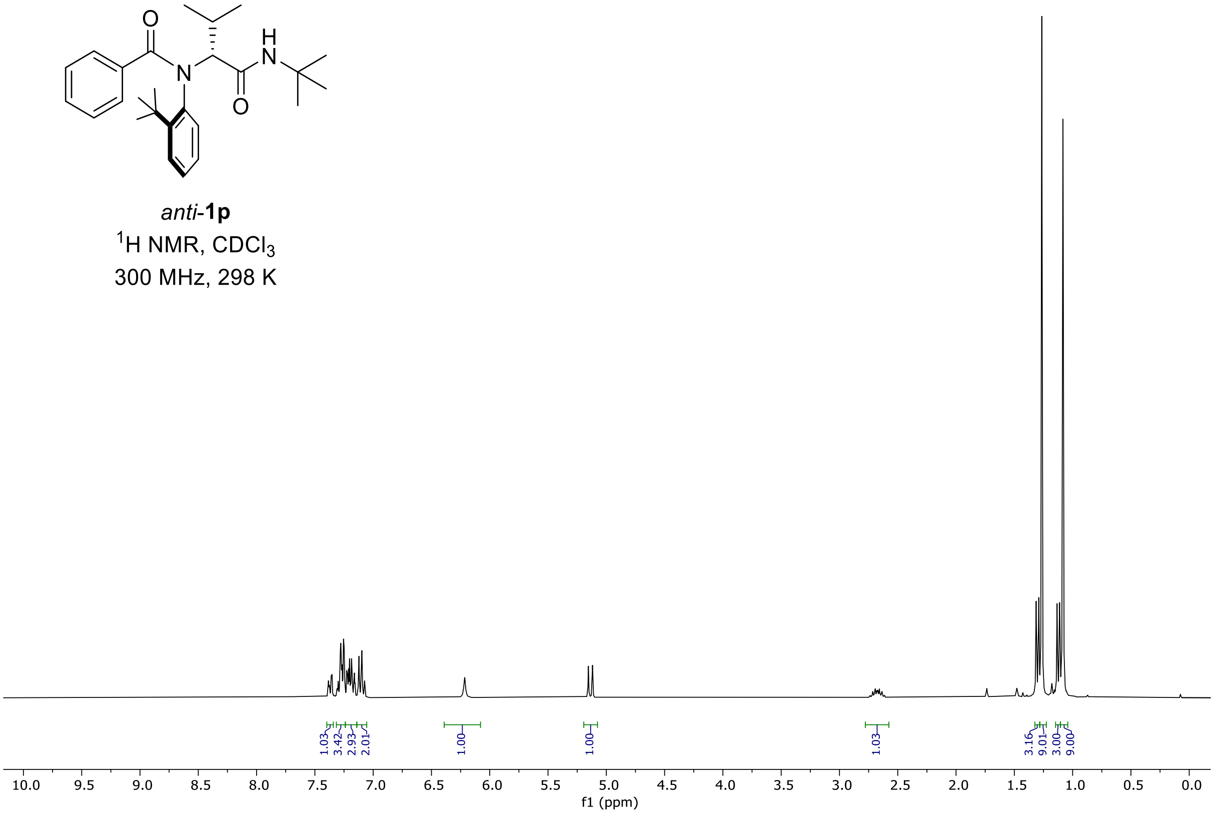

anti-**1p**
^{13}C NMR, $CDCl_3$
75 MHz, 298 K

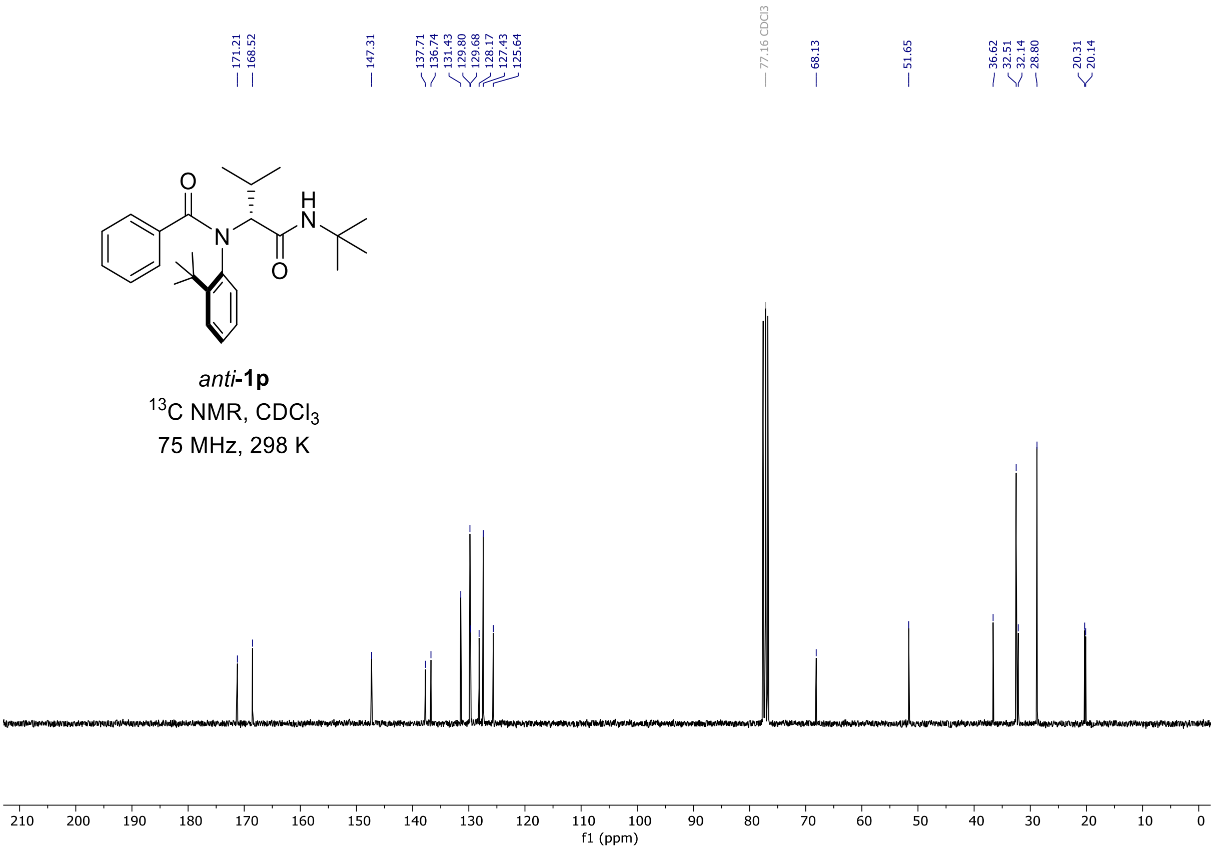

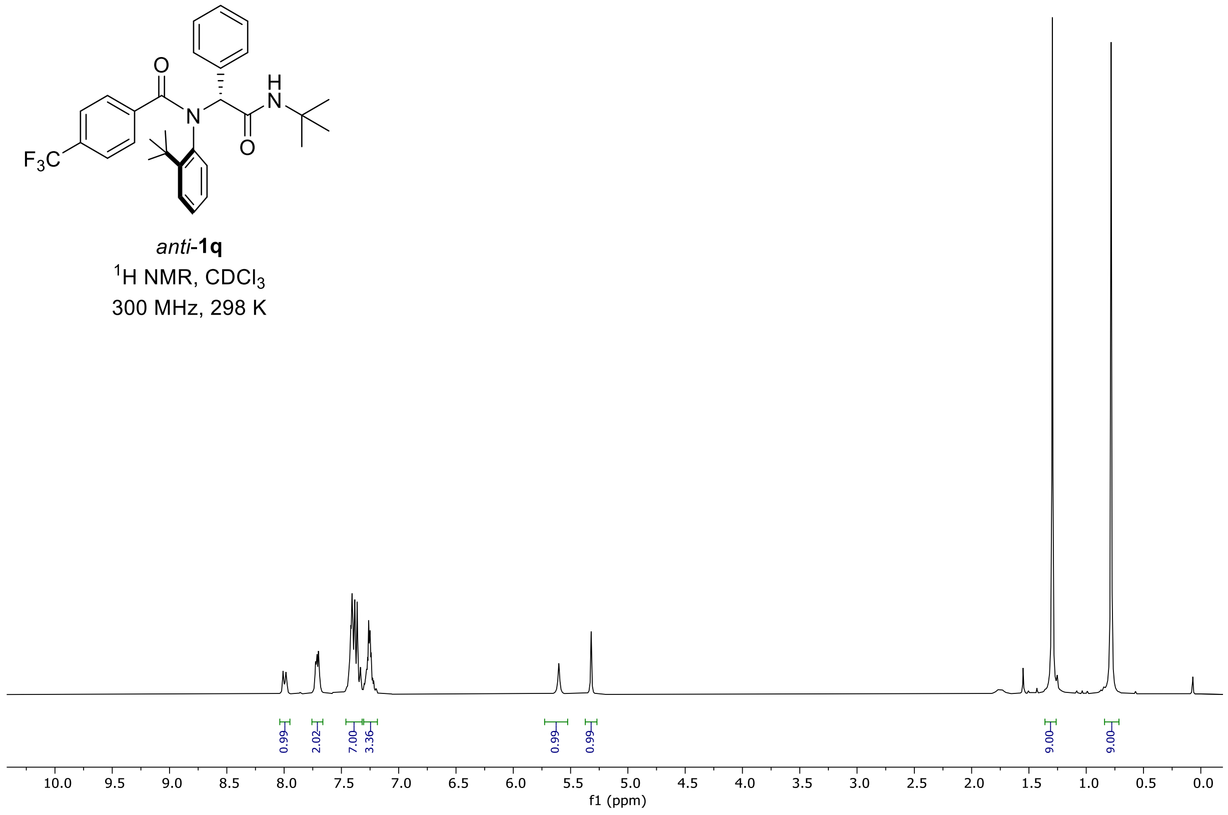
F3C
O
N
H
N
O
anti-1q
1H NMR, CDCl3
300 MHz, 298 K
0.99
2.02
7.00
3.36
0.99
0.99
9.00
9.00
10.0 9.5 9.0 8.5 8.0 7.5 7.0 6.5 6.0 5.5 5.0 4.5 4.0 3.5 3.0 2.5 2.0 1.5 1.0 0.5 0.0
f1 (ppm)

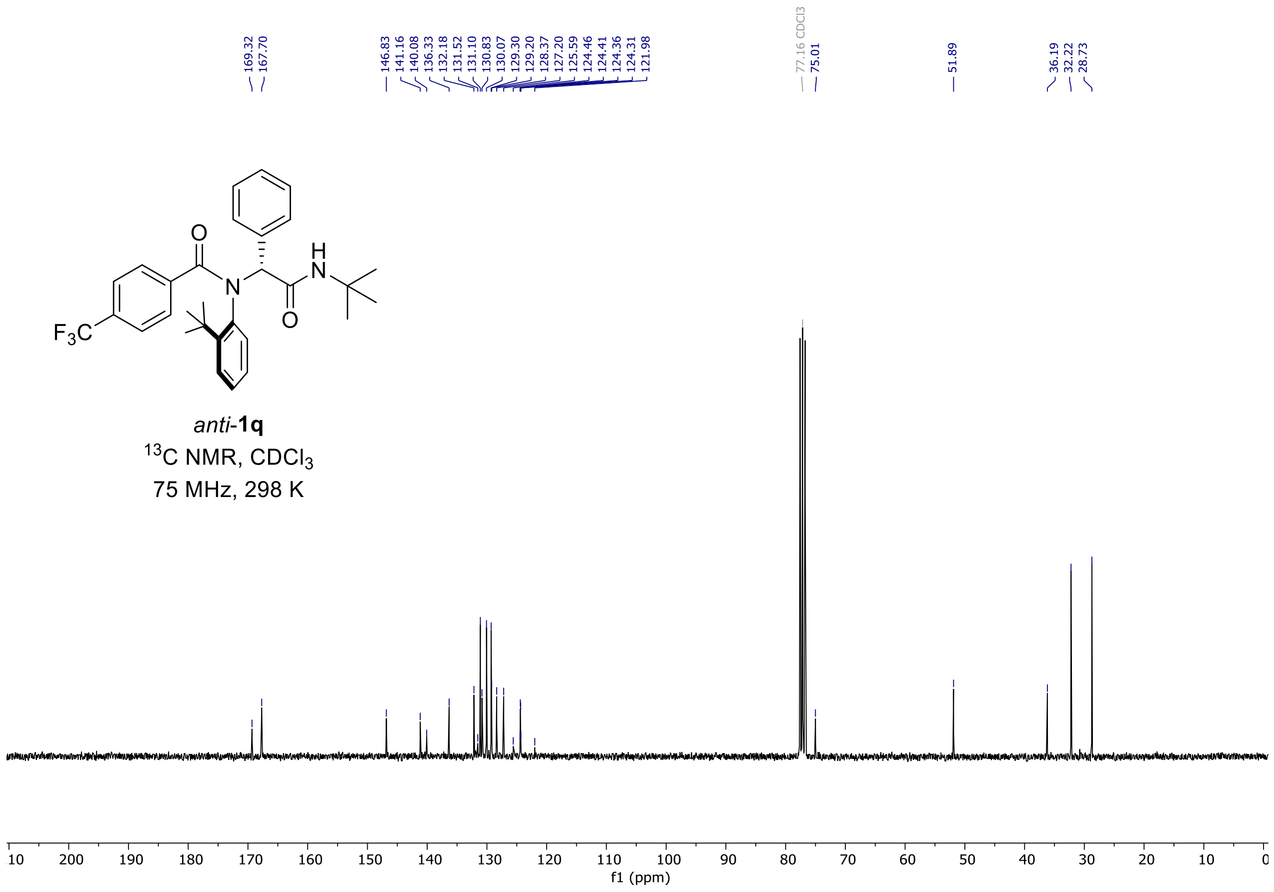
169.32
167.70
146.83
141.16
140.08
136.33
132.18
131.52
131.10
130.83
130.07
129.30
129.20
128.37
127.20
125.59
124.46
124.41
124.36
124.31
121.98
77.16 CDCl3
75.01
51.89
36.19
32.22
28.73
F3C
O
N
H
N
O
anti-1q
13C NMR, CDCl3
75 MHz, 298 K
10 200 190 180 170 160 150 140 130 120 110 100 90 80 70 60 50 40 30 20 10 0
f1 (ppm)

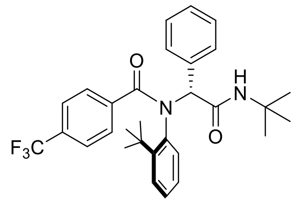

anti-**1q**
$^{19}F\{^{1}H\}$ NMR, $CDCl_3$
282 MHz, 298 K

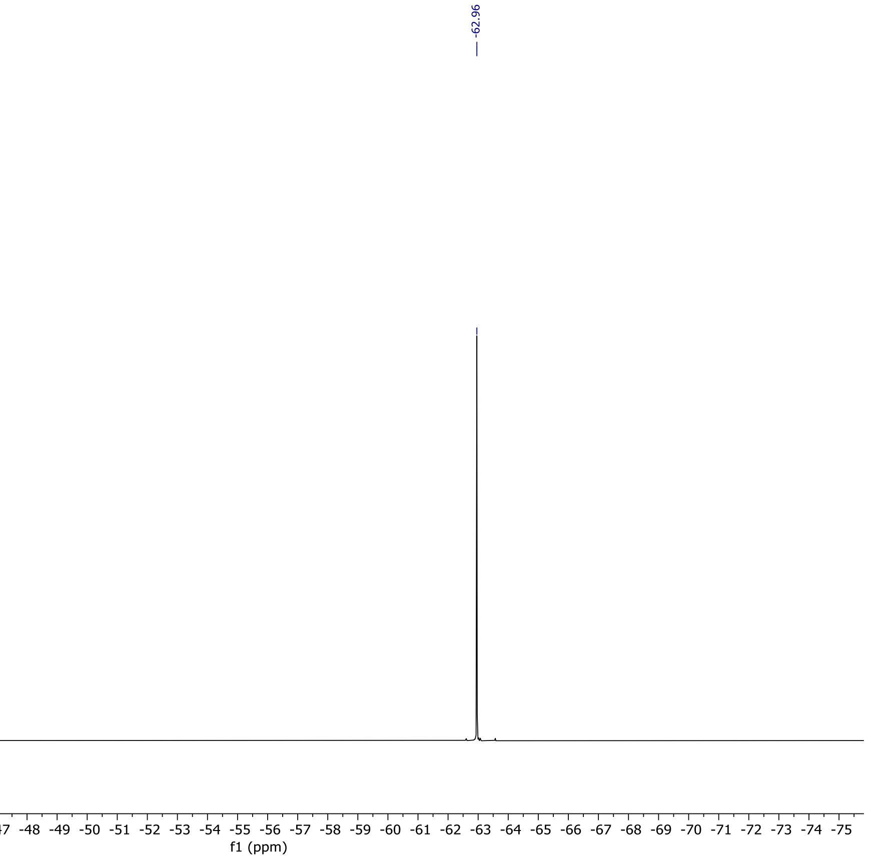

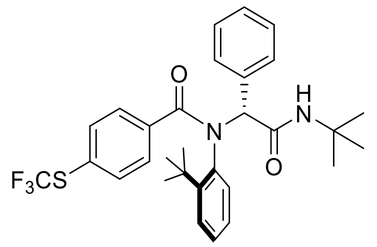

anti-**1r**
^{1}H NMR, $CDCl_3$
300 MHz, 298 K

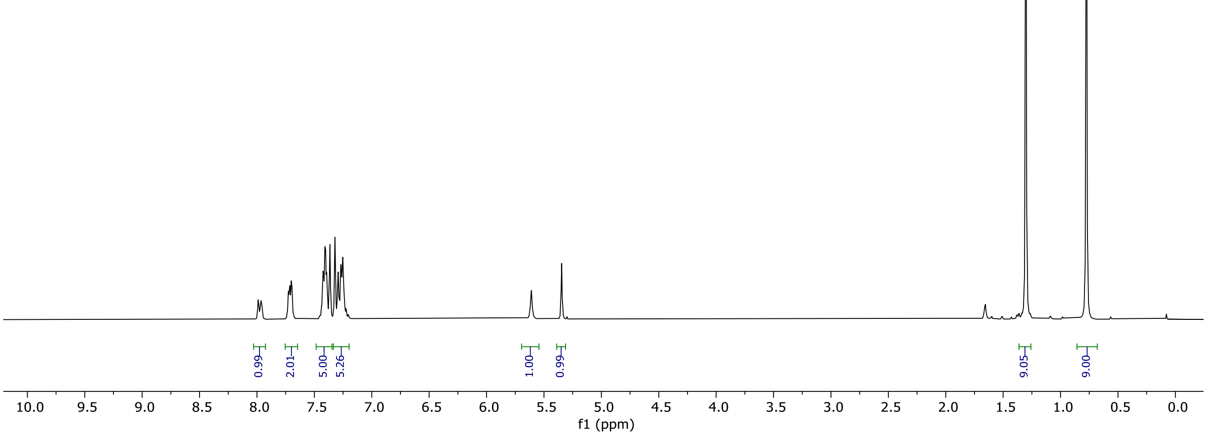

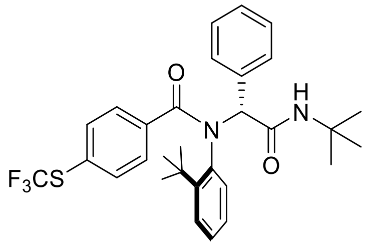

anti-**1r**
^{13}C NMR, $CDCl_3$
75 MHz, 298 K

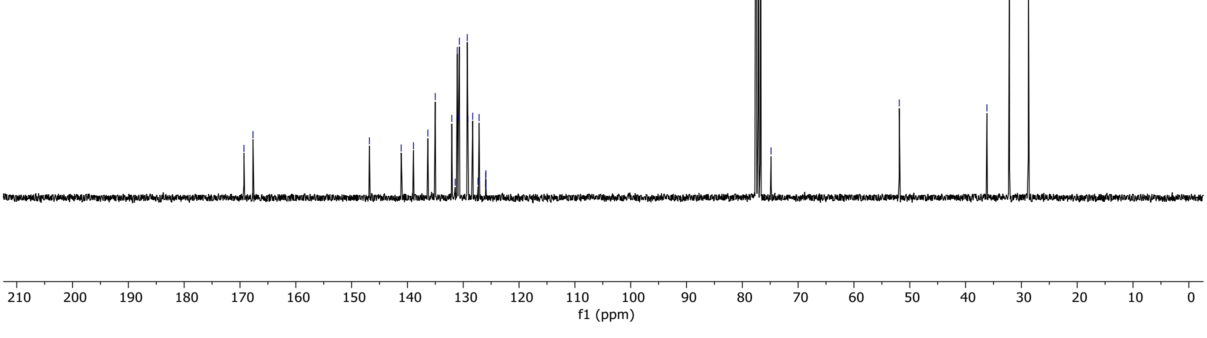

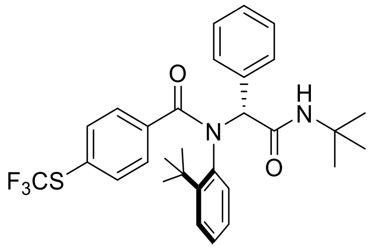

anti-**1r**
^{19}F NMR, $CDCl_3$
282 MHz, 298 K

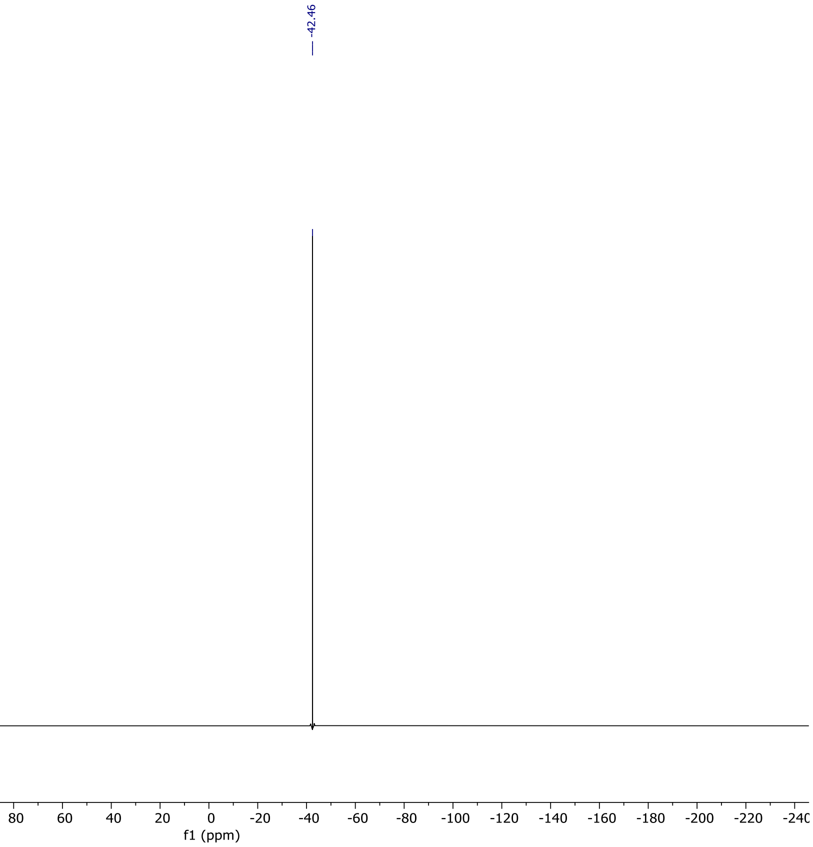

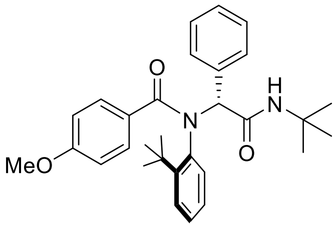

anti-**1s**
^{1}H NMR, $CDCl_3$
300 MHz, 298 K

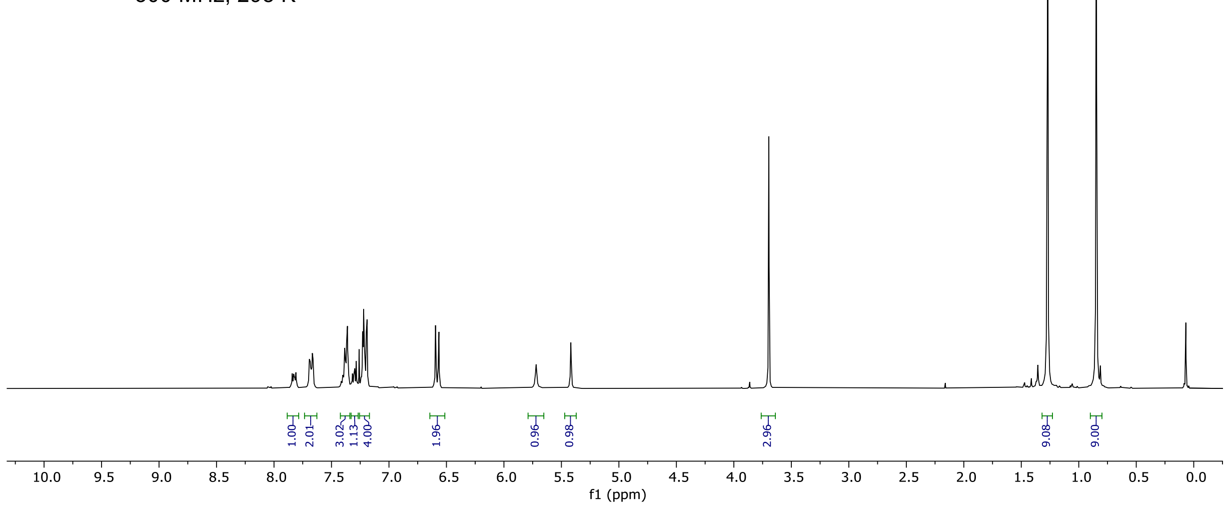

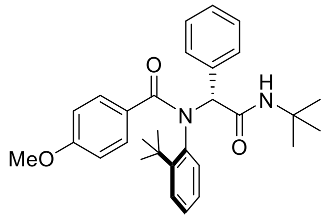

anti-**1s**
^{13}C NMR, $CDCl_3$
75 MHz, 298 K

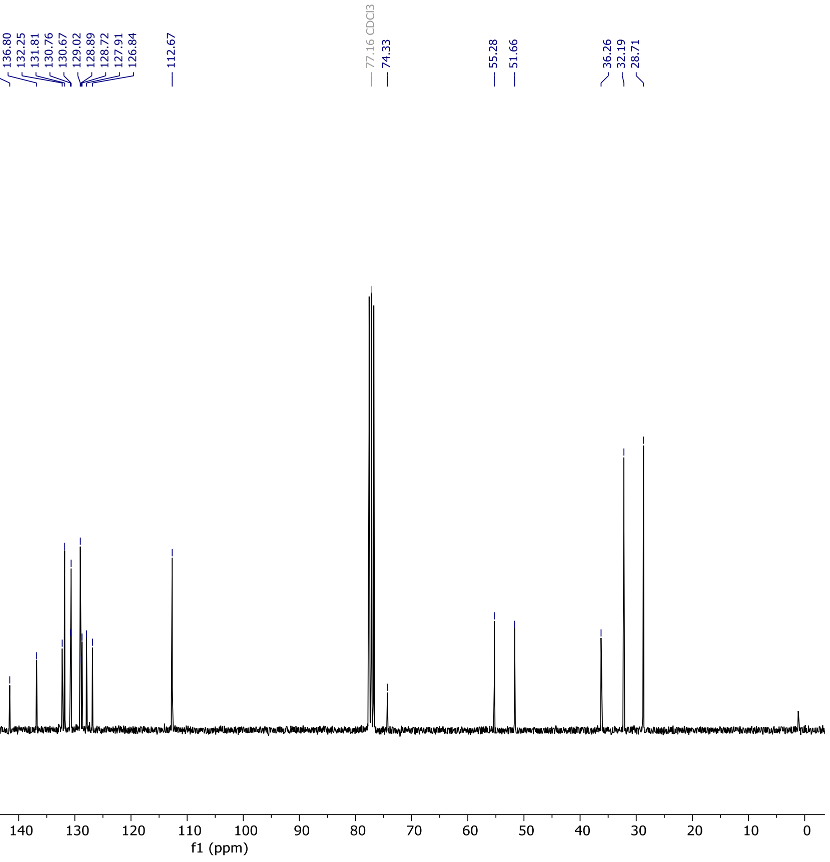

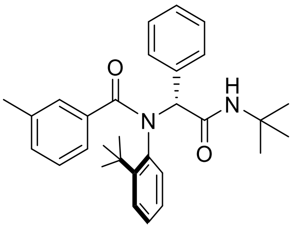

anti-**1t**
^{1}H NMR, $CDCl_3$
300 MHz, 298 K

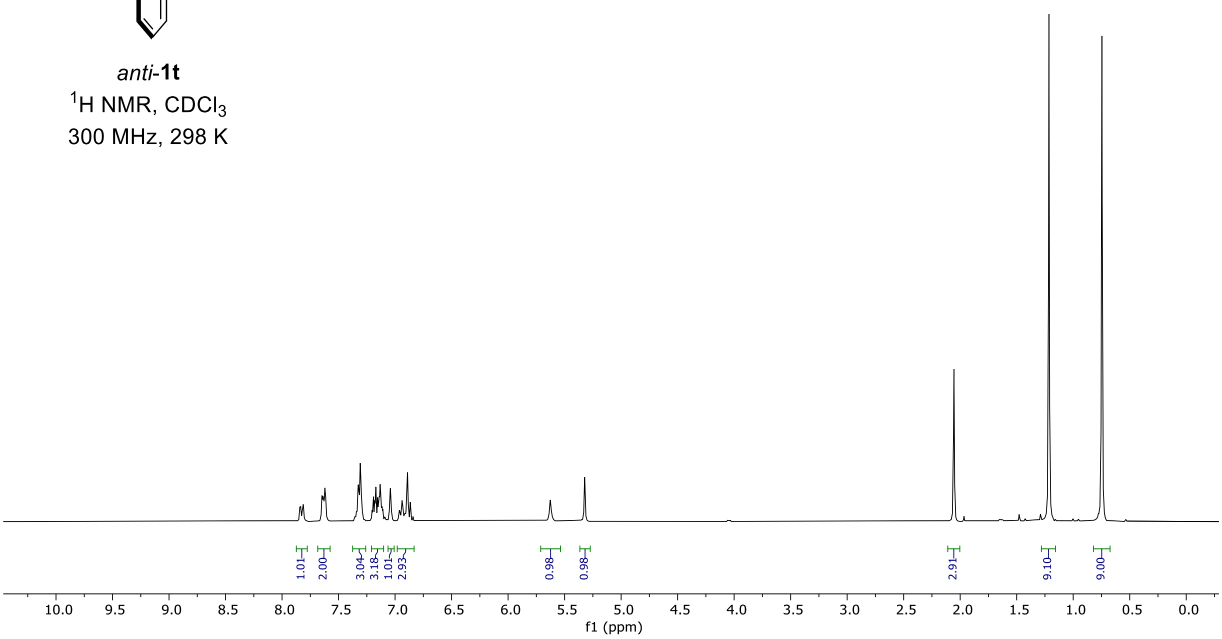

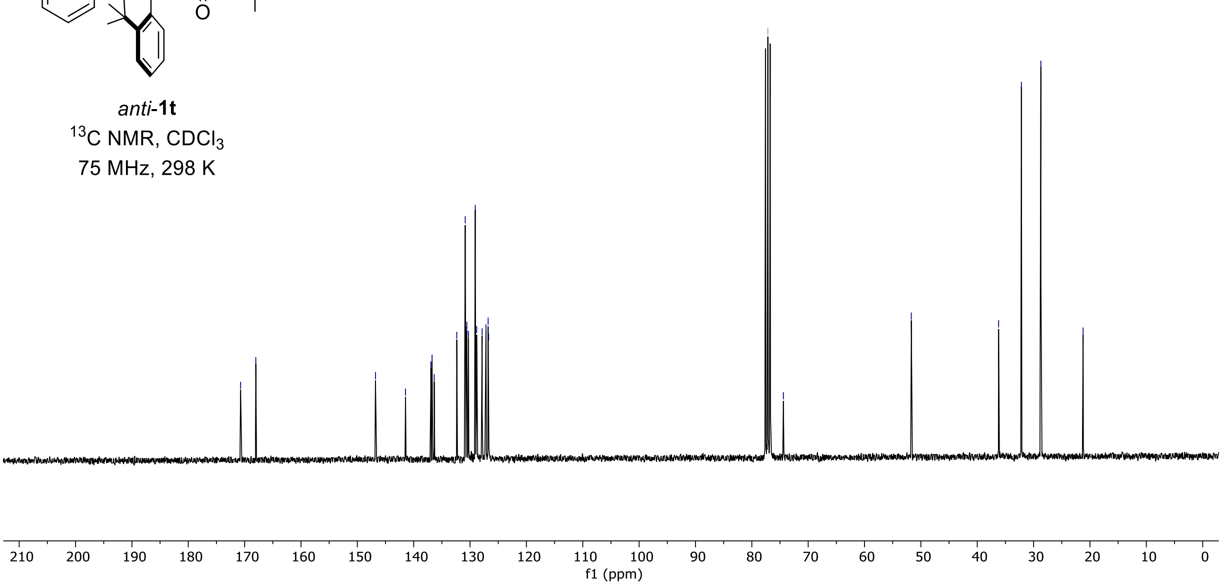

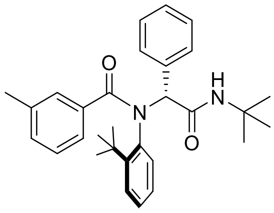

anti-**1t**
^{13}C NMR, $CDCl_3$
75 MHz, 298 K

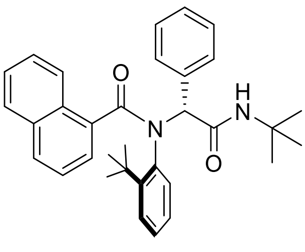

anti-**1u**
^{1}H NMR, $CDCl_3$
300 MHz, 298 K

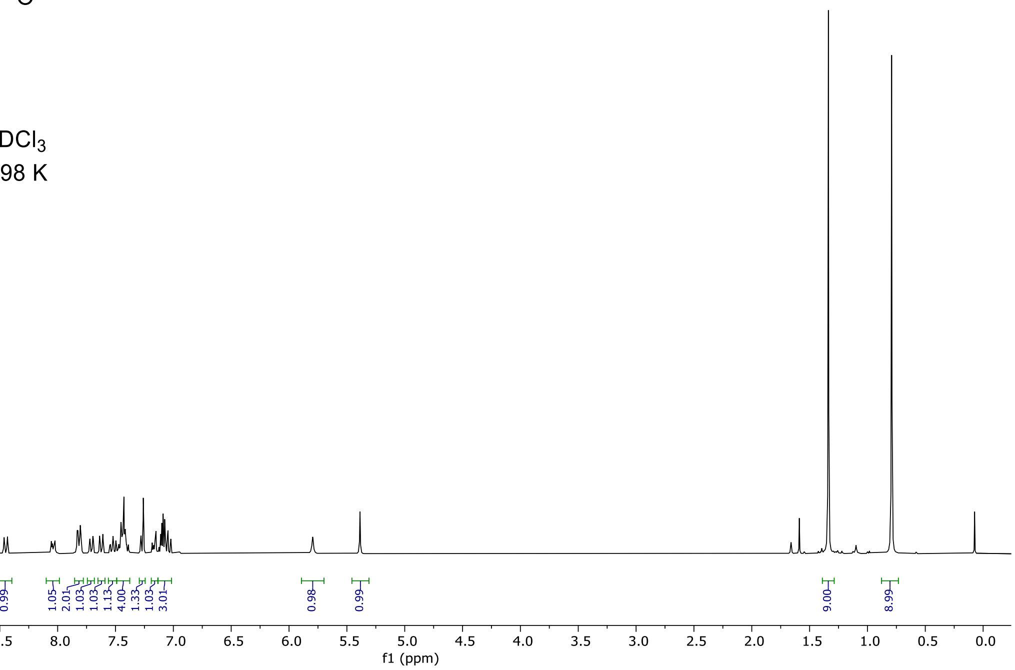

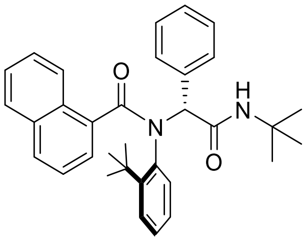

anti-**1u**
^{13}C NMR, $CDCl_3$
75 MHz, 298 K

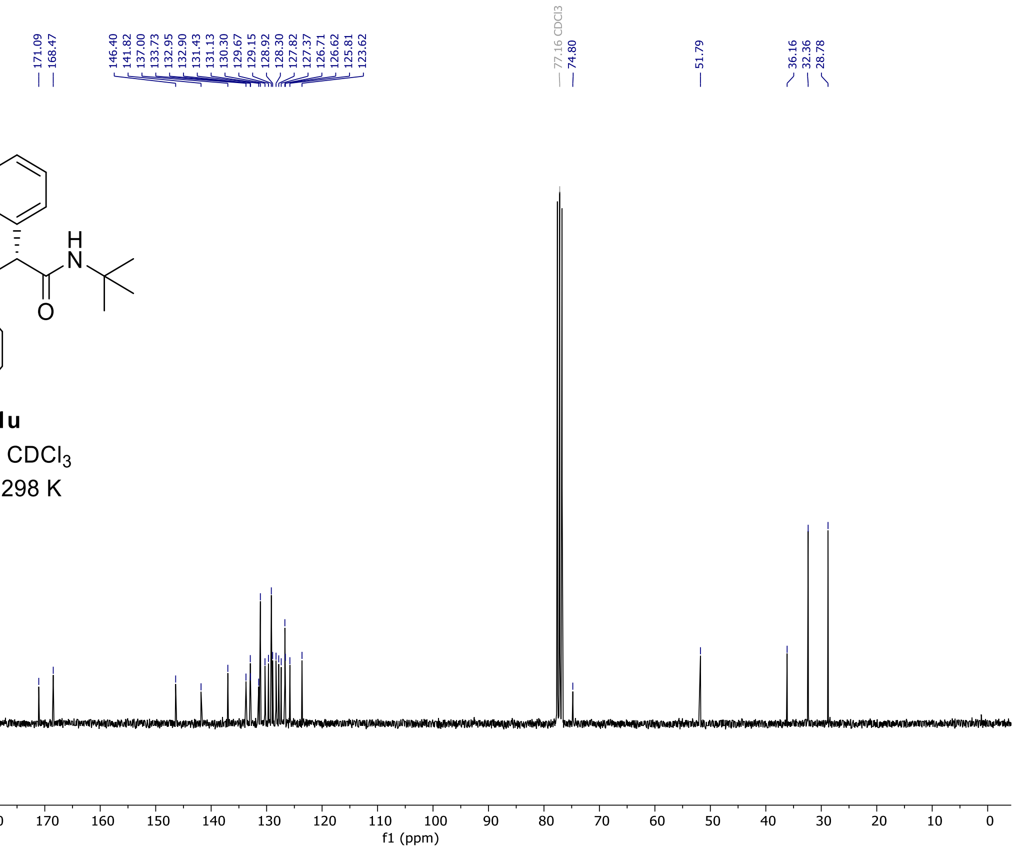

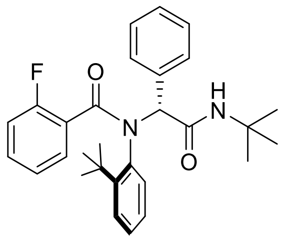

anti-**1v**
^{1}H NMR, $CDCl_3$
300 MHz, 298 K

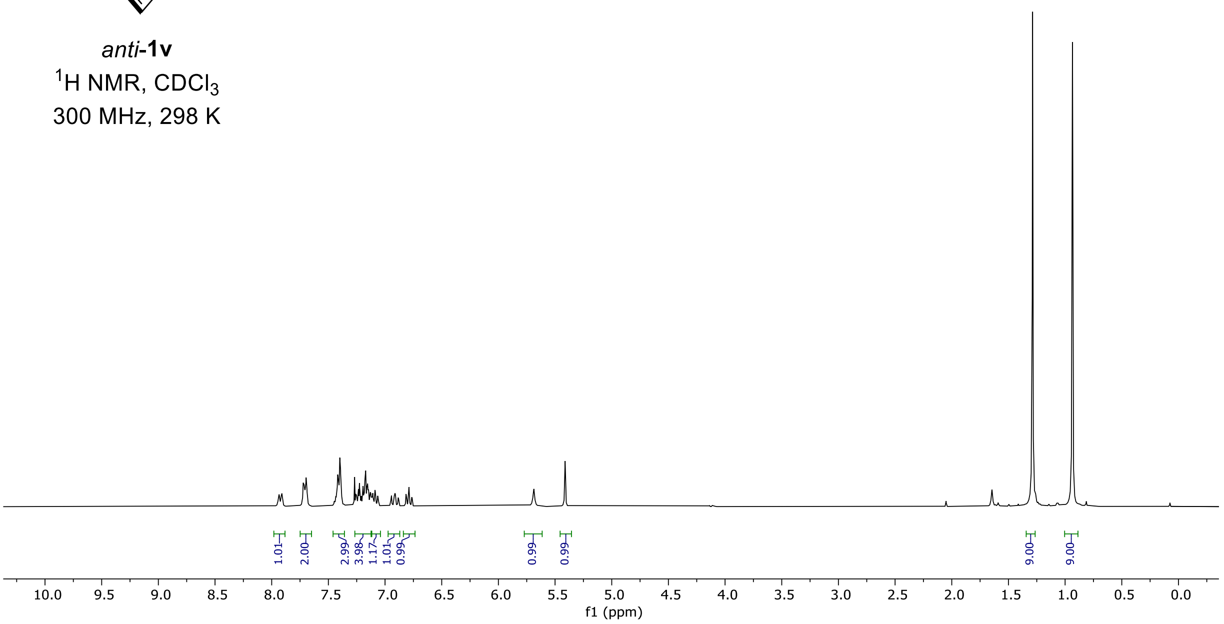

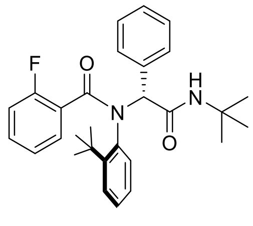

anti-**1v**
^{13}C NMR, $CDCl_3$
75 MHz, 298 K

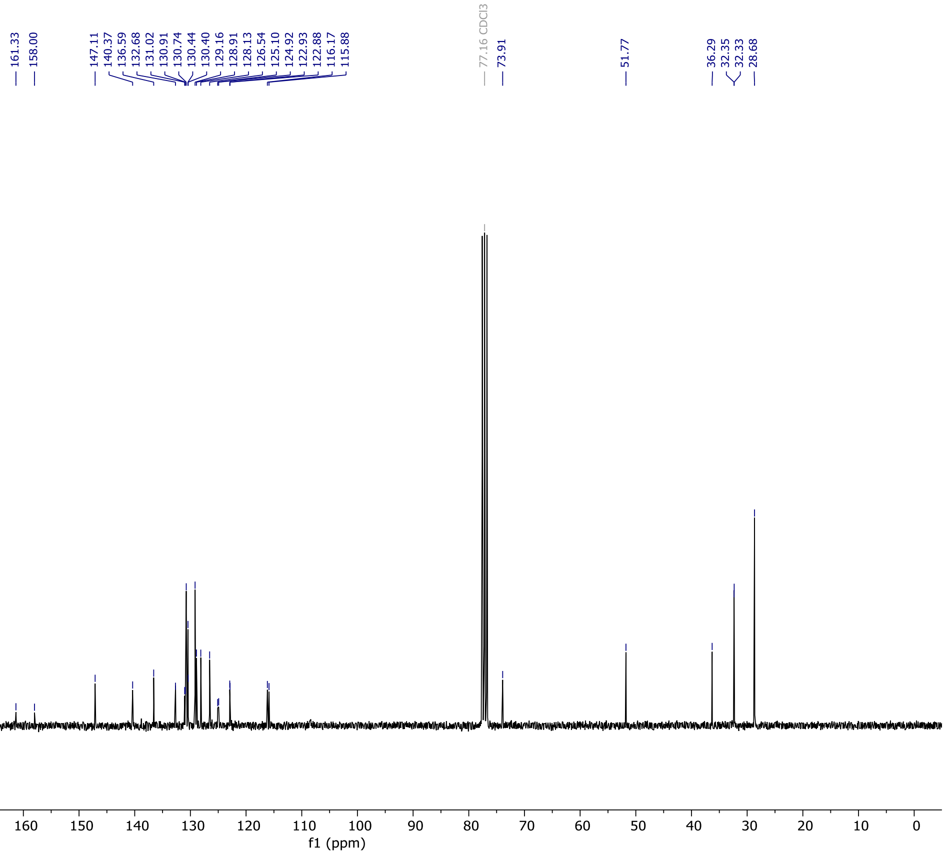

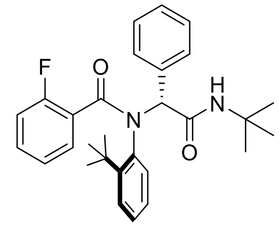

anti-**1v**
^{19}F NMR, $CDCl_3$
282 MHz, 298 K

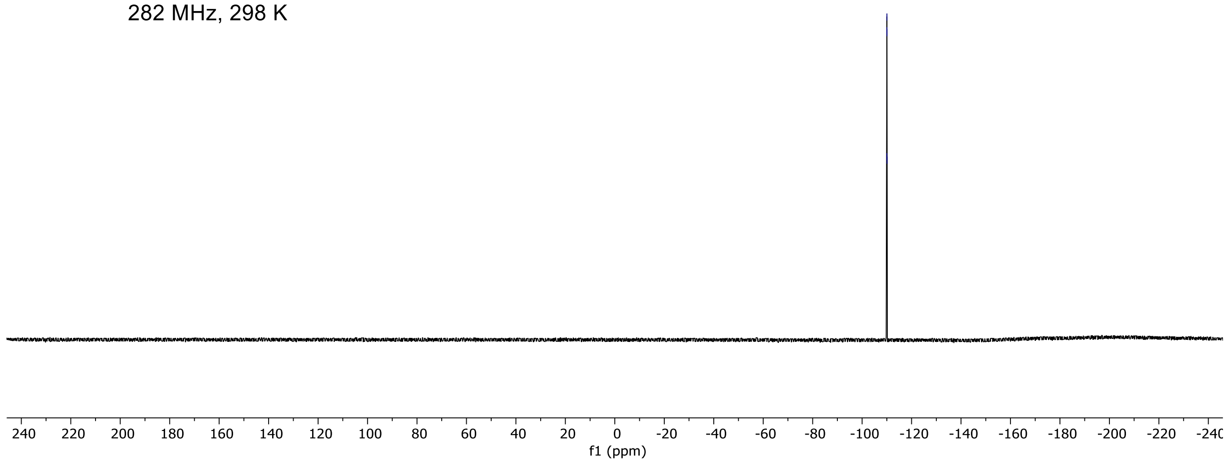

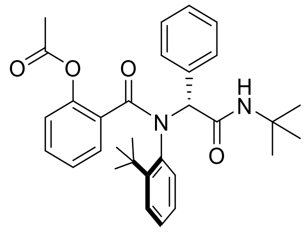

anti-**1w**
^{1}H NMR, $CDCl_3$
300 MHz, 298 K

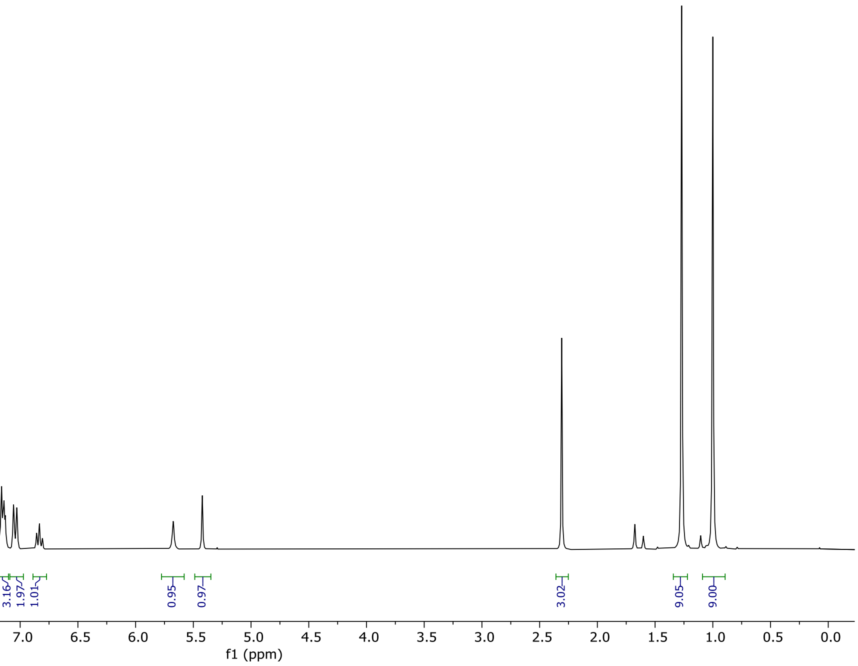

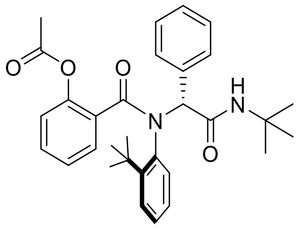

anti-**1w**
^{13}C NMR, $CDCl_3$
75 MHz, 298 K

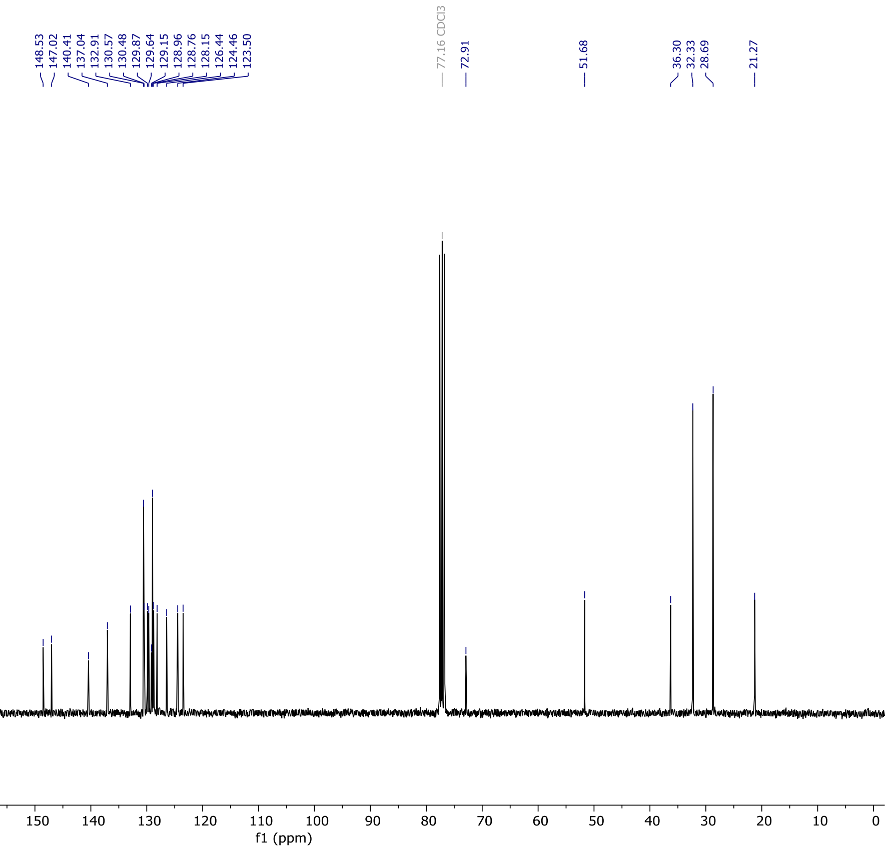

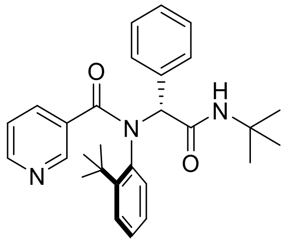

anti-**1x**
^{1}H NMR, $CDCl_3$
300 MHz, 298 K

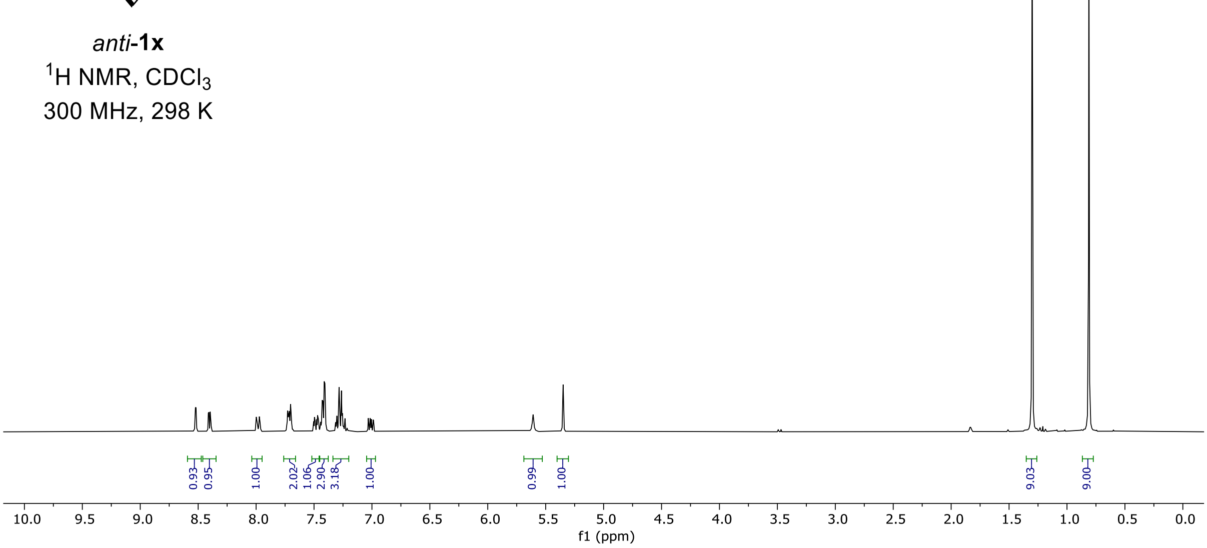

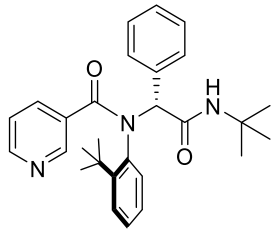

anti-**1x**
^{13}C NMR, $CDCl_3$
75 MHz, 298 K

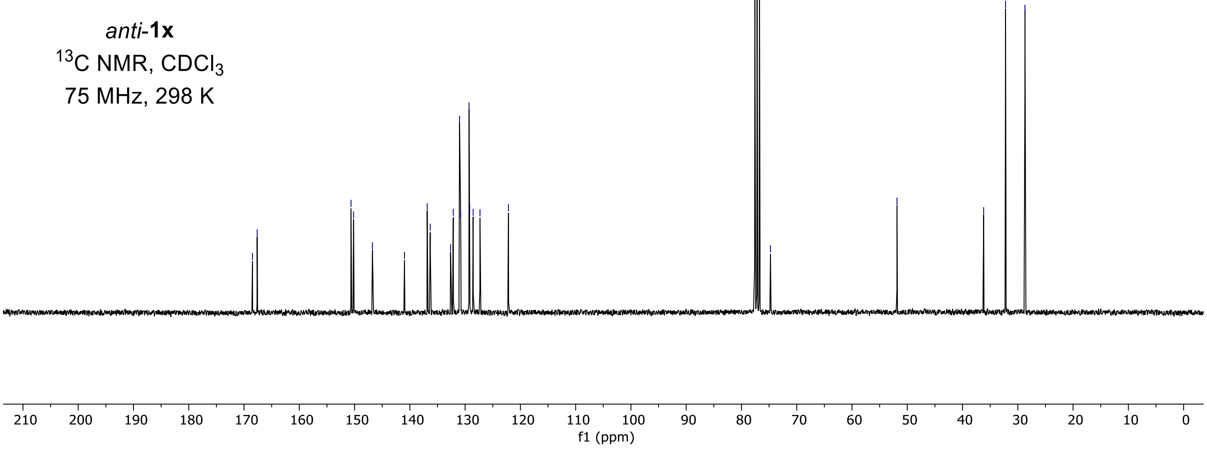

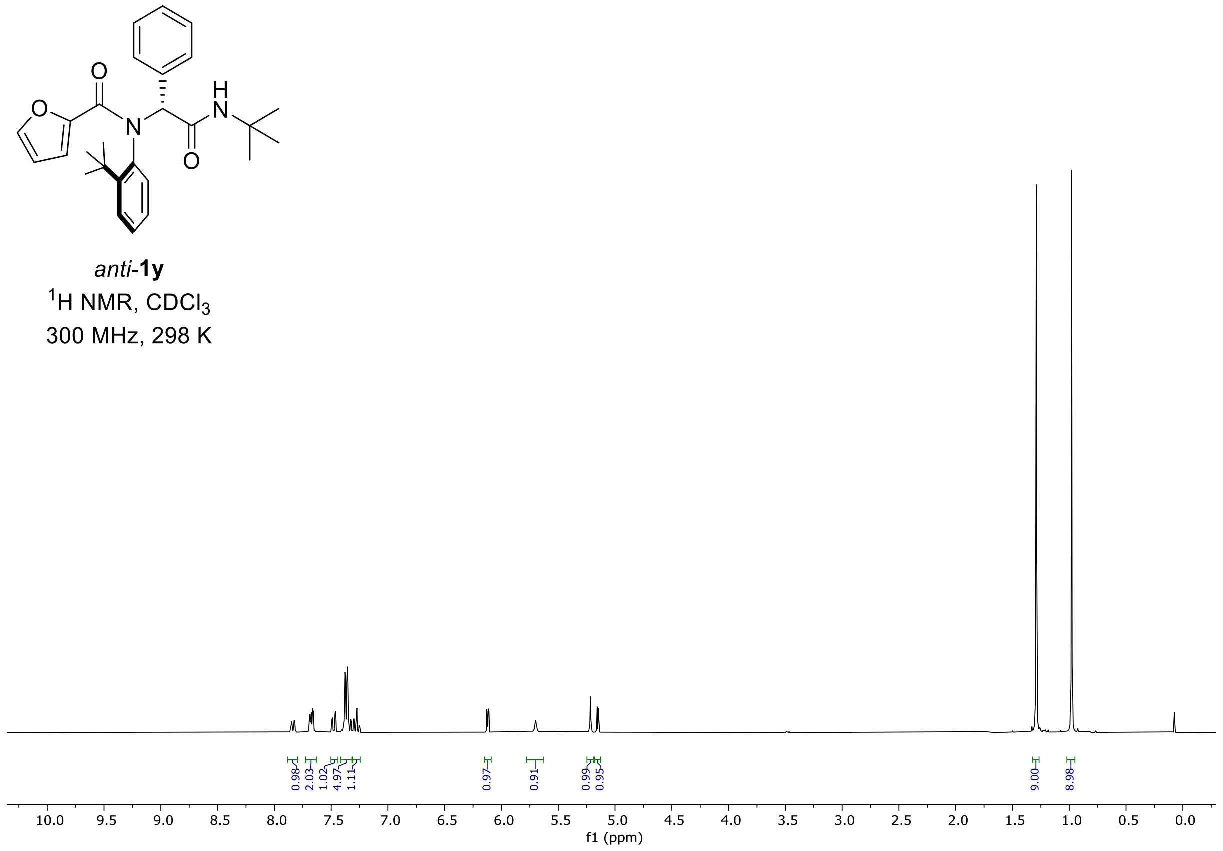
anti-1y
¹H NMR, CDCl₃
300 MHz, 298 K
0.98 2.03 1.02 4.97 1.11 0.97 0.91 0.99 0.95 9.00 8.98
10.0 9.5 9.0 8.5 8.0 7.5 7.0 6.5 6.0 5.5 5.0 4.5 4.0 3.5 3.0 2.5 2.0 1.5 1.0 0.5 0.0
f1 (ppm)

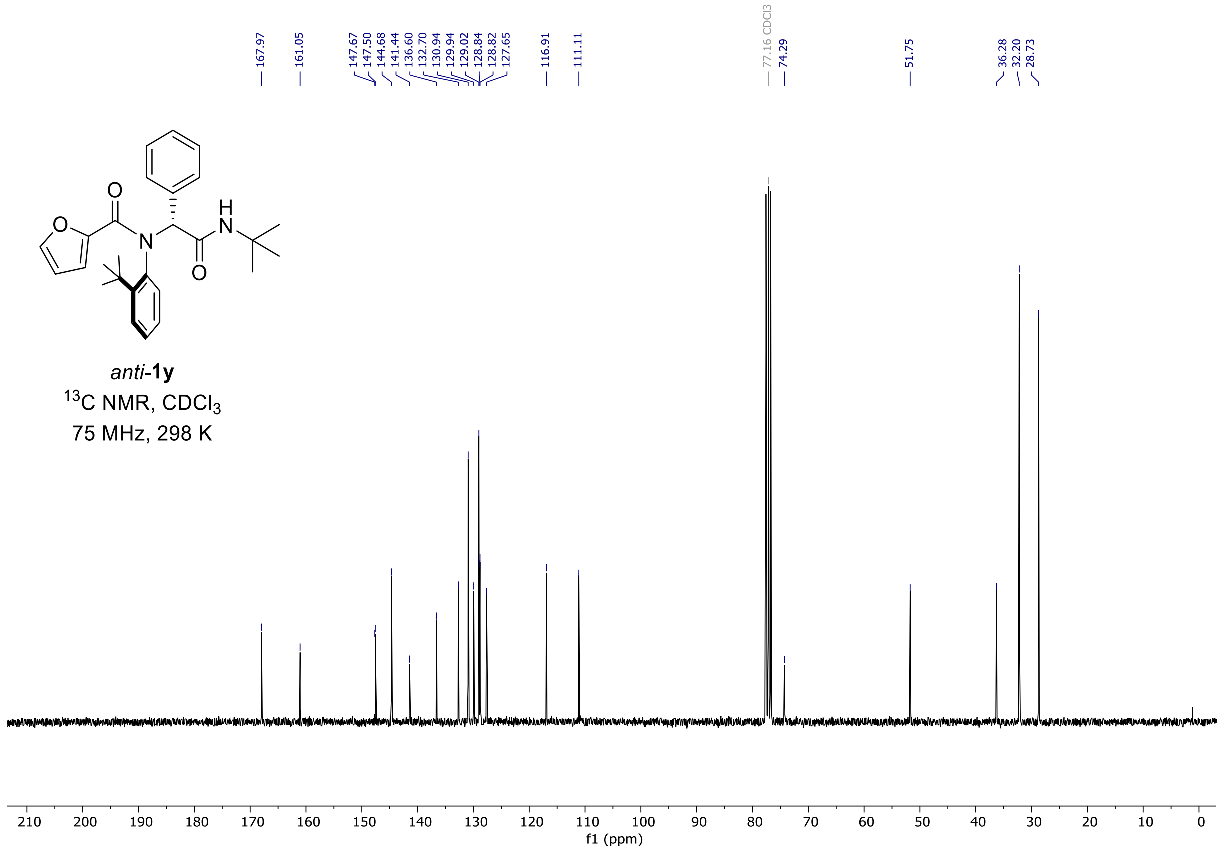
167.97
161.05
147.67 147.50 144.68 141.44 136.60 132.70 130.94 129.94 129.02 128.84 128.82 127.65
116.91
111.11
77.16 CDCl3
74.29
51.75
36.28 32.20 28.73
anti-1y
¹³C NMR, CDCl₃
75 MHz, 298 K
210 200 190 180 170 160 150 140 130 120 110 100 90 80 70 60 50 40 30 20 10 0
f1 (ppm)

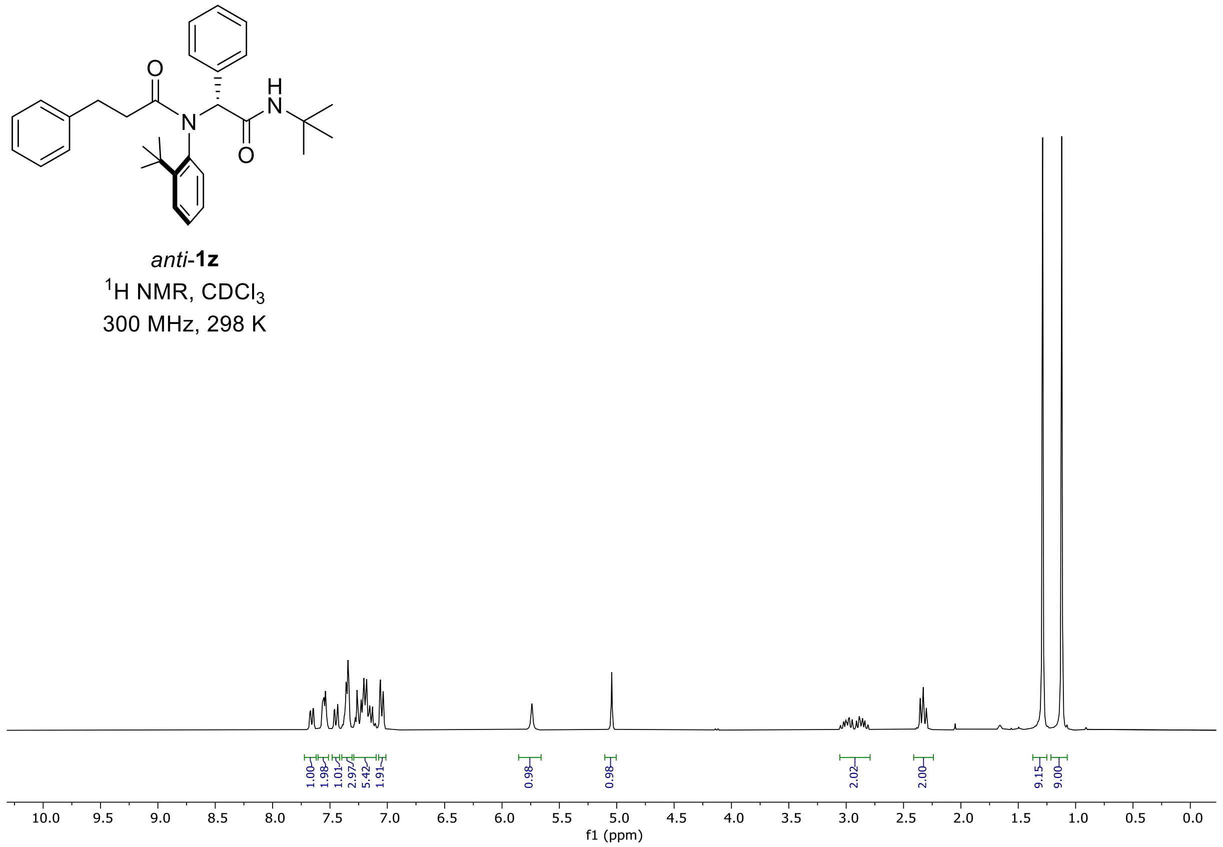

anti-1z
1H NMR, CDCl3
300 MHz, 298 K
1.00
1.98
1.01
2.97
5.42
1.91
0.98
0.98
2.02
2.00
9.15
9.00
10.0 9.5 9.0 8.5 8.0 7.5 7.0 6.5 6.0 5.5 5.0 4.5 4.0 3.5 3.0 2.5 2.0 1.5 1.0 0.5 0.0
f1 (ppm)

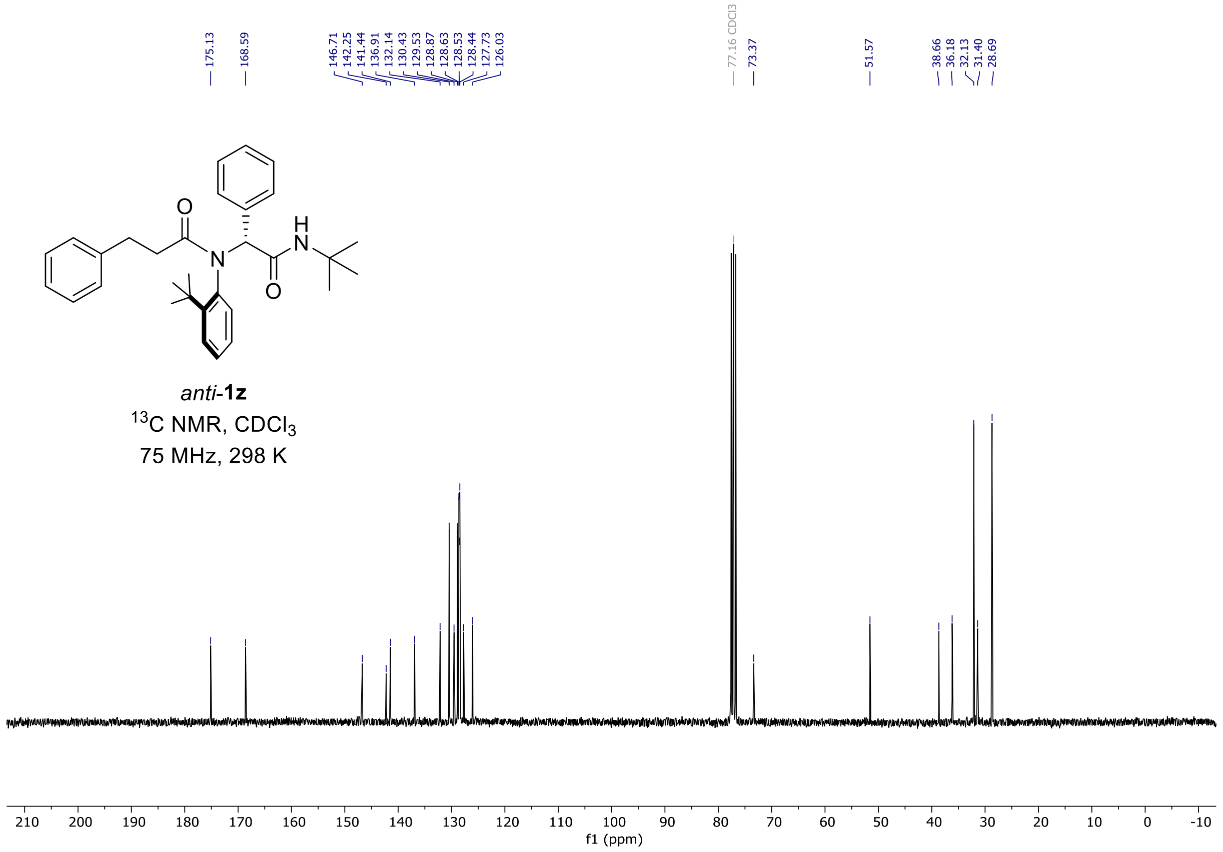

175.13
168.59
146.71
142.25
141.44
136.91
132.14
130.43
129.53
128.87
128.63
128.53
128.44
127.73
126.03
77.16 CDCl3
73.37
51.57
38.66
36.18
32.13
31.40
28.69
anti-1z
13C NMR, CDCl3
75 MHz, 298 K
210 200 190 180 170 160 150 140 130 120 110 100 90 80 70 60 50 40 30 20 10 0 -10
f1 (ppm)

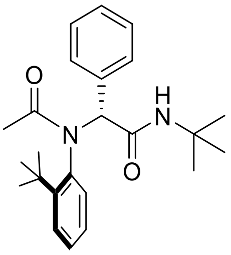

anti-**1aa**
^{1}H NMR, $CDCl_3$
300 MHz, 298 K

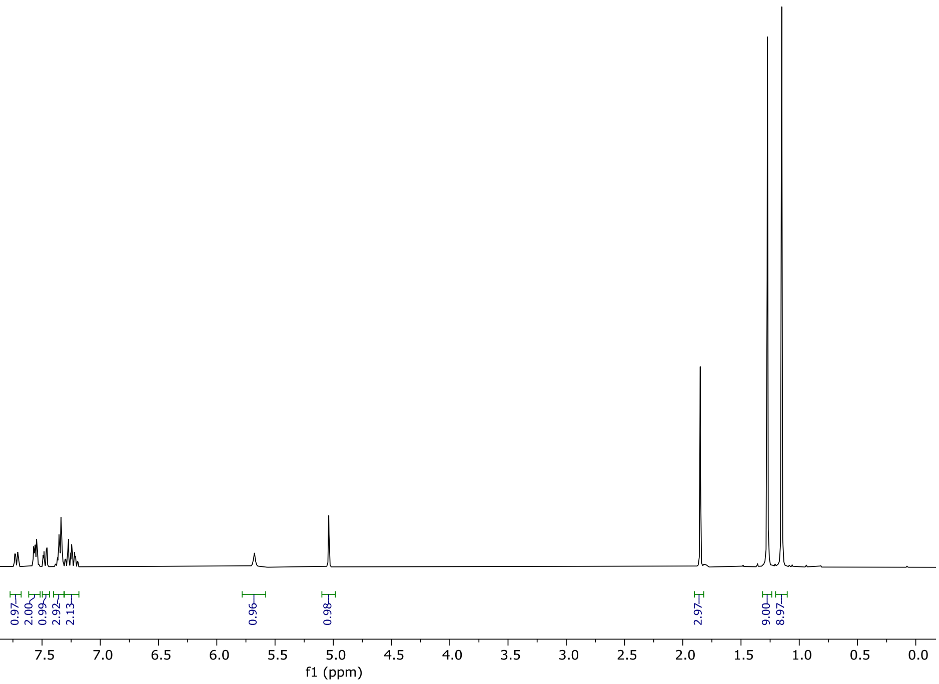

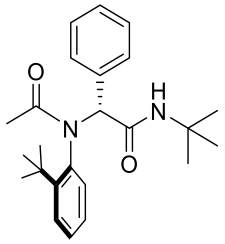

anti-**1aa**
^{13}C NMR, $CDCl_3$
75 MHz, 298 K

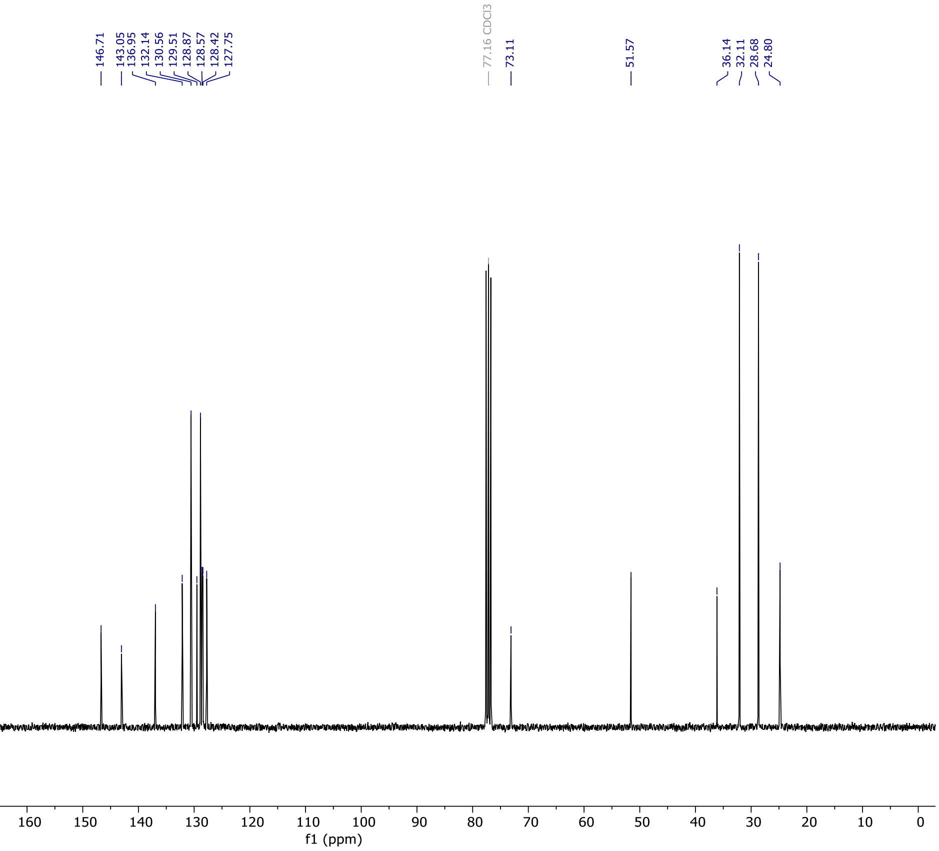

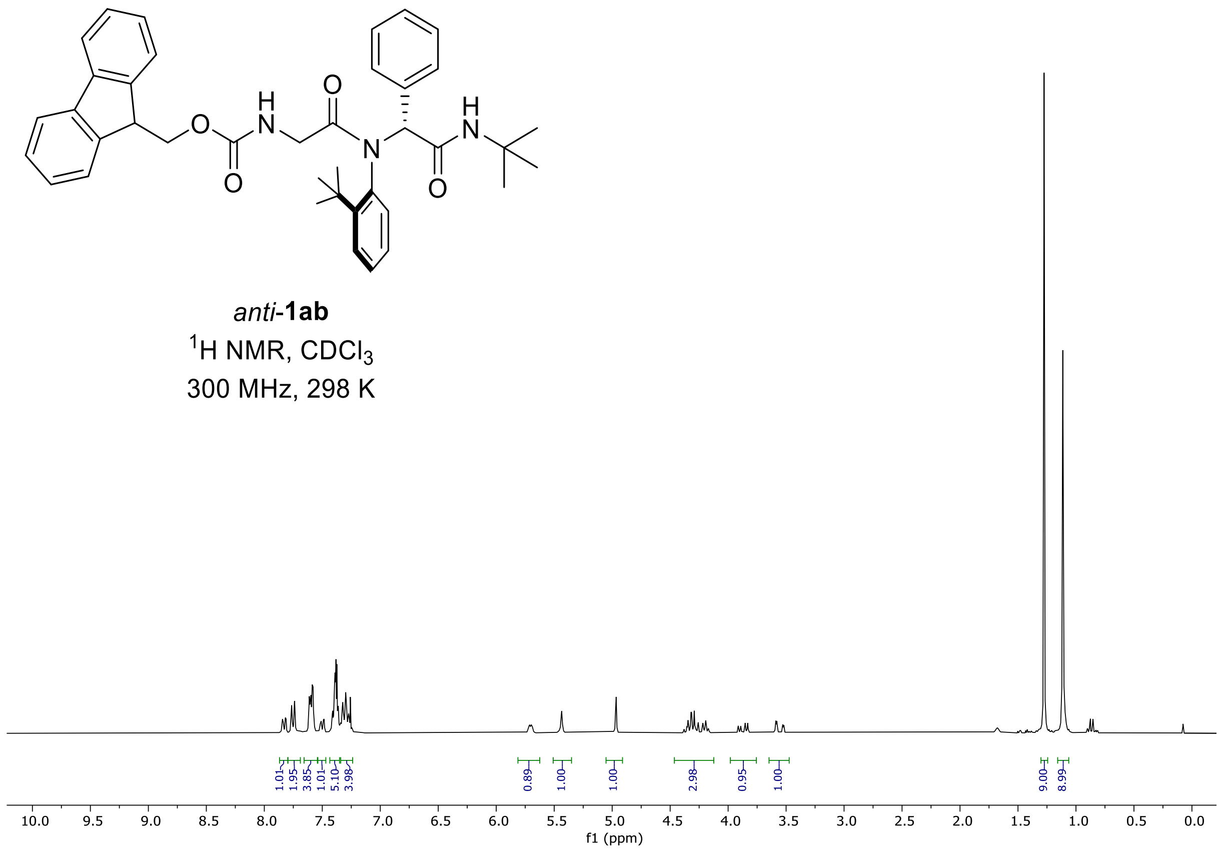

anti-1ab
1H NMR, CDCl3
300 MHz, 298 K
1.01 1.95 3.85 1.01 5.10 3.98 0.89 1.00 1.00 2.98 0.95 1.00 9.00 8.99
10.0 9.5 9.0 8.5 8.0 7.5 7.0 6.5 6.0 5.5 5.0 4.5 4.0 3.5 3.0 2.5 2.0 1.5 1.0 0.5 0.0
f1 (ppm)

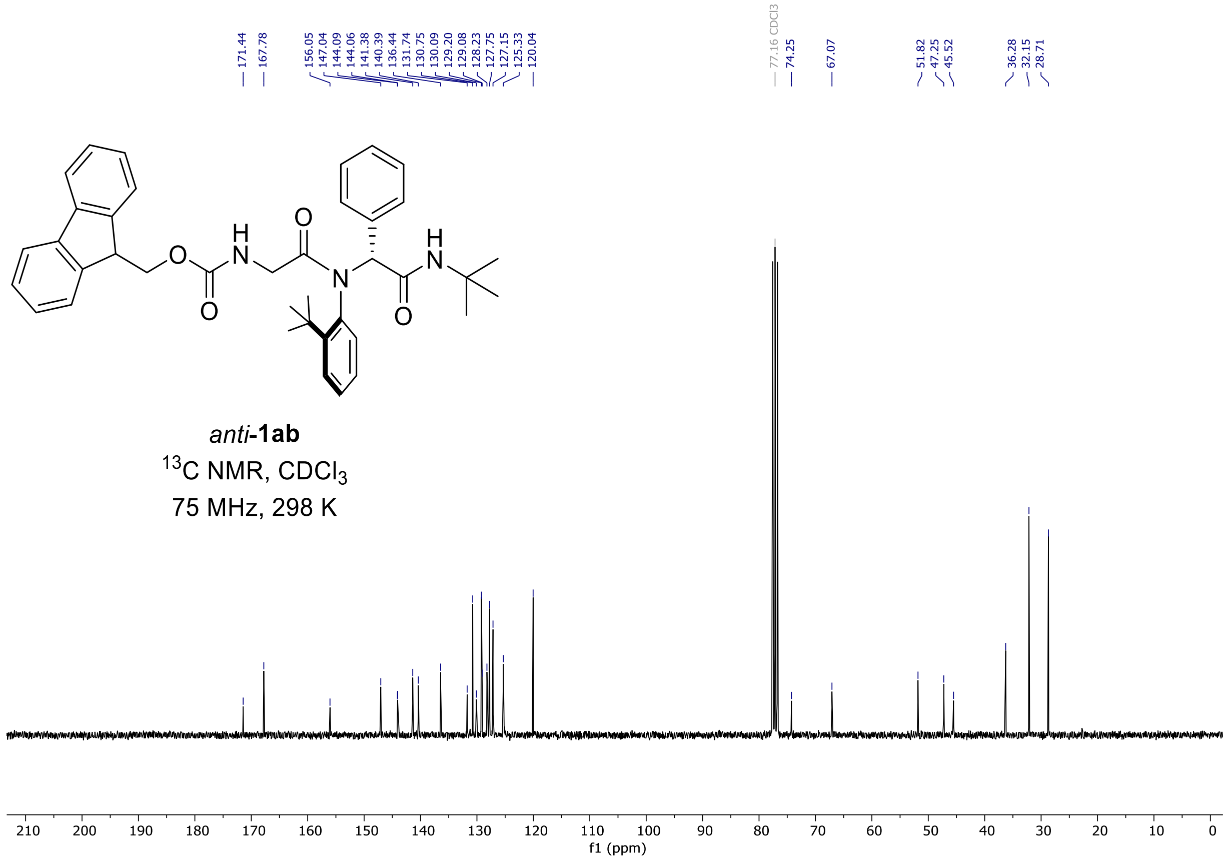

171.44 167.78 156.05 147.04 144.09 144.06 141.38 140.39 136.44 131.74 130.75 130.09 129.20 129.08 128.23 127.75 127.15 125.33 120.04
77.16 CDCl3 74.25 67.07 51.82 47.25 45.52 36.28 32.15 28.71
anti-1ab
13C NMR, CDCl3
75 MHz, 298 K
210 200 190 180 170 160 150 140 130 120 110 100 90 80 70 60 50 40 30 20 10 0
f1 (ppm)

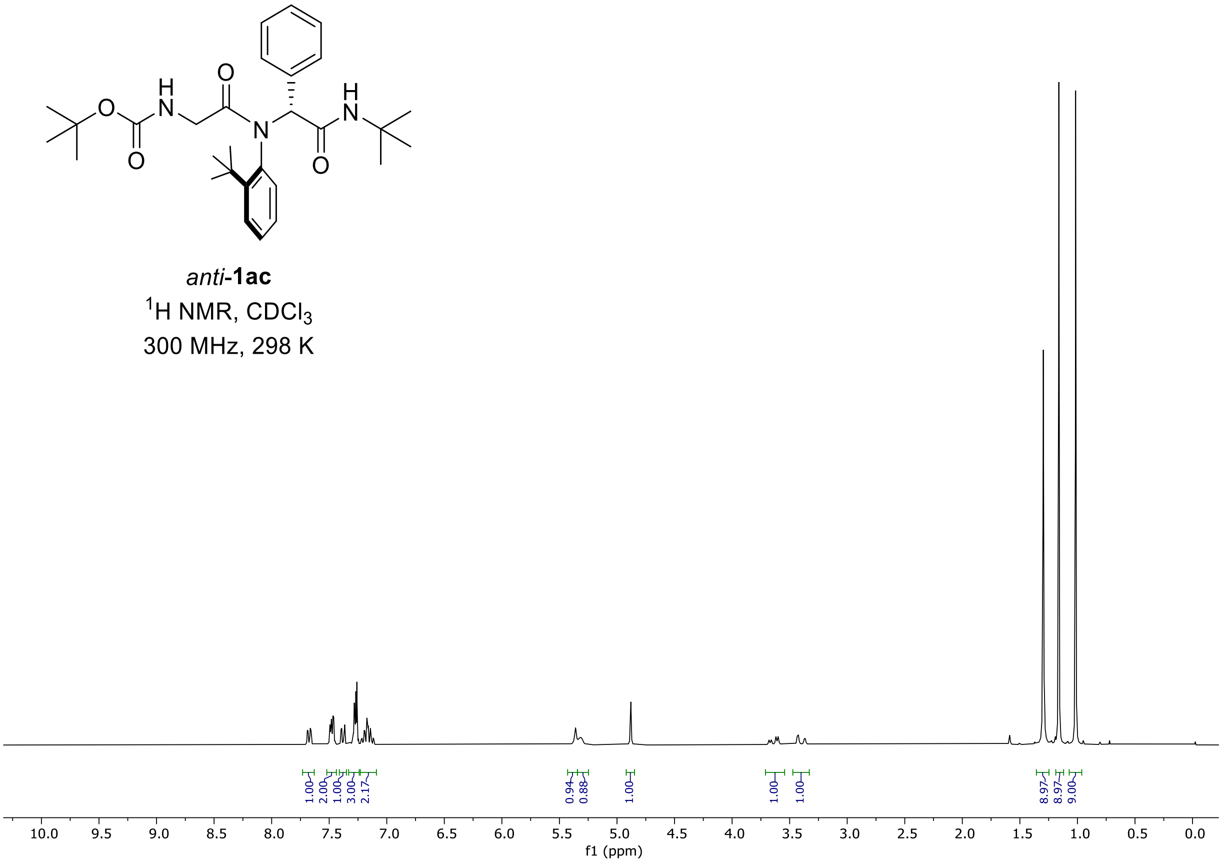

anti-**1ac**
^{1}H NMR, $CDCl_3$
300 MHz, 298 K

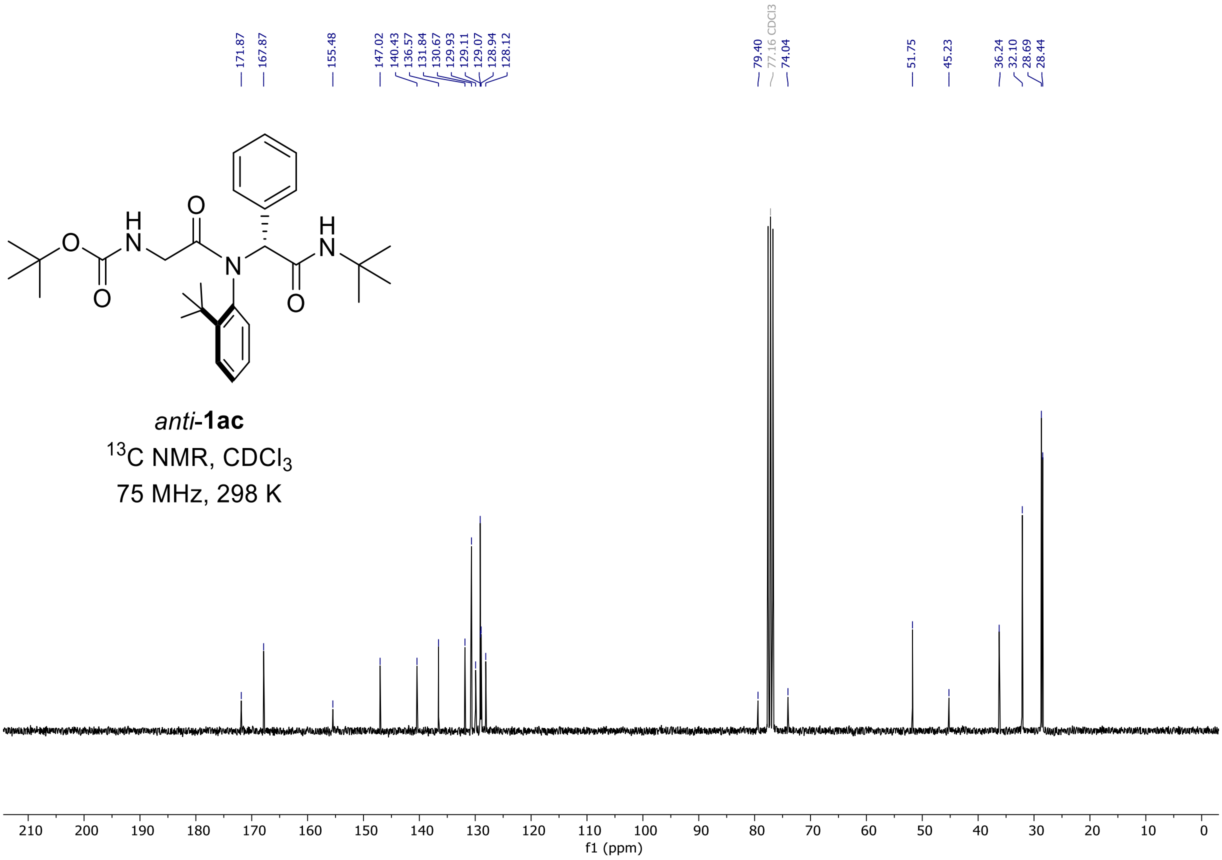

anti-**1ac**
^{13}C NMR, $CDCl_3$
75 MHz, 298 K

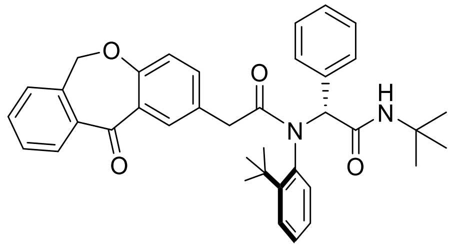

anti-**1ad**
^{1}H NMR, $CDCl_3$
400 MHz, 298 K

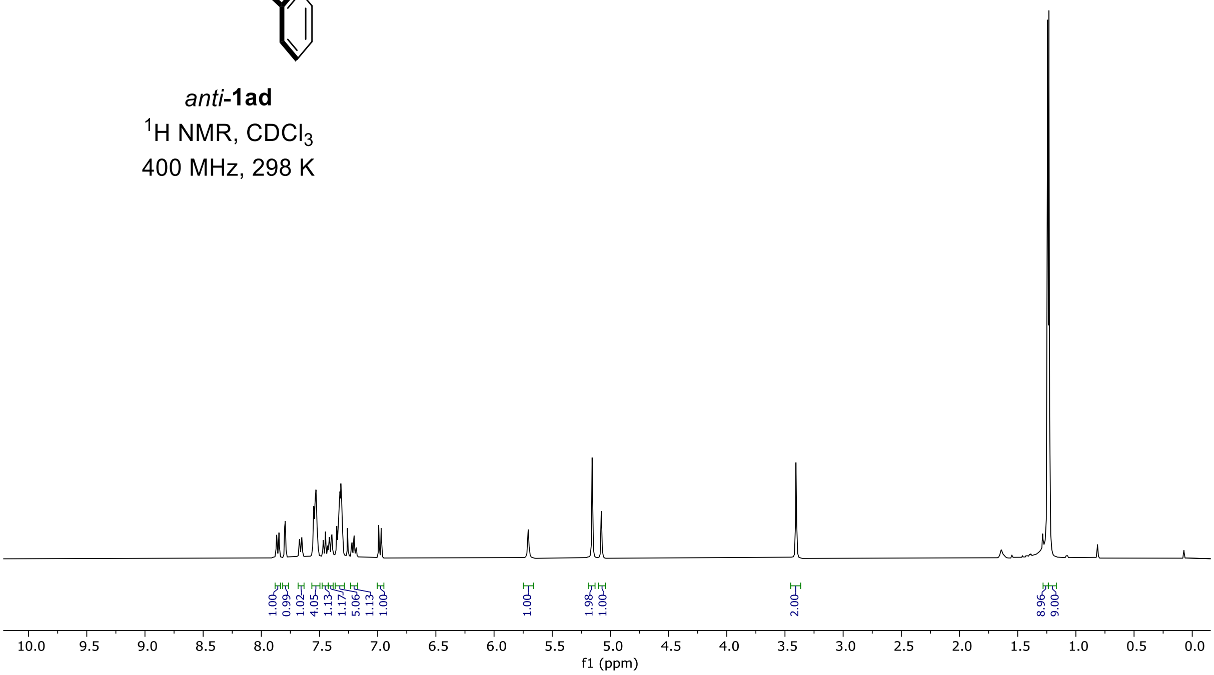

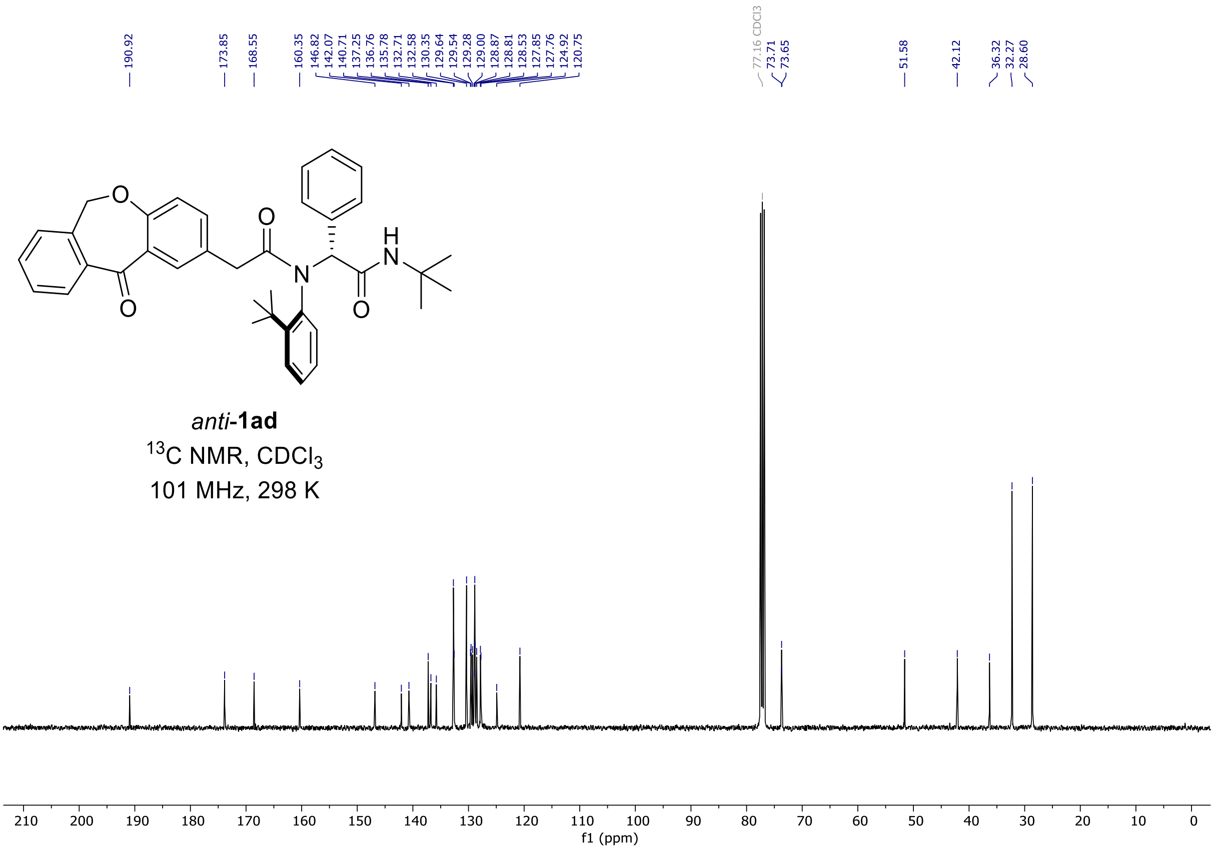

anti-**1ad**
^{13}C NMR, $CDCl_3$
101 MHz, 298 K

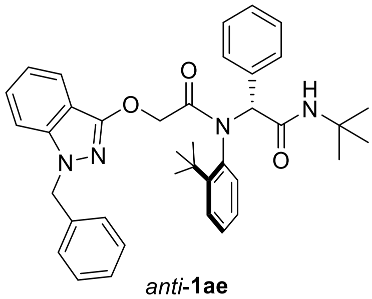

anti-**1ae**
^{1}H NMR, $CDCl_3$
400 MHz, 298 K

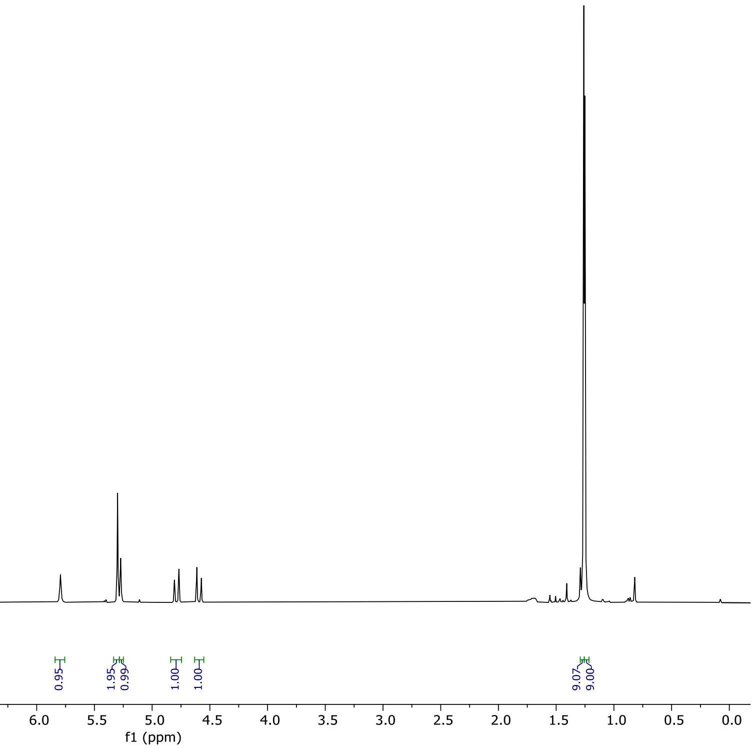

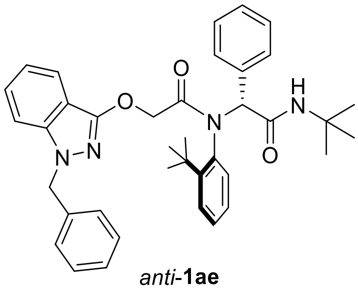

anti-**1ae**
^{13}C NMR, $CDCl_3$
101 MHz, 298 K

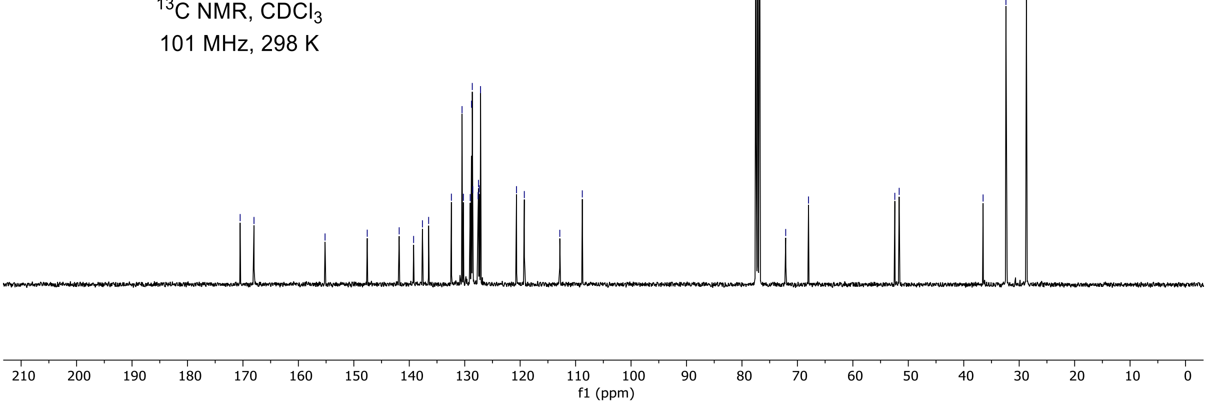

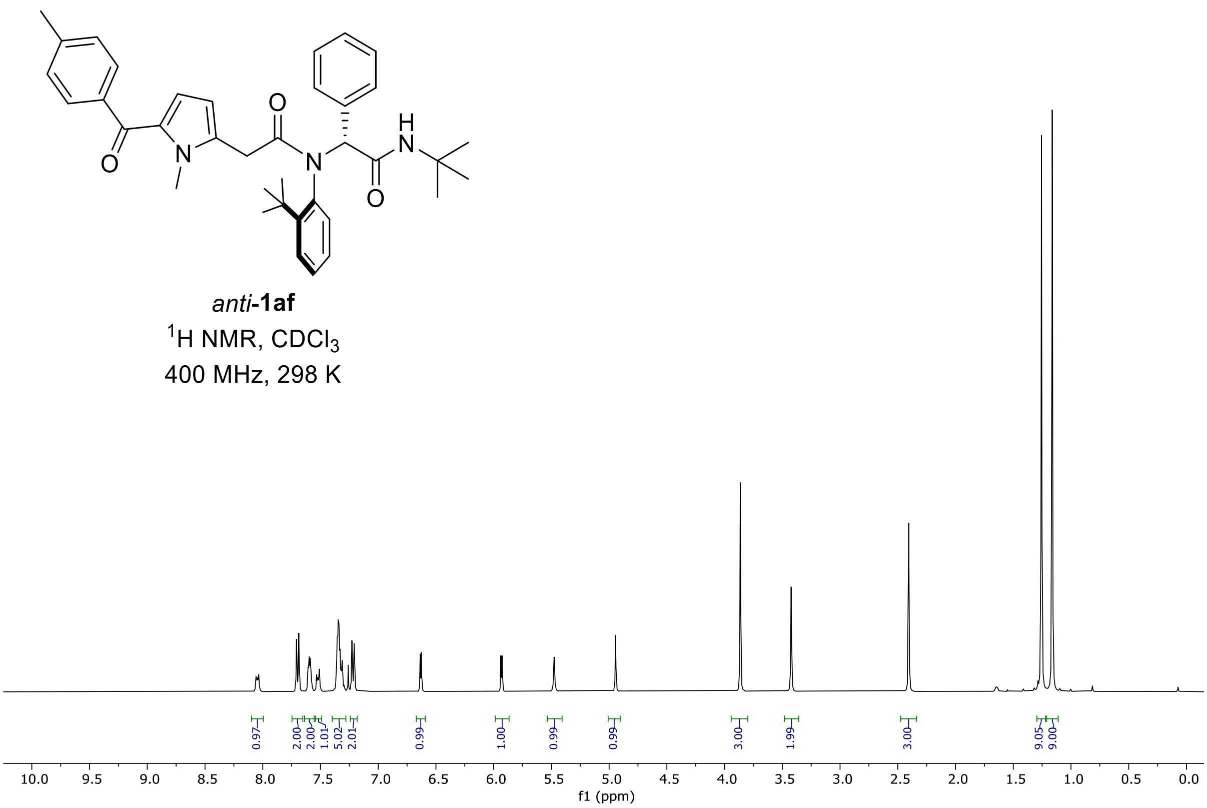

anti-1af
1H NMR, CDCl3
400 MHz, 298 K
0.97
2.00
2.00
1.01
5.02
2.01
0.99
1.00
0.99
0.99
3.00
1.99
3.00
9.05
9.00
10.0 9.5 9.0 8.5 8.0 7.5 7.0 6.5 6.0 5.5 5.0 4.5 4.0 3.5 3.0 2.5 2.0 1.5 1.0 0.5 0.0
f1 (ppm)

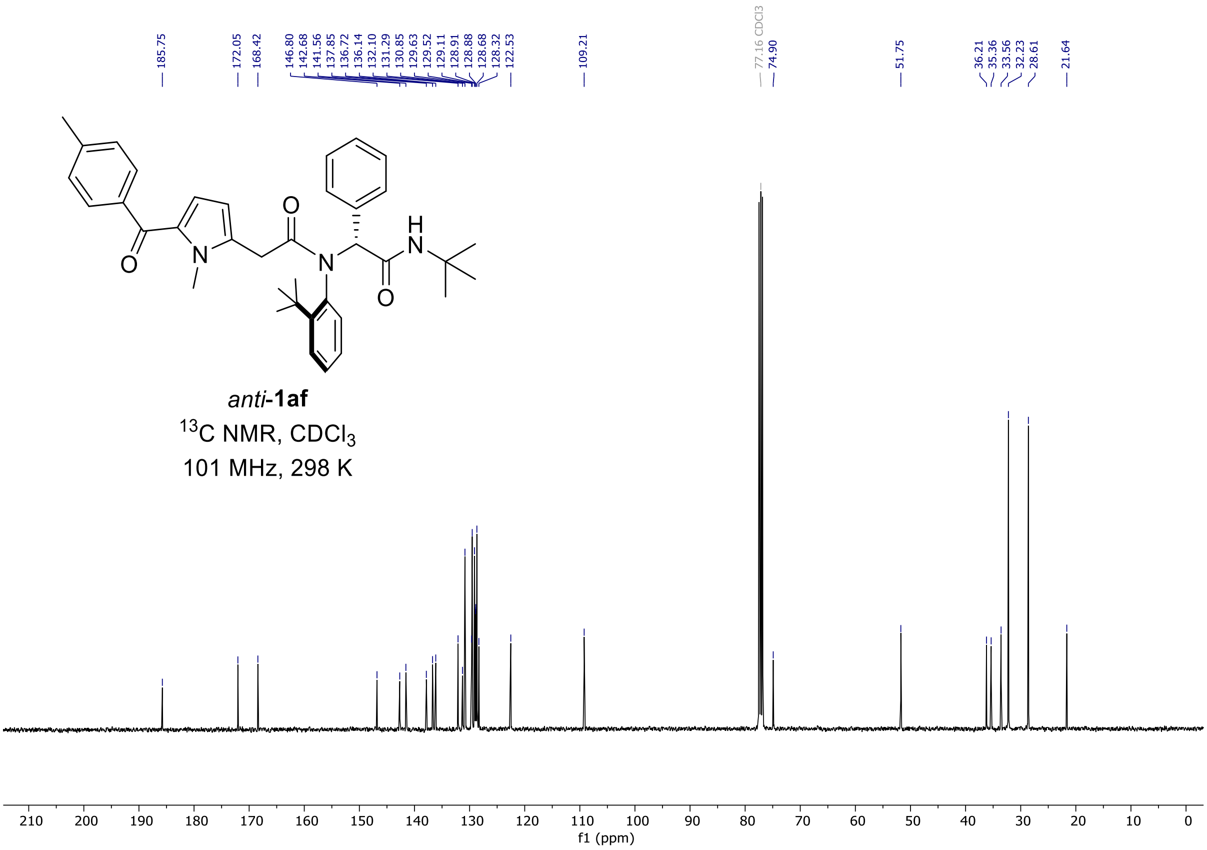

185.75
172.05
168.42
146.80
142.68
141.56
137.85
136.72
136.14
132.10
131.29
130.85
129.63
129.52
129.11
128.91
128.88
128.68
128.32
122.53
109.21
77.16 CDCl3
74.90
51.75
36.21
35.36
33.56
32.23
28.61
21.64
anti-1af
13C NMR, CDCl3
101 MHz, 298 K
210 200 190 180 170 160 150 140 130 120 110 100 90 80 70 60 50 40 30 20 10 0
f1 (ppm)

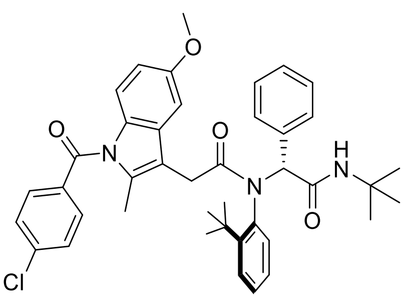

anti-**1ag**
^{1}H NMR, $CDCl_3$
400 MHz, 298 K

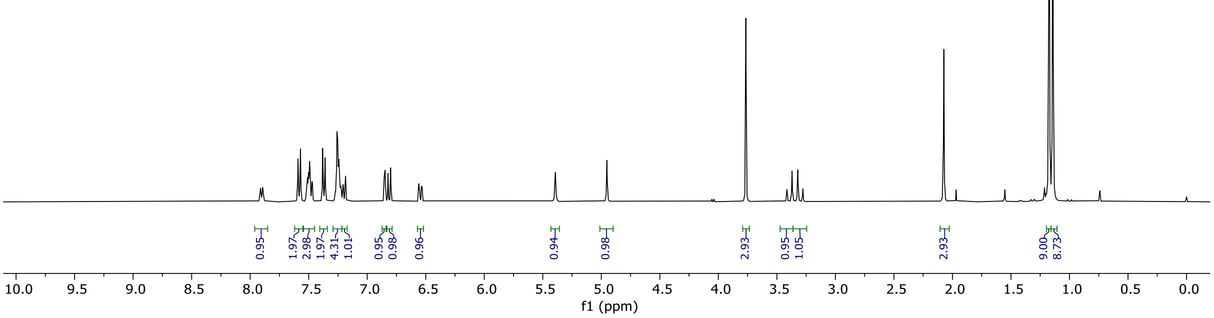

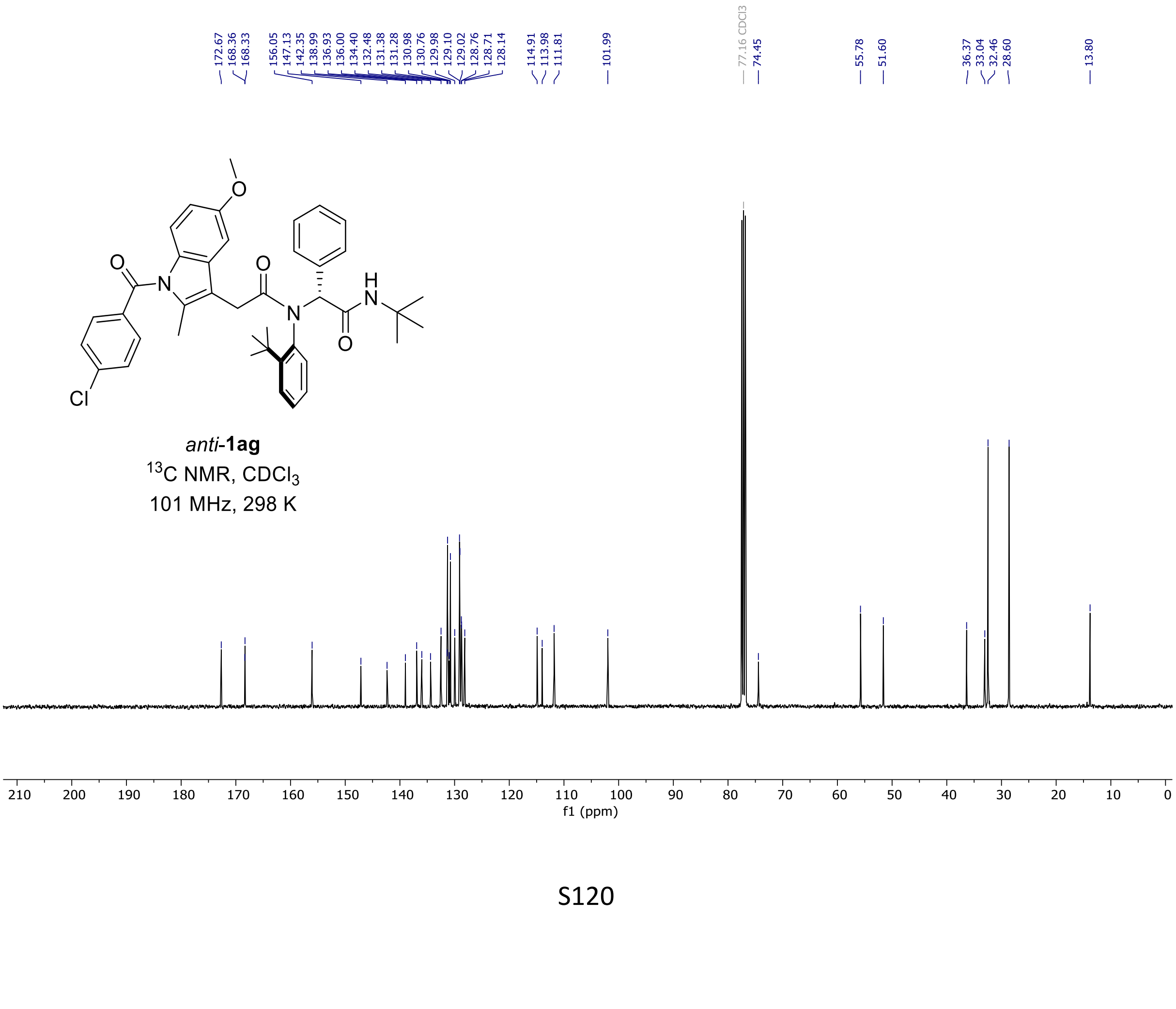

anti-**1ag**
^{13}C NMR, $CDCl_3$
101 MHz, 298 K

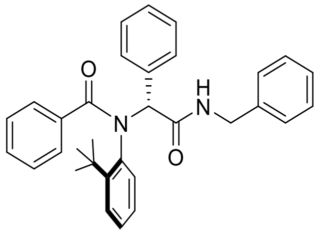

anti-**1ah**

^{1}H NMR, $CDCl_3$

300 MHz, 298 K

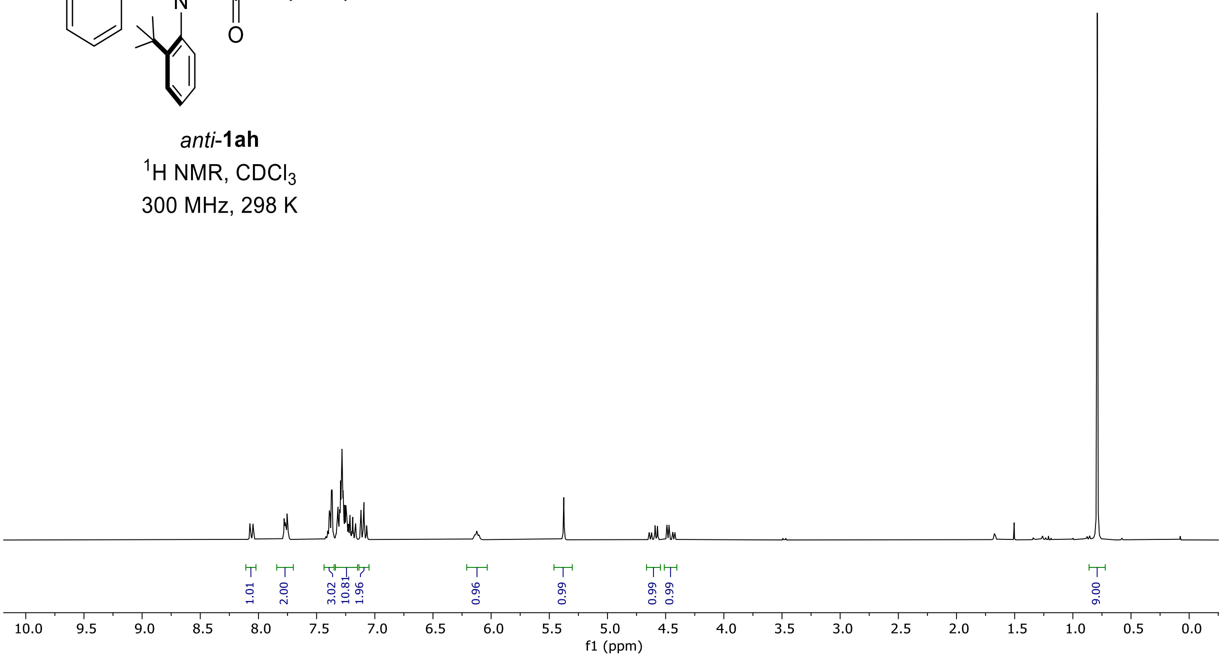

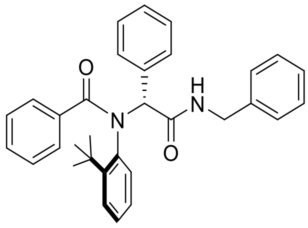

anti-**1ah**

^{13}C NMR, $CDCl_3$

75 MHz, 298 K

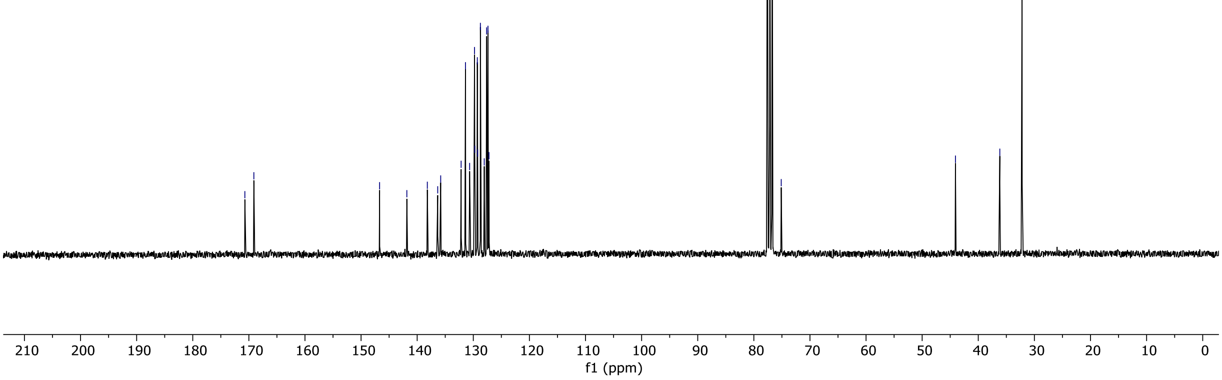

anti-**1ai**
^{1}H NMR, $CDCl_3$
300 MHz, 298 K

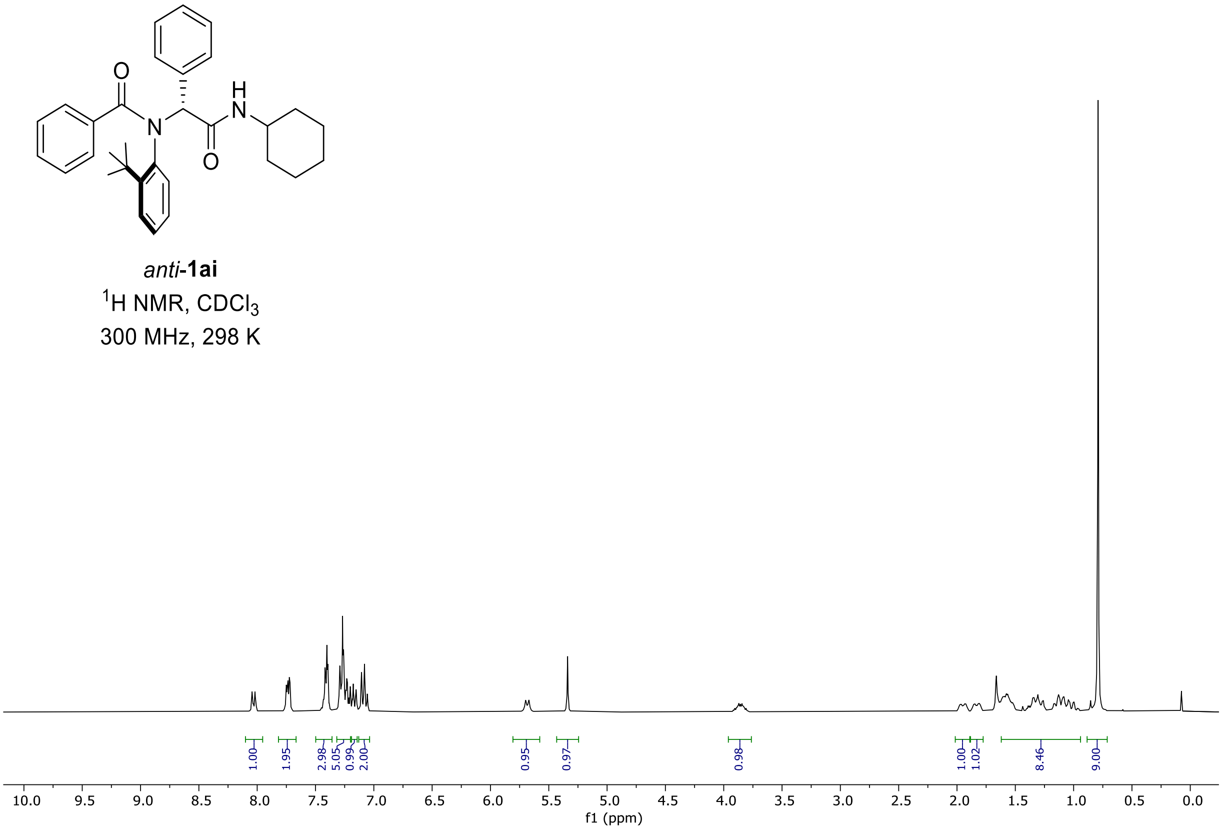

anti-**1ai**
^{13}C NMR, $CDCl_3$
75 MHz, 298 K

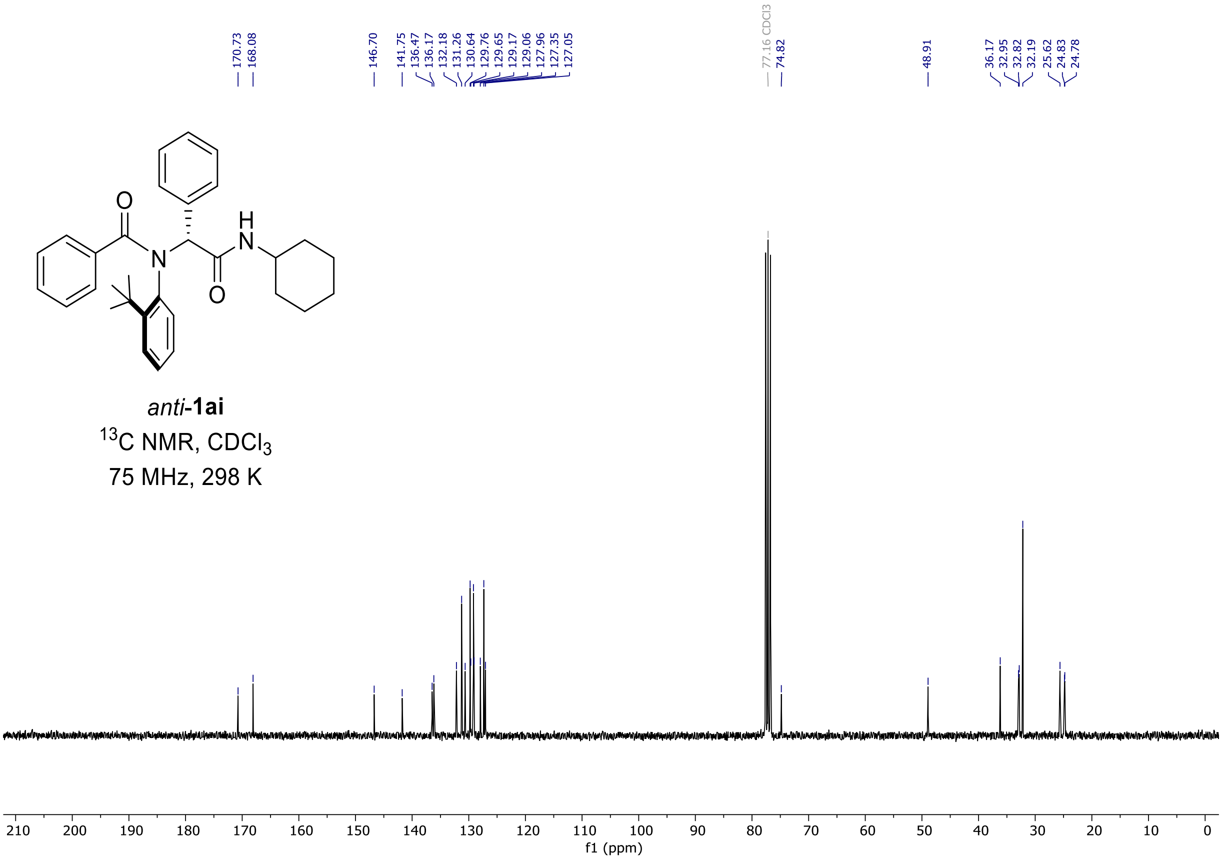

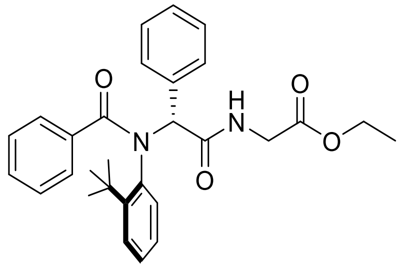

anti-**1aj**
^{1}H NMR, $CDCl_3$
300 MHz, 298 K

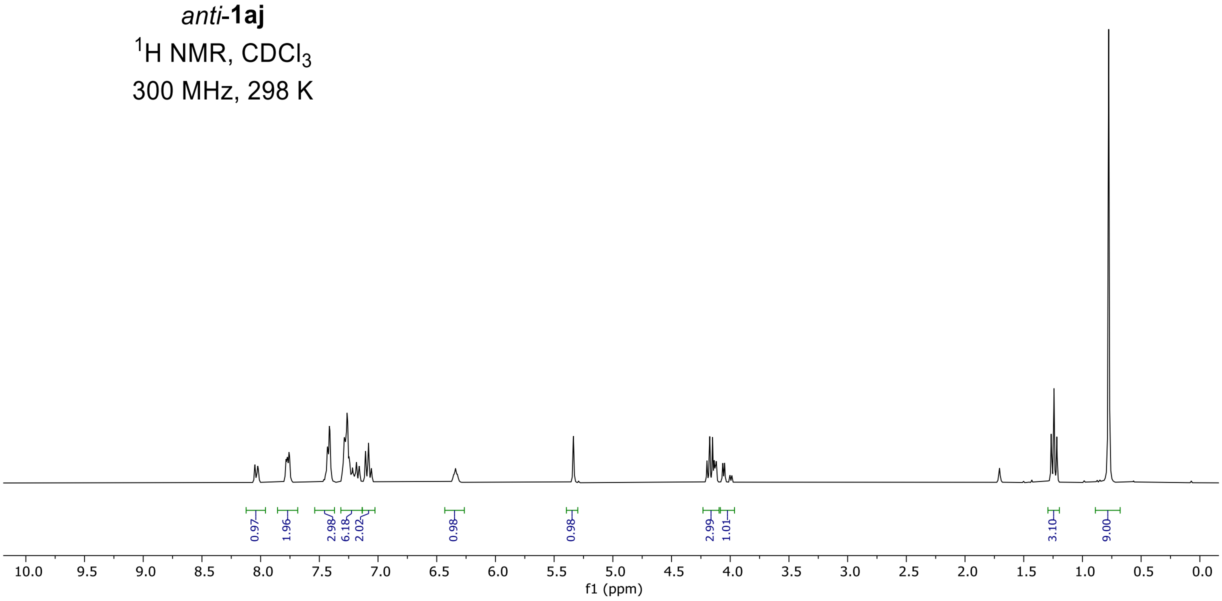

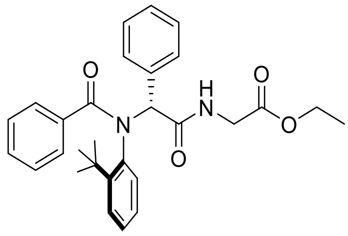

anti-**1aj**
^{13}C NMR, $CDCl_3$
75 MHz, 298 K

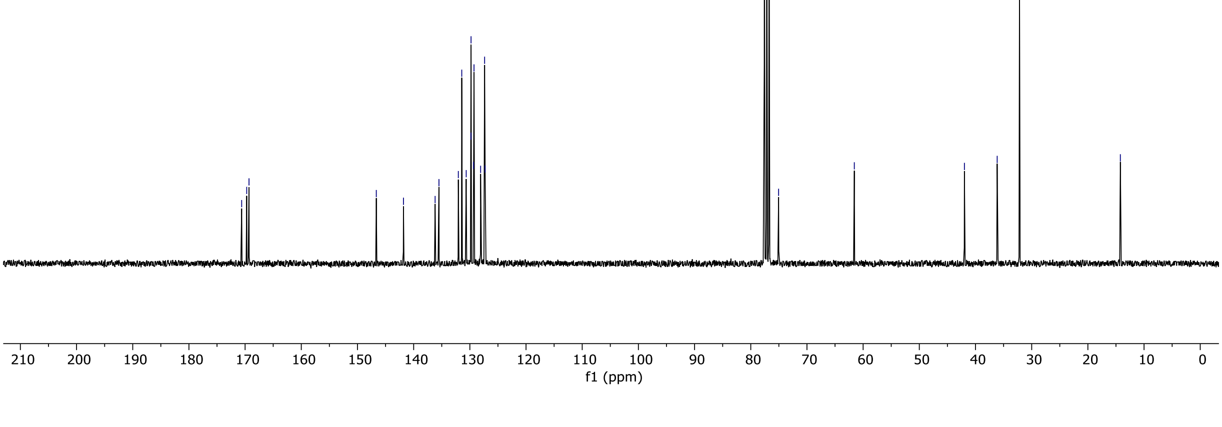

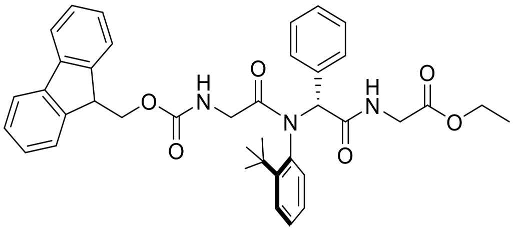

anti-**1ak**
^{1}H NMR, $CDCl_3$
300 MHz, 298 K

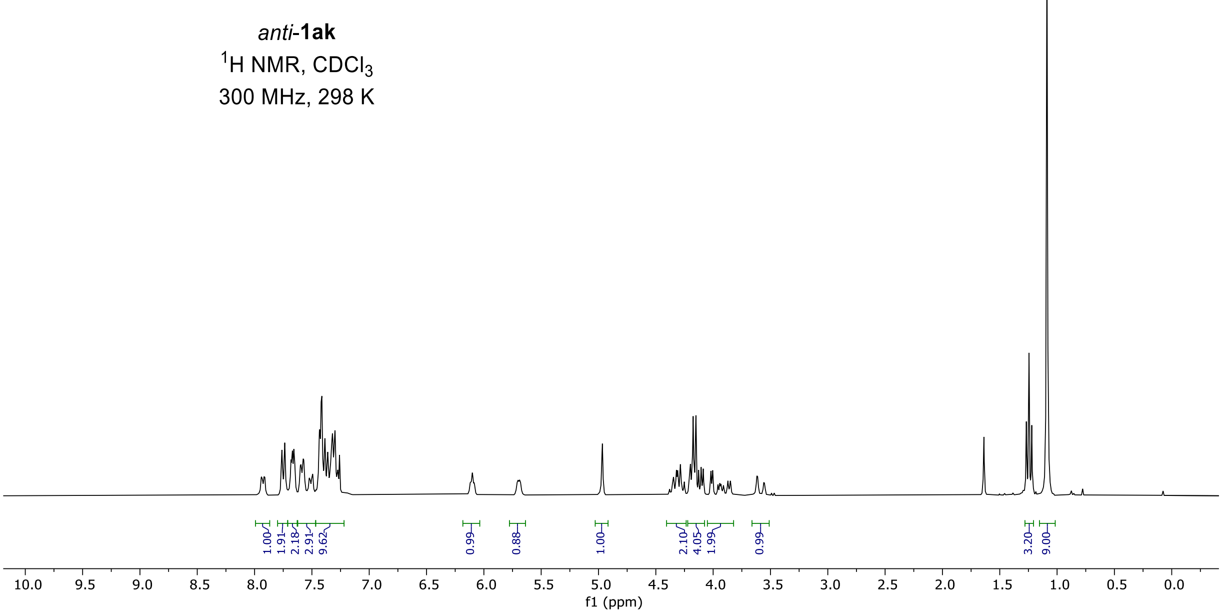

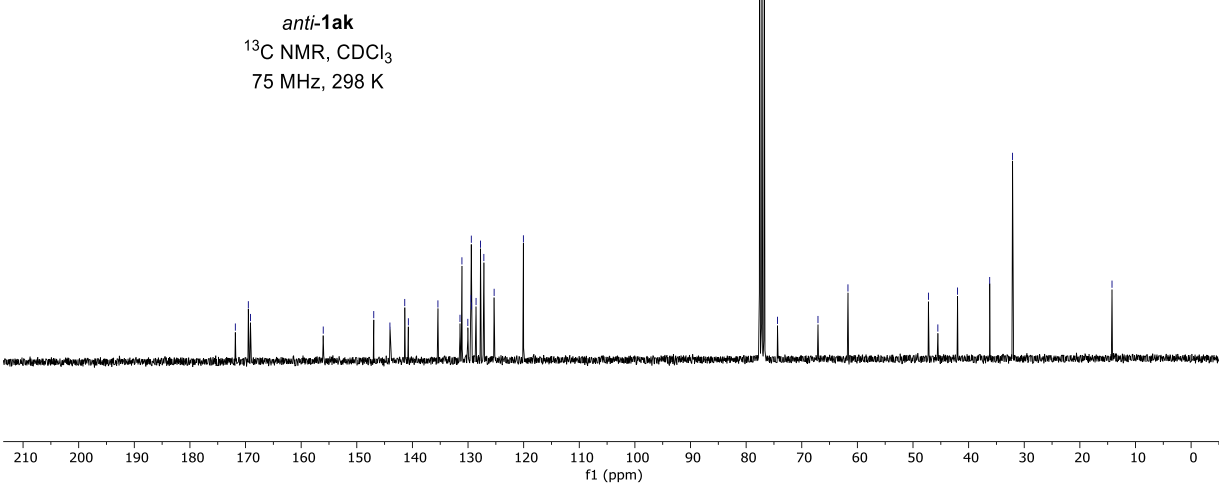

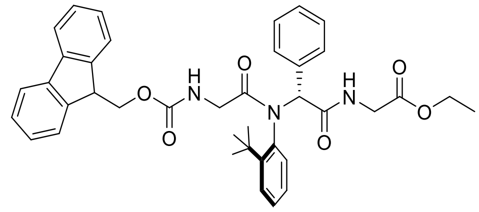

anti-**1ak**
^{13}C NMR, $CDCl_3$
75 MHz, 298 K

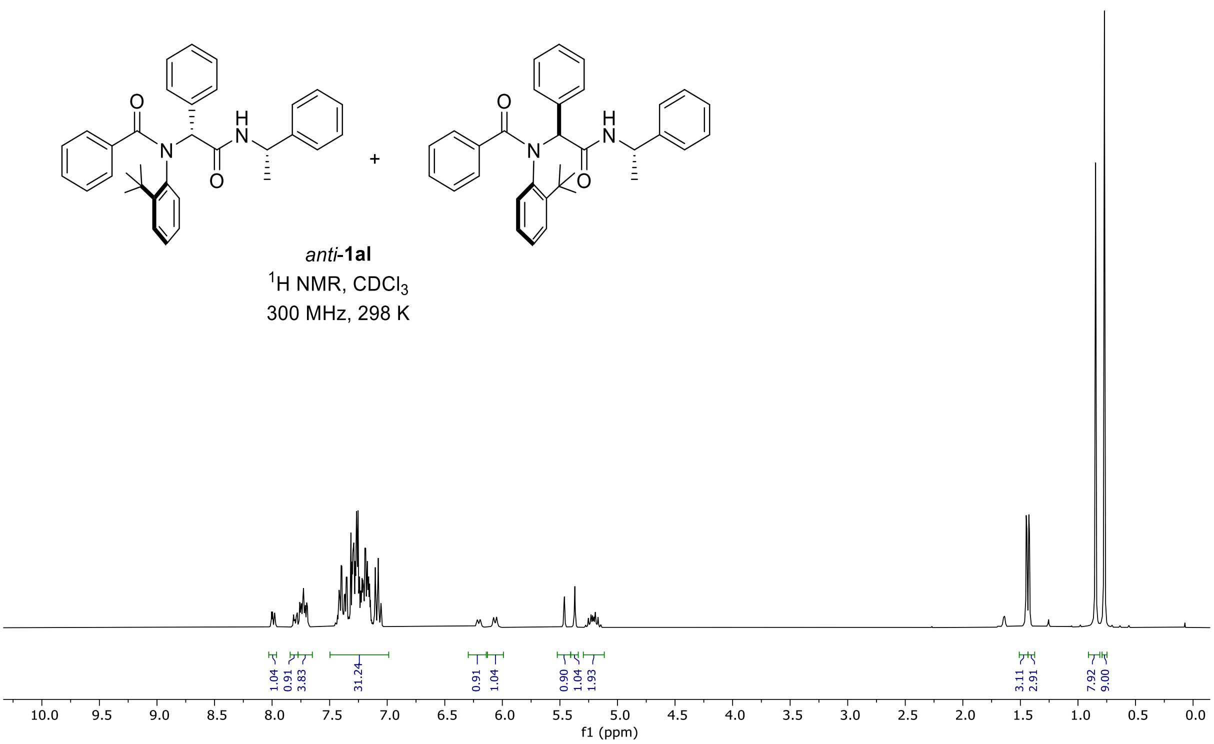
anti-1al
1H NMR, CDCl3
300 MHz, 298 K
f1 (ppm)

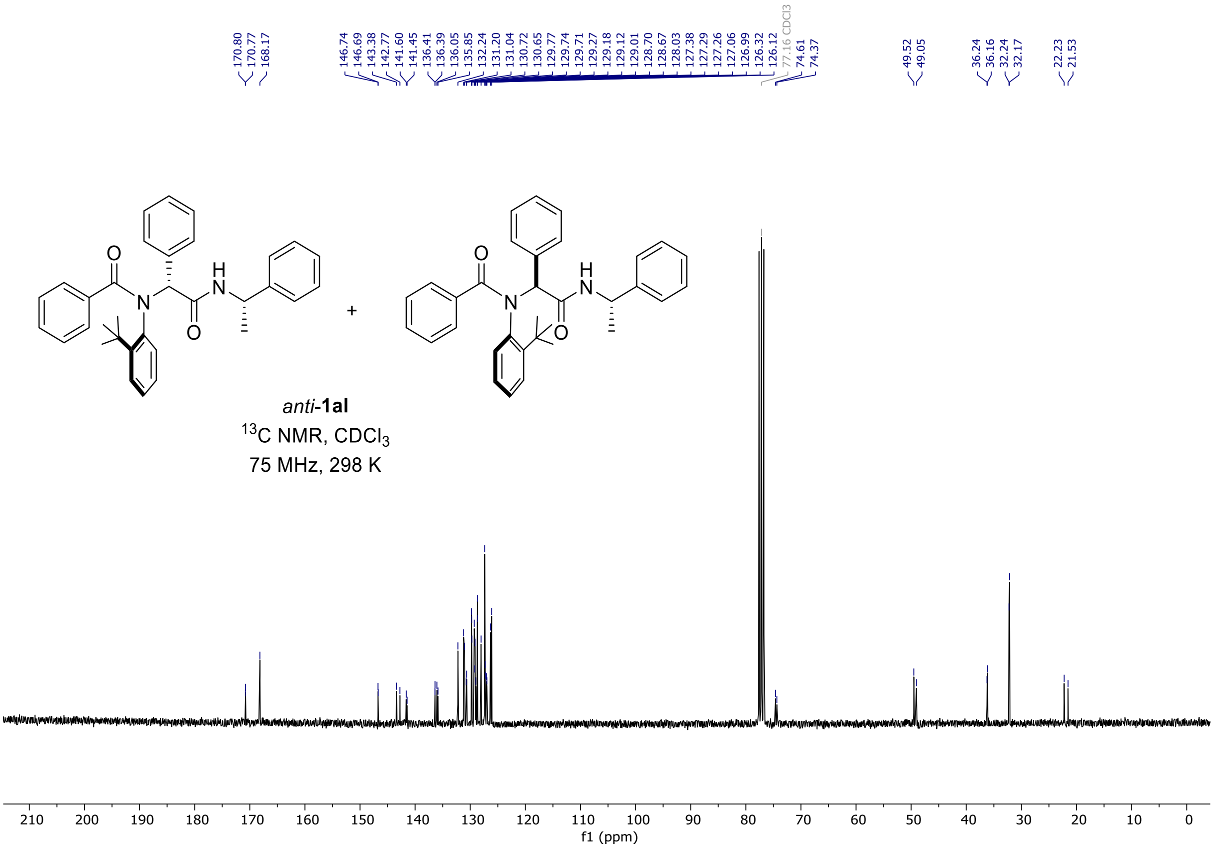
170.80
170.77
168.17
146.74
146.69
143.38
142.77
141.60
141.45
136.41
136.39
136.05
135.85
132.24
131.20
131.04
130.72
130.65
129.77
129.74
129.71
129.27
129.18
129.12
129.01
128.70
128.67
128.03
127.38
127.29
127.26
127.06
126.99
126.32
126.12
77.16 CDCl3
74.61
74.37
49.52
49.05
36.24
36.16
32.24
32.17
22.23
21.53
anti-1al
13C NMR, CDCl3
75 MHz, 298 K
f1 (ppm)

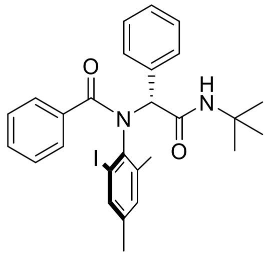

anti-**1am**
^{1}H NMR, $CDCl_3$
300 MHz, 298 K

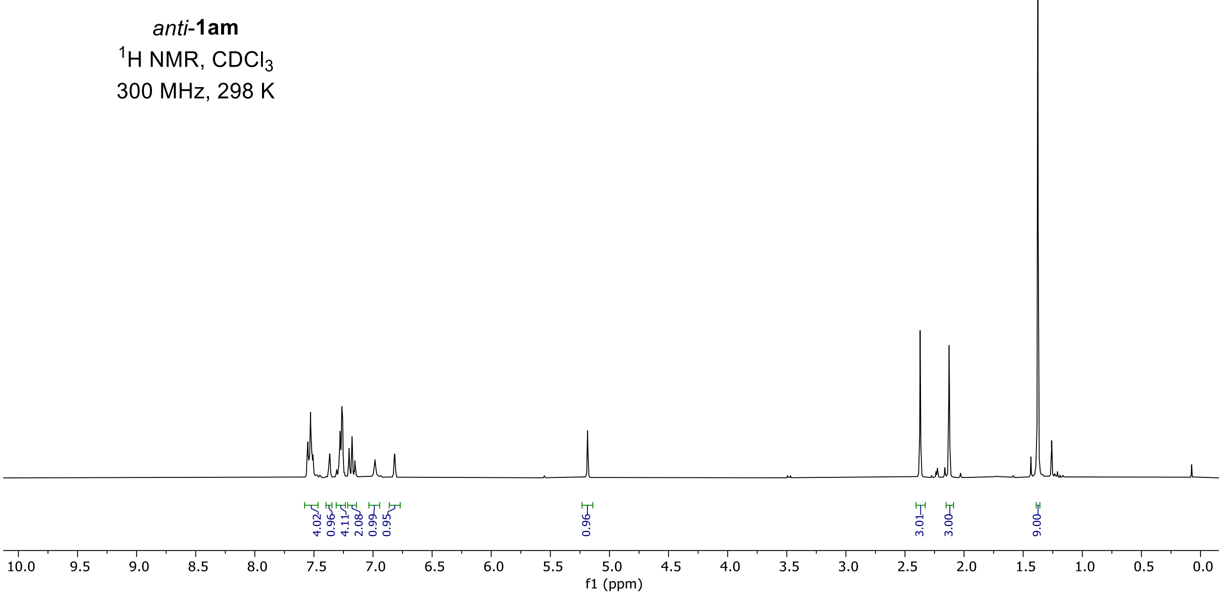

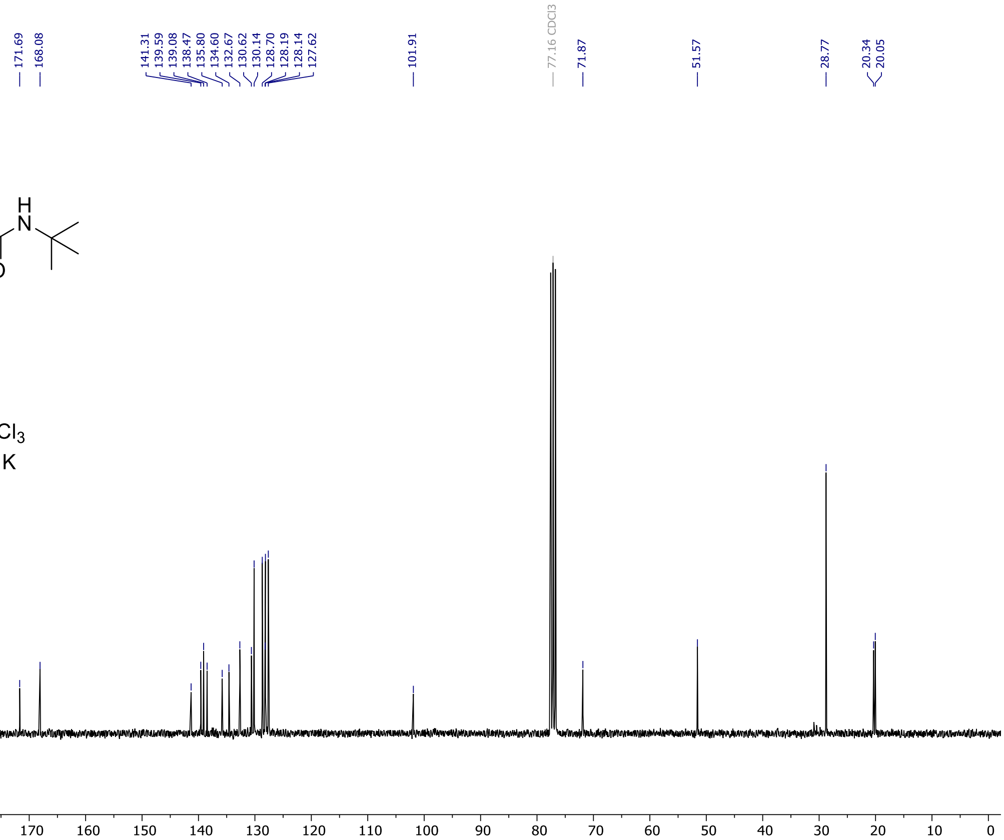

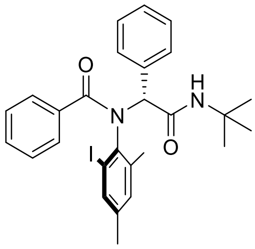

anti-**1am**
^{13}C NMR, $CDCl_3$
75 MHz, 298 K

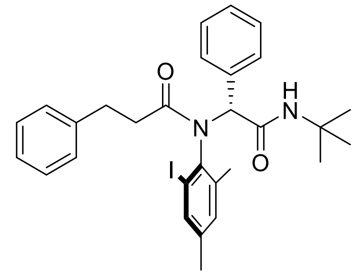

anti-**1an**
^{1}H NMR, $CDCl_3$
400 MHz, 298 K

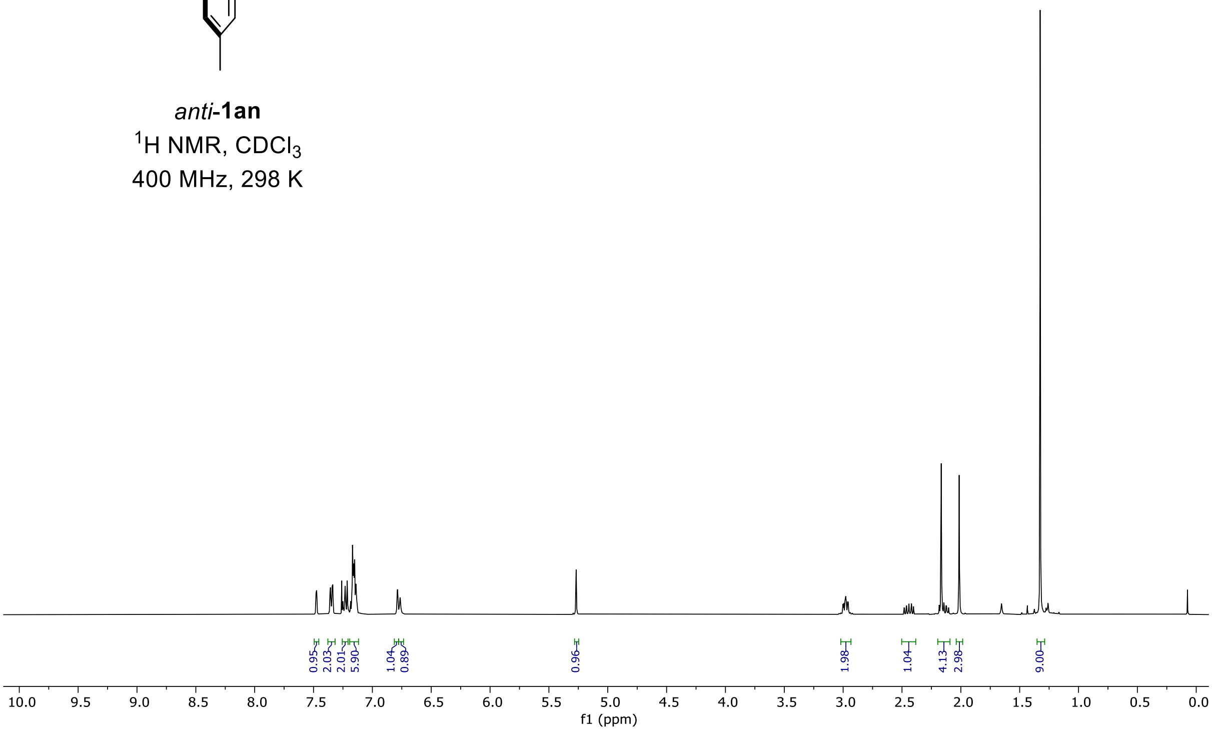

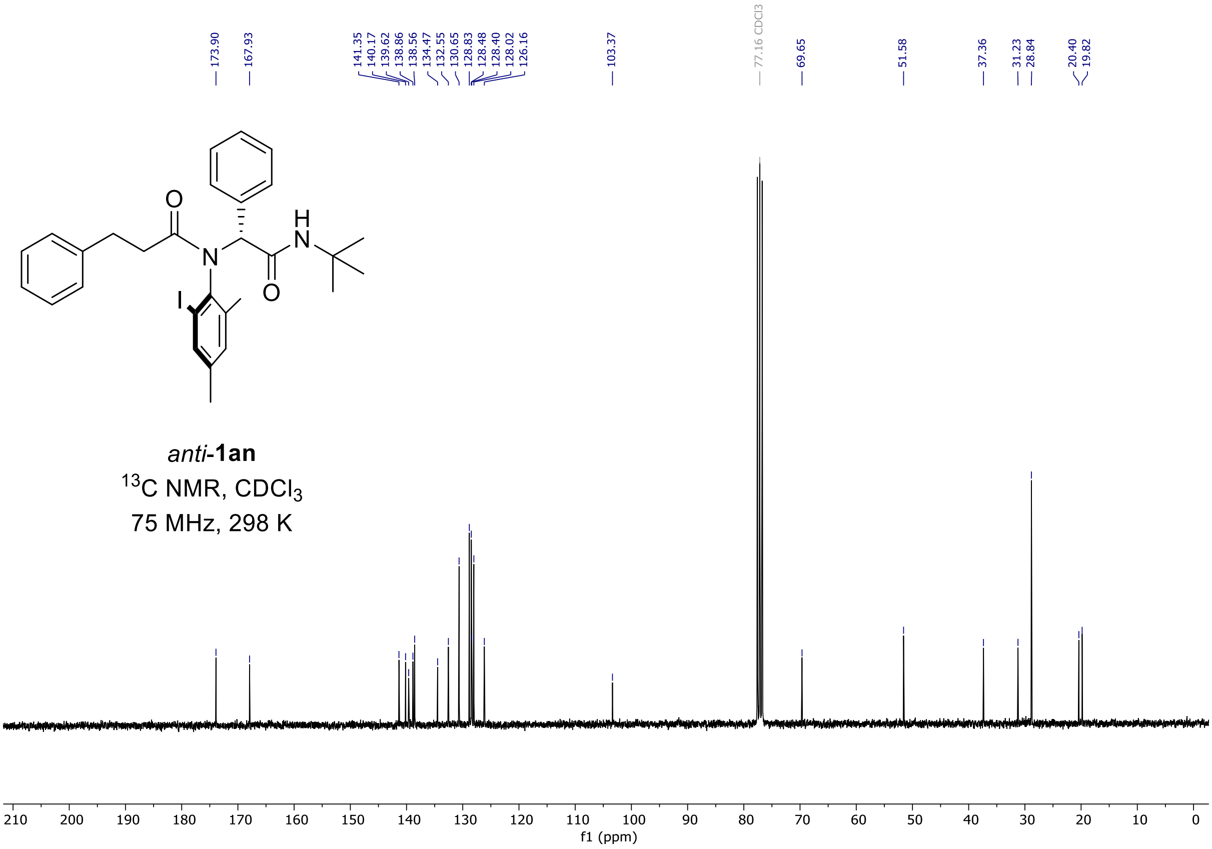

anti-**1an**
^{13}C NMR, $CDCl_3$
75 MHz, 298 K

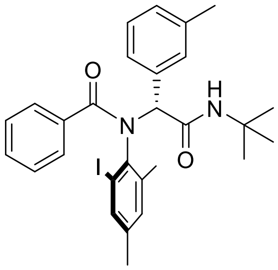

anti-**1ao**
^{1}H NMR, $CDCl_3$
400 MHz, 298 K

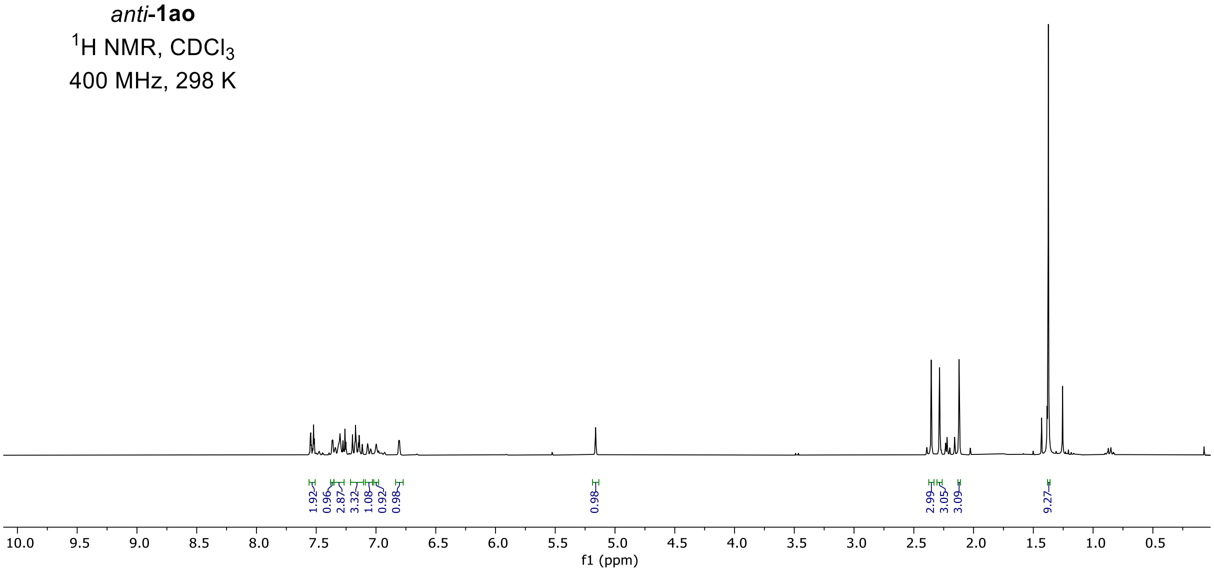

anti-**1ao**
^{13}C NMR, $CDCl_3$
75 MHz, 298 K

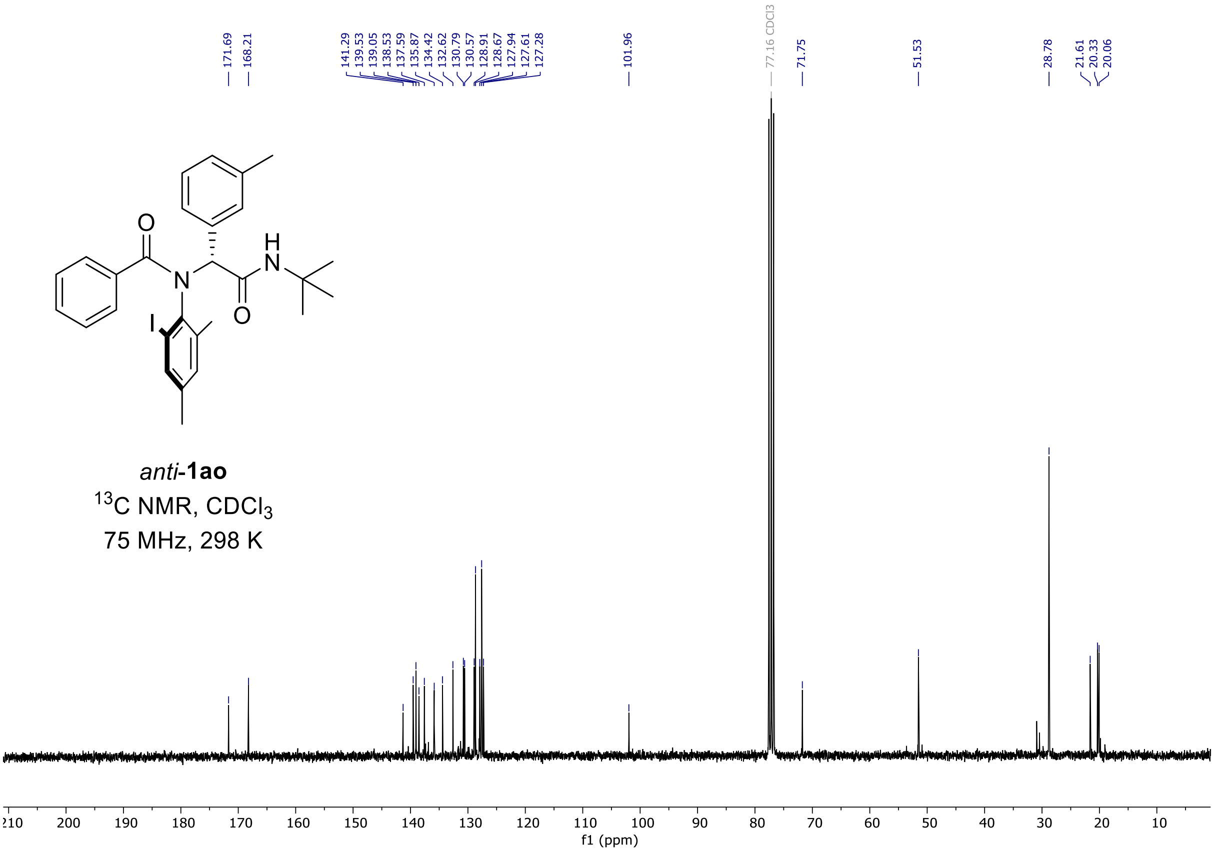

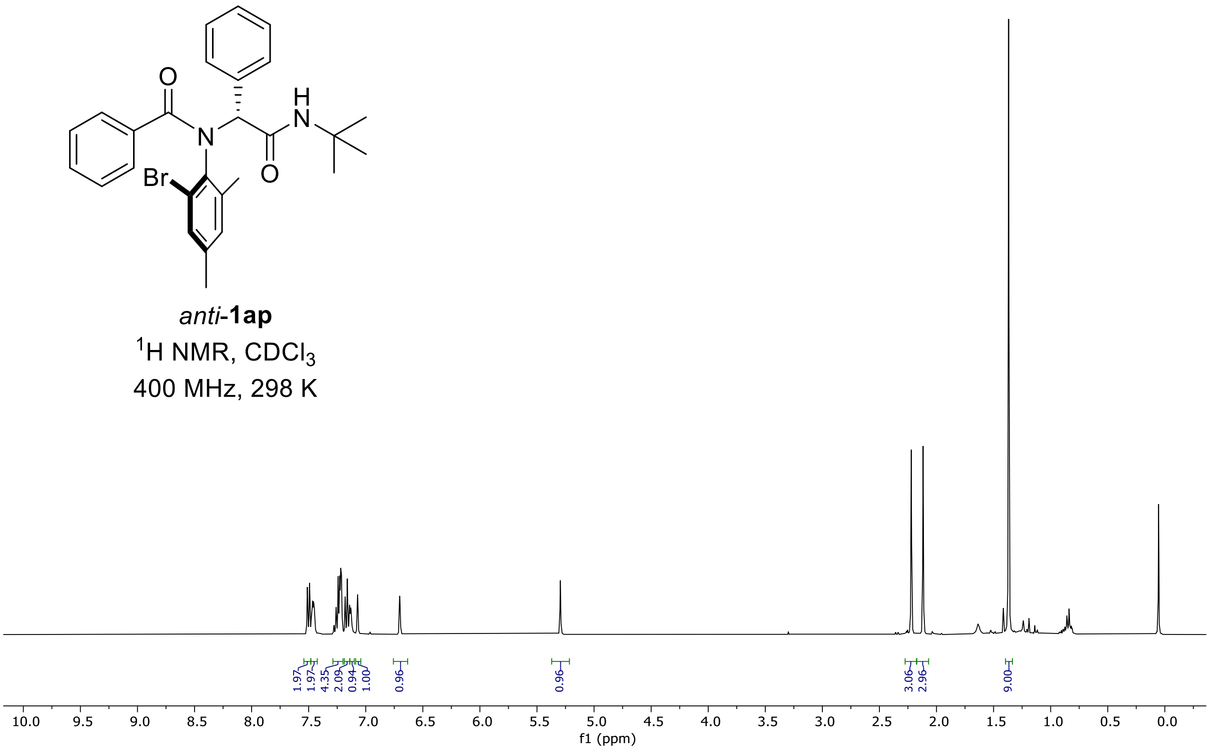
anti-1ap
^{1}H NMR, $CDCl_3$
400 MHz, 298 K
1.97
1.97
4.35
2.09
0.94
1.00
0.96
0.96
3.06
2.96
9.00
f1 (ppm)

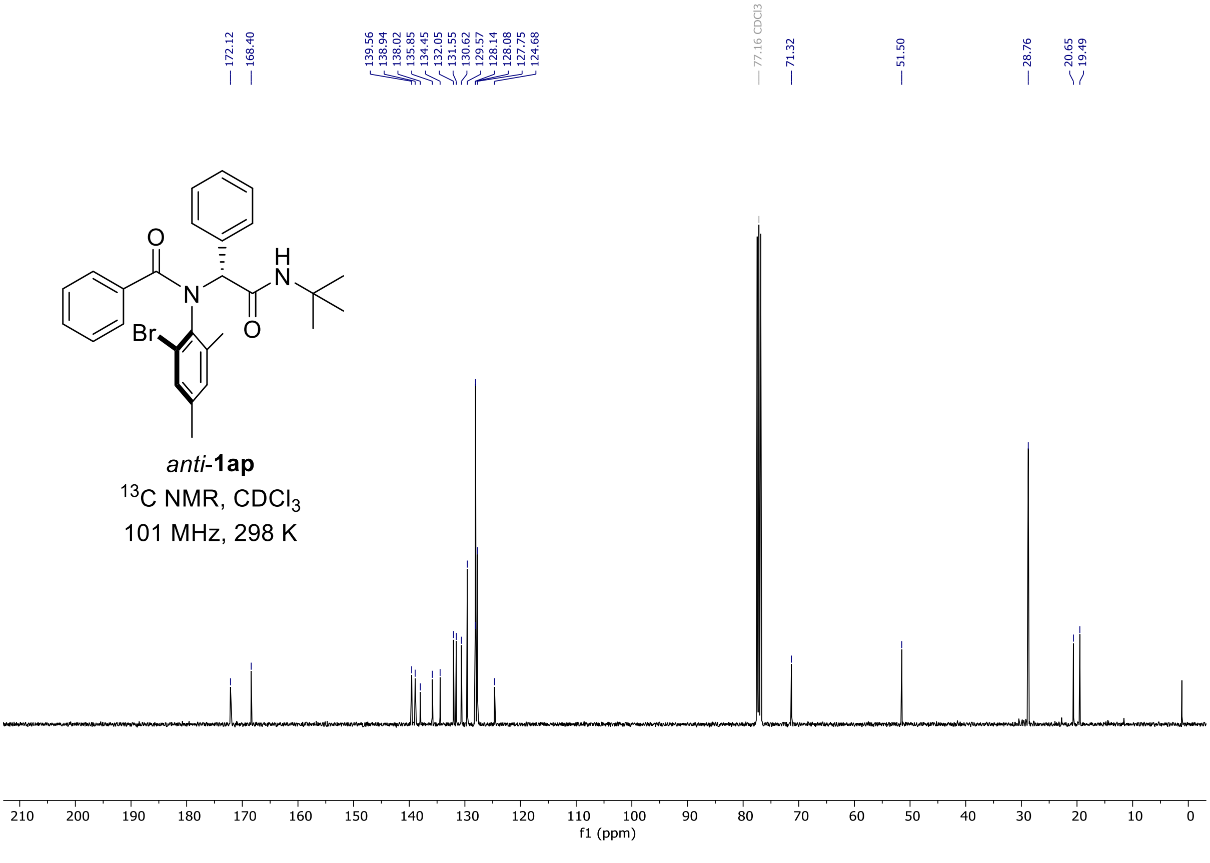
172.12
168.40
139.56
138.94
138.02
135.85
134.45
132.05
131.55
130.62
129.57
128.14
128.08
127.75
124.68
77.16 CDCl3
71.32
51.50
28.76
20.65
19.49
anti-1ap
^{13}C NMR, $CDCl_3$
101 MHz, 298 K
f1 (ppm)

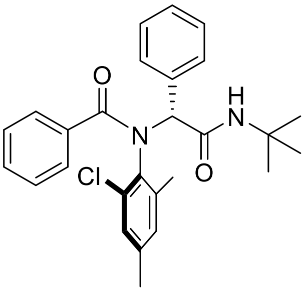

anti-**1aq**
^{1}H NMR, $CDCl_3$
400 MHz, 298 K

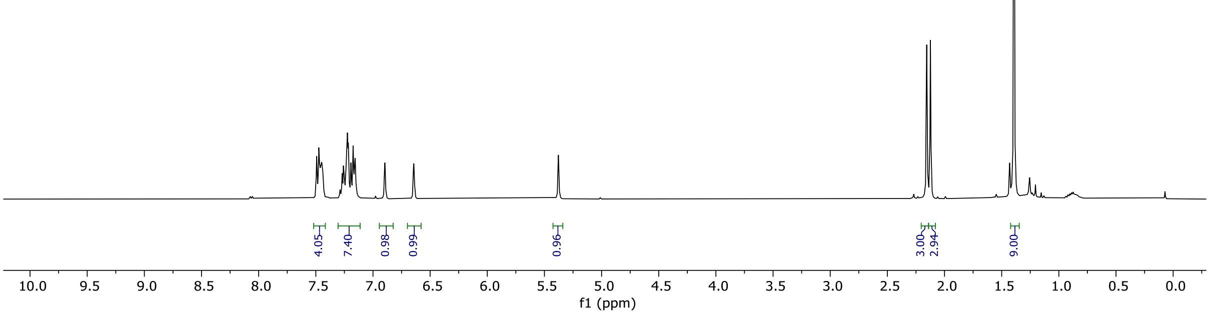

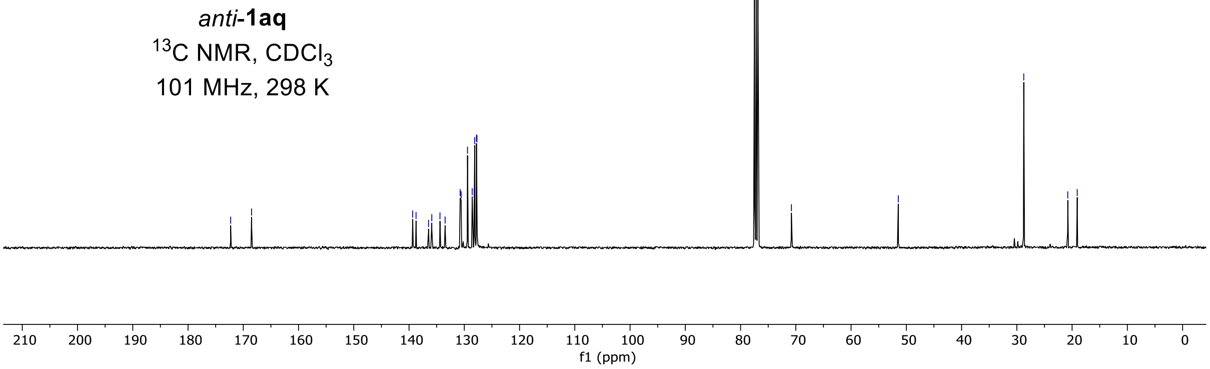

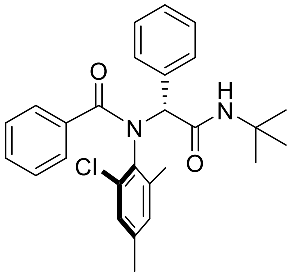

anti-**1aq**
^{13}C NMR, $CDCl_3$
101 MHz, 298 K

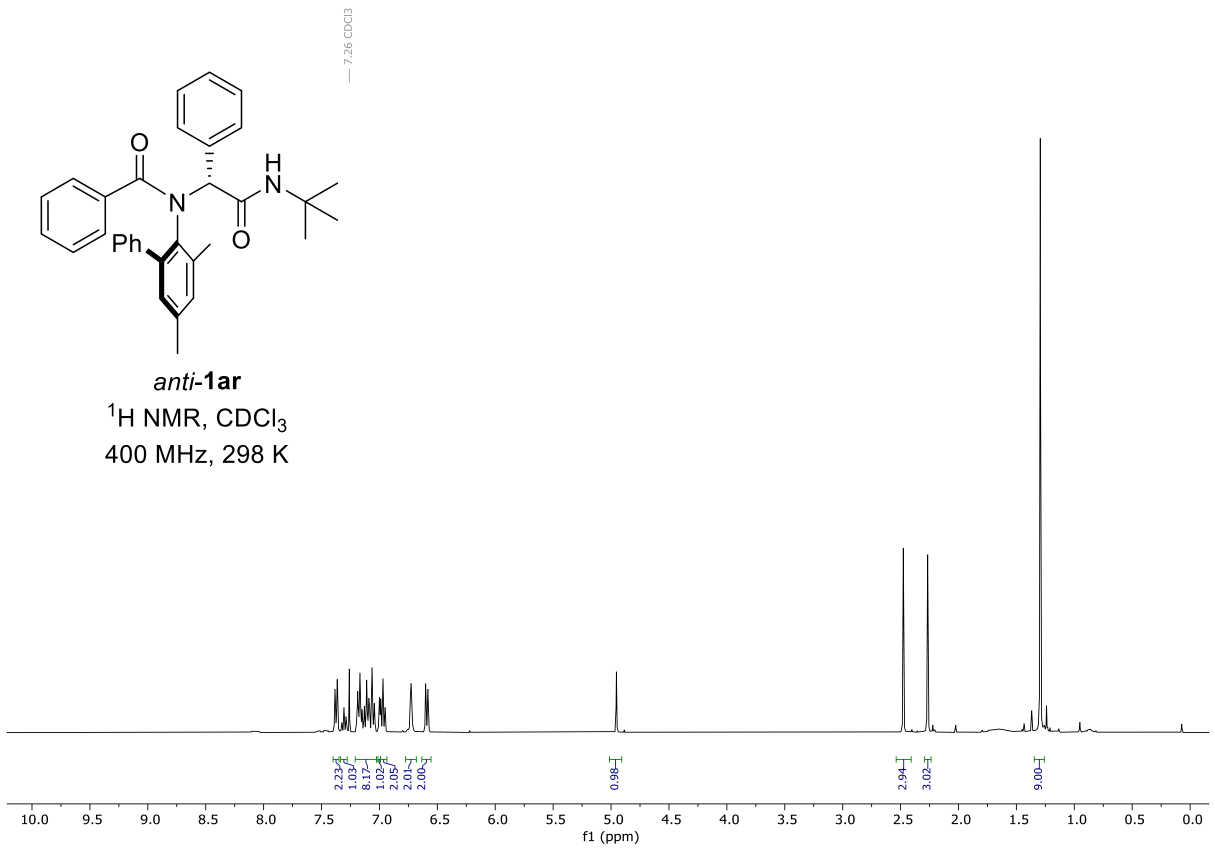
7.26 CDCl3
Ph
anti-**1ar**
¹H NMR, CDCl3
400 MHz, 298 K
2.23
1.03
8.17
1.02
2.05
2.01
2.00
0.98
2.94
3.02
9.00
f1 (ppm)

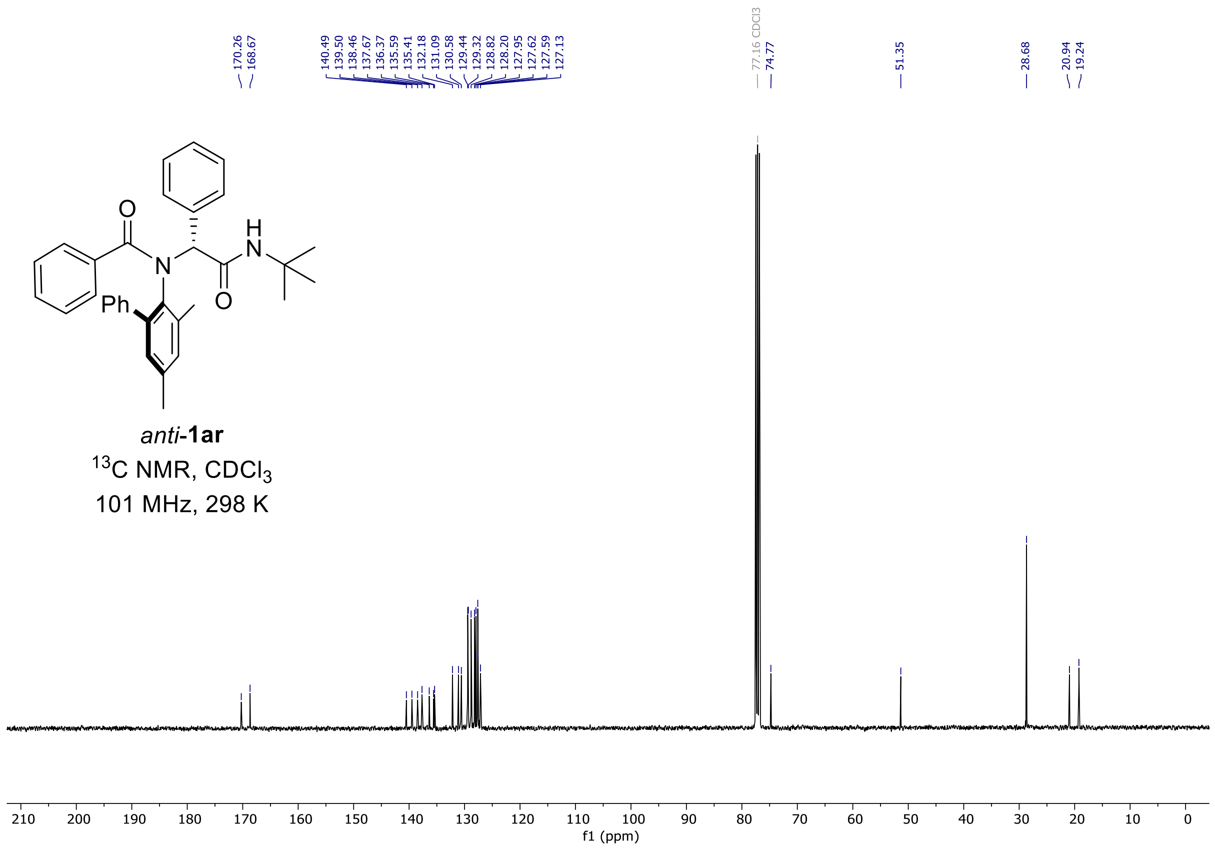
170.26
168.67
140.49
139.50
138.46
137.67
136.37
135.59
135.41
132.18
131.09
130.58
129.44
129.32
128.82
128.20
127.95
127.62
127.59
127.13
77.16 CDCl3
74.77
51.35
28.68
20.94
19.24
Ph
anti-**1ar**
¹³C NMR, CDCl3
101 MHz, 298 K
f1 (ppm)

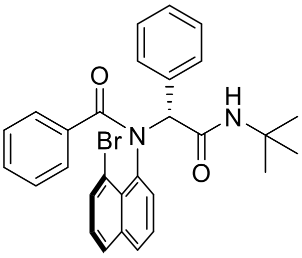

anti-**1as**

^{1}H NMR, $CDCl_3$

400 MHz, 298 K

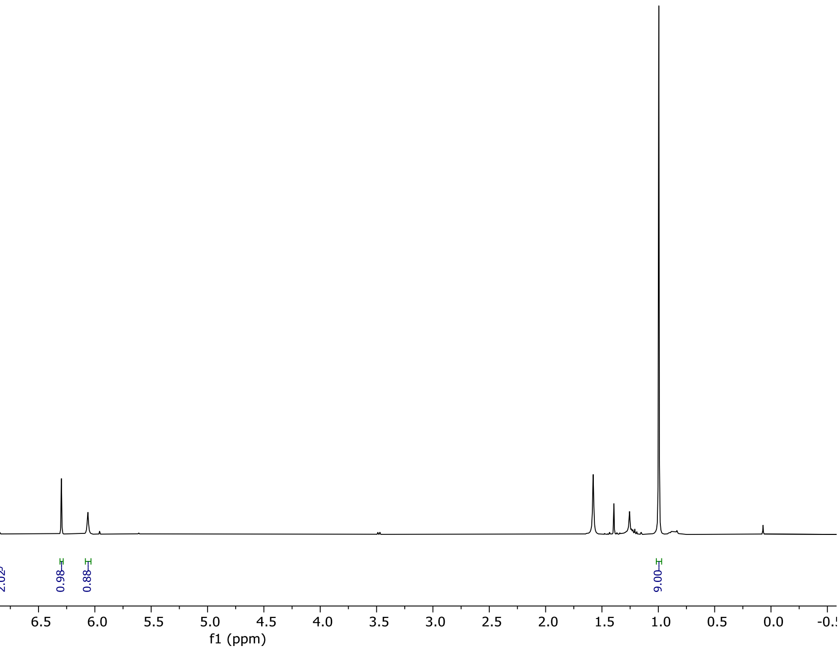

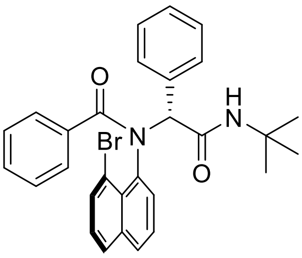

anti-**1as**

^{13}C NMR, $CDCl_3$

101 MHz, 298 K

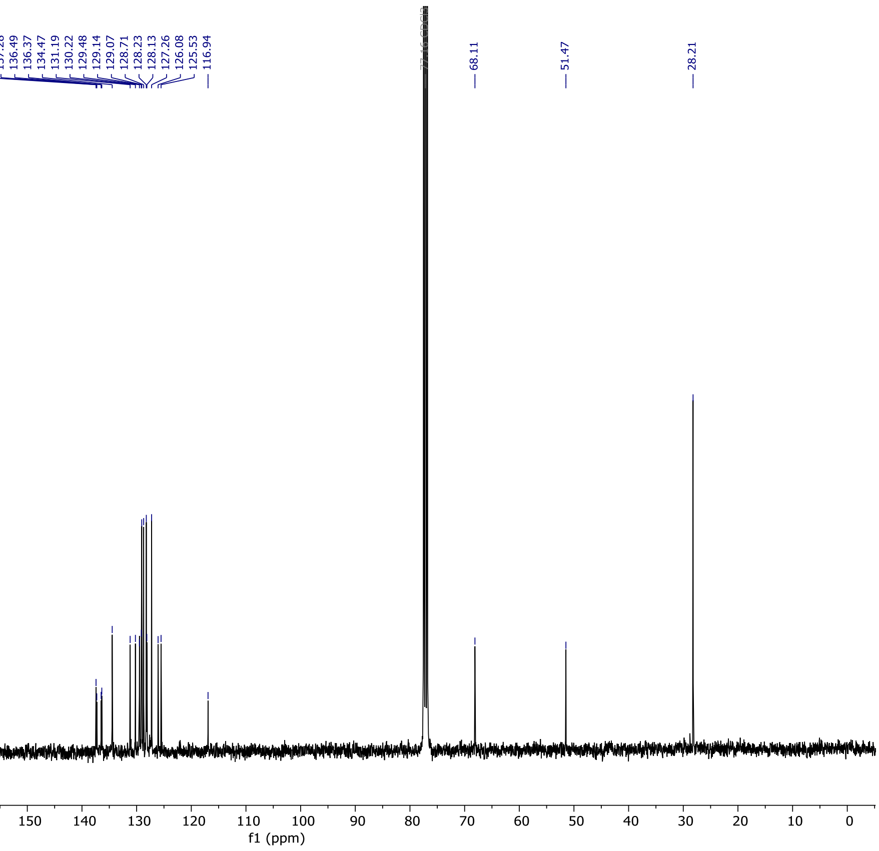

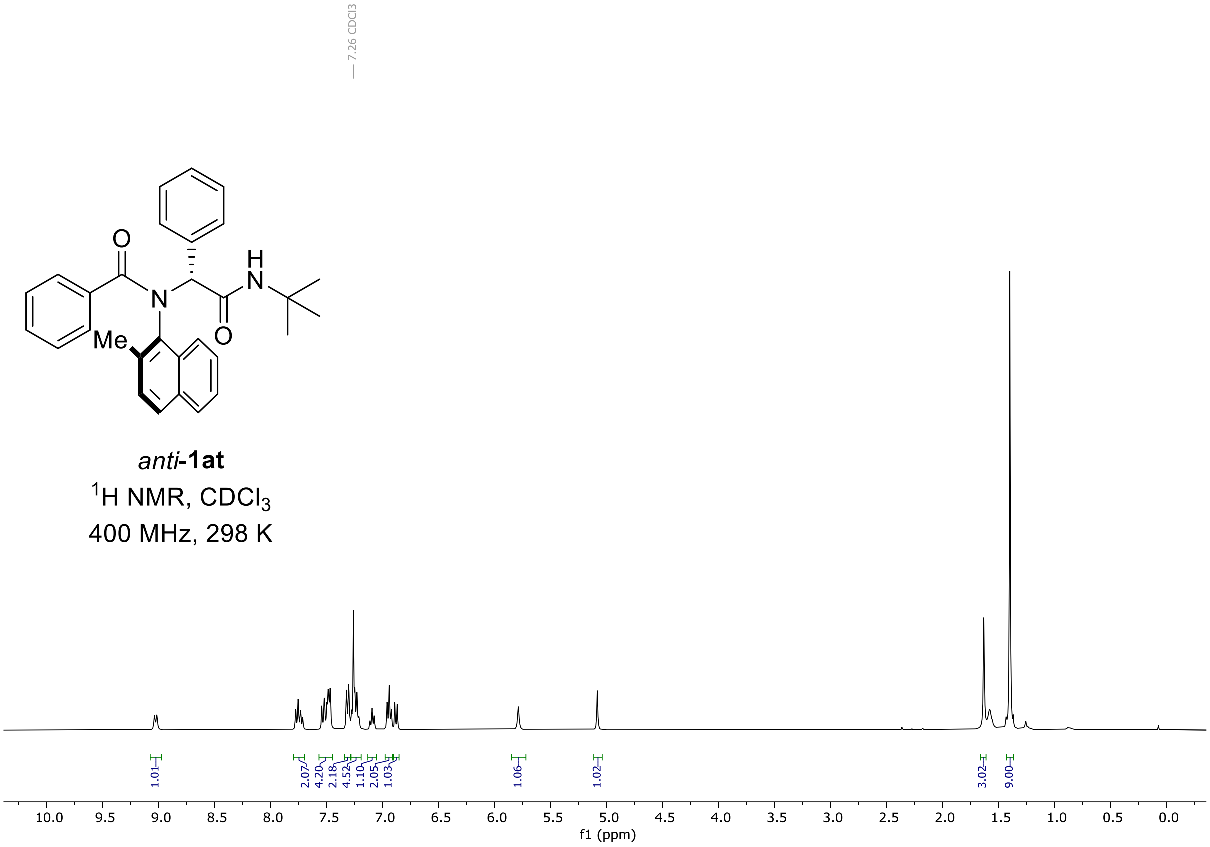

O
H
N
N
Me
O
anti-1at
¹H NMR, CDCl3
400 MHz, 298 K
7.26 CDCl3
1.01
2.07
4.20
2.18
4.52
1.10
2.05
1.03
1.06
1.02
3.02
9.00
10.0 9.5 9.0 8.5 8.0 7.5 7.0 6.5 6.0 5.5 5.0 4.5 4.0 3.5 3.0 2.5 2.0 1.5 1.0 0.5 0.0
f1 (ppm)

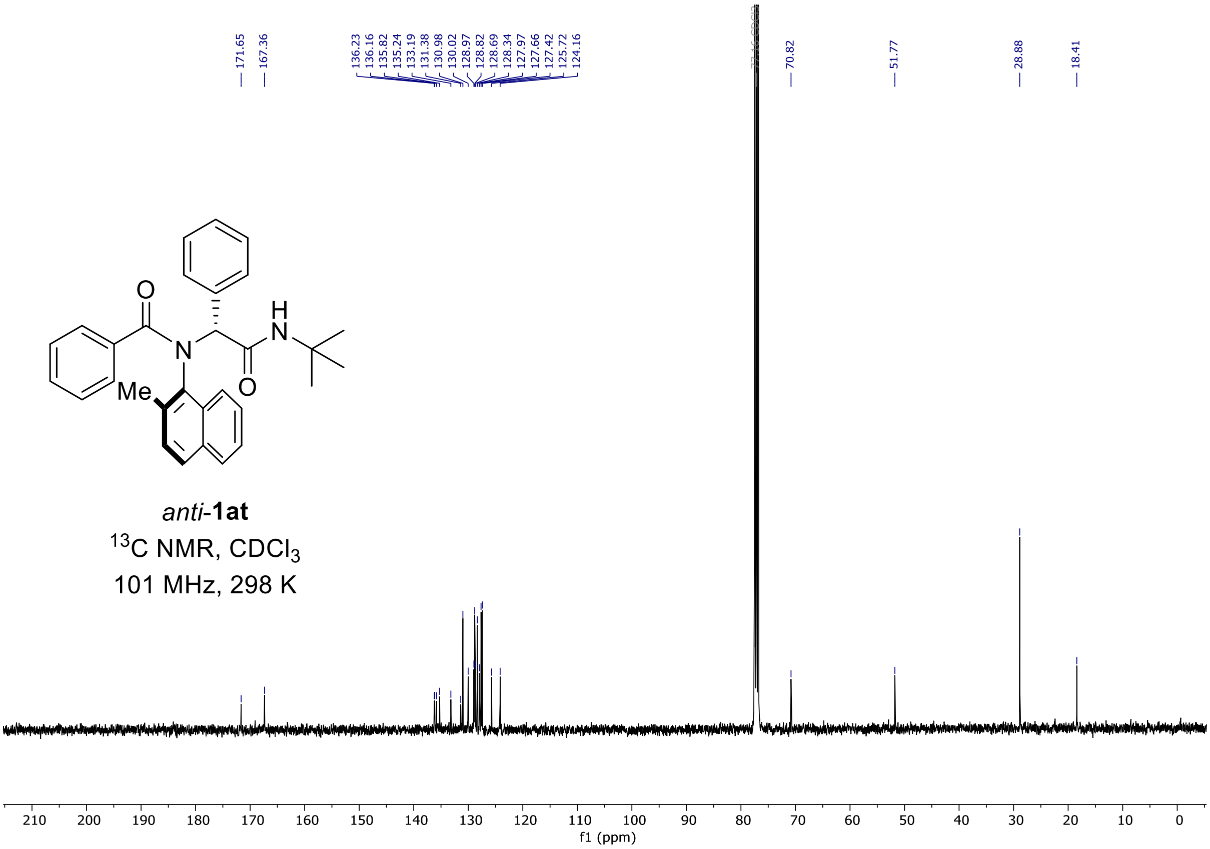

171.65
167.36
136.23
136.16
135.82
135.24
133.19
131.38
130.98
130.02
128.97
128.82
128.69
128.34
127.97
127.66
127.42
125.72
124.16
70.82
51.77
28.88
18.41
O
H
N
N
Me
O
anti-1at
¹³C NMR, CDCl3
101 MHz, 298 K
210 200 190 180 170 160 150 140 130 120 110 100 90 80 70 60 50 40 30 20 10 0
f1 (ppm)

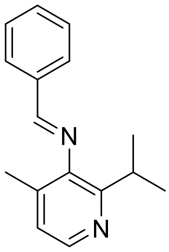

S2
^{1}H NMR, $CDCl_3$
400 MHz, 298 K

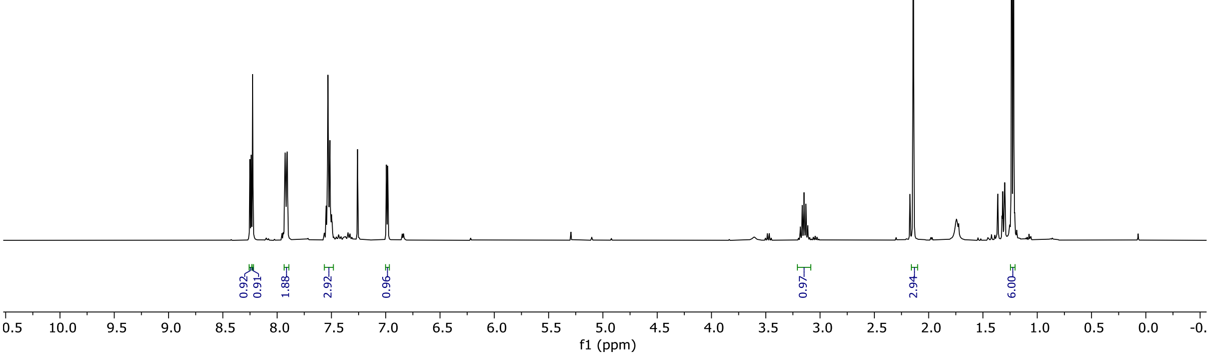

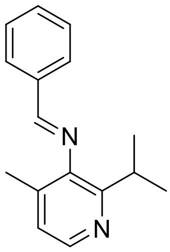

S2
^{13}C NMR, $CDCl_3$
101 MHz, 298 K

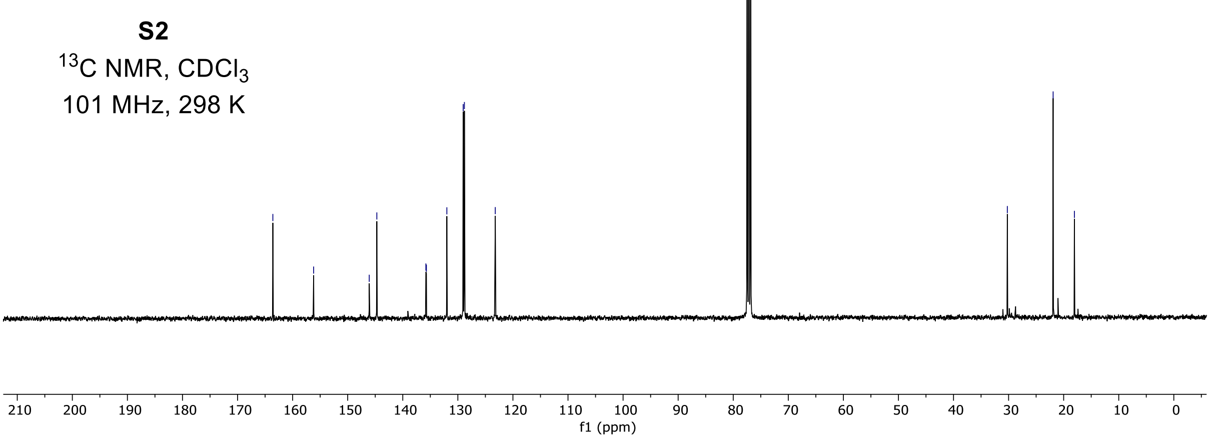

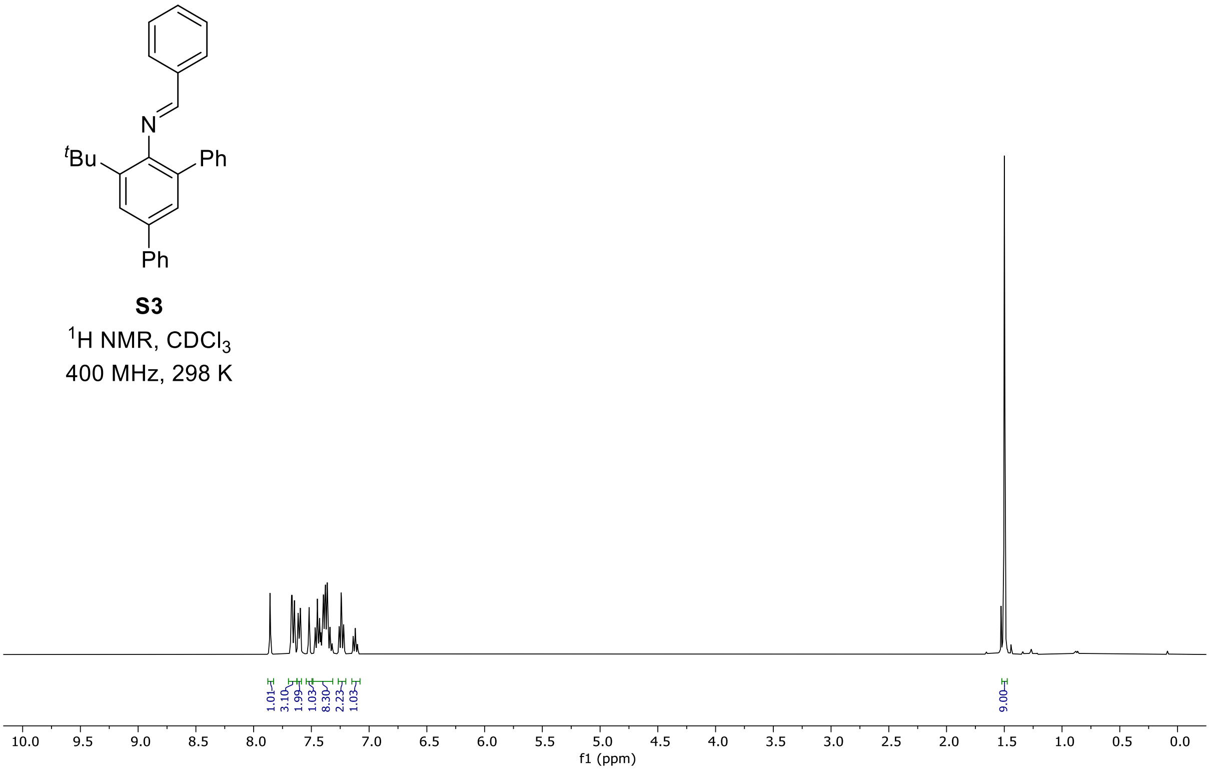
N
tBu
Ph
Ph
S3
1H NMR, CDCl3
400 MHz, 298 K
1.01
3.10
1.99
1.03
8.30
2.23
1.03
9.00
10.0 9.5 9.0 8.5 8.0 7.5 7.0 6.5 6.0 5.5 5.0 4.5 4.0 3.5 3.0 2.5 2.0 1.5 1.0 0.5 0.0
f1 (ppm)

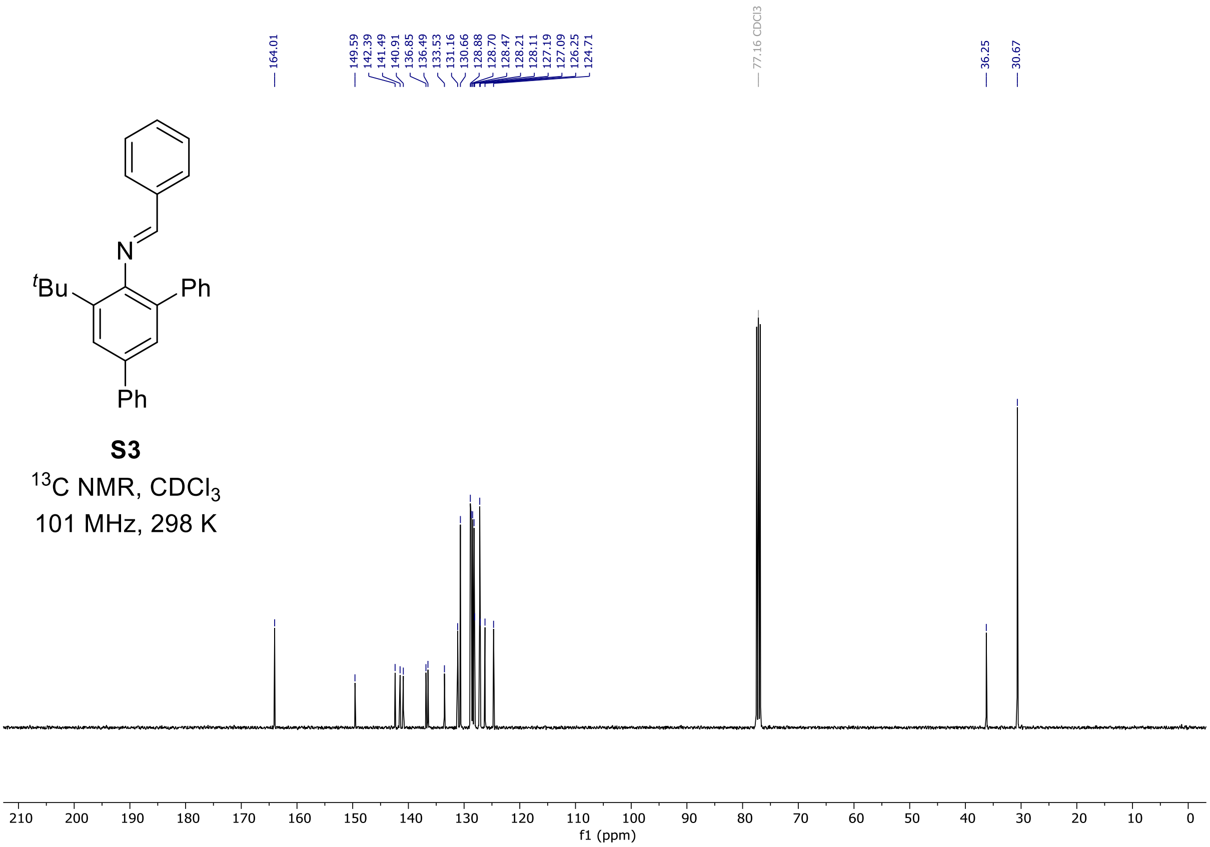
164.01
149.59
142.39
141.49
140.91
136.85
136.49
133.53
131.16
130.66
128.88
128.70
128.47
128.21
128.11
127.19
127.09
126.25
124.71
77.16 CDCl3
36.25
30.67
N
tBu
Ph
Ph
S3
13C NMR, CDCl3
101 MHz, 298 K
210 200 190 180 170 160 150 140 130 120 110 100 90 80 70 60 50 40 30 20 10 0
f1 (ppm)

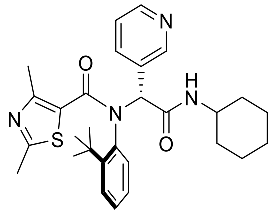

anti-**1aw**
^{1}H NMR, $CDCl_3$
400 MHz, 298 K

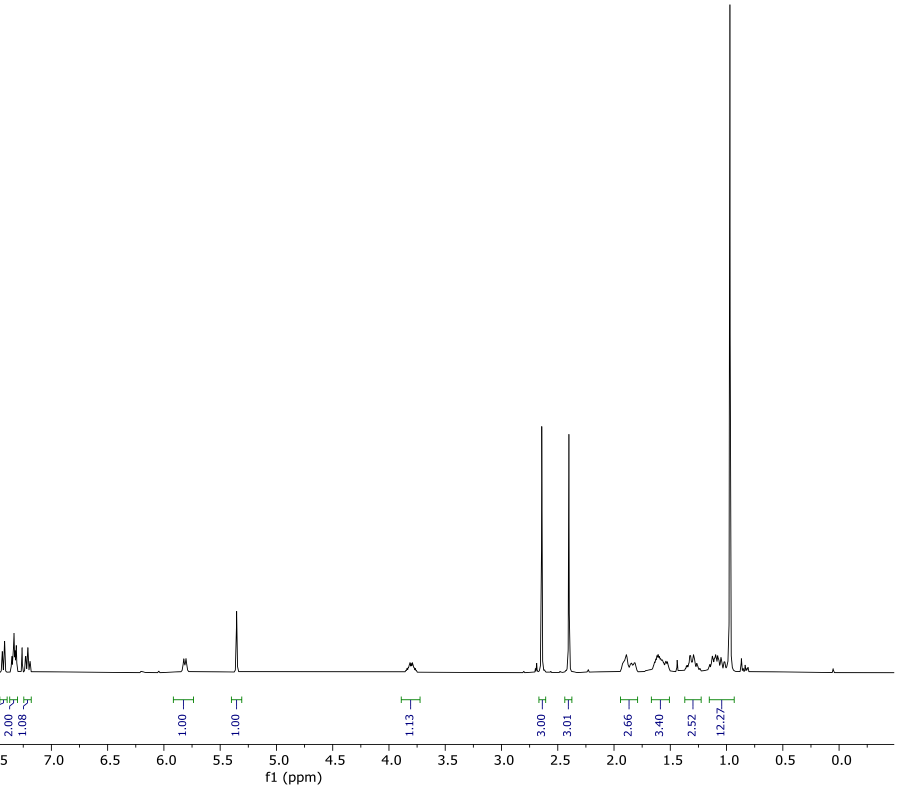

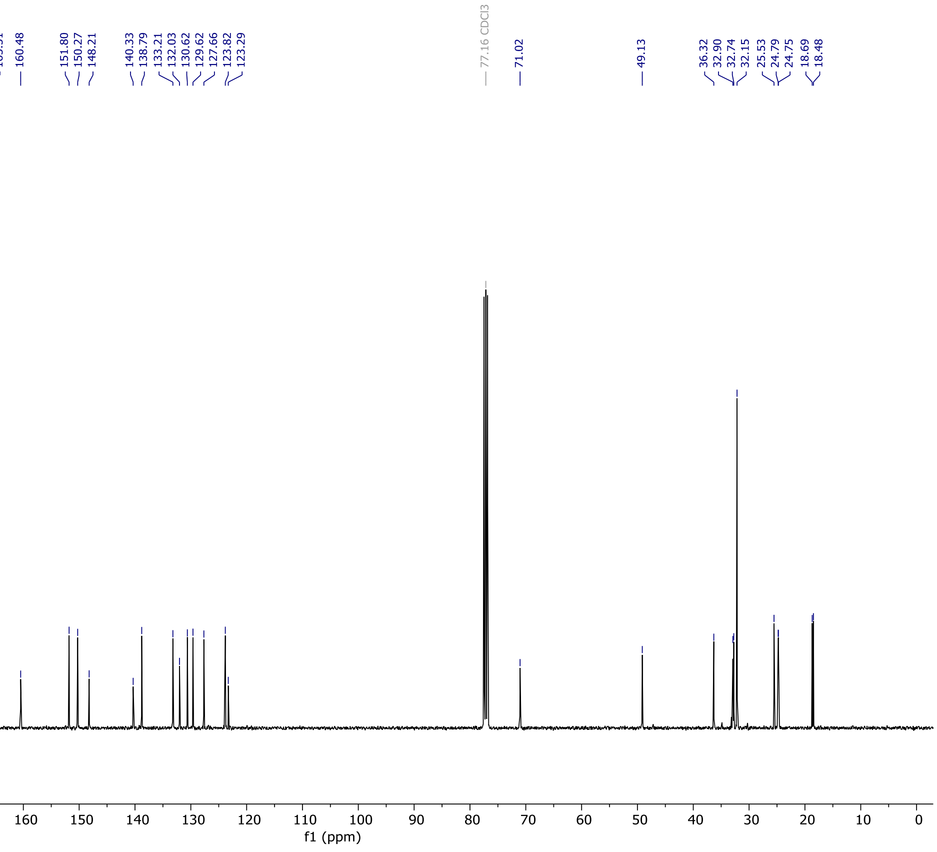

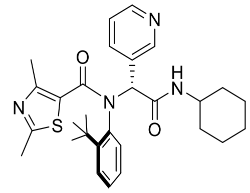

anti-**1aw**
^{13}C NMR, $CDCl_3$
101 MHz, 298 K

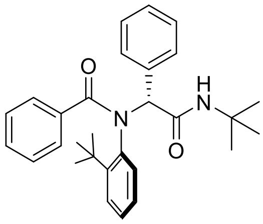

syn-**1a**
^{1}H NMR, $CDCl_3$
300 MHz, 298 K

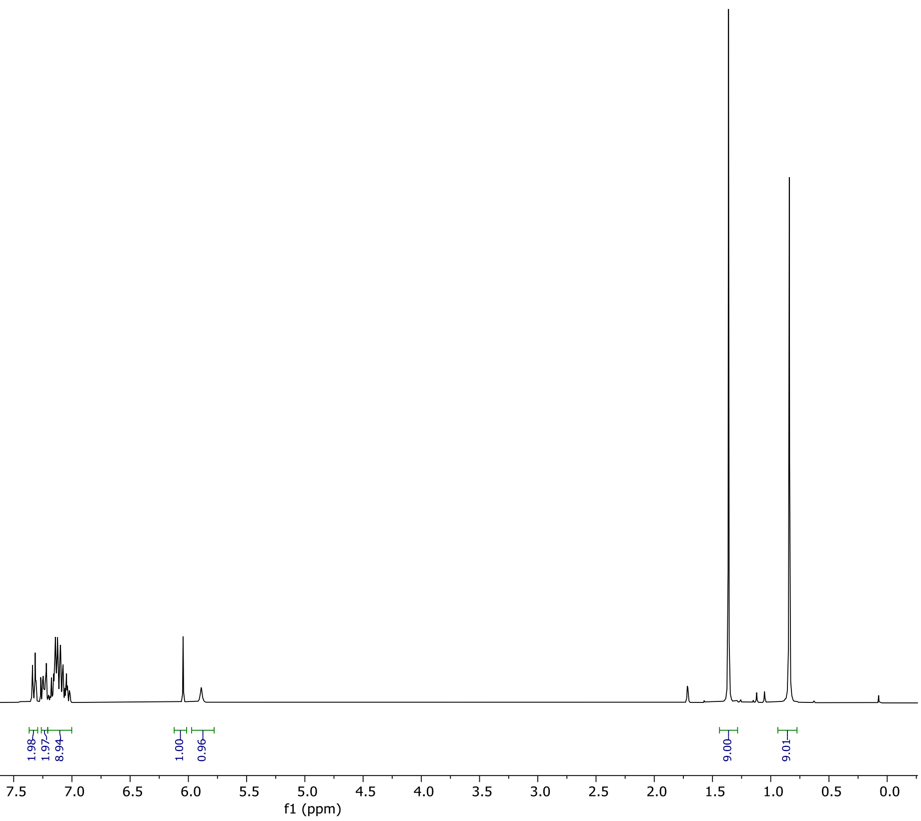

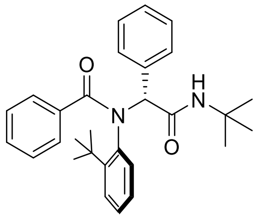

syn-**1a**
^{13}C NMR, $CDCl_3$
75 MHz, 298 K

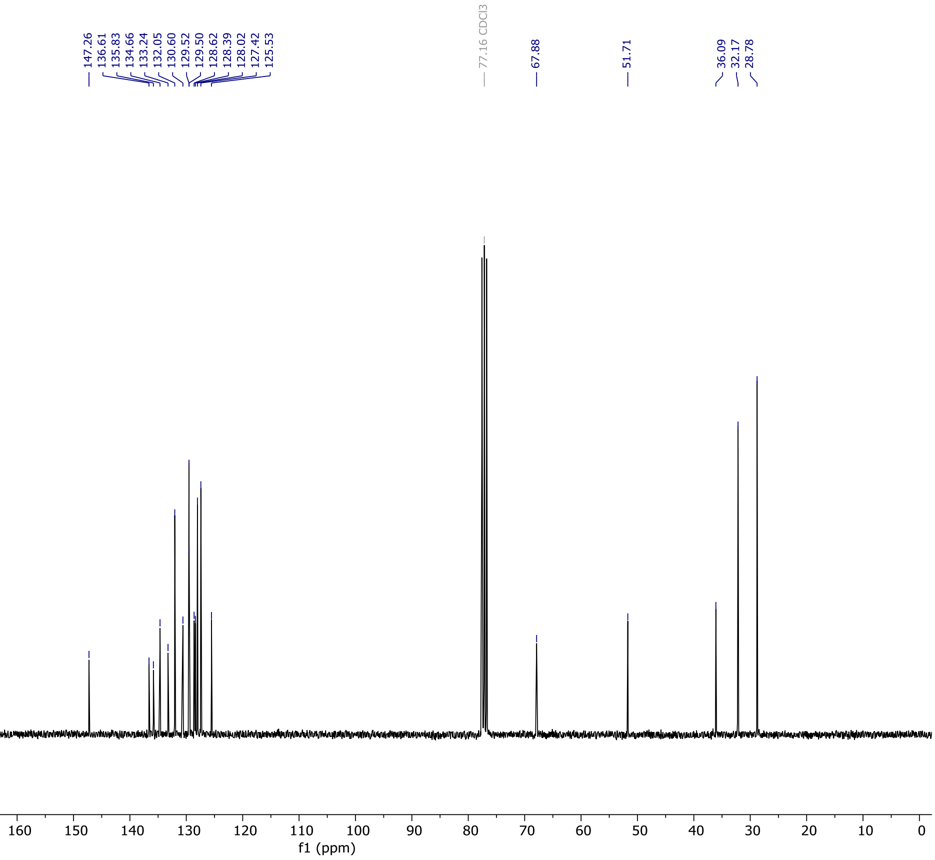

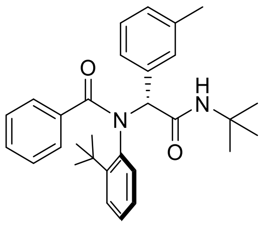

syn-**1f**
^{1}H NMR, $CDCl_3$
400 MHz, 298 K

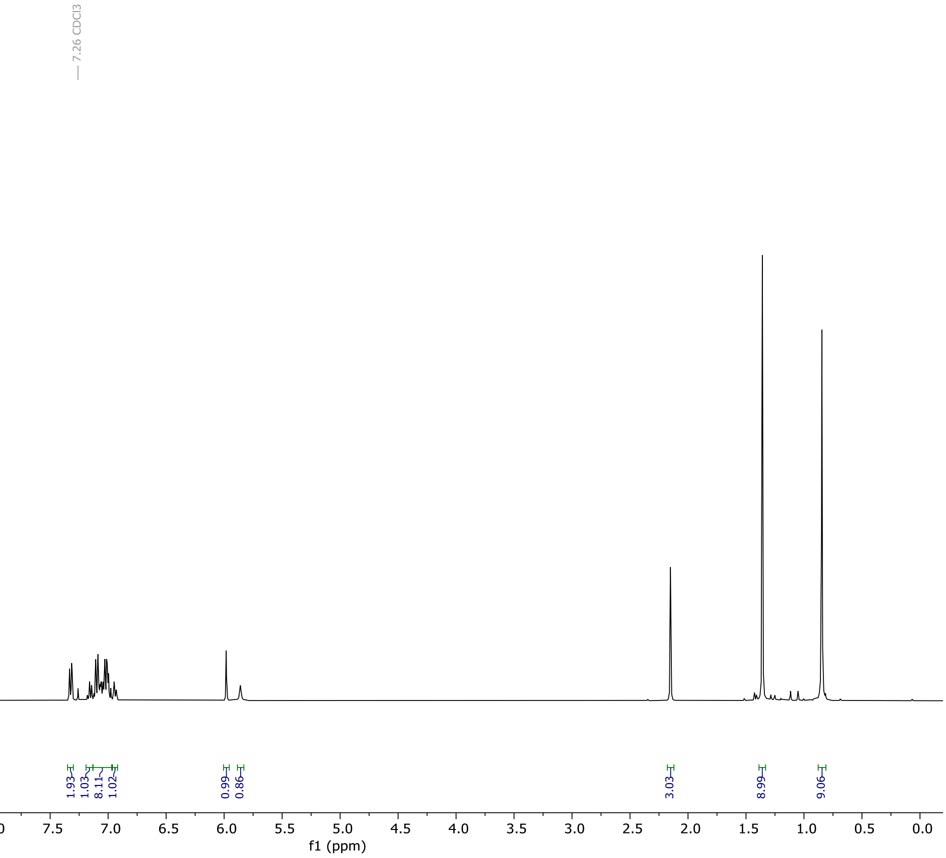

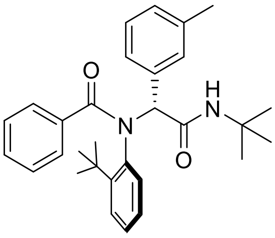

syn-**1f**
^{13}C NMR, $CDCl_3$
101 MHz, 298 K

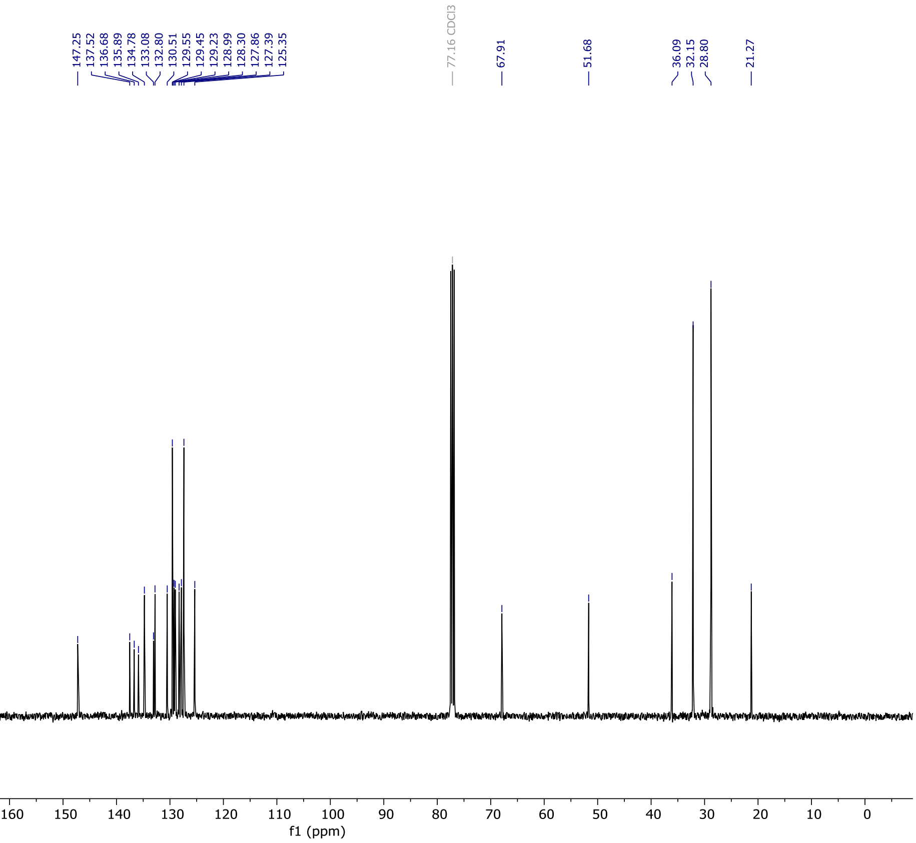

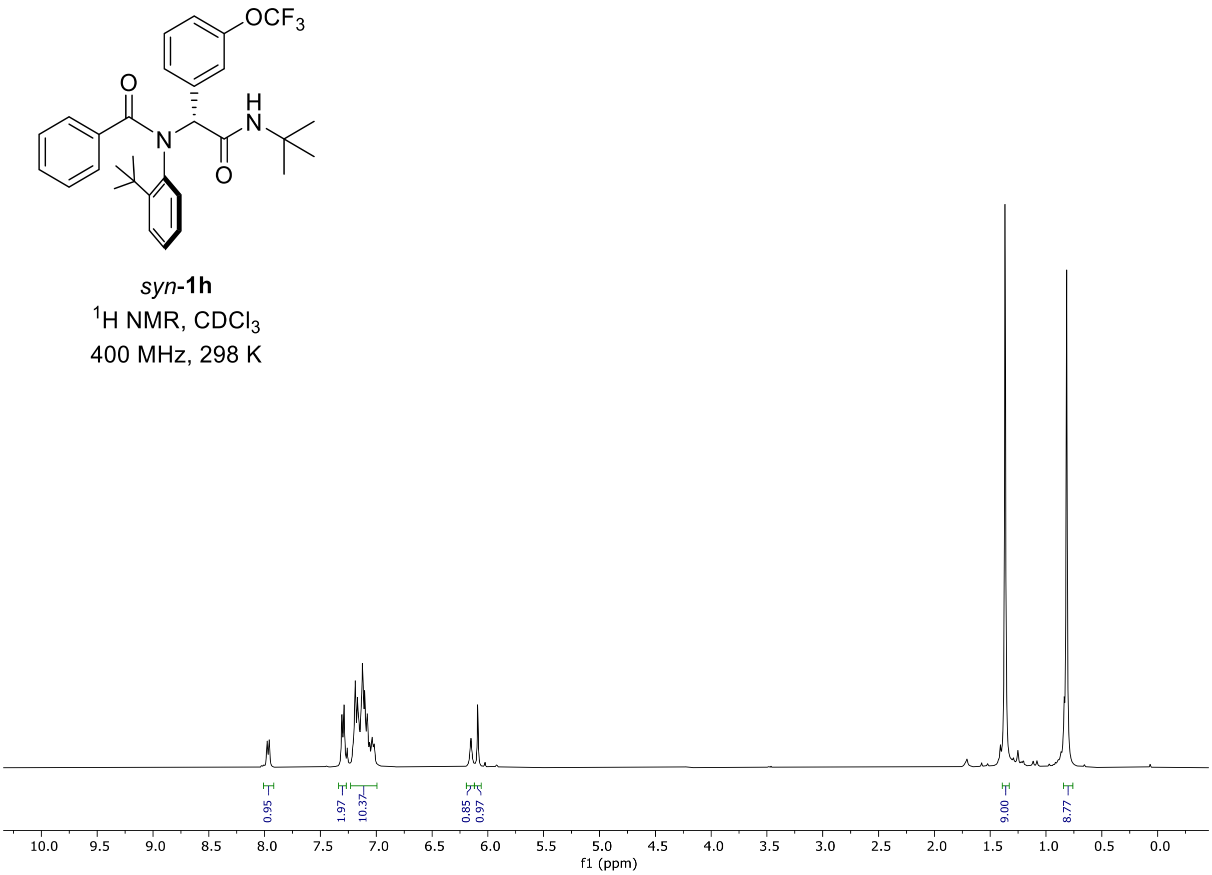
OCF3
O
H
N
N
O
syn-1h
1H NMR, CDCl3
400 MHz, 298 K
0.95
1.97
10.37
0.85
0.97
9.00
8.77
10.0 9.5 9.0 8.5 8.0 7.5 7.0 6.5 6.0 5.5 5.0 4.5 4.0 3.5 3.0 2.5 2.0 1.5 1.0 0.5 0.0
f1 (ppm)

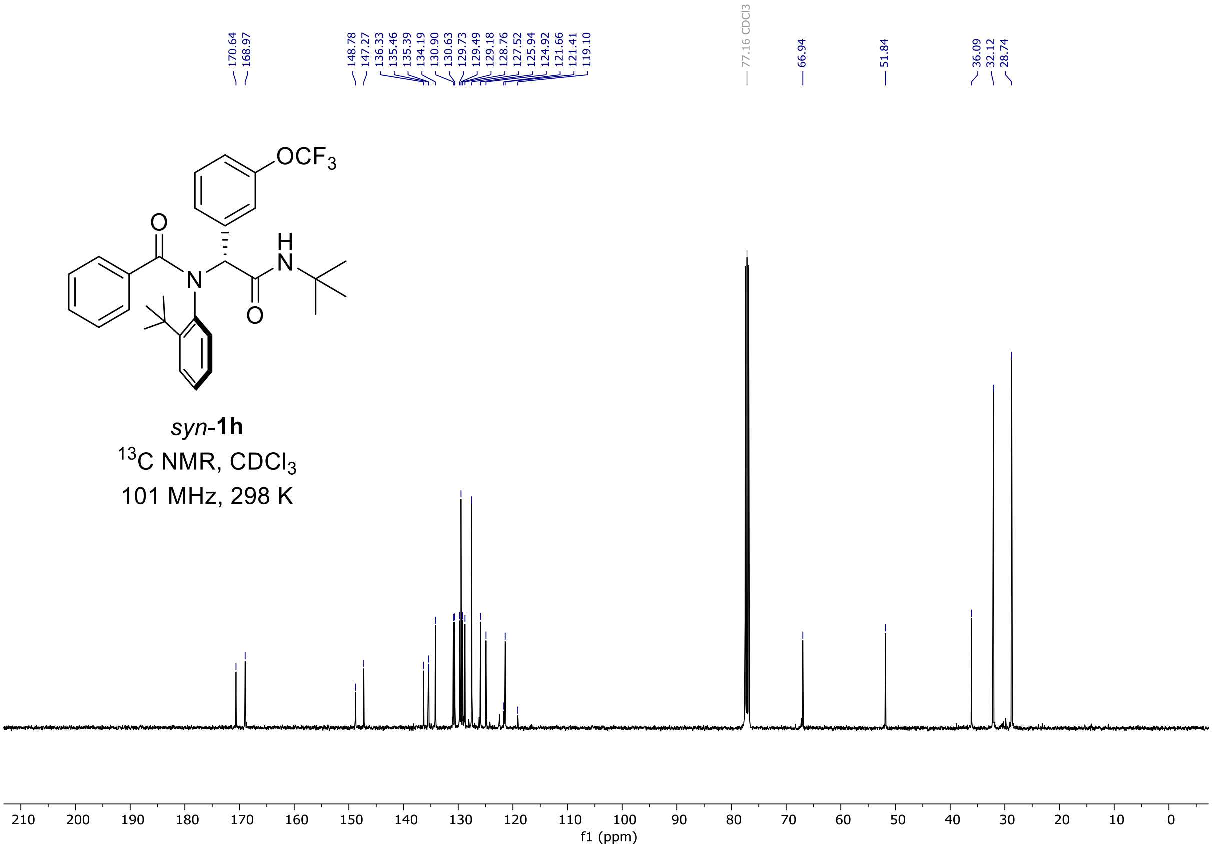
170.64
168.97
148.78
147.27
136.33
135.46
135.39
134.19
130.90
130.63
129.73
129.49
129.18
128.76
127.52
125.94
124.92
121.66
121.41
119.10
77.16 CDCl3
66.94
51.84
36.09
32.12
28.74
OCF3
O
H
N
N
O
syn-1h
13C NMR, CDCl3
101 MHz, 298 K
210 200 190 180 170 160 150 140 130 120 110 100 90 80 70 60 50 40 30 20 10 0
f1 (ppm)

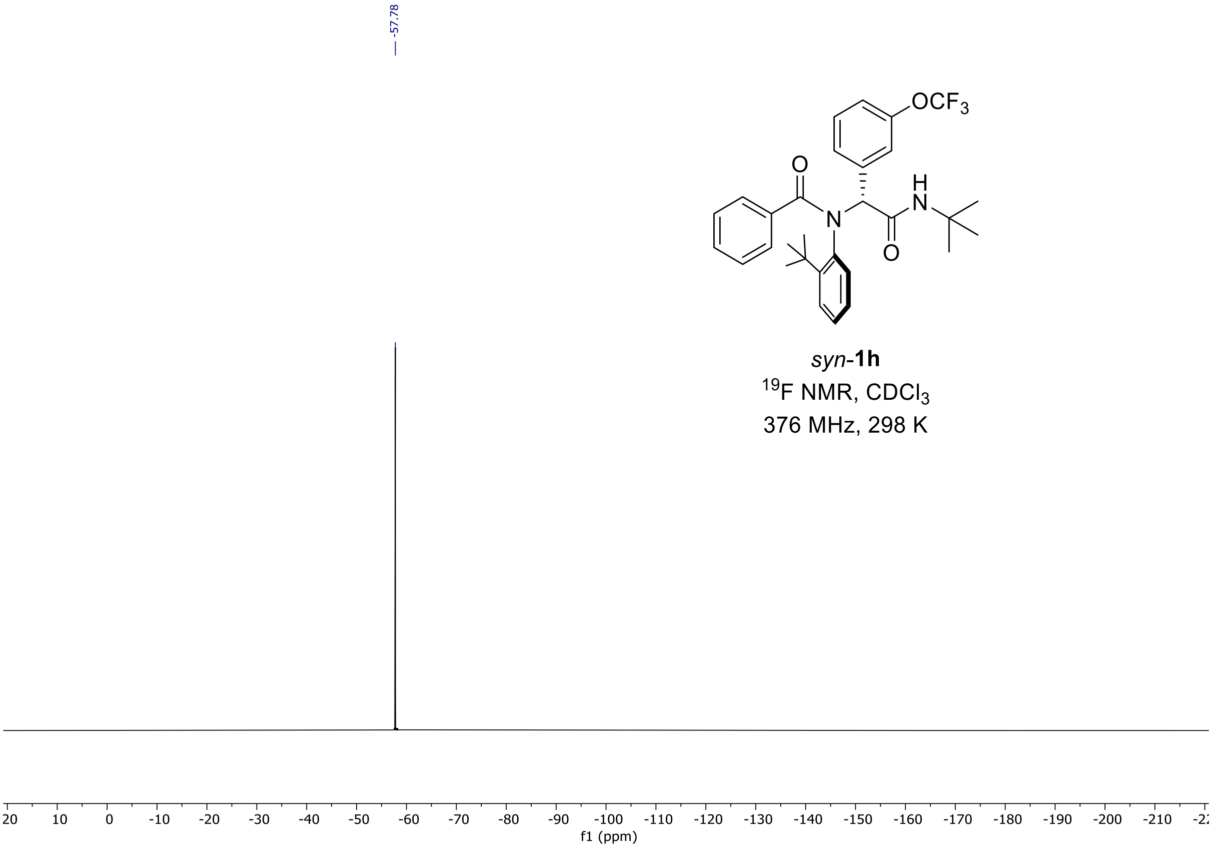

-57.78
OCF3
O
H
N
N
O
syn-1h
19F NMR, CDCl3
376 MHz, 298 K
20 10 0 -10 -20 -30 -40 -50 -60 -70 -80 -90 -100 -110 -120 -130 -140 -150 -160 -170 -180 -190 -200 -210
f1 (ppm)

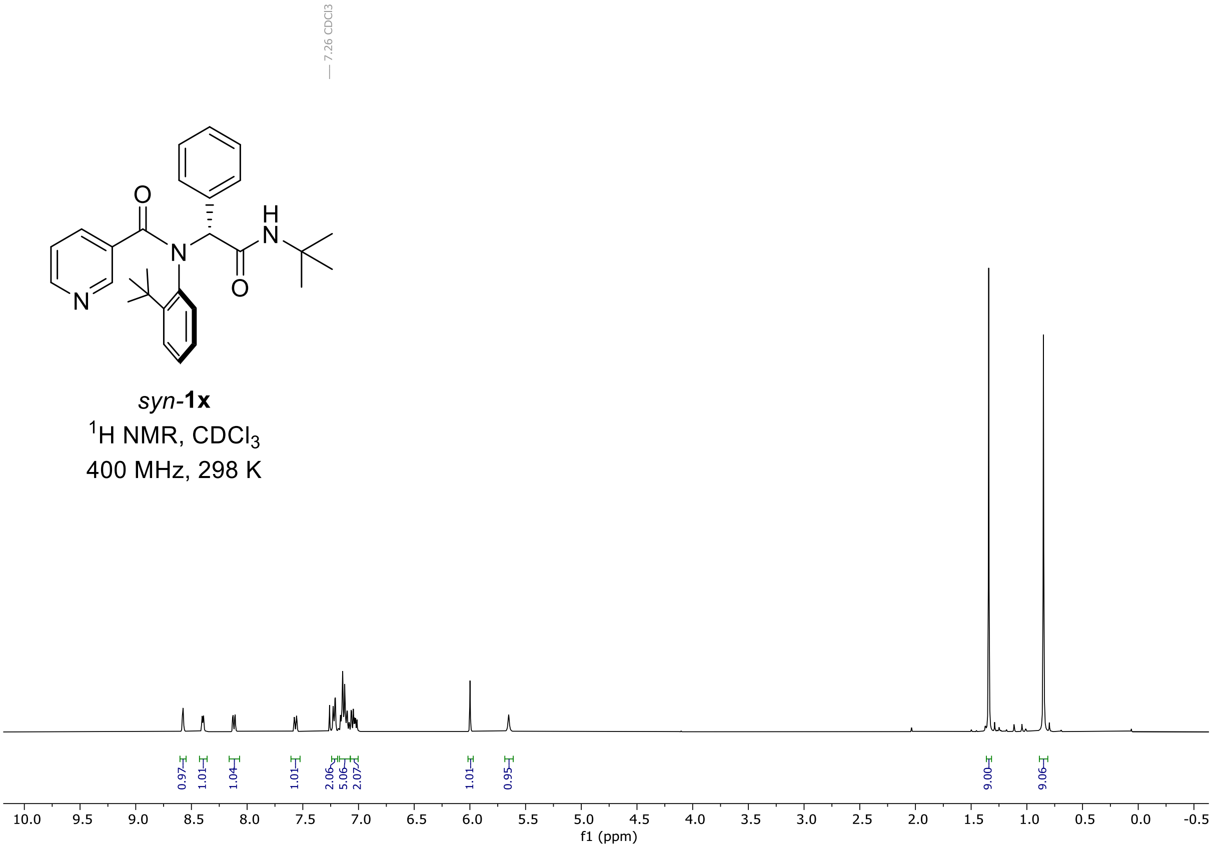

syn-**1x**
^{1}H NMR, $CDCl_3$
400 MHz, 298 K

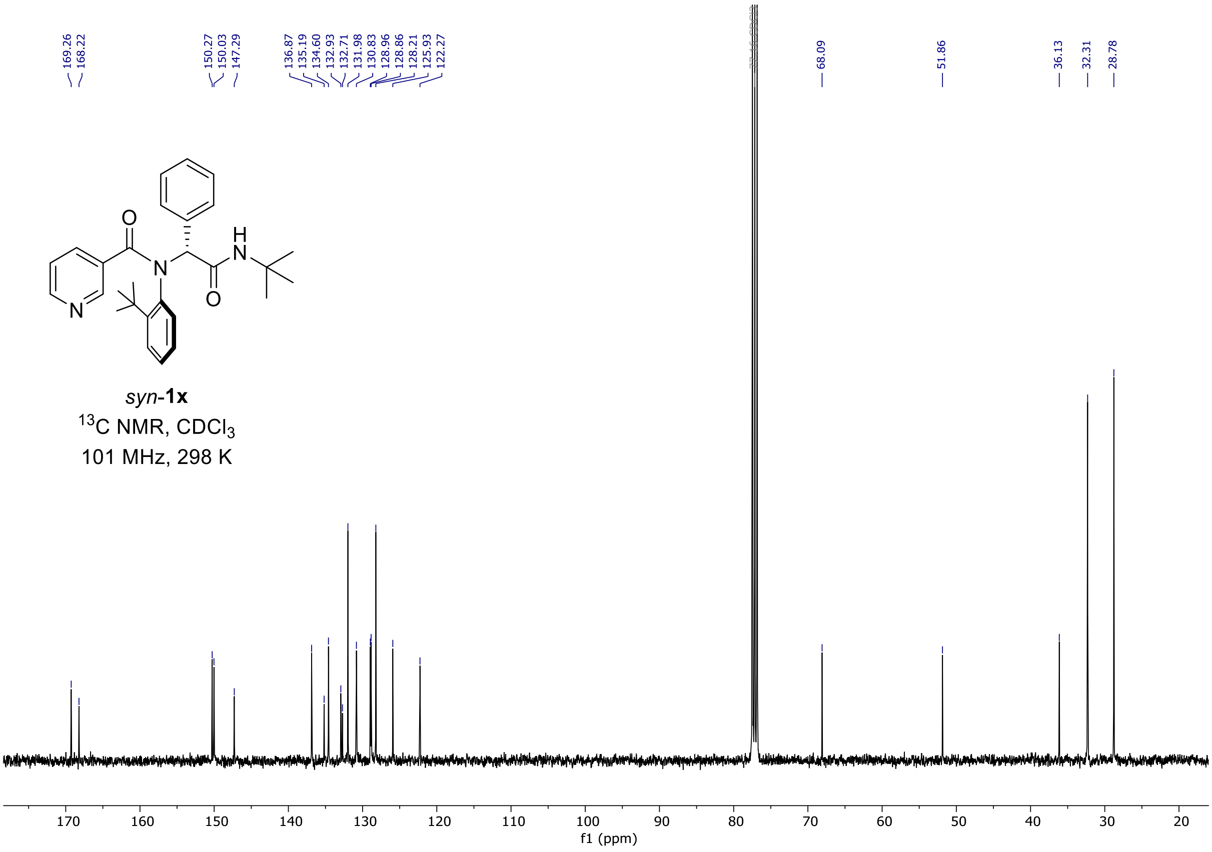

syn-**1x**
^{13}C NMR, $CDCl_3$
101 MHz, 298 K

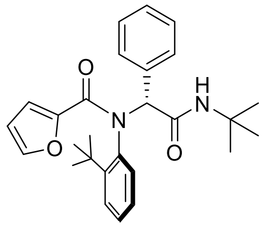

syn-**1y**
^1H NMR, $CDCl_3$
400 MHz, 298 K

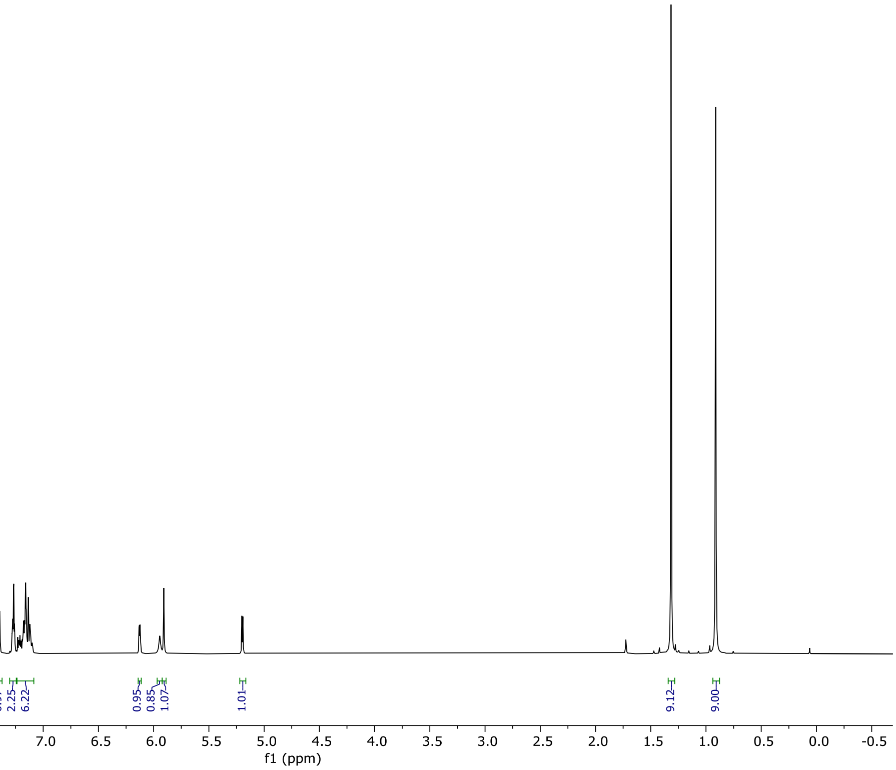

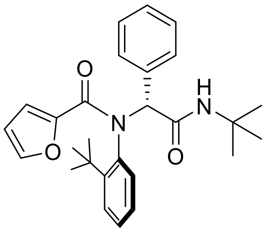

syn-**1y**
^{13}C NMR, $CDCl_3$
101 MHz, 298 K

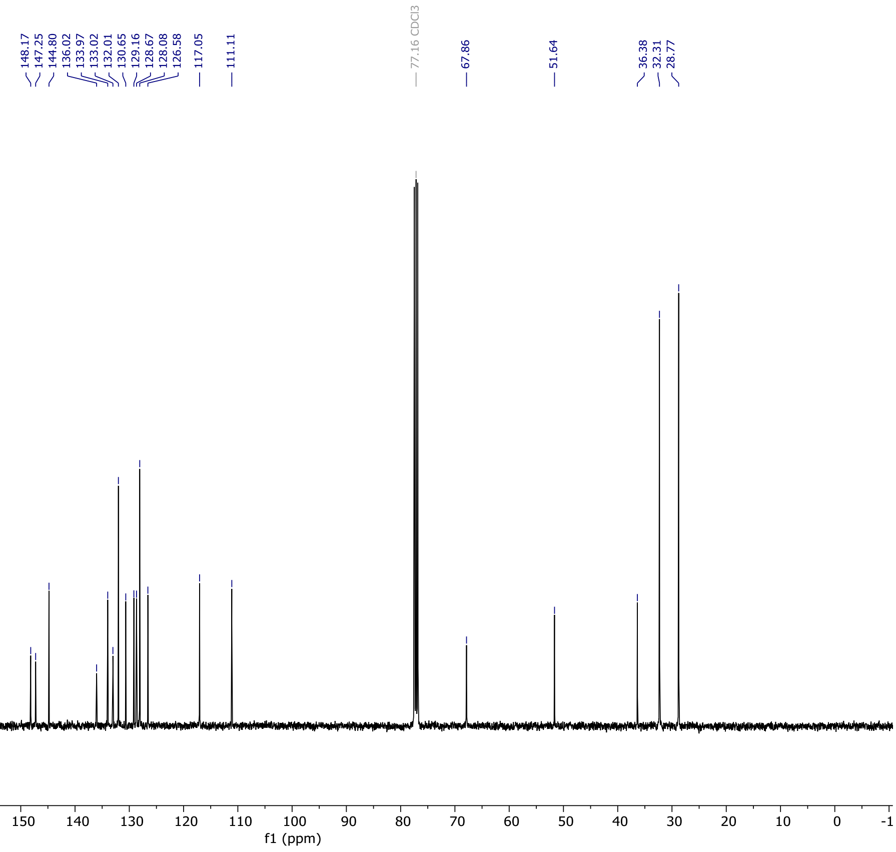

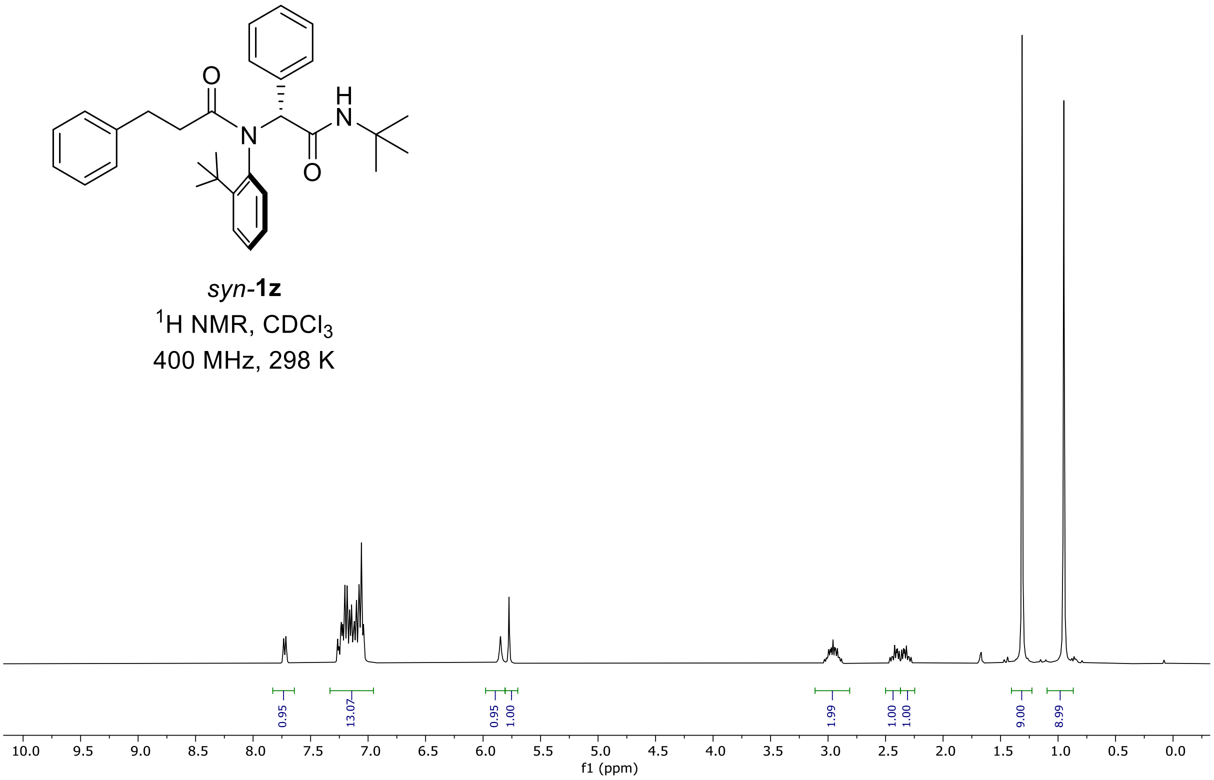
syn-1z
1H NMR, CDCl3
400 MHz, 298 K
0.95
13.07
0.95
1.00
1.99
1.00
1.00
9.00
8.99
10.0 9.5 9.0 8.5 8.0 7.5 7.0 6.5 6.0 5.5 5.0 4.5 4.0 3.5 3.0 2.5 2.0 1.5 1.0 0.5 0.0
f1 (ppm)

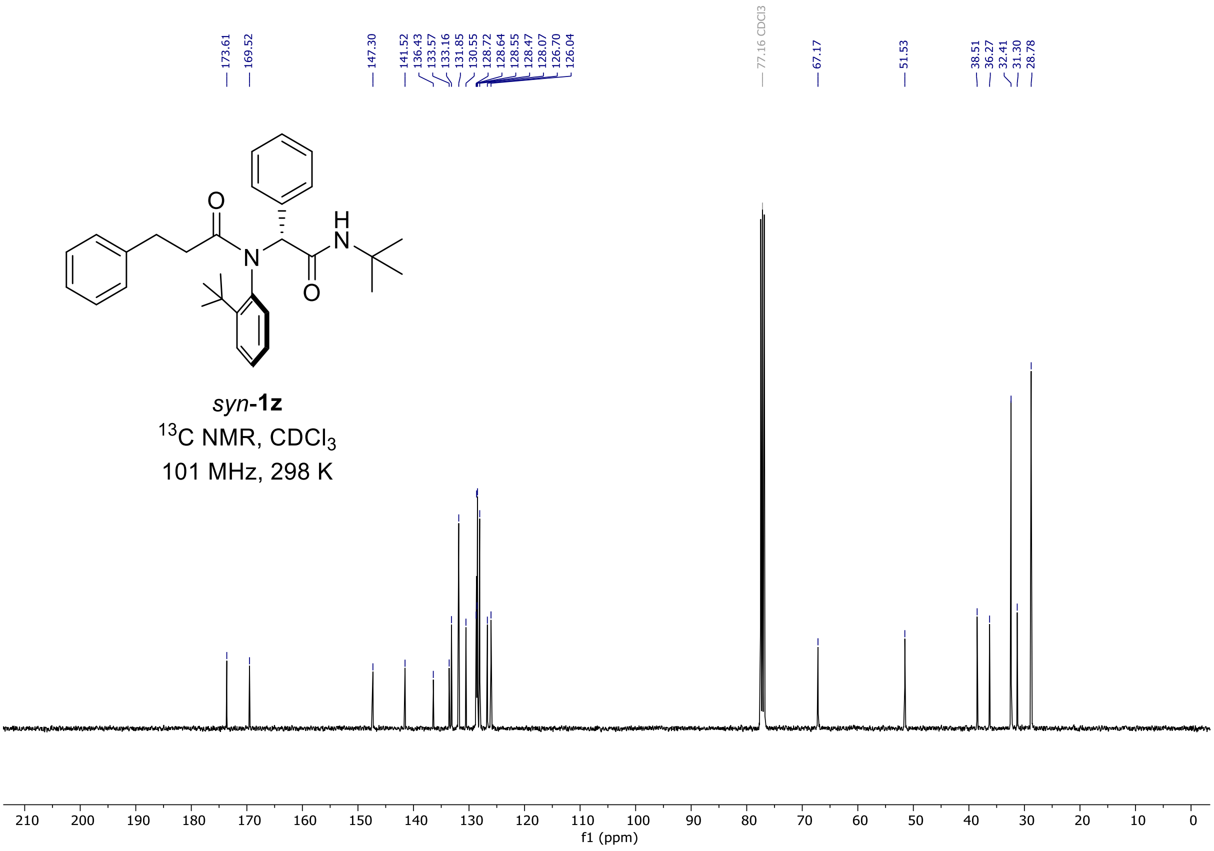
173.61
169.52
147.30
141.52
136.43
133.57
133.16
131.85
130.55
128.72
128.64
128.55
128.47
128.07
126.70
126.04
77.16 CDCl3
67.17
51.53
38.51
36.27
32.41
31.30
28.78
syn-1z
13C NMR, CDCl3
101 MHz, 298 K
210 200 190 180 170 160 150 140 130 120 110 100 90 80 70 60 50 40 30 20 10 0
f1 (ppm)

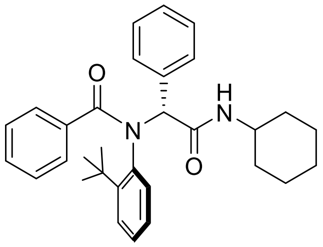

syn-**1ai**
^{1}H NMR, $CDCl_3$
400 MHz, 298 K

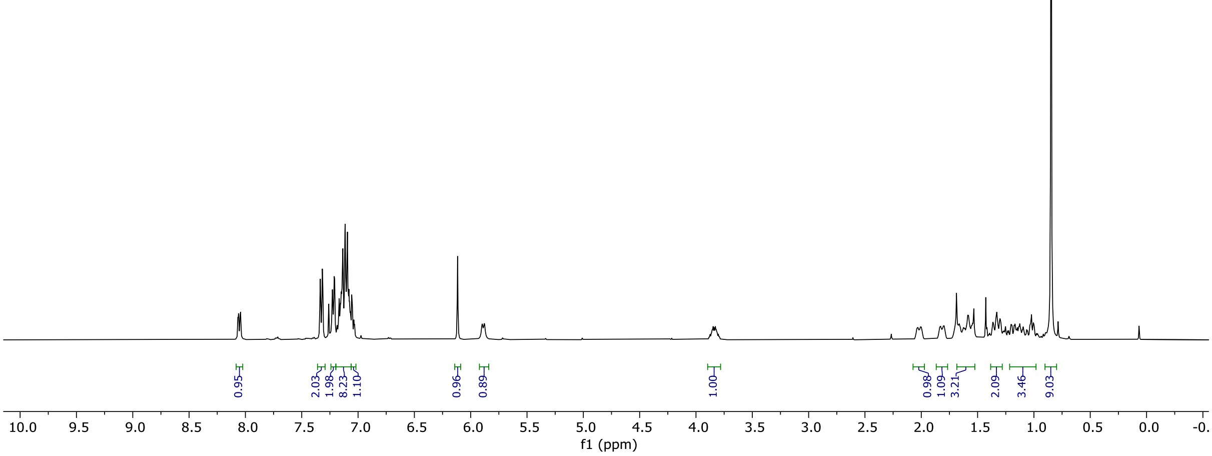

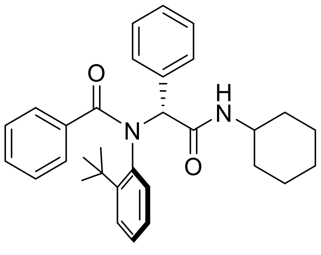

syn-**1ai**
^{13}C NMR, $CDCl_3$
101 MHz, 298 K

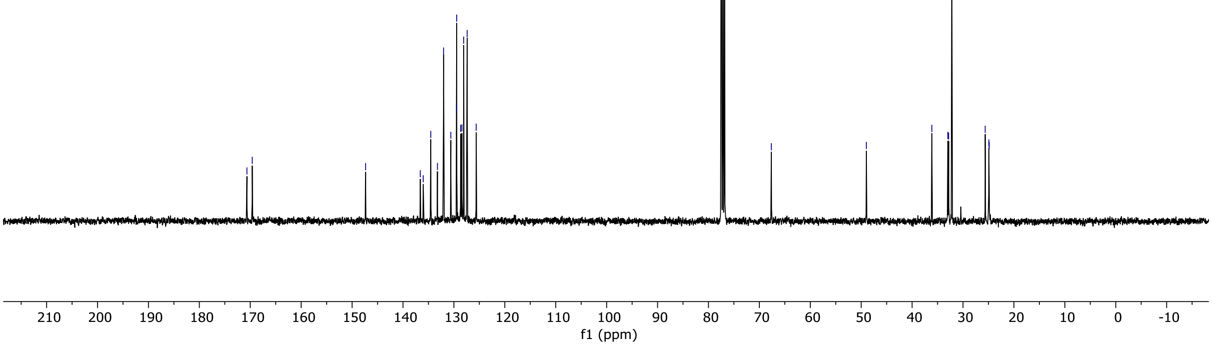

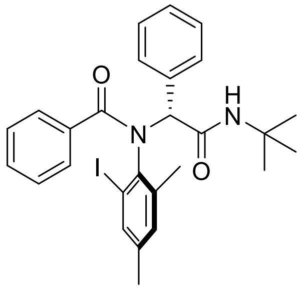

syn-**1am**

^{1}H NMR, $CDCl_3$

400 MHz, 298 K

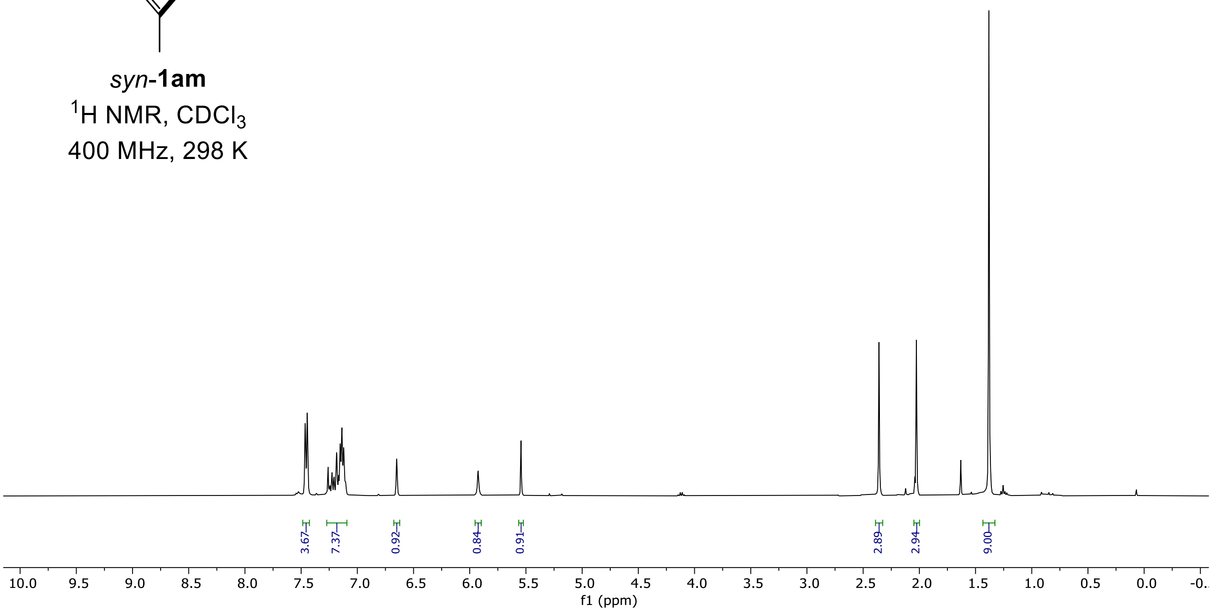

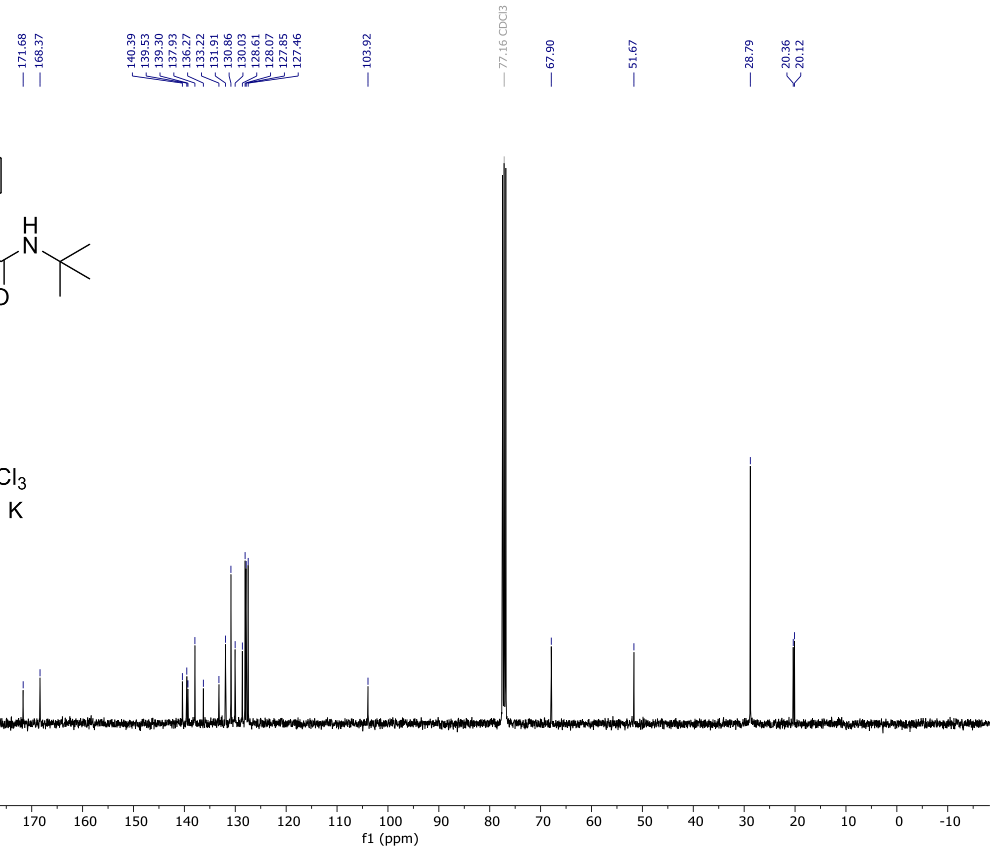

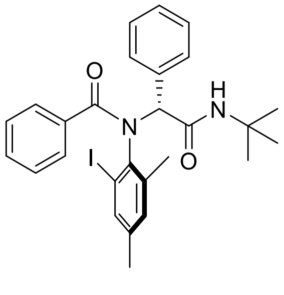

syn-**1am**

^{13}C NMR, $CDCl_3$

101 MHz, 298 K

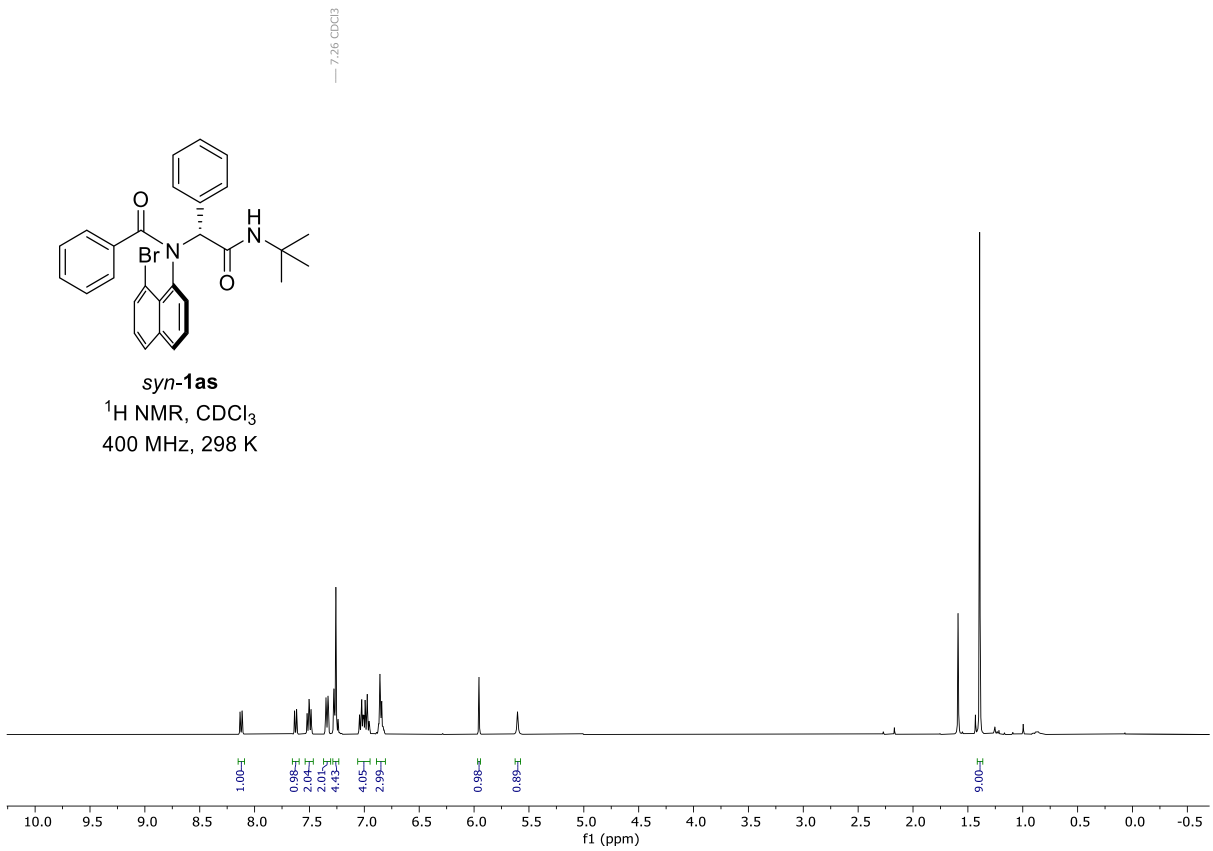
7.26 CDCl3
O
H
N
Br
O
syn-**1as**
^{1}H NMR, CDCl$_3$
400 MHz, 298 K
1.00
0.98
2.04
2.01
4.43
4.05
2.99
0.98
0.89
9.00
10.0 9.5 9.0 8.5 8.0 7.5 7.0 6.5 6.0 5.5 5.0 4.5 4.0 3.5 3.0 2.5 2.0 1.5 1.0 0.5 0.0 -0.5
f1 (ppm)

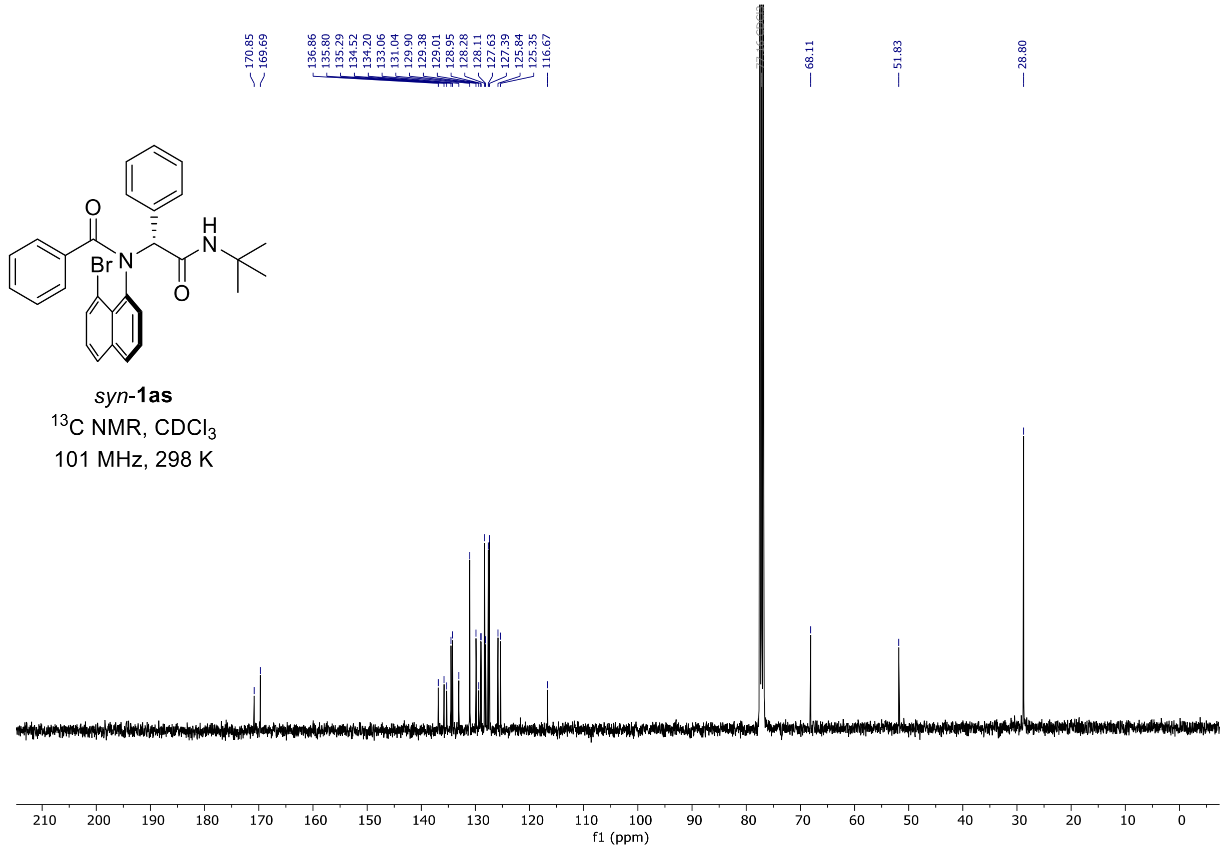
170.85
169.69
136.86
135.80
135.29
134.52
134.20
133.06
131.04
129.90
129.38
129.01
128.95
128.28
128.11
127.63
127.39
125.84
125.35
116.67
68.11
51.83
28.80
O
H
N
Br
O
syn-**1as**
^{13}C NMR, CDCl$_3$
101 MHz, 298 K
210 200 190 180 170 160 150 140 130 120 110 100 90 80 70 60 50 40 30 20 10 0
f1 (ppm)

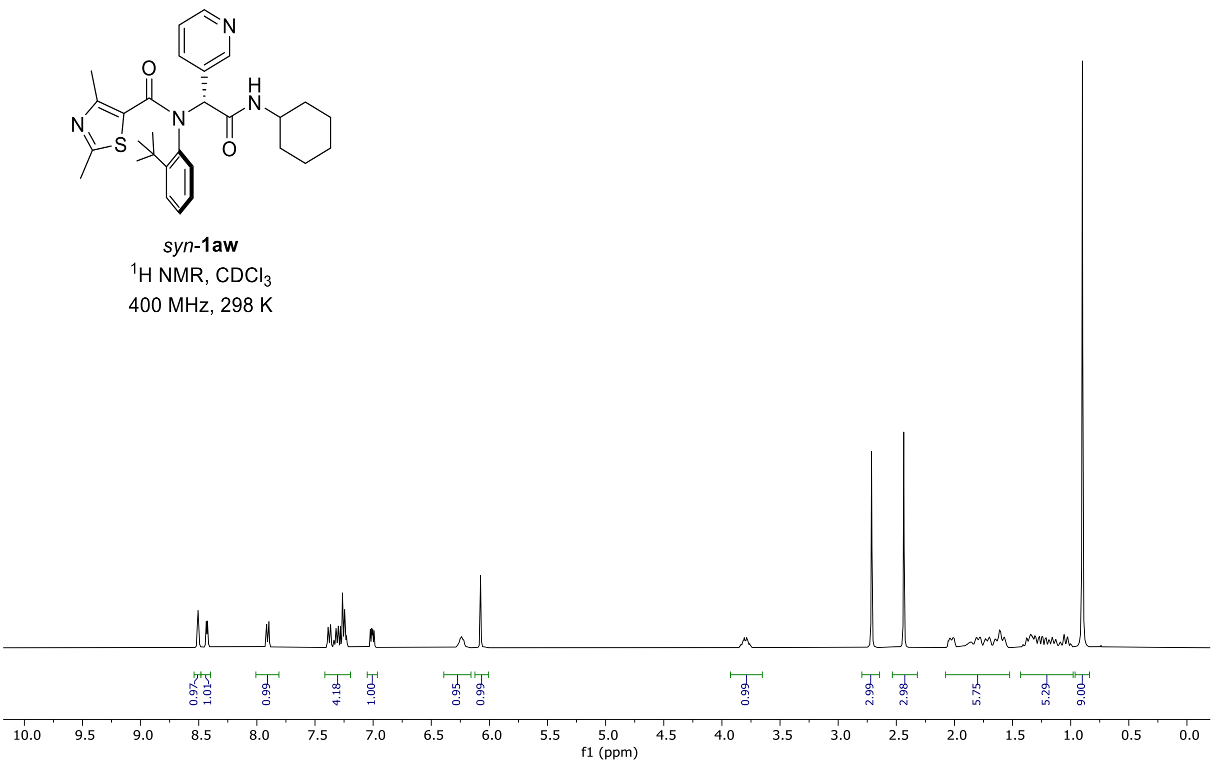
syn-1aw
1H NMR, CDCl3
400 MHz, 298 K
0.97 1.01 0.99 4.18 1.00 0.95 0.99 0.99 2.99 2.98 5.75 5.29 9.00
f1 (ppm)

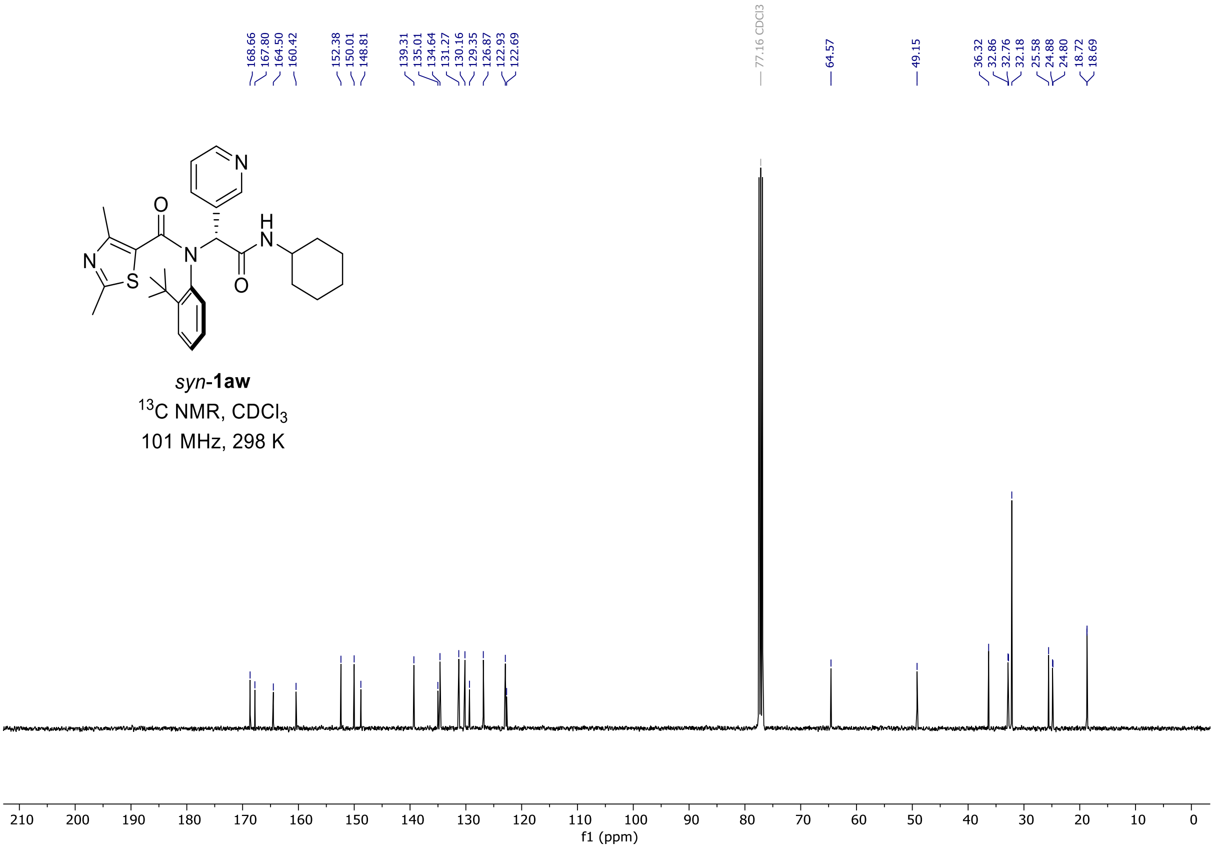
168.66 167.80 164.50 160.42 152.38 150.01 148.81 139.31 135.01 134.64 131.27 130.16 129.35 126.87 122.93 122.69
77.16 CDCl3
64.57
49.15
36.32 32.86 32.76 32.18 25.58 24.88 24.80 18.72 18.69
syn-1aw
13C NMR, CDCl3
101 MHz, 298 K
f1 (ppm)
